# Comparison of Treatments for Cocaine Use Disorder Among Adults: A Systematic Review and Meta-analysis

**DOI:** 10.1001/jamanetworkopen.2021.8049

**Published:** 2021-05-07

**Authors:** Brandon S. Bentzley, Summer S. Han, Sophie Neuner, Keith Humphreys, Kyle M. Kampman, Casey H. Halpern

**Affiliations:** 1 Department of Psychiatry and Behavioral Sciences, Stanford University, Stanford, California; 2 Department of Neurosurgery, Stanford University, Stanford, California; 3 Veterans Affairs Palo Alto Health Care System, Palo Alto, California; 4 Department of Psychiatry, University of Pennsylvania, Philadelphia

## Abstract

**Importance:**

In the US and the United Kingdom, cocaine use is the second leading cause of illicit drug overdose death. Psychosocial treatments for cocaine use disorder are limited, and no pharmacotherapy is approved for use in the US or Europe.

**Objective:**

To compare treatments for active cocaine use among adults.

**Data Sources:**

PubMed and the Cochrane Database of Systematic Reviews were searched for clinical trials published between December 31, 1995, and December 31, 2017.

**Study Selection:**

This meta-analysis was registered on Covidence.org (study 8731) on December 31, 2015. Clinical trials were included if they (1) had the term *cocaine* in the article title; (2) were published between December 31, 1995, and December 31, 2017; (3) were written in English; (4) enrolled outpatients 18 years or older with active cocaine use at baseline; and (5) reported treatment group size, treatment duration, retention rates, and urinalysis results for the presence of cocaine metabolites. A study was excluded if (1) more than 25% of participants were not active cocaine users or more than 80% of participants had negative test results for the presence of cocaine metabolites at baseline and (2) it reported only pooled urinalysis results indicating the presence of multiple substances and did not report the specific proportion of positive test results for cocaine metabolites. Multiple reviewers reached criteria consensus. Of 831 records screened, 157 studies (18.9%) met selection criteria and were included in the analysis.

**Data Extraction and Synthesis:**

This study followed the Preferred Reporting Items for Systematic Reviews and Meta-analyses (PRISMA) guideline. Search results were imported from PubMed XML into Covidence.org then Microsoft Excel. Data extraction was completed in 2 iterations to ensure fidelity. Analyses included a multilevel random-effects model, a multilevel mixed-effects meta-regression model, and sensitivity analyses. Treatments were clustered into 11 categories (psychotherapy, contingency management programs, placebo, opioids, psychostimulants, anticonvulsants, dopamine agonists, antidepressants, antipsychotics, miscellaneous medications, and other therapies). Missing data were imputed using multiple imputation by chained equations. The significance threshold for all analyses was *P* = .05. Data were analyzed using the metafor and mice packages in R software, version 3.3.2 (R Foundation for Statistical Computing). Data were analyzed from January 1, 2018, to February 28, 2021.

**Main Outcomes and Measures:**

The primary outcome was the intention-to-treat logarithm of the odds ratio (OR) of having a negative urinalysis result for the presence of cocaine metabolites at the end of each treatment period compared with baseline. The hypothesis, which was formulated after data collection, was that no treatment category would have a significant association with objective reductions in cocaine use.

**Results:**

A total of 157 studies comprising 402 treatment groups and 15 842 participants were included. Excluding other therapies, the largest treatment groups across all studies were psychotherapy (mean [SD] number of participants, 40.04 [36.88]) and contingency management programs (mean [SD] number of participants, 37.51 [25.51]). Only contingency management programs were significantly associated with an increased likelihood of having a negative test result for the presence of cocaine (OR, 2.13; 95% CI, 1.62-2.80), and this association remained significant in all sensitivity analyses.

**Conclusions and Relevance:**

In this meta-analysis, contingency management programs were associated with reductions in cocaine use among adults. Research efforts and policies that align with this treatment modality may benefit those who actively use cocaine and attenuate societal burdens.

## Key Points

**Question:**

What treatments for cocaine use disorder are associated with objective reductions in cocaine use among adults?

**Findings:**

In this meta-analysis of 157 clinical trials comprising 402 treatment groups and 15 842 participants, only contingency management programs were significantly associated with an increased likelihood of having a negative test result for the presence of cocaine, and this association remained significant in all sensitivity analyses.

**Meaning:**

The findings suggest that contingency management programs may be beneficial for the treatment of cocaine use disorder among adults who actively use cocaine.

## Teaser

This systematic review and meta-analysis uses data from clinical trials to compare treatments for cocaine use disorder and examines whether any treatment approaches are associated with reductions in cocaine use among adults who are actively using cocaine.

## Introduction

After years of decreasing rates, the prevalence of cocaine use has been increasing since 2012; cocaine is currently the second leading cause of overdose death (with opioids the first) associated with illicit drug use in the US^1,2^ and the United Kingdom.^3^ Cocaine use has taken a particular toll on certain vulnerable populations; for example, it is the leading cause of overdose death among Black persons.^4^ However, treatments for cocaine use disorders are limited and, despite the performance of many clinical trials over several decades, no pharmacotherapy has been approved by government agencies in the US or Europe. The lack of approved treatments for cocaine use disorder is in contrast to the approval of naltrexone, methadone, and buprenorphine medications for the treatment of opioid use disorder^5,6,7,8^; naltrexone, acamprosate, and disulfiram medications for the treatment of alcohol use disorder^9,10^; and varenicline, bupropion, and nicotine replacement medications for the treatment of tobacco use disorder.^11,12^

The absence of a standard treatment for cocaine use disorders has hampered clinical treatment. With no guiding prototype available, the development of new treatments has proven challenging. Furthermore, current understanding of the pathophysiologic characteristics of cocaine use disorders remains insufficient for the development of beneficial pharmacological treatments. Numerous meta-analyses have attempted to search for a signal of treatment benefit by pooling results from multiple clinical trials. However, meta-analytic investigations have reported no improvement in outcomes among those receiving anticonvulsant,^13,14,15^ antidepressant,^16^ antipsychotic,^17,18,19^ acupuncture,^20^ disulfiram,^21^ dopamine agonist,^22^ opioid,^23^ and psychostimulant^24,25,26^ therapies. Meta-analyses of psychosocial interventions have reported variable effect sizes given the heterogeneity of approaches.^23,27,28^ Meta-analyses of contingency management programs, which comprise positive reinforcement of drug abstinence, have indicated beneficial outcomes; however, these analyses have been limited to studies with distinct populations, studies with specific comparison groups,^23,29,30,31^ or studies that exclusively examined contingency management interventions.^32^ Furthermore, leaders in the field of substance use disorders continue to classify contingency management as a treatment with limited benefits,^33^ making its comparative role in the treatment of cocaine use disorder unclear. We performed a comprehensive meta-analysis of all treatments for cocaine use disorders published over 22 years, inclusive of all clinical trials and cocaine-using populations, to examine which treatment approaches, if any, were associated with a reduction in cocaine use. The hypothesis, which was formulated after data collection and based on the results reported in most previously published meta-analyses, was that no treatment category would have a significant association with objective reductions in cocaine use.

## Methods

### Search Strategy and Selection Criteria

In this systematic review and meta-analysis, all methods were conducted in accordance with the Preferred Reporting Items for Systematic Reviews and Meta-analyses (PRISMA) reporting guideline.^34^ This meta-analysis was prospectively registered on Covidence.org (study 8731) on December 31, 2015. Covidence.org was used to store search results, identify duplicates, and track screening decisions. The PubMed database was searched for clinical trials with the term *cocaine* in the article title that were published between December 31, 1995, and December 31, 2015. This search was temporally expanded and repeated on December 31, 2016, and December 31, 2017, to update the analysis with relevant studies. Only English language articles were included; this criterion excluded 6 of 831 abstracts, with only 2 of those 6 abstracts describing small studies relevant to this meta-analysis. The exact search string was as follows: cocaine[title] AND (Clinical Trial[ptyp] AND “loattrfull text”[sb] AND (“1995/12/31”[PDAT]: “2017/12/31”[PDAT]) AND English[lang]). In addition, all references within the Cochrane Database of Systematic Reviews that were identified as meta-analyses of treatments for cocaine use disorders, all references of clinical trials that were identified during full-text screening, and all references that were cited within identified references were included in the abstract screening.

All clinical trial designs were included if their goal was to assess the efficacy of a treatment for reducing cocaine use. Participants had to be 18 years or older with active cocaine use at baseline that was identified by self-report or urinalysis testing. Studies were excluded if more than 25% of participants were not active cocaine users based on self-report or if more than 80% of participants had negative test results for the presence of cocaine metabolites at baseline. Only studies that reported treatment group size, treatment duration, retention rates, and treatment outcomes using urinalysis testing for cocaine metabolites were included. Studies that reported treatment outcomes only as pooled urinalysis results across multiple drugs (ie, urinalysis results not reporting the specific proportion of negative and positive test results for the presence of cocaine metabolites) were excluded.

Study authors were not contacted, and unpublished data were not sought. Data included in the meta-analysis were extracted at the summary estimate level. One reviewer (B.S.B.) performed the search, full-text screening, data extraction, and data analysis. Two reviewers (B.S.B. and S.N.) screened all abstracts and references, and 2 reviewers (B.S.B and S.S.H.) performed the data analysis. Disagreements regarding inclusion were resolved via reconsideration by 1 reviewer (B.S.B.); however, no disagreements occurred. A detailed outline of the study protocol is available in eMethods 1 in the Supplement.

### Outcome Measures

The start and end of treatment were defined as the first and last points at which participants were exposed to the treatment; posttreatment data were not included. The primary outcome was defined as the intention-to-treat (ITT) logarithm of the odds ratio (log OR) of having a negative urinalysis result for the presence of cocaine metabolites at the end of the treatment period compared with baseline. Baseline urinalysis data were either reported directly, inferred based on the requirement of having a positive test result for cocaine at study entry, or estimated based on urinalysis results during the first week of treatment. The type of baseline data reported was coded as a dummy variable and included in the statistical analysis. For studies reporting multiple baseline types, direct baseline testing was preferred to positive test results at screening, which was in turn preferred to estimation based on the first week of treatment. Outcome urinalysis data were reported either directly as ITT outcomes or calculated based on retention rates and non-ITT outcomes. Urinalysis data from the end of treatment were either reported at the last treatment point or as the mean urinalysis result for the entire treatment period. The type of outcome data reported was also coded as a dummy variable and included in the statistical analysis. For studies reporting multiple outcome types, data from the last treatment point were preferred to mean urinalysis results across the treatment period, and direct ITT reporting was preferred to calculation of ITT outcomes based on retention and non-ITT outcomes.

### Data Extraction

All search results were imported from PubMed XML output into Covidence.org, with duplicates automatically removed during importation. Two reviewers (B.S.B. and S.N.) independently assessed references and abstracts. If both reviewers agreed that the clinical trial did or did not meet eligibility criteria, it was included or excluded, respectively. The full text of all remaining articles was obtained, and the same eligibility criteria were used to determine which, if any, articles should be excluded at this stage. Any disagreements were resolved via discussion and were ultimately decided based on the discretion of 2 reviewers (B.S.B. and C.H.H). One reviewer (B.S.B.) read each full-text article, determined whether the study met inclusion criteria, and extracted the data to a Microsoft Excel database, which was backed up continuously to offsite storage. Data extraction was completed in 2 iterations, with the second iteration ensuring the fidelity of the first.

In addition to urinalysis results and retention data, the information extracted included study characteristics (lead author, publication year, double-blindedness, randomization, and multisitedness), participant characteristics (age, sex, years of cocaine use, self-reported days of cocaine use per week, reported scores on the Addiction Severity Index drug composite subscale (score range, 0-1, with higher scores indicating more severe problems associated with drug use), and intervention details (treatment category, specific treatment, treatment dose, and duration of treatment).

Eleven treatment categories were defined based on categories used in previous systematic reviews of treatments for cocaine use disorders^13,14,15,16,17,18,19,22,23,24,25,27,28,29,31,32^; these categories comprised psychotherapy, contingency management programs, placebo, opioids, psychostimulants, anticonvulsants, dopamine agonists, antidepressants, antipsychotics, miscellaneous medications (medications that did not fit in other medication categories), and other therapies (nonmedications that did not fit in any treatment category). All treatments to which participants were simultaneously exposed in a treatment arm within a study were coded by category. For example, a single treatment group may have been treated with fluoxetine and methadone medications and cognitive behavioral therapy concomitantly, and this treatment group would have been coded in the antidepressant, psychotherapy, and opioid treatment categories.

### Statistical Analysis

All statistical analyses were performed using the metafor^35^ (meta-regression) and mice^36^ (multiple imputation by chained equations) packages in R software, version 3.3.2 (R Foundation for Statistical Computing).^37^ All R scripts with associated text output can be found in eMethods 2 and eMethods 3 in the Supplement. The escalc function within the metafor package was used to calculate the log OR (*y_i_* = *ln* [(*p*/1 − *p*)/(*q*/1 − *q*)]) and variability (*v_i_*) for each treatment group based on the group size and proportion of urinalysis results that were negative for the presence of cocaine metabolites at the start (*q*) and end (*p*) of treatment. This method took into account the number of participants in each treatment group with decreasing variability with increasing group size.

The mice package in R software was used to impute missing data. The mice package is a robust method of data imputation that creates multiple estimates for each missing value and pools the results to account for uncertainty in the imputations and to estimate accurate SEs.^38^ Predictive mean matching was used to impute missing baseline data on urinalysis results, duration of treatment, participant age, proportion of male participants, years of cocaine use, and score on the Addiction Severity Index^39^ drug composite subscale. In brief, predictive mean matching uses linear regression with all observed data from all variables to build a predictor matrix. The algorithm then finds cases with observed data in which the predicted value of the observed data point is proximal to the predicted value of the missing data point. The algorithm then selects 5 donors from the closest matches, randomly samples an observed value from one of the donors, and uses this value to impute the missing data point. Given that imputed data are always observed values within the data set, they always meet boundary criteria and have the same distribution. The type of baseline urinalysis data (inferred by positive urinalysis results for the presence of cocaine as a requirement at study entry, measured at baseline, or measured during the first week) was imputed using a bayesian polytomous regression model built from all observed data. Data imputation was performed 5 times to build 5 separate imputed data sets.

In the primary analyses, we constructed a multilevel random-effects model, with treatment groups nested within studies and studies nested within the first author (ie, multiple studies conducted by the first author would be nested together). The intraclass correlation coefficient was used to assess the effect of nesting, and the Higgins *I*^2^ statistic was used to estimate heterogeneity. Collinearity was defined as a variance inflation factor greater than 5. Statistical significance was set at *P* = .05. A multilevel mixed-effects meta-regression analysis was conducted for the data set composed of studies with complete data for baseline urinalysis results, treatment duration, proportion of male participants, and mean participant age. Data from the Addiction Severity Index were not included in the nonimputed analyses, as most studies did not report them. This analysis was then repeated for the 5 imputed data sets, and the results of the 5 analyses were pooled. These 2 analyses (multilevel without imputed data and pooled results of 5 multilevel models with imputed data) served as the primary analyses.

Sensitivity analyses were conducted by examining the results of several other random-effects models, comprising models with treatment factors only, models with retention rate as a covariate, single-level models, and models assessing the results of each of the 5 imputed data sets on an individual basis. Bias was assessed by regressing effect size against variance using the method for multilevel models described by Sterne et al^40^ and Egger et al.^41^ Data were analyzed from January 1, 2018, to February 28, 2021.

## Results

In total, 1580 records were identified, and 831 records were screened; of those, 305 full-text articles were assessed for eligibility. After exclusions, 157 studies (18.9%) with 402 treatment groups and 15 842 participants were included in the meta-analysis.^42,43,44,45,46,47,48,49,50,51,52,53,54,55,56,57,58,59,60,61,62,63,64,65,66,67,68,69,70,71,72,73,74,75,76,77,78,79,80,81,82,83,84,85,86,87,88,89,90,91,92,93,94,95,96,97,98,99,100,101,102,103,104,105,106,107,108,109,110,111,112,113,114,115,116,117,118,119,120,121,122,123,124,125,126,127,128,129,130,131,132,133,134,135,136,137,138,139,140,141,142,143,144,145,146,147,148,149,150,151,152,153,154,155,156,157,158,159,160,161,162,163,164,165,166,167,168,169,170,171,172,173,174,175,176,177,178,179,180,181,182,183,184,185,186,187,188,189,190,191,192,193,194,195,196,197,198^ The PRISMA^34^ diagram shows detailed information on the number of ineligible studies and the reasons for exclusion (Figure).

**Figure.**
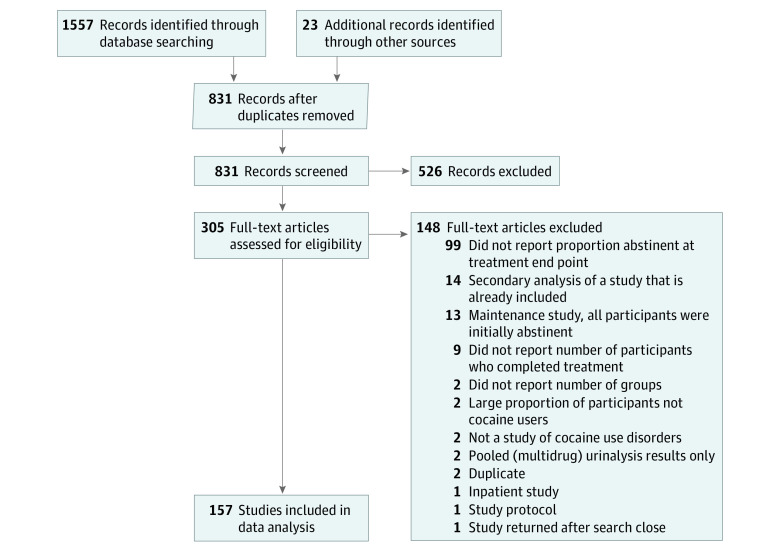
Study Selection

Table 1 includes summary statistics for each treatment category and covariate. Among 15 842 total participants, the mean (SD) age was 38.27 (4.30) years, and 10 541 of 15 262 participants (69.1%) were male. Excluding other therapies, the largest treatment groups across all studies were psychotherapy (mean [SD] number of participants, 40.04 [36.88]) and contingency management programs (mean [SD] number of participants, 37.51 [25.51]). The mean (SD) cocaine use per week was 3.25 (1.00) days, and the mean (SD) number of years of cocaine use was 11.82 (3.56). A total of 4258 of 15 842 participants (26.9%) completed treatment and were cocaine-free at the end of treatment.

**Table 1.** Summary Statistics for Treatment Categories and Covariates

Characteristic	Treatment category, mean (SD)										
	Anticonvulsants	Antidepressants	Antipsychotics	Contingency management	Dopamine agonists	Miscellaneous medications	Opioids	Other therapies	Placebo	Psychostimulants	Psychotherapy
Treatment groups, No.	20	18	12	88	20	54	159	26	96	24	330
Total participants, No.	683	574	281	3301	710	1751	5139	1805	3381	702	13213
Participants per treatment group	34.15 (20.64)	31.89 (15.84)	23.42 (10.99)	37.51 (25.51)	35.50 (19.19)	32.43 (28.86)	32.32 (18.61)	69.42 (83.73)	35.22 (25.41)	29.25 (17.00)	40.04 (36.88)
Age, y	36.52 (4.92)	36.03 (2.74)	39.46 (4.99)	38.12 (4.06)	38.65 (4.69)	39.44 (4.46)	37.64 (3.36)	38.39 (4.18)	38.26 (4.63)	38.01 (4.41)	37.82 (4.27)
No. of treatment groups with unreported values	0	0	0	1	0	1	0	3	1	0	4
Proportion of male participants	0.78 (0.15)	0.68 (0.29)	0.77 (0.19)	0.61 (0.18)	0.79 (0.12)	0.76 (0.14)	0.60 (0.19)	0.71 (0.15)	0.74 (0.18)	0.73 (0.12)	0.70 (0.20)
No. of treatment groups with unreported values	0	1	0	2	0	2	4	0	1	1	7
Cocaine use, d/wk	3.93 (1.10)	3.26 (0.86)	2.56 (0.76)	3.40 (1.13)	3.41 (0.62)	3.29 (0.95)	3.47 (0.78)	3.49 (0.59)	3.34 (0.83)	3.01 (0.68)	3.25 (0.96)
No. of treatment groups with unreported values	3	1	7	33	6	7	43	6	18	9	110
Cocaine use, y	11.51 (2.95)	9.75 (1.61)	15.10 (4.94)	10.81 (3.27)	10.54 (3.19)	12.67 (3.47)	10.51 (3.40)	11.50 (4.22)	11.89 (3.79)	12.02 (2.15)	11.36 (3.60)
No. of treatment groups with unreported values	11	14	9	37	3	15	74	10	39	11	127
Score on ASI drug composite subscale^a^	0.31 (0.08)	0.35 (0.07)	0.24 (0.01)	0.25 (0.09)	0.23 (0.02)	0.27 (0.09)	0.35 (0.10)	0.29 (0.05)	0.27 (0.07)	0.22 (0.02)	0.25 (0.09)
No. of treatment groups with unreported values	12	15	9	56	12	42	121	17	69	20	215
Proportion cocaine-free at baseline	0.26 (0.19)	0.37 (0.02)	0.33 (0.19)	0.24 (0.19)	0.30 (0.13)	0.25 (0.15)	0.19 (0.14)	0.17 (0.17)	0.28 (0.16)	0.23 (0.15)	0.28 (0.18)
No. of treatment groups with unreported values	8	16	4	43	9	37	81	12	57	11	183
Treatment duration, wk	0.61 (0.23)	0.51 (0.21)	0.44 (0.26)	0.64 (0.24)	0.43 (0.15)	0.64 (0.22)	0.72 (0.17)	0.71 (0.21)	0.58 (0.24)	0.56 (0.19)	0.60 (0.24)
No. of treatment groups with unreported values	0	0	0	5	0	0	2	0	0	0	7
Proportion completing treatment	0.48 (0.25)	0.54 (0.23)	0.35 (0.22)	0.60 (0.24)	0.48 (0.25)	0.44 (0.26)	0.41 (0.16)	0.48 (0.29)	0.40 (0.21)	0.45 (0.24)	0.45 (0.22)
No. of treatment groups with unreported values	6	5	2	51	7	23	61	8	31	13	135
Proportion completing treatment and cocaine-free at end of treatment	0.29 (0.15)	0.26 (0.12)	0.20 (0.18)	0.38 (0.19)	0.20 (0.15)	0.25 (0.18)	0.29 (0.13)	0.31 (0.18)	0.22 (0.15)	0.23 (0.11)	0.28 (0.18)
No. of treatment groups with unreported values	0	0	0	0	0	0	0	0	0	0	0

Abbreviation: ASI, Addiction Severity Index.

^a^ Score range, 0 to 1, with higher scores indicating more severe problems associated with drug use.

Table 2 includes main outcome measures for the primary analyses, which comprised multilevel models with covariates with and without imputed data. For the primary multilevel analysis with covariates, several treatments were associated with a significant increase in the ITT likelihood of producing a negative urinalysis result in the nonimputed data set; however, only contingency management programs were significant for both imputed (OR, 2.13; 95% CI, 1.62-2.80) and nonimputed (OR, 2.09; 95% CI, 1.59-2.75) data sets (Table 2). This statistical significance persisted across all sensitivity testing (Table 3). After contingency management programs, the treatment category that was significantly associated with the ITT likelihood of producing a negative urinalysis result across most analyses was opioids. Opioid agonist therapies were significantly associated with a reduction in cocaine use in 6 of 9 analyses that did not include completion rate as a covariate and in 1 of 8 analyses that included completion rate as a covariate. A total of 330 of 402 treatment groups incorporated some form of psychotherapy; however, none of the primary or sensitivity analyses indicated a significant association between psychotherapy and change in the ITT likelihood of producing a negative urinalysis. Notably, placebo was not associated with a significant change in the ITT likelihood of producing a negative urinalysis result for the imputed (OR, 1.03; 95% CI, 0.59-1.80) or nonimputed (OR, 1.48; 95% CI, 0.86-2.53) data set in the primary analyses, and this nonsignificance was also observed in all sensitivity analyses. When completion rate was added as a covariate to the primary nonimputed multilevel model, only contingency management programs remained significantly associated with outcomes (OR, 2.06; 95% CI, 1.53-2.77).

**Table 2.** Outcomes for Primary Analyses

Treatment category	Without imputed data				With imputed data			
	No.			OR (95% CI)	No.			OR (95% CI)
	Studies	Treatment groups	Participants		Studies	Treatment groups	Participants	
Anticonvulsants	14	17	570	2.32 (1.15-4.67)^a^	16	20	683	1.39 (0.59-3.29)
Antidepressants	8	15	482	1.96 (0.91-4.25)	11	18	574	1.36 (0.63-2.91)
Antipsychotics	8	11	241	1.59 (0.68-3.71)	9	12	281	1.02 (0.41-2.53)
Contingency management	38	69	2744	2.09 (1.59-2.75)^b^	50	88	3301	2.13 (1.62-2.80)^b^
Dopamine agonists	10	18	645	1.55 (0.80-3.00)	12	20	710	1.04 (0.51-2.14)
Miscellaneous medications	26	38	1455	1.64 (0.88-3.06)	37	54	1751	1.14 (0.60-2.17)
Opioids	49	142	4686	2.32 (1.54-3.49)^b^	55	159	5139	1.70 (0.98-2.94)
Other therapies	12	21	1577	2.19 (1.07-4.49)^c^	15	26	1805	1.35 (0.72-2.53)
Placebo	65	78	2889	1.48 (0.86-2.53)	80	96	3381	1.03 (0.59-1.80)
Psychostimulants	13	19	645	2.48 (1.27-4.85)^d^	17	24	702	1.74 (0.81-3.74)
Psychotherapy	98	258	10 773	1.08 (0.74-1.58)	131	330	13 213	1.18 (0.81-1.73)

Abbreviation: OR, odds ratio.

^a^ *P* = .02.

^b^ *P* < .001.

^c^ *P* = .03.

^d^ *P* = .008.

**Table 3.** Sensitivity Analyses^a^

Variable	Single-level analysis			Multilevel analysis													
	Treatments only	Treatments with covariates	Treatments with covariates plus completion rate	Treatments with covariates^b^	Treatments with covariates plus completion rate	Treatments with covariates imputed: model 1	Treatments with covariates imputed: model 2	Treatments with covariates imputed: model 3	Treatments with covariates imputed: model 4	Treatments with covariates imputed: model 5	Treatments with covariates imputed: pooled ^b^	Treatments with covariates imputed plus completion rate: model 1	Treatments with covariates imputed plus completion rate: model 2	Treatments with covariates imputed plus completion rate: model 3	Treatments with covariates imputed plus completion rate: model 4	Treatments with covariates imputed plus completion rate: model 5	Treatments with covariates imputed plus completion rate: pooled
**Treatment category**																	
Anticonvulsants	N	Y (*P* = .02)	N	Y (*P* = .02)	N	N	N	N	N	N	N	N	N	N	N	N	N
Antidepressants	Y (*P* = .001)	N	N	N	N	N	N	N	N	N	N	N	N	N	N	N	N
Antipsychotics	N	N	N	N	N	N	N	N	N	N	N	N	N	N	N	N	N
Contingency management	Y (*P* = .003)	Y (*P* < .001)	Y (*P* < .001)	Y (*P* < .001)	Y (*P* < .001)	Y (*P* < .001)	Y (*P* < .001)	Y (*P* < .001)	Y (*P* < .001)	Y (*P* < .001)	Y (*P* < .001)	Y (*P* < .001)	Y (*P* < .001)	Y (*P* < .001)	Y (*P* < .001)	Y (*P* < .001)	Y (*P* < .001)
Dopamine agonists	N	N	N	N	N	N	N	N	N	N	N	N	N	N	N	N	N
Miscellaneous medications	Y (*P* = .02)	N	N	N	N	N	N	N	N	N	N	N	N	N	N	N	N
Opioids	Y (*P* < .001)	Y (*P* < .001)	Y (*P* = .02)	Y (*P* < .001)	N	Y (*P* = .04)	N	Y (*P* = .005)	N	Y (*P* = .004)	N	N	N	N	N	N	N
Other	Y (*P* = .02)	Y (*P* = .008)	Y (*P* = .006)	Y (*P* = .03)	N	N	N	N	N	N	N	N	N	N	N	N	N
Placebo	N	N	N	N	N	N	N	N	N	N	N	N	N	N	N	N	N
Psychostimulants	N	N	Y (*P* = .02)	Y (*P* = .008)	N	Y (*P* = .01)	N	N	N	N	N	N	N	N	N	N	N
Psychotherapy	N	N	N	N	N	N	N	N	N	N	N	N	N	N	N	N	N
**Study type**																	
Double-blind	NA	Y (*P* = .01)	N	Y (*P* = .02)	N	N	N	N	N	N	N	N	N	N	N	N	N
Multisite	NA	N	N	N	N	N	N	N	N	N	N	N	N	N	N	N	N
Randomized	NA	N	N	N	N	N	N	N	N	N	N	N	N	N	N	N	N
**Baseline type**^**c**^																	
2^d^	NA	Y (*P* < .001)	Y (*P* < .001)	Y (*P* < .001)	Y (*P* < .001)	Y (*P* < .001)	Y (*P* < .001)	Y (*P* < .001)	Y (*P* < .001)	Y (*P* < .001)	Y (*P* = .002)	Y (*P* < .001)	Y (*P* < .001)	Y (*P* < .001)	Y (*P* < .001)	Y (*P* < .001)	Y (*P* < .001)
3^e^	NA	Y (*P* < .001)	Y (*P* < .001)	Y (*P* < .001)	Y (*P* < .001)	N	Y (*P* < .001)	Y (*P* < .001)	Y (*P* < .001)	N	N	N	Y (*P* < .001)	Y (*P* < .001)	Y (*P* < .001)	N (*P* < .001)	N
**Outcome type**^f^																	
2^g^	NA	Y (*P* = .001)	N	N	N	N	Y (*P* = .03)	Y (*P* = .03)	N	N	N	N	N	N	N	N	N
3^h^	NA	Y (*P* < .001)	Y (*P* = .003)	Y (*P* = .04)	N	N	Y (*P* < .001)	Y (*P* < .001)	N	Y (*P* = .005)	N	N	Y (*P* = .04)	N	N	Y (*P* = .009)	N
4^i^	NA	Y (*P* < .001)	N	N	N	N	Y (*P* < .001)	Y (*P* < .001)	Y (*P* = .02)	Y (*P* = .02)	N	Y (*P* = .04)	Y (*P* < .001)	Y (*P* < .001)	Y (*P* < .001)	Y (*P* = .002)	N
**Other variables**																	
Treatment duration	NA	N	Y (*P* = .01)	N	Y (*P* = .04)	Y (*P* = .02)	N	N	Y (*P* = .02)	N	N	Y (*P* = .005)	N	N	Y (*P* = .005)	Y (*P* = .03)	N
Male sex, %	NA	Y (*P* = .001)	Y (*P* = .02)	N	N	Y (*P* = .02)	N	Y (*P* = .009)	N	N	N	Y (*P* = .02)	N	Y (*P* = .01)	N	N	N
Age, y	NA	N	N	N	N	N	N	N	N	N	N	N	N	N	N	N	N
Baseline cocaine use, d/wk	NA	NA	NA	NA	NA	N	N	Y (*P* = .03)	N	N	N	N	N	Y (*P* = .01)	Y (*P* = .008)	N	N
Year of study publication	NA	Y (*P* = .006)	Y (*P* = .03)	N	N	N	N	N	N	N	N	N	N	N	N	N	N
Score on ASI drug composite subscale	NA	NA	NA	NA	NA	N	N	N	N	N	N	N	N	N	N	N	N
Completion rate	NA	NA	Y (*P* < .001)	NA	Y (*P* < .001)	NA	NA	NA	NA	NA	NA	Y (*P* < .001)	Y (*P* < .001)	Y (*P* < .001)	Y (*P* < .001)	Y (*P* < .001)	Y (*P* < .001)

Abbreviations: ASI, Addiction Severity Index; NA, not applicable.

^a^ Statistically significant result, Y or N.

^b^ Primary analysis.

^c^ All baseline types were compared with baseline type 1, which comprised all participants with positive urinalysis results for cocaine at screening (study inclusion criterion).

^d^ Proportion of participants with positive urinalysis results for cocaine reported at baseline.

^e^ Proportion of participants with positive urinalysis results for cocaine reported during first week of treatment;

^f^ All outcome types were compared with outcome type 1, which comprised the proportion of participants with positive urinalysis results for cocaine reported as intention-to-treat during last week of treatment.

^g^ Intention-to-treat proportion of participants with positive urinalysis results for cocaine calculated from retention rate and outcome during last week of treatment.

^h^ Mean proportion of participants with positive urinalysis results for cocaine reported as intention-to-treat during treatment.

^i^ Intention-to-treat mean proportion of participants with positive urinalysis results for cocaine calculated from retention rate and outcome during treatment.

Forest plots with unadjusted outcomes for all studies with baseline data are shown by treatment category in eFigures 1 to 11 in the Supplement. Baseline data type was the only covariate significantly associated with outcome for the primary multilevel analysis with covariates, with baseline urinalysis results that were reported before receipt of treatment being associated with lower ITT likelihood of producing a negative urinalysis result for both imputed (OR, 0.26; 95% CI, 0.13-0.55) and nonimputed (OR, 0.13; 95% CI, 0.08-0.20) data sets compared with the reference variable, which was the OR of having a positive urinalysis result for the presence of cocaine as a requirement for study entry. This finding was in contrast to baseline data type being reported as the urinalysis results for week 1 of treatment, which was significant for the nonimputed (OR, 0.11; 95% CI, 0.06-0.20) but not the imputed (OR, 0.42; 95% CI, 0.03-5.45) data set.

Heterogeneity (*I*^2^) was 80.96% in the base random-effects model without any treatment category factors or covariates, 41.79% in the model with all treatment categories and covariates (with the exception of completion rate), 22.57% in the model with completion rate also included as a covariate, 58.54% in the multilevel model with all treatment categories and covariates (with the exception of completion rate), and 49.40% in the model with completion rate also included as a covariate. The *I*^2^ ranged from 72.69% to 76.72% across the 5 imputed data sets that did not include completion rate as a covariate and from 68.35% to 72.78% for the 5 studies that included completion rate as a covariate. When data were divided into subsets for each treatment category, the *I*^2^ was greater than 50% for placebo, psychotherapy, contingency management programs, opioids, and miscellaneous medications.

The intraclass correlation coefficient was 0.498 for the multilevel model that did not include completion rate as a covariate (treatments with covariates) and 0.392 for the model that included completion rate as a covariate (treatments with covariates plus completion rate;Table 3). The intraclass correlation coefficient ranged from 0.222 to 0.608 for the 5 imputed models that did not include completion rate as a covariate (treatments with covariates imputed: models 1-5) and from 0.107 to 0.548 for the 5 imputed models that included completion rate as a covariate (treatments with covariates imputed plus completion rate: models 1-5).

Examination of funnel plots across models and across treatment categories did not indicate any major sources of bias (eFigure 12 and eFigure 13 in the Supplement), and Egger tests were nonsignificant for the full multilevel model without imputed data. However, Egger tests did indicate bias in 4 of 5 imputed data sets and in several treatment categories, including placebo (with the exception of antipsychotics, psychotherapy, psychostimulants, miscellaneous medications, and other therapies). Collinearity was not detected for any treatment category or covariate by variance inflation factor.

## Discussion

This meta-analysis was constructed to maximize inclusivity to detect a broad treatment category that was both generalizable and beneficial in reducing cocaine use. Although several treatment categories were associated with benefits in the nonimputed data set, when data were imputed to include the complete data set, only contingency management programs were consistently associated with a significant reduction in urinalysis-confirmed cocaine use. Other treatment categories were not significantly associated with this outcome. This finding is in contrast to placebo, which was consistently not associated with a significant change in objective cocaine use in any of the primary or sensitivity analyses.

The a priori hypothesis was that no treatment category would have a significant association with objective cocaine use. However, the positive association between contingency management treatment approaches and a significant reduction in objective cocaine use was not entirely unexpected. Previous meta-analyses of contingency management programs for reducing cocaine use have suggested benefits among particular clinical populations,^23,29,30^ and a 2018 high-quality meta-analysis that used comparison groups to assess psychosocial treatments for cocaine use disorders found contingency management to be the most beneficial treatment.^31^ Moreover, large-scale implementation of contingency management programs for the treatment of substance use disorders by the US Department of Veterans Affairs has indicated both clinical benefits similar to those reported in clinical trials and low costs.^199^ Given the results of our study and the fact that the Department of Veterans Affairs is the largest integrated provider of addiction services in the US,^200^ consideration of the implementation of contingency management programs on a national level or within other major health care systems in the US is warranted.

After contingency management programs, the treatment category associated with the most benefit across analyses was opioids. Opioid agonist therapies were significantly associated with a reduction in cocaine use in 6 of 9 analyses that did not include completion rate as a covariate and 1 of 8 analyses that included completion rate as a covariate, suggesting that the benefit of opioids for reducing ITT cocaine use was enhanced through an increase in retention rate. Notably, all studies that included opioid therapies (buprenorphine and methadone) as treatments were conducted among populations with concomitant opioid use disorders. These medications have been significantly associated with increases in retention rates^7^ and, consistent with our findings, the only other meta-analysis to examine the treatment of cocaine use disorder using opioid therapies did not find a significant association outside of this increased retention rate.^23^

Our analysis did not reveal a significant association between psychotherapy and reductions in cocaine use. Meta-analyses of psychosocial interventions have reported variable effect sizes that have been associated with the heterogeneity of approaches.^23,27,28^ Our analysis also did not take into account the type or dose (ie, session length and frequency) of psychotherapy provided. A total of 330 of 402 treatment groups incorporated some form of psychotherapy, and open-label as well as noncontrolled study designs were included. Hence, if a general association between psychotherapy and cocaine use was present, it would have likely been detected. However, our approach cannot rule out benefits associated with specific approaches or doses.

### Strengths and Limitations

This study has several strengths. Broad inclusivity is its major strength, as this inclusivity provided an increased likelihood of detecting an association that was consistently present in a treatment category (vs a response that was present only for a specific treatment within a category). In addition, our analyses have at least 2 other distinct aspects, which are the inclusion of all eligible studies regardless of quality and the calculation of effect sizes from baseline to end of treatment rather than the measurement of treatment benefits in comparison with a control group, even when a control group was present. Both of these approaches were used to maximize the sensitivity for detecting a beneficial treatment.

This study also has limitations. Given the study’s broad inclusivity, we were unable to resolve the effect size of specific treatments or identify the potential benefits of a specific treatment within a broad treatment category. The approaches used to maximize the sensitivity of detecting a beneficial treatment also increased the probability of a type I error, which is a major limitation; however, we decided to trade this limitation for the benefit of inclusiveness, as previous meta-analyses of treatments for cocaine use disorders have reported mostly negative results. We compensate for this limitation by reporting the results of all sensitivity analyses and limiting our conclusion to the significant association between contingency management programs and reductions in cocaine use, as contingency management was the only treatment category with positive results in both the primary analyses and all sensitivity analyses. Furthermore, study quality was not found to be associated with outcomes, and we did not find data indicating that the inclusion of low-quality studies biased the results.

## Conclusions

In this systematic review and meta-analysis, we specifically designed our approach to search for a signal of treatment benefit present among the broad treatment categories defined by previous systematic reviews. Given the largely negative results of published meta-analyses of treatments for cocaine use disorders, we expanded our search beyond the typical restrictions that lead to data exclusion. This approach allowed us to look broadly across the extant literature; however, this broad reach came at the expense of granularity and strength of conclusion. Our comprehensive analyses suggested that contingency management approaches were associated with reductions in cocaine use. Thus, there may not be a case for therapeutic pessimism regarding cocaine use disorder. Prioritizing implementation research that informs health care systems regarding beneficial and viable adoption approaches (eg, examining current limits on patient incentive programs)^201^ may produce greater public health benefits than additional efforts to assess whether contingency management programs are generally beneficial for the treatment of cocaine use disorders.

## Notes

Supplement. **eMethods 1.** Study Protocol **eMethods 2.** R Scripts With Associated Output **eMethods 3.** Model Outputs in Data Sets (Subsetted by Treatment Category) **eFigure 1.** Forest Plot Without Imputed Data: Anticonvulsants **eFigure 2.** Forest Plot Without Imputed Data: Antidepressants **eFigure 3.** Forest Plot Without Imputed Data: Antipsychotics **eFigure 4.** Forest Plot Without Imputed Data: Contingency Management **eFigure 5.** Forest Plot Without Imputed Data: Dopamine Agonists **eFigure 6.** Forest Plot Without Imputed Data: Miscellaneous Medications **eFigure 7.** Forest Plot Without Imputed Data: Opioids **eFigure 8.** Forest Plot Without Imputed Data: Other Interventions **eFigure 9.** Forest Plot Without Imputed Data: Placebo **eFigure 10.** Forest Plot Without Imputed Data: Psychostimulants **eFigure 11.** Forest Plot Without Imputed Data: Psychotherapy **eFigure 12.** Funnel Plots (Full Models) **eFigure 13.** Funnel Plots (By Treatment Category) eReferences
