# Supplementary material for: Comparison of Treatments for Cocaine Use Disorder Among Adults: A Systematic Review and Meta-analysis
Source: JAMA Netw Open. 2021 May 7;4(5):e218049. doi: 10.1001/jamanetworkopen.2021.8049 (PMC8105751; doi:10.1001/jamanetworkopen.2021.8049)
Supplement: Supplement. — eMethods 1. Study Protocol eMethods 2. R Scripts With Associated Output eMethods 3. Model Outputs in Data Sets (Subsetted by Treatment Category) eFigure 1. Forest Plot Without Imputed Data: Anticonvulsants eFigure 2. Forest Plot Without Imputed Data: Antidepressants eFigure 3. Forest Plot Without Imputed Data: Antipsychotics eFigure 4. Forest Plot Without Imputed Data: Contingency Management eFigure 5. Forest Plot Without Imputed Data: Dopamine Agonists eFigure 6. Forest Plot Without Imputed Data: Miscellaneous Medications eFigure 7. Forest Plot Without Imputed Data: Opioids eFigure 8. Forest Plot Without Imputed Data: Other Interventions eFigure 9. Forest Plot Without Imputed Data: Placebo eFigure 10. Forest Plot Without Imputed Data: Psychostimulants eFigure 11. Forest Plot Without Imputed Data: Psychotherapy eFigure 12. Funnel Plots (Full Models) eFigure 13. Funnel Plots (By Treatment Category) eReferences [file jamanetwopen-e218049-s001.pdf]

Supplementary Online Content

Bentzley BS, Han SS, Neuner S, Humphreys K, Kampman KM, Halpern CH. Comparison of treatments for cocaine use disorder among adults: a systematic review and meta-analysis. *JAMA Netw Open*. 2021;4(5):e218049. doi:10.1001/jamanetworkopen.2021.8049

eMethods 1. Study Protocol

eMethods 2. R Scripts With Associated Output

eMethods 3. Model Outputs in Data Sets (Subsetted by Treatment Category)

eFigure 1. Forest Plot Without Imputed Data: Anticonvulsants

eFigure 2. Forest Plot Without Imputed Data: Antidepressants

eFigure 3. Forest Plot Without Imputed Data: Antipsychotics

eFigure 4. Forest Plot Without Imputed Data: Contingency Management

eFigure 5. Forest Plot Without Imputed Data: Dopamine Agonists

eFigure 6. Forest Plot Without Imputed Data: Miscellaneous Medications

eFigure 7. Forest Plot Without Imputed Data: Opioids

eFigure 8. Forest Plot Without Imputed Data: Other Interventions

eFigure 9. Forest Plot Without Imputed Data: Placebo

eFigure 10. Forest Plot Without Imputed Data: Psychostimulants

eFigure 11. Forest Plot Without Imputed Data: Psychotherapy

eFigure 12. Funnel Plots (Full Models)

eFigure 13. Funnel Plots (By Treatment Category)

eReferences

This supplementary material has been provided by the authors to give readers additional information about their work.

© 2021 Bentzley BS et al. *JAMA Network Open*.

eMethods 1. Study Protocol

1) Search strategy
   a. Database: PubMED
      1} Query: cocaine[title] AND (Clinical Trial[ptyp] AND "loattrfull text"[sb] AND ("1995/12/31"[PDAT] : "2017/12/31"[PDAT]) AND English[lang])
         1. Title contains "cocaine"
         2. Article type: "Clinical Trial"
         3. Text availability: "Full text"
         4. Publication date: From 1995/12/31 to 2017/12/30 (20 years)
         5. Language: "English"
         6. Last search: 12/30/2015
         7. References returned = 733
      2} Query: cocaine[title] AND (Clinical Trial[ptyp] AND "loattrfull text"[sb] AND ("1995/12/31"[PDAT] : "2016/12/31"[PDAT]) AND English[lang])
         1. Title contains "cocaine"
         2. Article type: "Clinical Trial"
         3. Text availability: "Full text"
         4. Publication date: From 1995/12/31 to 2016/12/31 (21 years)
         5. Language: "English"
         6. Last search: 05/02/2017
         7. References returned = 747
      3} Query: cocaine[title] AND (Clinical Trial[ptyp] AND "loattrfull text"[sb] AND ("1995/12/31"[PDAT] : "2017/12/31"[PDAT]) AND English[lang])
         1. Title contains "cocaine"
         2. Article type: "Clinical Trial"
         3. Text availability: "Full text"
         4. Publication date: From 1995/12/31 to 2017/12/31 (22 years)
         5. Language: "English"
         6. Last search: 02/18/2018
         7. References returned = 775
      4} Query: cocaine[title] AND (Clinical Trial[ptyp] AND "loattrfull text"[sb] AND ("1995/12/31"[PDAT] : "2017/12/31"[PDAT]) NOT English[lang])
         1. Title contains "cocaine"
         2. Article type: "Clinical Trial"
         3. Text availability: "Full text"
         4. Publication date: From 1995/12/31 to 2017/12/31 (22 years)
         5. Language: Anything but English
         6. Last search: 12/16/2018
      5} References returned = 6
      6} After excluding duplicates these 3 searches together yielded 808 articles, indicating some articles that were returned in the original searches were no longer included in the later searches.
   b. References within Cochrane reviews on treatments for cocaine disorders were searched (6).
      1} Amato L, Minozzi S, Pani PP, Davoli M. Antipsychotic medications for cocaine dependence. Amato L, editor. Cochrane Database Syst Rev. Chichester, UK: John Wiley & Sons, Ltd; 2007;(3):CD006306.
         1. All references were included with the Pubmed search.
      2} Castells X, Casas M, Pérez-Mañá C, Roncero C, Vidal X, Capellà D. Efficacy of psychostimulant drugs for cocaine dependence. Castells X, editor. Cochrane Database Syst Rev. Chichester, UK: John Wiley & Sons, Ltd; 2010;(2):CD007380.
         1. Margolin A, Avants K, Malison RT, Kosten TR. High- and low-dose mazindol for cocaine dependence in methadone- maintained patients: a preliminary evaluation. Substance Abuse 1997;18:125–31.
         2. Perry EB, Gil R, Miles D, Brenner L, MacDougall L, Johson R, et al.Mazindol augmentation of antipsychotic treatment for schizophrenic patients with

© 2021 Bentzley BS et al. *JAMA Network Open*.

comorbid cocaine abuse or dependence: a preliminary double-blind, randomized, placebo-controlled trial. Journal of Dual Diagnosis 2004;1: 37–47.

3} Knapp WP, Soares BGO, Farrel M, Lima MS. Psychosocial interventions for cocaine and psychostimulant amphetamines related disorders. Knapp WP, editor. Cochrane Database Syst Rev. Chichester, UK: John Wiley & Sons, Ltd; 2007;(3):CD003023.

1. Magura S, Rosenblum A, Lovejoy M, Handelsman L, Foote J, Stimmel B. Neurobehavioral Treatment for Cocaine-Using Methadone Patients: A Preliminary Report. Journal of Addictive Diseases 1994;13(4):143–60.
2. Petry NM, Peirce JM, Stitzer ML, Blaine J, Roll JM, Cohen A, et al.Effect of prize-based incentives on outcomes in stimulant abusers in outpatient psychosocial treatment programs: a national drug abuse treatment clinical trials network study. Archives of General Psychiatry 2005;62(10): 1148–56.
3. Silverman K, Svikis D, Robles E, Stitzer ML, Bigelow GE. A Reinforcement-Based Therapeutic Workplace for the Treatment of Drug Abuse. Six-Month Abstinence Outcomes. Exp Clin Psychopharmacol. 2001 Feb;9(1):14-23.

4} Minozzi S, Cinquini M, Amato L, Davoli M, Farrell MF, Pani PP, et al. Anticonvulsants for cocaine dependence. Minozzi S, editor. Cochrane Database Syst Rev. Chichester, UK: John Wiley & Sons, Ltd; 2015;4:CD006754.

1. Kampan KM, Pettinati H, Lynch KG, Dackis C, Sparkman T, Weigley C, et al. A pilot trial of topiramate for the treatment of cocaine dependence. Drug and Alcohol Dependence 2004;75(3):233–40.

5} Minozzi S, Amato L, Pani PP, Solimini R, Vecchi S, De Crescenzo F, et al. Dopamine agonists for the treatment of cocaine dependence. Amato L, editor. Cochrane Database Syst Rev. Chichester, UK: John Wiley & Sons, Ltd; 2015;5:CD003352.

1. All references were included with the Pubmed search.

6} Pani PP, Trogu E, Vecchi S, Amato L. Antidepressants for cocaine dependence and problematic cocaine use. Pani PP, editor. Cochrane Database Syst Rev. Chichester, UK: John Wiley & Sons, Ltd; 2011;(12):CD002950.

1. All references were included with the Pubmed search.

7} Pani PP, Trogu E, Vacca R, Amato L, Vecchi S, Davoli M. Disulfiram for the treatment of cocaine dependence. Pani PP, editor. Cochrane Database Syst Rev. Chichester, UK: John Wiley & Sons, Ltd; 2010;(1):CD007024.

1. All references were included with the Pubmed search.

c. Screening references during full text review, the following references were added to abstract screening (17).

1} Petry NM, Alessi SM, Marx J, Austin M, Tardif M. Vouchers versus prizes: contingency management treatment of substance abusers in community settings. J Consult Clin Psychol. 2005 Dec;73(6):1005–14.

2} Petry NM, Alessi SM, Carroll KM, Hanson T, MacKinnon S, Rounsaville B, et al. Contingency management treatments: Reinforcing abstinence versus adherence with goal-related activities. J Consult Clin Psychol. 2006 Jun;74(3):592–601.

3} Carroll KM, Ball SA, Martino S, Nich C, Babuscio TA, Nuro KF, et al. Computer-assisted delivery of cognitive-behavioral therapy for addiction: a randomized trial of CBT4CBT. Am J Psychiatry. 2008 Jul;165(7):881–8. PMCID: PMC2562873

4} Beck T, Haasen C, Verthein U, Walcher S, Schuler C, Backmund M, et al. Maintenance treatment for opioid dependence with slow-release oral morphine: a randomized cross-over, non-inferiority study versus methadone. Addiction. 2014 Apr;109(4):617–26. PMCID: PMC4226326

5} Holtyn AF, Koffarnus MN, DeFulio A, Sigurdsson SO, Strain EC, Schwartz RP, et al. The therapeutic workplace to promote treatment engagement and drug abstinence in out-of-treatment injection drug users: a randomized controlled trial. Prev Med. 2014 Nov;68:62–70. PMCID: PMC4155024

6} Milby JB, Schumacher JE, Vuchinich RE, Freedman MJ, Kertesz S, Wallace D. Toward cost-effective initial care for substance-abusing homeless. Journal of Substance Abuse Treatment. 2008 Mar;34(2):180–91. PMCID: PMC2764243

7} Milby JB, Schumacher JE, Wallace D, Freedman MJ, Vuchinich RE. To house or not to house: the effects of providing housing to homeless substance abusers in treatment. Am J Public Health. 2005 Jul;95(7):1259–65. PMCID: PMC1449349

8} Milby JB, Schumacher JE, Raczynski JM, Caldwell E, Engle M, Michael M, et al. Sufficient conditions for effective treatment of substance abusing homeless persons. Drug Alcohol Depend. 1996 Dec 2;43(1-2):39–47.

© 2021 Bentzley BS et al. *JAMA Network Open*.

9} Schmitz JM, Rathnayaka N, Green CE, Moeller FG, Dougherty AE, Grabowski J. Combination of Modafinil and d-amphetamine for the Treatment of Cocaine Dependence: A Preliminary Investigation. Front Psychiatry. Frontiers; 2012;3:77. PMCID: PMC3430875
10} Nuijten M, Blanken P, Van den Brink W, Hendriks V. Modafinil in the treatment of crack-cocaine dependence in the Netherlands: Results of an open-label randomised controlled feasibility trial. J. Psychopharmacol. (Oxford). 2015 Jun;29(6):678–87.
11} Mannelli P, Patkar AA, Murray HW, Certa K, Peindl K, Mattila-Evenden M, et al. Polymorphism in the serotonin transporter gene and response to treatment in African American cocaine and alcohol-abusing individuals. Addict Biol. 2005 Sep;10(3):261–8.
12} Wechsberg WM, Lam WKK, Zule WA, Bobashev G. Efficacy of a woman-focused intervention to reduce HIV risk and increase self-sufficiency among African American crack abusers. Am J Public Health. 2004 Jul;94(7):1165–73. PMCID: PMC1448416
13} Donovan DM, Daley DC, Brigham GS, Hodgkins CC, Perl HI, Garrett SB, et al. Stimulant abuser groups to engage in 12-step: a multisite trial in the National Institute on Drug Abuse Clinical Trials Network. Journal of Substance Abuse Treatment. Elsevier; 2013 Jan;44(1):103–14. PMCID: PMC3434261
14} Winhusen TM, Brigham GS, Kropp F, Lindblad R, Gardin JG, Penn P, et al. A randomized trial of concurrent smoking-cessation and substance use disorder treatment in stimulant-dependent smokers. J Clin Psychiatry. 2014 Apr;75(4):336–43. PMCID: PMC4019678
15} Gottheil E, Thornton C, Weinstein S. Effectiveness of high versus low structure individual counseling for substance abuse. Am J Addict. 2002;11(4):279–90.
16} Thornton CC, Gottheil E, Weinstein SP, Kerachsky RS. Patient-treatment matching in substance abuse. Drug addiction severity. Journal of Substance Abuse Treatment. 1998 Nov;15(6):505–11.
17} Brown ES, Todd JP, Hu LT, Schmitz JM, Carmody TJ, Nakamura A, et al. A Randomized, Double-Blind, Placebo-Controlled Trial of Citicoline for Cocaine Dependence in Bipolar I Disorder. Am J Psychiatry. 2015 Oct;172(10):1014–21.

2) Abstract screening
    a. Performed using Covidence.org
    b. Total abstracts screened: 831 = 808 from 4 serial Pubmed searches +6 Cochrane references +17 references from references.
        1} Inclusion criteria
            1. Human subjects
            2. Testing a treatment for cocaine use disorder
            3. If uncertain, include in full text screening.
    c. Total articles identified for full text screening: 305
3) Full text screening
    a. Performed using Covidence.org
    b. Inclusion criteria
        1} Population
            1. Meets criteria for cocaine use disorder (DSM-V), cocaine dependence (DSM-IV or -III), cocaine abuse (DSM-IV or -III), or cocaine use.
            2. Age: ≥18 years.
        2} Number of participants: ≥1
        3} Outcome measures
            1. Duration of treatment is defined
            2. Intention-to-treat (ITT) proportion abstinent from cocaine based on urinalysis at the end of the treatment period is defined.
                a. Directly reported
                b. Indirectly reported by reporting proportion abstinent (or proportion urine drug screens negative) from cocaine based on urinalysis at the end of the treatment (non-ITT) as well as number of subjects retained in the study at that time.
            3. Mean intention-to-treat (ITT) proportion urine drug screens negative from cocaine during the treatment period.

© 2021 Bentzley BS et al. *JAMA Network Open*.

a. Directly reported

b. Indirectly reported by reporting proportion urine drug screens negative from cocaine across the treatment period as well as number of subjects retained in the study at the end of treatment.

c. Exclusion criteria

1} >25% subjects are not cocaine users

2} Maintenance study: >80% of subjects test negative at study start.

3} Secondary analysis of a study already included.

4} Reporting only pooled urinalysis results, i.e. proportion negative from multiple drugs without reporting the specific proportion negative for cocaine.

d. Excluded studies base on full text review: 147

1} 99 Did not report proportion coke-free (mean or at study endpoint)

2} 14 Secondary analysis of a study that is already included

3} 13 Maintenance study, all participants were initially coke-free

4} 9 Does not report number of completers

5} 2 Does not report N of each group.

6} 2 Large portion of participants not cocaine users

7} 2 Not a study of cocaine use disorders.

8} 2 Pooled urinalysis results only

9} 2 Duplicate

10} 1 Inpatient study

11} 1 Study protocol

12} 1 Study found after search close

e. Studies included in review: 157

4) Data extraction

a. Data were recorded in an Excel spread sheet.

b. If data were only available in graph form, then Adobe Illustrator was used to measure values directly from graphs.

c. Data from 402 treatment groups were extracted

d. Opioid use disorders

1} Treatment groups were categorically coded as having concomitant opioid use disorder if the study reported that the majority >50% of participants were active opioid users; reported a recent diagnosis of opioid abuse, dependence or use disorder; or reported that participants were enrolled in an opioid substitution program.

2} Similarly, if >50% of participants in a treatment group were on methadone or buprenorphine then the treatment group was categorically coded as being treated with an opioid.

© 2021 Bentzley BS et al. *JAMA Network Open*.

eMethods 2. R Scripts With Associated Output

Calculate primary outcomes and variance

```
***R script:

#Author: Brandon S Bentzley
#Project: Meta analysis of treatments for cocaine use disorders
#Date this R script was created: 2/20/2017
#Date this R script was last updated: 3/26/2018
#Description: This script uses escalc from the package metafor to calculate the log odds ratio (yi)
#and the variances (vi) for use in metaregression in a different script


# store the current directory
initial.dir<-getwd()
# change to the new directory
setwd("/Users/bsbentzley/Documents/Stanford/Malenka/2015.12.03 - DBS cocaine/Ranalysis")
# load the necessary libraries
library(gdata)
library(metafor)
# set the output file
sink("Output.out")
# load the dataset
mydata = read.csv("data.csv")


#Do analysis
    #CFE: number of participants cocaine free at end of trial
    #CPE: number of participants cocaine positive at end of trial
    #CFS: number of participants cocaine free at start of trial
    #CPS: number of participants cocaine positive at start of trial

mydata <- escalc(measure = "OR", ai = CFE, bi = CPE, ci = CFS, di = CPS, data = mydata, add = 1, to
= "only0", append = TRUE)
write.csv(mydata, file = "Calculate_yi_vi.csv")


# close the output file
sink()
# unload the libraries
detach("package:gdata")
detach("package:metafor")
# change back to the original directory
setwd(initial.dir)


***Output:

None, log odds ratios (yi) and variance (vi) output to main data table (data.xls) included in
online materials.
```

© 2021 Bentzley BS et al. *JAMA Network Open*.

*****Fixed-effects model without covariates *****

```
***R script:

#Author: Brandon S Bentzley
#Project: Meta-analysis of treatments for cocaine use disorders
#Date this R script was created: 2/20/2017
#Date this R script was last updated: 04/06/2018
#Description: This script uses rma() from the package metafor to calculate fixed effects with no
moderators

# store the current directory
initial.dir<-getwd()
# change to the new directory
setwd("/Users/bsbentzley/Documents/Stanford/Malenka/2015.12.03 - DBS cocaine/Ranalysis")
# load the necessary libraries
library(gdata)
library(metafor)
# set the output file
sink("Output.out")
# load the dataset
mydata = read.csv("data.csv", colClasses=c(
"Author"="character", "Year"="numeric", "Title"="character", "StudyID"="factor",
"Double_Blind"="factor", "Multisite"="factor", "Randomized"="factor", "SubDsrdr"="character",
"Placebo"="factor", "Psychotherapy"="factor", "ContingencyManagement"="factor", "Other"="factor",
"Opioid"="factor", "MiscMeds"="factor", "Psychostimulants"="factor", "Anticonvulsants"="factor",
"DopamineAgonists"="factor", "Antidepressants"="factor", "Antipsychotics"="factor",
"TreatmentNmbr"= "numeric", "Trtmnt1orMore"="factor", "N_start"="numeric",
"Proportion_Male"="numeric", "Mean_Age"="numeric", "Mean_daysweek"="numeric",
"Proportion_30days"="numeric", "Years_use"="numeric", "ASI_Drug"="numeric",
"TestPosScreen"="factor", "CokeFreeBase"="numeric", "CokeFreeWk1"="numeric",
"BaselineType"="factor", "CokeFreeStart"="numeric", "Complication"="character", "FU"="numeric",
"Complete"="numeric", "CokeFreeLastWk_NotITT"="numeric", "CokeFreeMean_NotITT"="numeric",
"CokeFreeLastWk_ITT"="numeric", "CokeFreeMean_ITT"="numeric", "CokeFreeOutcome_Type"="factor",
"CokeFreeOutcome_ITT"="numeric", "CFS"="numeric", "CPS"="numeric", "CFE"="numeric",
"CPE"="numeric", "yi"="numeric", "vi"="numeric"))

#Do analysis
results<-rma(yi, vi, data = mydata, method = "FE", measure = "OR")
print(results)

# close the output file
sink()
# unload the libraries
detach("package:gdata")
detach("package:metafor")
# change back to the original directory
setwd(initial.dir)


***Output:

Fixed-Effects Model (k = 314)

Test for Heterogeneity:
Q(df = 313) = 1337.9201, p-val < .0001

Model Results:

estimate       se     zval     pval    ci.lb    ci.ub
  0.0580   0.0366   1.5854   0.1129  -0.0137   0.1297

---
Signif. codes:  0 ‘***’ 0.001 ‘**’ 0.01 ‘*’ 0.05 ‘.’ 0.1 ‘ ’ 1
```

© 2021 Bentzley BS et al. *JAMA Network Open*.

***Random-effects model, single-level, without covariates ***

```
***R script:

#Author: Brandon S Bentzley
#Project: Meta-analysis of treatments for cocaine use disorders
#Date this R script was created: 2/20/2017
#Date this R script was last updated: 04/03/2018
#Description: This script uses rma() from the package metafor to calculate 1-level random effects
with no moderators. It also performs a "leave1out" analysis in which it reruns the model leaving
out 1 treatment group at a time. Metafor only allows for this with no moderators.


# store the current directory
initial.dir<-getwd()
# change to the new directory
setwd("/Users/bsbentzley/Documents/Stanford/Malenka/2015.12.03 - DBS cocaine/Ranalysis")
# load the necessary libraries
library(gdata)
library(metafor)
# set the output file
sink("Output.out")
# load the dataset
mydata = read.csv("data.csv", colClasses=c(
"Author"="character", "Year"="numeric", "Title"="character", "StudyID"="factor",
"Double_Blind"="factor", "Multisite"="factor", "Randomized"="factor", "SubDsrdr"="character",
"Placebo"="factor", "Psychotherapy"="factor", "ContingencyManagement"="factor", "Other"="factor",
"Opioid"="factor", "MiscMeds"="factor", "Psychostimulants"="factor", "Anticonvulsants"="factor",
"DopamineAgonists"="factor", "Antidepressants"="factor", "Antipsychotics"="factor",
"TreatmentNmbr"= "numeric", "Trtmnt1orMore"="factor", "N_start"="numeric",
"Proportion_Male"="numeric", "Mean_Age"="numeric", "Mean_daysweek"="numeric",
"Proportion_30days"="numeric", "Years_use"="numeric", "ASI_Drug"="numeric",
"TestPosScreen"="factor", "CokeFreeBase"="numeric", "CokeFreeWk1"="numeric",
"BaselineType"="factor", "CokeFreeStart"="numeric", "Complication"="character", "FU"="numeric",
"Complete"="numeric", "CokeFreeLastWk_NotITT"="numeric", "CokeFreeMean_NotITT"="numeric",
"CokeFreeLastWk_ITT"="numeric", "CokeFreeMean_ITT"="numeric", "CokeFreeOutcome_Type"="factor",
"CokeFreeOutcome_ITT"="numeric", "CFS"="numeric", "CPS"="numeric", "CFE"="numeric",
"CPE"="numeric", "yi"="numeric", "vi"="numeric"))


#Do analysis
results<-rma(yi, vi, data = mydata, method = "REML", measure = "OR")
print(results)

#Leave 1 out analysis
resLeaveout<-leave1out(results)
print(resLeaveout)
resLeaveoutexp<-leave1out(results, transf=exp)
print(resLeaveoutexp)

# close the output file
sink()
# unload the libraries
detach("package:gdata")
detach("package:metafor")
# change back to the original directory
setwd(initial.dir)



***Output:

Random-Effects Model (k = 314; tau^2 estimator: REML)

tau^2 (estimated amount of total heterogeneity): 1.7965 (SE = 0.1969)
tau (square root of estimated tau^2 value):      1.3403
I^2 (total heterogeneity / total variability):   80.96%
H^2 (total variability / sampling variability):  5.25
```

© 2021 Bentzley BS et al. *JAMA Network Open*.

Test for Heterogeneity:
Q(df = 313) = 1337.9201, p-val < .0001

Model Results:

estimate	se	zval	pval	ci.lb	ci.ub	
0.5045	0.0893	5.6487	<.0001	0.3295	0.6796	***

Signif. codes: 0 ‘***’ 0.001 ‘**’ 0.01 ‘*’ 0.05 ‘.’ 0.1 ‘ ’ 1

Leave 1 out sensitivity analysis:

	estimate	se	zval	pval	ci.lb	ci.ub	Q	Qp	tau2	I2	H2
3	0.5013	0.0895	5.6038	0.0000	0.3260	0.6766	1335.7488	0.0000	1.7980	81.0113	5.2663
4	0.5011	0.0895	5.5973	0.0000	0.3256	0.6765	1334.3671	0.0000	1.7995	81.0077	5.2653
5	0.4984	0.0893	5.5789	0.0000	0.3233	0.6734	1332.0826	0.0000	1.7905	80.9421	5.2472
6	0.5024	0.0896	5.6098	0.0000	0.3269	0.6780	1335.9597	0.0000	1.8022	81.0326	5.2722
9	0.5014	0.0895	5.6037	0.0000	0.3261	0.6768	1335.6687	0.0000	1.7990	81.0164	5.2677
10	0.4935	0.0889	5.5476	0.0000	0.3191	0.6678	1324.0834	0.0000	1.7699	80.7628	5.1983
11	0.5052	0.0896	5.6379	0.0000	0.3295	0.6808	1337.7523	0.0000	1.8044	81.0561	5.2787
12	0.4930	0.0889	5.5447	0.0000	0.3188	0.6673	1322.6350	0.0000	1.7681	80.7444	5.1933
13	0.4912	0.0887	5.5351	0.0000	0.3173	0.6651	1318.8276	0.0000	1.7585	80.6597	5.1705
14	0.5009	0.0895	5.5991	0.0000	0.3256	0.6763	1335.1364	0.0000	1.7980	81.0068	5.2650
15	0.4960	0.0892	5.5628	0.0000	0.3213	0.6708	1328.4176	0.0000	1.7818	80.8659	5.2263
16	0.4990	0.0894	5.5840	0.0000	0.3239	0.6742	1333.0336	0.0000	1.7924	80.9602	5.2522
17	0.4972	0.0893	5.5705	0.0000	0.3223	0.6722	1330.2591	0.0000	1.7865	80.9065	5.2374
18	0.4966	0.0892	5.5664	0.0000	0.3217	0.6715	1329.2950	0.0000	1.7841	80.8858	5.2317
19	0.5124	0.0892	5.7418	0.0000	0.3375	0.6872	1330.5937	0.0000	1.7837	80.8665	5.2264
32	0.5090	0.0896	5.6836	0.0000	0.3335	0.6846	1336.7272	0.0000	1.8010	81.0079	5.2653
33	0.5112	0.0894	5.7190	0.0000	0.3360	0.6864	1332.9231	0.0000	1.7918	80.9259	5.2427
34	0.5083	0.0896	5.6735	0.0000	0.3327	0.6839	1337.3337	0.0000	1.8029	81.0227	5.2695
36	0.5127	0.0892	5.7475	0.0000	0.3378	0.6875	1329.3107	0.0000	1.7815	80.8442	5.2204
37	0.5115	0.0894	5.7234	0.0000	0.3363	0.6866	1332.6471	0.0000	1.7903	80.9176	5.2404
38	0.5063	0.0897	5.6465	0.0000	0.3306	0.6821	1337.8812	0.0000	1.8063	81.0352	5.2729
39	0.5060	0.0897	5.6419	0.0000	0.3302	0.6818	1337.7455	0.0000	1.8067	81.0244	5.2699
40	0.5036	0.0897	5.6165	0.0000	0.3279	0.6793	1335.4430	0.0000	1.8056	81.0136	5.2669
41	0.4957	0.0891	5.5604	0.0000	0.3210	0.6704	1327.7301	0.0000	1.7803	80.8524	5.2226
42	0.4983	0.0893	5.5779	0.0000	0.3232	0.6734	1331.8161	0.0000	1.7903	80.9395	5.2465
43	0.5030	0.0895	5.6188	0.0000	0.3275	0.6784	1336.9600	0.0000	1.8013	81.0389	5.2740
44	0.4961	0.0893	5.5558	0.0000	0.3211	0.6711	1299.9203	0.0000	1.7846	80.7657	5.1990
45	0.5002	0.0894	5.5935	0.0000	0.3249	0.6754	1334.4845	0.0000	1.7958	80.9898	5.2603
46	0.5034	0.0896	5.6185	0.0000	0.3278	0.6790	1336.6914	0.0000	1.8038	81.0440	5.2754
47	0.5011	0.0895	5.6010	0.0000	0.3257	0.6764	1335.4044	0.0000	1.7980	81.0093	5.2657
48	0.5031	0.0895	5.6194	0.0000	0.3276	0.6785	1336.9947	0.0000	1.8015	81.0401	5.2743
49	0.5011	0.0895	5.6010	0.0000	0.3257	0.6764	1335.4044	0.0000	1.7980	81.0093	5.2657
50	0.5030	0.0895	5.6191	0.0000	0.3276	0.6785	1336.9797	0.0000	1.8014	81.0396	5.2741
51	0.5020	0.0895	5.6092	0.0000	0.3266	0.6774	1336.2206	0.0000	1.7999	81.0255	5.2702
54	0.4989	0.0894	5.5826	0.0000	0.3238	0.6741	1332.6627	0.0000	1.7925	80.9582	5.2516
55	0.4977	0.0893	5.5737	0.0000	0.3227	0.6727	1330.9265	0.0000	1.7884	80.9222	5.2417
56	0.4995	0.0895	5.5828	0.0000	0.3241	0.6749	1330.2809	0.0000	1.7961	80.9619	5.2526
57	0.5016	0.0896	5.5984	0.0000	0.3260	0.6772	1332.5431	0.0000	1.8020	80.9939	5.2615
58	0.5027	0.0896	5.6119	0.0000	0.3271	0.6783	1336.1227	0.0000	1.8028	81.0358	5.2731
59	0.5055	0.0897	5.6379	0.0000	0.3298	0.6813	1337.6469	0.0000	1.8063	81.0417	5.2747
60	0.5067	0.0896	5.6535	0.0000	0.3310	0.6824	1337.9142	0.0000	1.8050	81.0479	5.2765
61	0.5067	0.0896	5.6535	0.0000	0.3310	0.6824	1337.9142	0.0000	1.8050	81.0478	5.2764
62	0.5028	0.0896	5.6088	0.0000	0.3271	0.6785	1333.9696	0.0000	1.8046	81.0004	5.2633
63	0.5037	0.0897	5.6181	0.0000	0.3280	0.6795	1335.8333	0.0000	1.8057	81.0210	5.2690
64	0.5032	0.0897	5.6124	0.0000	0.3275	0.6789	1334.6920	0.0000	1.8052	81.0065	5.2650
65	0.5033	0.0897	5.6137	0.0000	0.3276	0.6790	1335.1696	0.0000	1.8051	81.0152	5.2674
66	0.4978	0.0893	5.5743	0.0000	0.3227	0.6728	1331.0936	0.0000	1.7885	80.9237	5.2421
67	0.4972	0.0893	5.5701	0.0000	0.3222	0.6721	1330.1727	0.0000	1.7862	80.9044	5.2368
68	0.4974	0.0893	5.5718	0.0000	0.3224	0.6724	1330.5647	0.0000	1.7872	80.9125	5.2390
69	0.4965	0.0892	5.5659	0.0000	0.3217	0.6714	1329.1835	0.0000	1.7838	80.8831	5.2310
70	0.5031	0.0896	5.6133	0.0000	0.3274	0.6788	1335.7314	0.0000	1.8044	81.0299	5.2715
71	0.5033	0.0896	5.6147	0.0000	0.3276	0.6789	1335.8413	0.0000	1.8046	81.0303	5.2716
80	0.5014	0.0895	5.6051	0.0000	0.3261	0.6768	1335.8651	0.0000	1.7984	81.0149	5.2673

© 2021 Bentzley BS et al. *JAMA Network Open*.

81	0.5037	0.0896	5.6229	0.0000	0.3281	0.6792	1337.1364	0.0000	1.8032	81.0481	5.2765
82	0.5032	0.0895	5.6205	0.0000	0.3277	0.6787	1337.0595	0.0000	1.8018	81.0422	5.2749
83	0.5043	0.0895	5.6325	0.0000	0.3288	0.6798	1337.6271	0.0000	1.8025	81.0498	5.2770
84	0.5008	0.0895	5.5982	0.0000	0.3255	0.6762	1335.0349	0.0000	1.7976	81.0042	5.2643
85	0.5013	0.0895	5.6024	0.0000	0.3259	0.6766	1335.5393	0.0000	1.7985	81.0129	5.2667
86	0.5096	0.0895	5.6923	0.0000	0.3341	0.6851	1336.0694	0.0000	1.7991	80.9927	5.2611
87	0.5081	0.0896	5.6685	0.0000	0.3324	0.6838	1337.4777	0.0000	1.8042	81.0129	5.2667
88	0.5061	0.0897	5.6428	0.0000	0.3303	0.6819	1337.7668	0.0000	1.8068	81.0189	5.2684
89	0.5033	0.0895	5.6257	0.0000	0.3279	0.6786	1337.3317	0.0000	1.7998	81.0327	5.2722
90	0.5043	0.0896	5.6258	0.0000	0.3286	0.6800	1337.0508	0.0000	1.8054	81.0454	5.2758
91	0.5066	0.0896	5.6531	0.0000	0.3309	0.6822	1337.9156	0.0000	1.8045	81.0519	5.2776
92	0.5031	0.0896	5.6147	0.0000	0.3275	0.6787	1336.2183	0.0000	1.8038	81.0379	5.2737
93	0.5076	0.0896	5.6645	0.0000	0.3320	0.6832	1337.7201	0.0000	1.8040	81.0374	5.2735
94	0.5067	0.0896	5.6534	0.0000	0.3310	0.6824	1337.9145	0.0000	1.8049	81.0487	5.2767
95	0.5067	0.0896	5.6534	0.0000	0.3310	0.6824	1337.9145	0.0000	1.8049	81.0487	5.2767
98	0.5127	0.0892	5.7460	0.0000	0.3378	0.6876	1325.7762	0.0000	1.7825	80.8200	5.2138
99	0.5132	0.0891	5.7577	0.0000	0.3385	0.6879	1325.7475	0.0000	1.7776	80.7964	5.2074
100	0.5068	0.0896	5.6538	0.0000	0.3311	0.6826	1337.9123	0.0000	1.8055	81.0400	5.2743
101	0.5081	0.0896	5.6688	0.0000	0.3324	0.6838	1337.4663	0.0000	1.8041	81.0136	5.2669
102	0.5125	0.0893	5.7413	0.0000	0.3375	0.6874	1328.2428	0.0000	1.7842	80.8478	5.2213
103	0.5082	0.0896	5.6718	0.0000	0.3326	0.6839	1337.3860	0.0000	1.8034	81.0209	5.2690
104	0.5086	0.0895	5.6806	0.0000	0.3331	0.6841	1337.1124	0.0000	1.8008	81.0228	5.2695
105	0.5094	0.0896	5.6867	0.0000	0.3338	0.6850	1335.9524	0.0000	1.8010	80.9774	5.2569
106	0.5122	0.0893	5.7379	0.0000	0.3373	0.6872	1330.0871	0.0000	1.7853	80.8696	5.2273
107	0.5082	0.0896	5.6715	0.0000	0.3326	0.6839	1337.3941	0.0000	1.8035	81.0201	5.2687
108	0.5099	0.0895	5.6969	0.0000	0.3344	0.6853	1335.7959	0.0000	1.7978	80.9868	5.2595
109	0.5128	0.0891	5.7550	0.0000	0.3381	0.6874	1330.2605	0.0000	1.7779	80.8327	5.2172
110	0.5098	0.0895	5.6956	0.0000	0.3344	0.6852	1335.8753	0.0000	1.7982	80.9886	5.2600
111	0.5129	0.0891	5.7574	0.0000	0.3383	0.6875	1329.9644	0.0000	1.7770	80.8245	5.2150
112	0.5098	0.0894	5.7022	0.0000	0.3346	0.6850	1335.8769	0.0000	1.7951	80.9852	5.2591
113	0.5103	0.0895	5.7006	0.0000	0.3349	0.6858	1333.4327	0.0000	1.7978	80.9282	5.2433
114	0.5085	0.0896	5.6726	0.0000	0.3328	0.6842	1336.9253	0.0000	1.8041	80.9652	5.2535
115	0.5134	0.0891	5.7611	0.0000	0.3388	0.6881	1320.9951	0.0000	1.7763	80.7592	5.1973
116	0.5031	0.0896	5.6149	0.0000	0.3275	0.6788	1336.2369	0.0000	1.8039	81.0381	5.2737
117	0.5097	0.0895	5.6931	0.0000	0.3342	0.6851	1335.9675	0.0000	1.7990	80.9900	5.2604
118	0.5059	0.0895	5.6514	0.0000	0.3304	0.6813	1337.9185	0.0000	1.8018	81.0473	5.2763
121	0.5014	0.0896	5.5958	0.0000	0.3258	0.6770	1329.3209	0.0000	1.8022	80.9556	5.2509
122	0.5053	0.0897	5.6324	0.0000	0.3294	0.6811	1336.8162	0.0000	1.8074	80.9749	5.2562
123	0.5069	0.0897	5.6541	0.0000	0.3312	0.6827	1337.9102	0.0000	1.8058	81.0299	5.2714
124	0.5034	0.0897	5.6147	0.0000	0.3277	0.6791	1335.1868	0.0000	1.8054	81.0126	5.2667
125	0.5034	0.0897	5.6149	0.0000	0.3277	0.6792	1335.1948	0.0000	1.8054	81.0119	5.2665
126	0.5059	0.0895	5.6515	0.0000	0.3304	0.6813	1337.9185	0.0000	1.8019	81.0477	5.2764
127	0.5088	0.0895	5.6830	0.0000	0.3333	0.6842	1337.0129	0.0000	1.8002	81.0200	5.2687
128	0.5105	0.0894	5.7138	0.0000	0.3354	0.6857	1334.9332	0.0000	1.7920	80.9583	5.2516
129	0.5021	0.0895	5.6096	0.0000	0.3266	0.6775	1336.2555	0.0000	1.8000	81.0265	5.2705
130	0.4982	0.0893	5.5774	0.0000	0.3231	0.6733	1331.7279	0.0000	1.7900	80.9374	5.2459
131	0.5015	0.0895	5.6044	0.0000	0.3261	0.6769	1335.7315	0.0000	1.7992	81.0180	5.2682
132	0.4998	0.0894	5.5895	0.0000	0.3245	0.6750	1333.8376	0.0000	1.7950	80.9805	5.2578
133	0.5016	0.0895	5.6049	0.0000	0.3262	0.6770	1335.7842	0.0000	1.7994	81.0194	5.2685
134	0.5030	0.0895	5.6191	0.0000	0.3276	0.6785	1336.9797	0.0000	1.8014	81.0396	5.2741
135	0.4995	0.0894	5.5878	0.0000	0.3243	0.6747	1333.6566	0.0000	1.7938	80.9725	5.2556
136	0.5011	0.0895	5.6010	0.0000	0.3257	0.6764	1335.4044	0.0000	1.7980	81.0093	5.2657
139	0.5043	0.0896	5.6276	0.0000	0.3286	0.6799	1337.3387	0.0000	1.8043	81.0522	5.2777
140	0.5016	0.0895	5.6048	0.0000	0.3262	0.6770	1335.7685	0.0000	1.7993	81.0190	5.2684
141	0.5003	0.0894	5.5941	0.0000	0.3250	0.6756	1334.4956	0.0000	1.7965	80.9940	5.2615
142	0.4999	0.0895	5.5875	0.0000	0.3246	0.6753	1332.5137	0.0000	1.7967	80.9808	5.2578
143	0.5044	0.0896	5.6285	0.0000	0.3288	0.6800	1337.3562	0.0000	1.8047	81.0522	5.2777
144	0.5089	0.0895	5.6888	0.0000	0.3336	0.6843	1336.7644	0.0000	1.7981	81.0113	5.2663
145	0.5123	0.0892	5.7420	0.0000	0.3374	0.6872	1331.1765	0.0000	1.7834	80.8707	5.2276
146	0.5062	0.0897	5.6455	0.0000	0.3304	0.6819	1337.8693	0.0000	1.8061	81.0415	5.2747
154	0.4967	0.0892	5.5673	0.0000	0.3219	0.6716	1329.5676	0.0000	1.7845	80.8898	5.2328
155	0.4987	0.0894	5.5816	0.0000	0.3236	0.6739	1332.5384	0.0000	1.7918	80.9534	5.2503
158	0.4966	0.0892	5.5665	0.0000	0.3218	0.6715	1329.3202	0.0000	1.7842	80.8864	5.2319
159	0.4949	0.0891	5.5556	0.0000	0.3203	0.6695	1326.3748	0.0000	1.7769	80.8227	5.2145
164	0.4992	0.0894	5.5851	0.0000	0.3240	0.6744	1333.1522	0.0000	1.7933	80.9662	5.2538
165	0.5003	0.0894	5.5935	0.0000	0.3250	0.6755	1334.4151	0.0000	1.7963	80.9920	5.2609
166	0.4987	0.0894	5.5816	0.0000	0.3236	0.6739	1332.5384	0.0000	1.7918	80.9534	5.2503
167	0.5009	0.0895	5.5985	0.0000	0.3255	0.6762	1335.0669	0.0000	1.7977	81.0051	5.2646
168	0.5045	0.0896	5.6271	0.0000	0.3288	0.6802	1337.0998	0.0000	1.8057	81.0440	5.2754

© 2021 Bentzley BS et al. *JAMA Network Open*.

169	0.5087	0.0895	5.6822	0.0000	0.3332	0.6842	1337.0489	0.0000	1.8004	81.0211	5.2690
170	0.5064	0.0897	5.6476	0.0000	0.3307	0.6822	1337.8920	0.0000	1.8064	81.0267	5.2706
171	0.5052	0.0897	5.6337	0.0000	0.3294	0.6810	1337.3785	0.0000	1.8066	81.0327	5.2722
172	0.4938	0.0890	5.5487	0.0000	0.3193	0.6682	1324.1103	0.0000	1.7716	80.7755	5.2017
173	0.4939	0.0890	5.5497	0.0000	0.3195	0.6684	1324.4364	0.0000	1.7724	80.7827	5.2037
176	0.5067	0.0896	5.6535	0.0000	0.3311	0.6824	1337.9140	0.0000	1.8051	81.0469	5.2762
177	0.5019	0.0896	5.6006	0.0000	0.3262	0.6775	1332.6703	0.0000	1.8027	80.9937	5.2614
178	0.5038	0.0897	5.6191	0.0000	0.3280	0.6795	1336.1923	0.0000	1.8054	81.0303	5.2716
179	0.5108	0.0895	5.7073	0.0000	0.3354	0.6862	1331.3220	0.0000	1.7960	80.8940	5.2340
180	0.5104	0.0895	5.7019	0.0000	0.3350	0.6859	1332.3441	0.0000	1.7976	80.9037	5.2366
181	0.5077	0.0895	5.6728	0.0000	0.3323	0.6831	1337.5066	0.0000	1.8006	81.0342	5.2726
182	0.5077	0.0896	5.6692	0.0000	0.3322	0.6832	1337.6189	0.0000	1.8022	81.0397	5.2742
183	0.5066	0.0897	5.6470	0.0000	0.3308	0.6825	1337.7895	0.0000	1.8075	80.8449	5.2205
184	0.5071	0.0897	5.6529	0.0000	0.3313	0.6829	1337.9160	0.0000	1.8071	80.8422	5.2198
185	0.5047	0.0897	5.6253	0.0000	0.3288	0.6805	1334.1355	0.0000	1.8076	80.8571	5.2239
186	0.5083	0.0896	5.6738	0.0000	0.3327	0.6839	1337.3243	0.0000	1.8028	81.0229	5.2695
187	0.5089	0.0895	5.6892	0.0000	0.3336	0.6843	1336.7370	0.0000	1.7980	81.0104	5.2660
188	0.5082	0.0896	5.6710	0.0000	0.3326	0.6839	1337.4062	0.0000	1.8036	81.0194	5.2685
189	0.5077	0.0896	5.6696	0.0000	0.3322	0.6832	1337.6099	0.0000	1.8021	81.0394	5.2741
190	0.5034	0.0896	5.6188	0.0000	0.3278	0.6790	1336.7553	0.0000	1.8036	81.0446	5.2756
191	0.5116	0.0894	5.7232	0.0000	0.3364	0.6868	1328.7995	0.0000	1.7910	80.8692	5.2272
192	0.5125	0.0893	5.7392	0.0000	0.3375	0.6875	1322.9174	0.0000	1.7853	80.8032	5.2092
193	0.5095	0.0896	5.6864	0.0000	0.3339	0.6852	1333.5651	0.0000	1.8017	80.8557	5.2235
194	0.5101	0.0896	5.6961	0.0000	0.3346	0.6856	1332.5772	0.0000	1.7992	80.8875	5.2322
195	0.5113	0.0894	5.7177	0.0000	0.3360	0.6866	1330.8059	0.0000	1.7927	80.8960	5.2345
196	0.4996	0.0894	5.5884	0.0000	0.3244	0.6748	1333.6791	0.0000	1.7945	80.9766	5.2567
197	0.4996	0.0894	5.5885	0.0000	0.3244	0.6749	1333.6980	0.0000	1.7945	80.9771	5.2568
198	0.5015	0.0895	5.6042	0.0000	0.3261	0.6769	1335.7216	0.0000	1.7992	81.0178	5.2681
204	0.5104	0.0896	5.6994	0.0000	0.3349	0.6859	1324.7021	0.0000	1.7986	80.6850	5.1773
205	0.5095	0.0896	5.6856	0.0000	0.3339	0.6852	1330.6002	0.0000	1.8020	80.6898	5.1786
206	0.5099	0.0896	5.6911	0.0000	0.3343	0.6855	1329.2563	0.0000	1.8007	80.7241	5.1878
210	0.5090	0.0895	5.6895	0.0000	0.3336	0.6843	1336.7191	0.0000	1.7979	81.0099	5.2659
211	0.4999	0.0894	5.5930	0.0000	0.3247	0.6751	1334.4846	0.0000	1.7946	80.9826	5.2583
212	0.5065	0.0896	5.6530	0.0000	0.3309	0.6821	1337.9160	0.0000	1.8042	81.0527	5.2778
213	0.4962	0.0892	5.5639	0.0000	0.3214	0.6710	1328.6967	0.0000	1.7826	80.8726	5.2281
214	0.4965	0.0892	5.5659	0.0000	0.3217	0.6714	1329.1835	0.0000	1.7838	80.8831	5.2310
215	0.5011	0.0896	5.5936	0.0000	0.3255	0.6767	1330.1606	0.0000	1.8012	80.9692	5.2546
216	0.5013	0.0896	5.5946	0.0000	0.3256	0.6769	1329.4706	0.0000	1.8017	80.9598	5.2521
224	0.5089	0.0896	5.6793	0.0000	0.3333	0.6845	1336.6190	0.0000	1.8026	80.9861	5.2593
225	0.5107	0.0895	5.7076	0.0000	0.3353	0.6861	1332.7439	0.0000	1.7958	80.9234	5.2420
226	0.5135	0.0891	5.7648	0.0000	0.3389	0.6880	1325.7005	0.0000	1.7744	80.7782	5.2024
227	0.5075	0.0897	5.6600	0.0000	0.3318	0.6832	1337.7867	0.0000	1.8056	81.0039	5.2642
228	0.5045	0.0897	5.6238	0.0000	0.3287	0.6803	1335.4478	0.0000	1.8071	80.9640	5.2532
229	0.5115	0.0894	5.7209	0.0000	0.3363	0.6868	1328.2143	0.0000	1.7918	80.8603	5.2248
230	0.5064	0.0897	5.6445	0.0000	0.3305	0.6822	1337.6950	0.0000	1.8075	80.9097	5.2383
231	0.5063	0.0897	5.6436	0.0000	0.3305	0.6822	1337.5972	0.0000	1.8076	80.8804	5.2302
236	0.4984	0.0893	5.5791	0.0000	0.3233	0.6735	1332.0743	0.0000	1.7908	80.9441	5.2477
237	0.4993	0.0894	5.5856	0.0000	0.3241	0.6745	1333.2161	0.0000	1.7935	80.9677	5.2542
238	0.4976	0.0893	5.5730	0.0000	0.3226	0.6726	1330.8529	0.0000	1.7878	80.9179	5.2405
239	0.4969	0.0892	5.5685	0.0000	0.3220	0.6718	1329.8424	0.0000	1.7853	80.8965	5.2346
240	0.5090	0.0896	5.6834	0.0000	0.3335	0.6846	1336.7373	0.0000	1.8011	81.0079	5.2654
241	0.5108	0.0894	5.7118	0.0000	0.3355	0.6861	1334.1640	0.0000	1.7939	80.9498	5.2493
242	0.5069	0.0897	5.6539	0.0000	0.3312	0.6826	1337.9116	0.0000	1.8056	81.0367	5.2733
243	0.5109	0.0893	5.7198	0.0000	0.3358	0.6860	1334.3563	0.0000	1.7903	80.9424	5.2473
244	0.5104	0.0894	5.7103	0.0000	0.3352	0.6855	1335.2155	0.0000	1.7931	80.9666	5.2539
245	0.5108	0.0895	5.7091	0.0000	0.3354	0.6861	1333.2381	0.0000	1.7952	80.9341	5.2450
246	0.5096	0.0896	5.6896	0.0000	0.3340	0.6851	1335.8032	0.0000	1.8002	80.9787	5.2573
247	0.5092	0.0896	5.6851	0.0000	0.3337	0.6848	1336.3551	0.0000	1.8011	80.9922	5.2610
248	0.5106	0.0895	5.7063	0.0000	0.3352	0.6860	1333.4943	0.0000	1.7960	80.9377	5.2460
249	0.5101	0.0895	5.6993	0.0000	0.3347	0.6856	1334.8670	0.0000	1.7978	80.9638	5.2532
250	0.5067	0.0896	5.6535	0.0000	0.3310	0.6824	1337.9142	0.0000	1.8050	81.0479	5.2765
251	0.5018	0.0895	5.6037	0.0000	0.3263	0.6773	1335.2336	0.0000	1.8011	81.0217	5.2692
252	0.5064	0.0897	5.6473	0.0000	0.3307	0.6822	1337.8894	0.0000	1.8064	81.0290	5.2712
253	0.4996	0.0894	5.5884	0.0000	0.3244	0.6748	1333.6791	0.0000	1.7945	80.9766	5.2567
254	0.4996	0.0894	5.5879	0.0000	0.3243	0.6748	1333.6142	0.0000	1.7943	80.9750	5.2562
255	0.5042	0.0895	5.6319	0.0000	0.3288	0.6797	1337.6084	0.0000	1.8023	81.0488	5.2767
256	0.5010	0.0895	5.6006	0.0000	0.3257	0.6764	1335.3649	0.0000	1.7979	81.0082	5.2654
257	0.4965	0.0892	5.5659	0.0000	0.3217	0.6713	1329.2465	0.0000	1.7836	80.8820	5.2307
258	0.4982	0.0893	5.5778	0.0000	0.3231	0.6733	1331.8920	0.0000	1.7899	80.9373	5.2459

© 2021 Bentzley BS et al. *JAMA Network Open*.

259	0.5042	0.0895	5.6317	0.0000	0.3287	0.6797	1337.6043	0.0000	1.8023	81.0485	5.2766
260	0.5013	0.0895	5.6025	0.0000	0.3259	0.6767	1335.5581	0.0000	1.7986	81.0134	5.2669
261	0.4991	0.0894	5.5845	0.0000	0.3239	0.6743	1333.0494	0.0000	1.7930	80.9636	5.2531
262	0.4977	0.0893	5.5743	0.0000	0.3227	0.6727	1331.1425	0.0000	1.7883	80.9232	5.2420
263	0.4986	0.0893	5.5808	0.0000	0.3235	0.6738	1332.4088	0.0000	1.7914	80.9502	5.2494
264	0.4991	0.0894	5.5845	0.0000	0.3239	0.6743	1333.0494	0.0000	1.7930	80.9636	5.2531
265	0.5035	0.0897	5.6164	0.0000	0.3278	0.6793	1335.6892	0.0000	1.8054	81.0221	5.2693
266	0.5063	0.0897	5.6468	0.0000	0.3306	0.6821	1337.8843	0.0000	1.8063	81.0332	5.2724
267	0.5061	0.0897	5.6450	0.0000	0.3304	0.6818	1337.8637	0.0000	1.8060	81.0439	5.2754
268	0.5041	0.0897	5.6217	0.0000	0.3283	0.6798	1336.3451	0.0000	1.8059	81.0274	5.2708
269	0.5035	0.0897	5.6165	0.0000	0.3278	0.6793	1335.6498	0.0000	1.8054	81.0207	5.2689
270	0.5080	0.0896	5.6672	0.0000	0.3323	0.6837	1337.5095	0.0000	1.8045	81.0067	5.2650
273	0.5105	0.0895	5.7014	0.0000	0.3350	0.6860	1323.9075	0.0000	1.7981	80.6875	5.1780
274	0.5111	0.0895	5.7120	0.0000	0.3358	0.6865	1318.3099	0.0000	1.7950	80.6699	5.1733
275	0.5125	0.0893	5.7372	0.0000	0.3374	0.6876	1302.7327	0.0000	1.7863	80.6145	5.1585
279	0.5053	0.0897	5.6361	0.0000	0.3296	0.6811	1337.5965	0.0000	1.8061	81.0456	5.2758
280	0.5104	0.0895	5.7031	0.0000	0.3350	0.6858	1334.5049	0.0000	1.7967	80.9576	5.2515
281	0.5039	0.0897	5.6194	0.0000	0.3282	0.6797	1335.5935	0.0000	1.8062	81.0059	5.2648
282	0.5095	0.0895	5.6898	0.0000	0.3340	0.6850	1336.1523	0.0000	1.7999	80.9929	5.2612
283	0.5149	0.0888	5.8008	0.0000	0.3409	0.6888	1317.9136	0.0000	1.7576	80.6284	5.1622
284	0.5137	0.0891	5.7668	0.0000	0.3391	0.6883	1320.0758	0.0000	1.7739	80.7413	5.1925
285	0.5125	0.0893	5.7399	0.0000	0.3375	0.6875	1325.8506	0.0000	1.7849	80.8289	5.2162
286	0.5080	0.0896	5.6674	0.0000	0.3323	0.6837	1337.5042	0.0000	1.8045	81.0076	5.2653
287	0.5065	0.0897	5.6476	0.0000	0.3307	0.6822	1337.8925	0.0000	1.8064	81.0261	5.2704
288	0.5084	0.0896	5.6741	0.0000	0.3328	0.6840	1337.3136	0.0000	1.8028	81.0231	5.2696
292	0.5060	0.0897	5.6442	0.0000	0.3303	0.6817	1337.8538	0.0000	1.8059	81.0471	5.2762
293	0.5034	0.0896	5.6169	0.0000	0.3278	0.6791	1336.3020	0.0000	1.8045	81.0380	5.2737
294	0.4994	0.0894	5.5866	0.0000	0.3242	0.6746	1333.4314	0.0000	1.7937	80.9704	5.2550
295	0.4988	0.0894	5.5819	0.0000	0.3236	0.6739	1332.6454	0.0000	1.7918	80.9535	5.2503
296	0.4954	0.0891	5.5592	0.0000	0.3208	0.6701	1327.5920	0.0000	1.7790	80.8427	5.2199
297	0.4985	0.0893	5.5798	0.0000	0.3234	0.6736	1332.2287	0.0000	1.7909	80.9457	5.2482
298	0.4997	0.0894	5.5893	0.0000	0.3245	0.6749	1333.8755	0.0000	1.7945	80.9782	5.2571
299	0.5001	0.0894	5.5929	0.0000	0.3248	0.6754	1334.4085	0.0000	1.7955	80.9878	5.2598
300	0.5061	0.0897	5.6445	0.0000	0.3303	0.6818	1337.8583	0.0000	1.8059	81.0458	5.2759
301	0.5045	0.0897	5.6277	0.0000	0.3288	0.6802	1337.1416	0.0000	1.8057	81.0443	5.2755
302	0.5034	0.0896	5.6154	0.0000	0.3277	0.6791	1335.6485	0.0000	1.8051	81.0238	5.2698
303	0.5035	0.0897	5.6161	0.0000	0.3278	0.6793	1335.3489	0.0000	1.8056	81.0124	5.2666
306	0.4949	0.0891	5.5562	0.0000	0.3203	0.6695	1326.7666	0.0000	1.7768	80.8237	5.2148
307	0.4968	0.0892	5.5680	0.0000	0.3219	0.6717	1329.7988	0.0000	1.7847	80.8920	5.2334
308	0.4974	0.0893	5.5722	0.0000	0.3225	0.6724	1330.7256	0.0000	1.7871	80.9129	5.2392
309	0.5000	0.0894	5.5919	0.0000	0.3248	0.6753	1334.2128	0.0000	1.7956	80.9869	5.2595
310	0.5019	0.0895	5.6086	0.0000	0.3265	0.6773	1336.1744	0.0000	1.7997	81.0242	5.2699
311	0.5042	0.0895	5.6317	0.0000	0.3287	0.6797	1337.6043	0.0000	1.8023	81.0485	5.2766
312	0.5059	0.0895	5.6514	0.0000	0.3304	0.6813	1337.9185	0.0000	1.8019	81.0476	5.2764
313	0.5043	0.0895	5.6322	0.0000	0.3288	0.6798	1337.6194	0.0000	1.8024	81.0494	5.2769
314	0.5043	0.0895	5.6323	0.0000	0.3288	0.6798	1337.6213	0.0000	1.8024	81.0495	5.2769
315	0.5021	0.0895	5.6096	0.0000	0.3266	0.6775	1336.2555	0.0000	1.8000	81.0265	5.2705
317	0.5054	0.0897	5.6323	0.0000	0.3295	0.6812	1335.4324	0.0000	1.8080	80.7981	5.2078
318	0.5060	0.0897	5.6402	0.0000	0.3301	0.6818	1337.5166	0.0000	1.8074	80.9693	5.2547
319	0.5021	0.0896	5.6069	0.0000	0.3266	0.6777	1335.6406	0.0000	1.8017	81.0278	5.2709
320	0.5000	0.0895	5.5882	0.0000	0.3246	0.6754	1332.6866	0.0000	1.7969	80.9830	5.2585
321	0.5066	0.0896	5.6532	0.0000	0.3310	0.6823	1337.9151	0.0000	1.8047	81.0507	5.2773
322	0.5017	0.0895	5.6020	0.0000	0.3261	0.6772	1334.9101	0.0000	1.8010	81.0182	5.2682
323	0.5063	0.0896	5.6524	0.0000	0.3307	0.6818	1337.9172	0.0000	1.8035	81.0531	5.2779
324	0.5053	0.0896	5.6389	0.0000	0.3297	0.6809	1337.7725	0.0000	1.8047	81.0560	5.2787
325	0.5128	0.0892	5.7510	0.0000	0.3380	0.6875	1329.4743	0.0000	1.7800	80.8374	5.2185
326	0.5106	0.0895	5.7074	0.0000	0.3353	0.6860	1333.8766	0.0000	1.7955	80.9459	5.2482
327	0.5085	0.0896	5.6771	0.0000	0.3329	0.6840	1337.2253	0.0000	1.8019	81.0242	5.2699
328	0.5108	0.0893	5.7169	0.0000	0.3357	0.6859	1334.6135	0.0000	1.7913	80.9499	5.2493
329	0.5103	0.0894	5.7090	0.0000	0.3351	0.6855	1335.3197	0.0000	1.7935	80.9697	5.2548
330	0.5092	0.0895	5.6875	0.0000	0.3337	0.6847	1336.5694	0.0000	1.7999	81.0062	5.2649
331	0.5087	0.0895	5.6813	0.0000	0.3332	0.6842	1337.0864	0.0000	1.8007	81.0221	5.2693
332	0.5069	0.0897	5.6539	0.0000	0.3312	0.6826	1337.9119	0.0000	1.8055	81.0380	5.2737
333	0.5063	0.0897	5.6467	0.0000	0.3306	0.6821	1337.8835	0.0000	1.8063	81.0336	5.2725
334	0.5091	0.0896	5.6825	0.0000	0.3335	0.6847	1336.4673	0.0000	1.8018	80.9904	5.2605
335	0.5092	0.0896	5.6854	0.0000	0.3337	0.6848	1336.3395	0.0000	1.8011	80.9923	5.2610
336	0.5120	0.0893	5.7343	0.0000	0.3370	0.6870	1331.1042	0.0000	1.7866	80.8852	5.2316
337	0.5039	0.0897	5.6194	0.0000	0.3282	0.6797	1335.5935	0.0000	1.8062	81.0059	5.2648
338	0.5064	0.0897	5.6469	0.0000	0.3306	0.6821	1337.8852	0.0000	1.8063	81.0325	5.2722

© 2021 Bentzley BS et al. *JAMA Network Open*.

339	0.5031	0.0896	5.6122	0.0000	0.3274	0.6788	1335.0230	0.0000	1.8048	81.0156	5.2675
340	0.5058	0.0897	5.6407	0.0000	0.3301	0.6816	1337.7176	0.0000	1.8066	81.0310	5.2717
341	0.5000	0.0894	5.5912	0.0000	0.3247	0.6752	1334.1315	0.0000	1.7954	80.9848	5.2590
342	0.5006	0.0894	5.5967	0.0000	0.3253	0.6759	1334.8696	0.0000	1.7971	81.0000	5.2632
347	0.5065	0.0896	5.6529	0.0000	0.3309	0.6821	1337.9161	0.0000	1.8042	81.0528	5.2778
348	0.4994	0.0894	5.5871	0.0000	0.3242	0.6746	1333.5588	0.0000	1.7935	80.9699	5.2548
349	0.5141	0.0889	5.7795	0.0000	0.3397	0.6884	1322.5851	0.0000	1.7679	80.7195	5.1866
350	0.5119	0.0894	5.7288	0.0000	0.3368	0.6870	1329.0571	0.0000	1.7889	80.8707	5.2276
351	0.5129	0.0892	5.7513	0.0000	0.3381	0.6877	1327.0205	0.0000	1.7802	80.8194	5.2136
352	0.5102	0.0895	5.6999	0.0000	0.3348	0.6857	1334.3515	0.0000	1.7978	80.9510	5.2496
353	0.5004	0.0895	5.5930	0.0000	0.3250	0.6757	1334.1346	0.0000	1.7971	80.9947	5.2617
354	0.5090	0.0895	5.6899	0.0000	0.3336	0.6843	1336.6974	0.0000	1.7978	81.0091	5.2657
355	0.5043	0.0895	5.6322	0.0000	0.3288	0.6798	1337.6194	0.0000	1.8024	81.0494	5.2769
356	0.5003	0.0894	5.5940	0.0000	0.3250	0.6755	1334.5491	0.0000	1.7960	80.9915	5.2608
357	0.5059	0.0895	5.6514	0.0000	0.3304	0.6813	1337.9185	0.0000	1.8019	81.0476	5.2764
358	0.5030	0.0895	5.6191	0.0000	0.3276	0.6785	1336.9797	0.0000	1.8014	81.0396	5.2741
359	0.5127	0.0892	5.7464	0.0000	0.3379	0.6876	1326.2568	0.0000	1.7823	80.8231	5.2146
360	0.5093	0.0896	5.6853	0.0000	0.3337	0.6849	1336.0158	0.0000	1.8014	80.9754	5.2564
361	0.5043	0.0895	5.6321	0.0000	0.3288	0.6797	1337.6147	0.0000	1.8024	81.0491	5.2768
362	0.5042	0.0895	5.6314	0.0000	0.3287	0.6796	1337.5933	0.0000	1.8022	81.0479	5.2765
363	0.5028	0.0895	5.6177	0.0000	0.3274	0.6783	1336.8944	0.0000	1.8010	81.0367	5.2733
364	0.4976	0.0893	5.5726	0.0000	0.3226	0.6727	1330.3850	0.0000	1.7885	80.9202	5.2411
365	0.5016	0.0895	5.6036	0.0000	0.3262	0.6771	1335.4423	0.0000	1.8003	81.0206	5.2689
366	0.5015	0.0895	5.6008	0.0000	0.3260	0.6770	1334.7923	0.0000	1.8005	81.0156	5.2675
367	0.5004	0.0895	5.5919	0.0000	0.3250	0.6758	1333.5018	0.0000	1.7979	80.9937	5.2614
368	0.5041	0.0896	5.6267	0.0000	0.3285	0.6798	1337.3030	0.0000	1.8041	81.0517	5.2775
369	0.4991	0.0894	5.5795	0.0000	0.3238	0.6744	1329.4422	0.0000	1.7947	80.9503	5.2494
370	0.5022	0.0895	5.6105	0.0000	0.3267	0.6776	1336.3308	0.0000	1.8003	81.0286	5.2711
373	0.5021	0.0896	5.6055	0.0000	0.3265	0.6777	1335.2234	0.0000	1.8020	81.0242	5.2699
374	0.4997	0.0895	5.5858	0.0000	0.3244	0.6751	1331.9757	0.0000	1.7963	80.9750	5.2562
375	0.5040	0.0896	5.6257	0.0000	0.3284	0.6796	1337.2588	0.0000	1.8039	81.0509	5.2773
376	0.4978	0.0893	5.5751	0.0000	0.3228	0.6728	1331.4023	0.0000	1.7884	80.9249	5.2424
377	0.5043	0.0895	5.6323	0.0000	0.3288	0.6798	1337.6213	0.0000	1.8024	81.0495	5.2769
378	0.4975	0.0893	5.5726	0.0000	0.3225	0.6724	1330.8005	0.0000	1.7873	80.9148	5.2397
379	0.5043	0.0895	5.6327	0.0000	0.3289	0.6798	1337.6343	0.0000	1.8026	81.0502	5.2771
380	0.5014	0.0895	5.6036	0.0000	0.3260	0.6768	1335.6600	0.0000	1.7989	81.0161	5.2676
381	0.4977	0.0893	5.5741	0.0000	0.3227	0.6727	1331.0956	0.0000	1.7882	80.9221	5.2417
382	0.4947	0.0891	5.5547	0.0000	0.3202	0.6693	1326.2288	0.0000	1.7759	80.8152	5.2125
383	0.4969	0.0892	5.5685	0.0000	0.3220	0.6718	1329.8811	0.0000	1.7852	80.8959	5.2345
384	0.5003	0.0894	5.5940	0.0000	0.3250	0.6755	1334.5491	0.0000	1.7960	80.9915	5.2608
385	0.5001	0.0894	5.5924	0.0000	0.3248	0.6754	1334.2764	0.0000	1.7958	80.9885	5.2600
386	0.5010	0.0895	5.6000	0.0000	0.3257	0.6764	1335.2273	0.0000	1.7983	81.0091	5.2657
387	0.5000	0.0894	5.5912	0.0000	0.3247	0.6753	1334.0732	0.0000	1.7957	80.9863	5.2594
388	0.5126	0.0892	5.7463	0.0000	0.3378	0.6875	1329.0854	0.0000	1.7821	80.8452	5.2206
389	0.5076	0.0896	5.6632	0.0000	0.3319	0.6832	1337.7424	0.0000	1.8045	81.0334	5.2724
394	0.5107	0.0894	5.7092	0.0000	0.3354	0.6860	1334.2087	0.0000	1.7948	80.9519	5.2499
395	0.5095	0.0895	5.6928	0.0000	0.3341	0.6849	1336.3532	0.0000	1.7984	81.0011	5.2635
396	0.5046	0.0897	5.6272	0.0000	0.3288	0.6803	1336.9019	0.0000	1.8063	81.0318	5.2720
397	0.5059	0.0897	5.6408	0.0000	0.3301	0.6816	1337.7206	0.0000	1.8066	81.0304	5.2716
398	0.5131	0.0892	5.7506	0.0000	0.3382	0.6880	1297.8421	0.0000	1.7810	80.6090	5.1570
399	0.5163	0.0882	5.8562	0.0000	0.3435	0.6890	1312.7108	0.0000	1.7272	80.3784	5.0964
400	0.5162	0.0882	5.8538	0.0000	0.3434	0.6890	1313.2026	0.0000	1.7286	80.3911	5.0997
401	0.5092	0.0896	5.6837	0.0000	0.3336	0.6848	1336.1325	0.0000	1.8017	80.9755	5.2564
402	0.5071	0.0897	5.6544	0.0000	0.3313	0.6828	1337.9056	0.0000	1.8062	81.0053	5.2646

© 2021 Bentzley BS et al. *JAMA Network Open*.

***Random-effects model, single-level, treatment group moderators ***

```
***R script:

#Author: Brandon S Bentzley
#Project: Meta analysis of treatments for cocaine use disorders
#Date this R script was created: 2/20/2017
#Date this R script was last updated: 04/17/2018
#Description: This script uses rma() from the package metafor to calculate random effects with
treatment group moderators only

# store the current directory
initial.dir<-getwd()
# change to the new directory
setwd("/Users/bsbentzley/Documents/Stanford/Malenka/2015.12.03 - DBS cocaine/Ranalysis")
# load the necessary libraries
library(gdata)
library(metafor)
# set the output file
sink("Output.out")
options(width=120)
# load the dataset and define variable data types
mydata = read.csv("data.csv", colClasses=c(
"Author"="character", "Year"="numeric", "Title"="character", "StudyID"="factor",
"Double_Blind"="factor", "Multisite"="factor", "Randomized"="factor", "SubDsrdr"="character",
"Placebo"="factor", "Psychotherapy"="factor", "ContingencyManagement"="factor", "Other"="factor",
"Opioid"="factor", "MiscMeds"="factor", "Psychostimulants"="factor", "Anticonvulsants"="factor",
"DopamineAgonists"="factor", "Antidepressants"="factor", "Antipsychotics"="factor",
"TreatmentNmbr"= "numeric", "Trtmnt1orMore"="factor", "N_start"="numeric",
"Proportion_Male"="numeric", "Mean_Age"="numeric", "Mean_daysweek"="numeric",
"Proportion_30days"="numeric", "Years_use"="numeric", "ASI_Drug"="numeric",
"TestPosScreen"="factor", "CokeFreeBase"="numeric", "CokeFreeWk1"="numeric",
"BaselineType"="factor", "CokeFreeStart"="numeric", "Complication"="character", "FU"="numeric",
"Complete"="numeric", "CokeFreeLastWk_NotITT"="numeric", "CokeFreeMean_NotITT"="numeric",
"CokeFreeLastWk_ITT"="numeric", "CokeFreeMean_ITT"="numeric", "CokeFreeOutcome_Type"="factor",
"CokeFreeOutcome_ITT"="numeric", "CFS"="numeric", "CPS"="numeric", "CFE"="numeric",
"CPE"="numeric", "yi"="numeric", "vi"="numeric"))

#Use this to output data types for the variables for testing
#print(str(mydata))

#Do analysis
#Other mods:
results<-rma(yi, vi,
    mods =  ~ factor(Anticonvulsants) + factor(Antidepressants) + factor(Antipsychotics) +
factor(ContingencyManagement)
    + factor(DopamineAgonists) + factor(MiscMeds) + factor(Opioid) + factor(Other) +
factor(Placebo)
    + factor(Psychotherapy) + factor(Psychostimulants),
    data = mydata, method = "REML", measure = "OR")
print(results)


# close the output file
sink()
# unload the libraries
detach("package:gdata")
detach("package:metafor")
# change back to the original directory
setwd(initial.dir)

***Output:


Mixed-Effects Model (k = 314; tau^2 estimator: REML)

tau^2 (estimated amount of residual heterogeneity):     1.2715 (SE = 0.1555)
tau (square root of estimated tau^2 value):             1.1276
```

© 2021 Bentzley BS et al. *JAMA Network Open*.

```
I^2 (residual heterogeneity / unaccounted variability): 74.40%
H^2 (unaccounted variability / sampling variability):   3.91
R^2 (amount of heterogeneity accounted for):            29.22%

Test for Residual Heterogeneity:
QE(df = 302) = 962.0227, p-val < .0001

Test of Moderators (coefficient(s) 2,3,4,5,6,7,8,9,10,11,12):
QM(df = 11) = 89.5220, p-val < .0001

Model Results:

                                estimate      se     zval    pval    ci.lb   ci.ub
intrcpt                          -0.3339  0.2908  -1.1483  0.2508  -0.9038  0.2360
factor(Anticonvulsants)1          0.7008  0.3849   1.8206  0.0687  -0.0537  1.4553     .
factor(Antidepressants)1          1.3898  0.4212   3.2994  0.0010   0.5642  2.2155   ***
factor(Antipsychotics)1          -0.6881  0.4750  -1.4486  0.1474  -1.6191  0.2429
factor(ContingencyManagement)1    0.6296  0.2128   2.9591  0.0031   0.2126  1.0466    **
factor(DopamineAgonists)1        -0.0453  0.3656  -0.1240  0.9014  -0.7620  0.6713
factor(MiscMeds)1                 0.6824  0.2881   2.3687  0.0179   0.1177  1.2470     *
factor(Opioid)1                   1.1518  0.1741   6.6154  <.0001   0.8106  1.4931   ***
factor(Other)1                    0.8896  0.3657   2.4328  0.0150   0.1729  1.6063     *
factor(Placebo)1                  0.2931  0.2332   1.2569  0.2088  -0.1639  0.7501
factor(Psychotherapy)1           -0.1917  0.2223  -0.8625  0.3884  -0.6273  0.2439
factor(Psychostimulants)1         0.4786  0.3612   1.3252  0.1851  -0.2293  1.1865

---
Signif. codes:  0 '***' 0.001 '**' 0.01 '*' 0.05 '.' 0.1 ' ' 1
```

© 2021 Bentzley BS et al. *JAMA Network Open*.

*****Random-effects model, single-level, all covariates, with collinearity diagnostics*****

```
***R script:

#Author: Brandon S Bentzley
#Project: Meta analysis of treatments for cocaine use disorders
#Date this R script was created: 2/20/2017
#Date this R script was last updated: 04/24/2018
#Description: This script uses rma() from the package metafor to calculate random effects with
treatment and other moderators.
#Note: Complete = Proportion of participants who completed the trial is currently commented out
with a #

# store the current directory
initial.dir<-getwd()
# change to the new directory
setwd("/Users/bsbentzley/Documents/Stanford/Malenka/2015.12.03 - DBS cocaine/Ranalysis")
# load the necessary libraries
library(gdata)
library(metafor)
library(mctest)
# set the output file
sink("Output.out")
options(width=120)
# load the dataset and define variable data types
mydata = read.csv("Data.csv", colClasses=c(
"Author"="character", "Year"="numeric", "Title"="character", "StudyID"="factor",
"Double_Blind"="factor", "Multisite"="factor", "Randomized"="factor", "SubDsrdr"="character",
"Placebo"="factor", "Psychotherapy"="factor", "ContingencyManagement"="factor", "Other"="factor",
"Opioid"="factor", "MiscMeds"="factor", "Psychostimulants"="factor", "Anticonvulsants"="factor",
"DopamineAgonists"="factor", "Antidepressants"="factor", "Antipsychotics"="factor",
"TreatmentNmbr"= "numeric", "Trtmnt1orMore"="factor", "N_start"="numeric",
"Proportion_Male"="numeric", "Mean_Age"="numeric", "Mean_daysweek"="numeric",
"Proportion_30days"="numeric", "Years_use"="numeric", "ASI_Drug"="numeric",
"TestPosScreen"="factor", "CokeFreeBase"="numeric", "CokeFreeWk1"="numeric",
"BaselineType"="factor", "CokeFreeStart"="numeric", "Complication"="character", "FU"="numeric",
"Complete"="numeric", "CokeFreeLastWk_NotITT"="numeric", "CokeFreeMean_NotITT"="numeric",
"CokeFreeLastWk_ITT"="numeric", "CokeFreeMean_ITT"="numeric", "CokeFreeOutcome_Type"="factor",
"CokeFreeOutcome_ITT"="numeric", "CFS"="numeric", "CPS"="numeric", "CFE"="numeric",
"CPE"="numeric", "yi"="numeric", "vi"="numeric"))

#Do analysis
#Other mods:
results<-rma(yi, vi,
    mods = ~ factor(Anticonvulsants) + factor(Antidepressants) + factor(Antipsychotics)
    + factor(ContingencyManagement)+ factor(DopamineAgonists) + factor(MiscMeds)
    + factor(Opioid) + factor(Other) + factor(Placebo)
    + factor(Psychotherapy) + factor(Psychostimulants)
    + factor(Double_Blind) + factor(Multisite) + factor(Randomized)
    + factor(BaselineType) + factor(CokeFreeOutcome_Type)
    + FU + Year + Proportion_Male + Mean_Age + Complete,  #Complete included in 2nd output
    data = mydata, method = "REML", measure = "OR")
print(results)

#Calculte I2 http://www.metafor-project.org/doku.php/tips:i2_multilevel_multivariate
X <- model.matrix(results)
W <- diag(1/mydata$vi)
    #Only include rows with values (Not NAs)
    rowsInclude =as.numeric(row.names(X))
    W2=W[rowsInclude, rowsInclude]
P <- W2 - W2 %*% X %*% solve(t(X) %*% W2 %*% X) %*% t(X) %*% W2
Isquared <- 100 * results$tau2 / (results$tau2 + (results$k-results$p)/sum(diag(P)))
print(paste0("I2 = ", Isquared), quote = FALSE)

#Calc collinear
X <- X[,-c(1)] #for deleting columns; this takes out the intrcp=1 for all groups
Y <- mydata$yi
Y2=Y[rowsInclude] #only include data with outcomes
```

© 2021 Bentzley BS et al. *JAMA Network Open*.

```
coldiag <- mctest(X, Y2, type="i") #perform colinearity diagnostics
print(coldiag)

# close the output file
sink()
closeAllConnections()
# unload the libraries
detach("package:gdata")
detach("package:metafor")
detach("package:mctest")
# change back to the original directory
setwd(initial.dir)


***Output 1: (Without completion rate as covariate and collinearity diagnostics)


Mixed-Effects Model (k = 300; tau^2 estimator: REML)

tau^2 (estimated amount of residual heterogeneity):     0.3260 (SE = 0.0712)
tau (square root of estimated tau^2 value):             0.5709
I^2 (residual heterogeneity / unaccounted variability): 41.79%
H^2 (unaccounted variability / sampling variability):   1.72
R^2 (amount of heterogeneity accounted for):            80.62%

Test for Residual Heterogeneity:
QE(df = 276) = 470.0981, p-val < .0001

Test of Moderators (coefficient(s) 2,3,4,5,6,7,8,9,10,11,12,13,14,15,16,17,18,19,20,21,22,23,24):
QM(df = 23) = 420.2026, p-val < .0001

Model Results:

                                 estimate       se      zval    pval      ci.lb     ci.ub
intrcpt                          -77.2125  28.0742   -2.7503  0.0060  -132.2369  -22.1882   **
factor(Anticonvulsants)1           0.7648   0.3246    2.3560  0.0185     0.1286    1.4010    *
factor(Antidepressants)1           0.4163   0.3638    1.1445  0.2524    -0.2966    1.1293
factor(Antipsychotics)1           -0.3023   0.3889   -0.7774  0.4369    -1.0645    0.4599
factor(ContingencyManagement)1     0.5649   0.1595    3.5406  0.0004     0.2522    0.8776  ***
factor(DopamineAgonists)1          0.2358   0.3094    0.7622  0.4460    -0.3706    0.8422
factor(MiscMeds)1                  0.4526   0.2776    1.6304  0.1030    -0.0915    0.9967
factor(Opioid)1                    1.0543   0.1360    7.7533  <.0001     0.7878    1.3208  ***
factor(Other)1                     0.7482   0.2805    2.6677  0.0076     0.1985    1.2980   **
factor(Placebo)1                   0.1735   0.2442    0.7104  0.4775    -0.3052    0.6521
factor(Psychotherapy)1            -0.1550   0.1719   -0.9019  0.3671    -0.4918    0.1818
factor(Psychostimulants)1          0.5343   0.3102    1.7227  0.0849    -0.0736    1.1422    .
factor(Double_Blind)1             -0.5472   0.2202   -2.4846  0.0130    -0.9789   -0.1156    *
factor(Multisite)1                -0.0527   0.1987   -0.2651  0.7910    -0.4421    0.3367
factor(Randomized)1               -0.1859   0.3823   -0.4862  0.6268    -0.9353    0.5635
factor(BaselineType)2             -2.2027   0.1690  -13.0349  <.0001    -2.5339   -1.8715  ***
factor(BaselineType)3             -2.3502   0.2156  -10.9034  <.0001    -2.7727   -1.9278  ***
factor(CokeFreeOutcome_Type)2     -0.4909   0.1526   -3.2165  0.0013    -0.7901   -0.1918   **
factor(CokeFreeOutcome_Type)3     -1.0311   0.2719   -3.7921  0.0001    -1.5640   -0.4982  ***
factor(CokeFreeOutcome_Type)4     -0.4405   0.2113   -2.0846  0.0371    -0.8547   -0.0263    *
FU                                 0.0078   0.0109    0.7191  0.4721    -0.0135    0.0291
Year                               0.0390   0.0141    2.7657  0.0057     0.0114    0.0667   **
Proportion_Male                    1.2896   0.4050    3.1845  0.0014     0.4959    2.0833   **
Mean_Age                           0.0076   0.0164    0.4661  0.6412    -0.0244    0.0397

---
Signif. codes:  0 '***' 0.001 '**' 0.01 '*' 0.05 '.' 0.1 ' ' 1

[1] I2 = 41.7905199672199

Call:
imcdiag(x = x, y = y, method = method, corr = FALSE, vif = vif,
    tol = tol, conf = conf, cvif = cvif, leamer = leamer, all = all)
```

© 2021 Bentzley BS et al. *JAMA Network Open*.

```
All Individual Multicollinearity Diagnostics Result

                                  VIF    TOL      Wi      Fi Leamer   CVIF Klein
factor(Anticonvulsants)1       1.7849 0.5603  9.8824 10.3903 0.7485 1.7776     0
factor(Antidepressants)1       1.6124 0.6202  7.7103  8.1066 0.7875 1.6058     0
factor(Antipsychotics)1        1.6230 0.6161  7.8446  8.2478 0.7849 1.6165     0
factor(ContingencyManagement)1 1.4550 0.6873  5.7289  6.0234 0.8290 1.4491     0
factor(DopamineAgonists)1      1.8983 0.5268 11.3100 11.8913 0.7258 1.8906     0
factor(MiscMeds)1              2.6489 0.3775 20.7613 21.8284 0.6144 2.6382     1
factor(Opioid)1                1.5834 0.6316  7.3452  7.7227 0.7947 1.5770     0
factor(Other)1                 1.6085 0.6217  7.6612  8.0550 0.7885 1.6019     0
factor(Placebo)1               3.7901 0.2638 35.1295 36.9352 0.5137 3.7747     1
factor(Psychotherapy)1         1.3170 0.7593  3.9909  4.1961 0.8714 1.3116     0
factor(Psychostimulants)1      1.9133 0.5227 11.4992 12.0903 0.7230 1.9055     0
factor(Double_Blind)1          3.6602 0.2732 33.4946 35.2162 0.5227 3.6454     1
factor(Multisite)1             1.3985 0.7150  5.0177  5.2756 0.8456 1.3928     0
factor(Randomized)1            1.5271 0.6549  6.6361  6.9772 0.8092 1.5209     0
factor(BaselineType)2          1.6795 0.5954  8.5552  8.9949 0.7716 1.6727     0
factor(BaselineType)3          1.4615 0.6842  5.8106  6.1093 0.8272 1.4556     0
factor(CokeFreeOutcome_Type)2  1.7800 0.5618  9.8205 10.3253 0.7495 1.7728     0
factor(CokeFreeOutcome_Type)3  1.4339 0.6974  5.4635  5.7443 0.8351 1.4281     0
factor(CokeFreeOutcome_Type)4  1.4448 0.6922  5.6001  5.8879 0.8320 1.4389     0
FU                             1.2395 0.8068  3.0151  3.1701 0.8982 1.2344     0
Year                           1.7177 0.5822  9.0363  9.5008 0.7630 1.7107     0
Proportion_Male                1.4192 0.7046  5.2782  5.5495 0.8394 1.4135     0
Mean_Age                       1.4301 0.6992  5.4156  5.6939 0.8362 1.4243     0

1 --> COLLINEARITY is detected by the test
0 --> COLLINEARITY is not detected by the test

factor(Antidepressants)1 , factor(Antipsychotics)1 , factor(DopamineAgonists)1 , factor(MiscMeds)1
, factor(Placebo)1 , factor(Psychotherapy)1 , factor(Psychostimulants)1 , factor(Multisite)1 ,
factor(Randomized)1 , factor(CokeFreeOutcome_Type)2 , factor(CokeFreeOutcome_Type)4 , FU , Mean_Age
, coefficient(s) are non-significant may be due to multicollinearity

R-square of y on all x: 0.5556

* use method argument to check which regressors may be the reason of collinearity
```

```
***Output 2: (With completion rate as covariate)

Mixed-Effects Model (k = 295; tau^2 estimator: REML)

tau^2 (estimated amount of residual heterogeneity):     0.1391 (SE = 0.0513)
tau (square root of estimated tau^2 value):             0.3730
I^2 (residual heterogeneity / unaccounted variability): 22.57%
H^2 (unaccounted variability / sampling variability):   1.29
R^2 (amount of heterogeneity accounted for):            91.49%

Test for Residual Heterogeneity:
QE(df = 270) = 360.8767, p-val = 0.0002

Test of Moderators (coefficient(s)
2,3,4,5,6,7,8,9,10,11,12,13,14,15,16,17,18,19,20,21,22,23,24,25):
QM(df = 24) = 576.4932, p-val < .0001

Model Results:

                                estimate      se     zval    pval      ci.lb    ci.ub
intrcpt                         -52.8881 24.7395  -2.1378  0.0325  -101.3767  -4.3996    *
factor(Anticonvulsants)1          0.5338  0.2861   1.8653  0.0621    -0.0271   1.0946    .
factor(Antidepressants)1          0.6158  0.3296   1.8682  0.0617    -0.0302   1.2617    .
factor(Antipsychotics)1           0.0121  0.3456   0.0349  0.9721    -0.6652   0.6893
factor(ContingencyManagement)1    0.6991  0.1429   4.8908  <.0001     0.4189   0.9792  ***
factor(DopamineAgonists)1         0.4971  0.2690   1.8481  0.0646    -0.0301   1.0242    .
factor(MiscMeds)1                 0.4015  0.2476   1.6218  0.1048    -0.0837   0.8867
factor(Opioid)1                   0.3481  0.1438   2.4206  0.0155     0.0662   0.6300    *
factor(Other)1                    0.6760  0.2441   2.7687  0.0056     0.1975   1.1545   **
```

© 2021 Bentzley BS et al. *JAMA Network Open*.

```
factor(Placebo)1                  0.2060   0.2131    0.9669  0.3336    -0.2116   0.6236
factor(Psychotherapy)1           -0.1181   0.1482   -0.7966  0.4257    -0.4086   0.1725
factor(Psychostimulants)1         0.6275   0.2710    2.3154  0.0206     0.0963   1.1587      *
factor(Double_Blind)1            -0.2305   0.1947   -1.1840  0.2364    -0.6121   0.1511
factor(Multisite)1               -0.0085   0.1827   -0.0466  0.9628    -0.3665   0.3495
factor(Randomized)1              -0.5110   0.3358   -1.5218  0.1281    -1.1691   0.1471
factor(BaselineType)2            -2.2693   0.1560  -14.5456  <.0001    -2.5751  -1.9635    ***
factor(BaselineType)3            -2.5035   0.1940  -12.9023  <.0001    -2.8838  -2.1232    ***
factor(CokeFreeOutcome_Type)2    -0.1909   0.1393   -1.3706  0.1705    -0.4638   0.0821
factor(CokeFreeOutcome_Type)3    -0.7695   0.2614   -2.9437  0.0032    -1.2818  -0.2572     **
factor(CokeFreeOutcome_Type)4    -0.2304   0.1860   -1.2392  0.2153    -0.5949   0.1340
FU                                0.0247   0.0098    2.5221  0.0117     0.0055   0.0438      *
Year                              0.0270   0.0124    2.1686  0.0301     0.0026   0.0514      *
Proportion_Male                   0.8453   0.3701    2.2840  0.0224     0.1199   1.5706      *
Mean_Age                         -0.0284   0.0152   -1.8648  0.0622    -0.0583   0.0015      .
Complete                          2.5504   0.2836    8.9928  <.0001     1.9946   3.1063    ***

---
Signif. codes:  0 ‘***’ 0.001 ‘**’ 0.01 ‘*’ 0.05 ‘.’ 0.1 ‘ ’ 1

[1] I2 = 22.5747638275299
```

© 2021 Bentzley BS et al. *JAMA Network Open*.

***Random-effects model, multi-level, all covariates ***

```
***R script:

#Author: Brandon S Bentzley
#Project: Meta analysis of treatments for cocaine use disorders
#Date this R script was created: 2/20/2017
#Date this R script was last updated: 06/04/2018
#Description: This script uses rma.mv() from the package metafor to calculate random effects with
treatment and other moderators. It also includes multilevel modeling with random effects grouped by
study individual study within lead author. It calculates I2, the intraclass correlational
coefficient (ICC), and makes various diagnostic plots (some stuff is commented out)
#Note: Complete = Proportion of participants who completed the trial is currently commented out
with a #


# store the current directory
initial.dir<-getwd()
# change to the new directory
setwd("/Users/bsbentzley/Documents/Stanford/Malenka/2015.12.03 - DBS cocaine/Ranalysis")
# load the necessary libraries
library(gdata)
library(metafor)
# set the output file
sink("Output.out")
# set file width
options(width=120)
# load the dataset and define variable data types
mydata = read.csv("data.csv", colClasses=c(
"Author"="character", "Year"="numeric", "Title"="character", "StudyID"="factor",
"Double_Blind"="factor", "Multisite"="factor", "Randomized"="factor", "SubDsrdr"="character",
"Placebo"="factor", "Psychotherapy"="factor", "ContingencyManagement"="factor", "Other"="factor",
"Opioid"="factor", "MiscMeds"="factor", "Psychostimulants"="factor", "Anticonvulsants"="factor",
"DopamineAgonists"="factor", "Antidepressants"="factor", "Antipsychotics"="factor",
"TreatmentNmbr"= "numeric", "Trtmnt1orMore"="factor", "N_start"="numeric",
"Proportion_Male"="numeric", "Mean_Age"="numeric", "Mean_daysweek"="numeric",
"Proportion_30days"="numeric", "Years_use"="numeric", "ASI_Drug"="numeric",
"TestPosScreen"="factor", "CokeFreeBase"="numeric", "CokeFreeWk1"="numeric",
"BaselineType"="factor", "CokeFreeStart"="numeric", "Complication"="character", "FU"="numeric",
"Complete"="numeric", "CokeFreeLastWk_NotITT"="numeric", "CokeFreeMean_NotITT"="numeric",
"CokeFreeLastWk_ITT"="numeric", "CokeFreeMean_ITT"="numeric", "CokeFreeOutcome_Type"="factor",
"CokeFreeOutcome_ITT"="numeric", "CFS"="numeric", "CPS"="numeric", "CFE"="numeric",
"CPE"="numeric", "yi"="numeric", "vi"="numeric"))

#Do analysis (multilevel)
results<-rma.mv(yi, vi, random = ~ 1 | Author/StudyID,
    mods = ~ factor(Anticonvulsants) + factor(Antidepressants) + factor(Antipsychotics)
    + factor(ContingencyManagement) + factor(DopamineAgonists) + factor(MiscMeds)
    + factor(Opioid) + factor(Other) + factor(Placebo)
    + factor(Psychotherapy) + factor(Psychostimulants)
    + factor(Double_Blind) + factor(Multisite) + factor(Randomized)
    + factor(BaselineType) + factor(CokeFreeOutcome_Type)
    + FU + Year + Proportion_Male + Mean_Age, + Complete, #Complete in Output2 only
    data = mydata, method = "REML", measure = "OR")
print(results)

#Makes Profile Likelihood Plots of sigma2
par(mfrow=c(2,1))
profile(results, sigma2=1)
profile(results, sigma2=2)

#Calculuate Intraclass Correlation of the True Effects
print(paste0("ICC = ", round(results$sigma2[1] / sum(results$sigma2), 3)), quote = FALSE)

#Calculte I2 http://www.metafor-project.org/doku.php/tips:i2_multilevel_multivariate
X <- model.matrix(results)
W <- diag(1/mydata$vi)
    #Only include rows with values (Not NAs)
```

© 2021 Bentzley BS et al. *JAMA Network Open*.

```
    rowsInclude =as.numeric(row.names(X))
    W2=W[rowsInclude, rowsInclude]
P <- W2 - W2 %*% X %*% solve(t(X) %*% W2 %*% X) %*% t(X) %*% W2
Isquared<-100 * sum(results$sigma2) / (sum(results$sigma2) + (results$k-results$p)/sum(diag(P)))
print(paste0("I2 = ", Isquared), quote = FALSE)


# close the output file
sink()
# unload the libraries
detach("package:gdata")
detach("package:metafor")
# change back to the original directory
setwd(initial.dir)
```

```
***Output 1: (Without completion rate as a covariate)

Multivariate Meta-Analysis Model (k = 300; method: REML)

Variance Components:

            estim    sqrt  nlvls  fixed          factor
sigma^2.1  0.3190  0.5648     75     no          Author
sigma^2.2  0.3220  0.5675    114     no  Author/StudyID

Test for Residual Heterogeneity:
QE(df = 276) = 470.0981, p-val < .0001

Test of Moderators (coefficient(s) 2,3,4,5,6,7,8,9,10,11,12,13,14,15,16,17,18,19,20,21,22,23,24):
QM(df = 23) = 172.8707, p-val < .0001

Model Results:

                                 estimate       se     zval    pval      ci.lb    ci.ub
intrcpt                          -79.1880  41.1179  -1.9259  0.0541  -159.7775   1.4015    .
factor(Anticonvulsants)1           0.8396   0.3579   2.3456  0.0190     0.1380   1.5411    *
factor(Antidepressants)1           0.6749   0.3934   1.7156  0.0862    -0.0961   1.4459    .
factor(Antipsychotics)1            0.4645   0.4312   1.0772  0.2814    -0.3807   1.3098
factor(ContingencyManagement)1     0.7388   0.1390   5.3153  <.0001     0.4664   1.0112  ***
factor(DopamineAgonists)1          0.4400   0.3359   1.3098  0.1903    -0.2184   1.0983
factor(MiscMeds)1                  0.4963   0.3181   1.5601  0.1187    -0.1272   1.1199
factor(Opioid)1                    0.8406   0.2093   4.0171  <.0001     0.4305   1.2508  ***
factor(Other)1                     0.7850   0.3663   2.1431  0.0321     0.0671   1.5029    *
factor(Placebo)1                   0.3889   0.2743   1.4178  0.1563    -0.1487   0.9265
factor(Psychotherapy)1             0.0737   0.1942   0.3795  0.7043    -0.3069   0.4543
factor(Psychostimulants)1          0.9091   0.3422   2.6566  0.0079     0.2384   1.5799   **
factor(Double_Blind)1             -0.7168   0.3183  -2.2524  0.0243    -1.3406  -0.0931    *
factor(Multisite)1                 0.2064   0.3063   0.6738  0.5004    -0.3939   0.8067
factor(Randomized)1                0.2029   0.4590   0.4421  0.6584    -0.6967   1.1026
factor(BaselineType)2             -2.0780   0.2410  -8.6226  <.0001    -2.5503  -1.6057  ***
factor(BaselineType)3             -2.2036   0.3082  -7.1506  <.0001    -2.8076  -1.5996  ***
factor(CokeFreeOutcome_Type)2     -0.2724   0.2214  -1.2301  0.2186    -0.7064   0.1616
factor(CokeFreeOutcome_Type)3     -0.9075   0.4483  -2.0243  0.0429    -1.7862  -0.0288    *
factor(CokeFreeOutcome_Type)4     -0.3042   0.3135  -0.9704  0.3319    -0.9187   0.3102
FU                                 0.0149   0.0147   1.0189  0.3083    -0.0138   0.0436
Year                               0.0402   0.0206   1.9501  0.0512    -0.0002   0.0806    .
Proportion_Male                    0.7181   0.4607   1.5589  0.1190    -0.1848   1.6211
Mean_Age                          -0.0115   0.0215  -0.5366  0.5915    -0.0537   0.0306

---
Signif. codes:  0 '***' 0.001 '**' 0.01 '*' 0.05 '.' 0.1 ' ' 1

[1] ICC = 0.498
[1] I2 = 58.5381289694449
```

© 2021 Bentzley BS et al. *JAMA Network Open*.

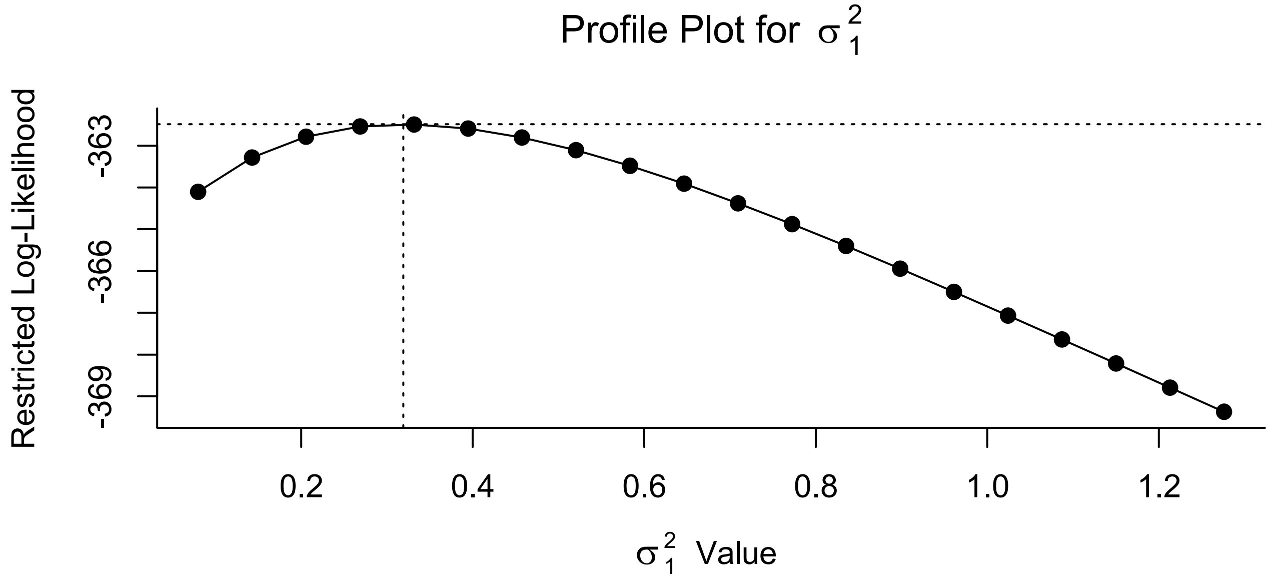

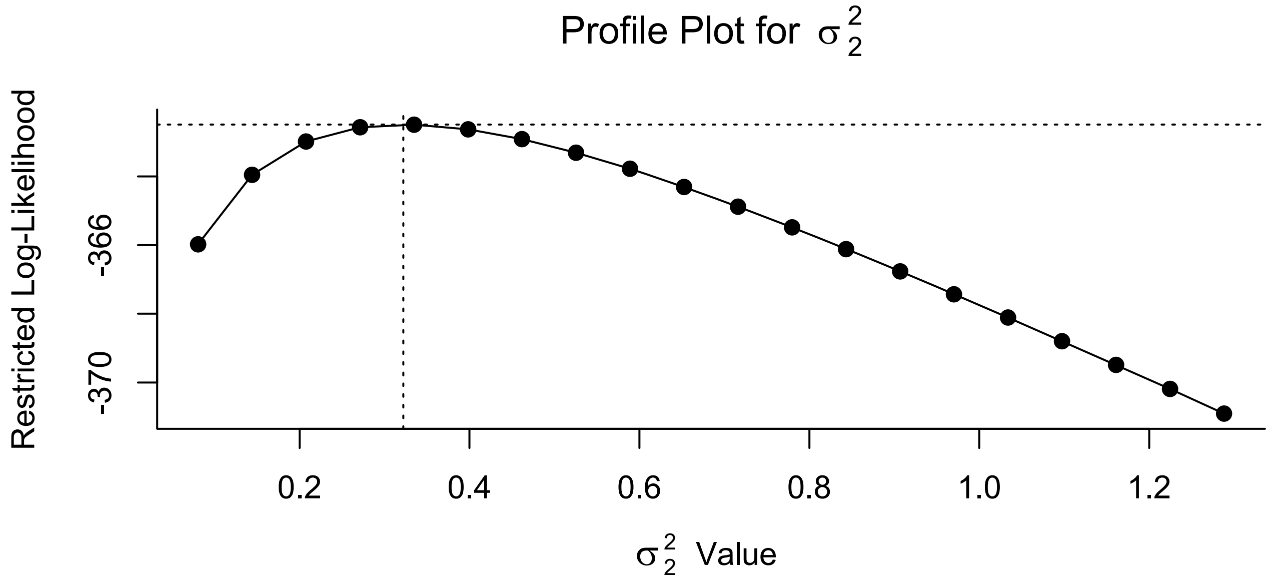

© 2021 Bentzley BS et al. *JAMA Network Open*.

```
Output 2: (With completion rate as a covariate)

Multivariate Meta-Analysis Model (k = 295; method: REML)

Variance Components:

             estim    sqrt  nlvls  fixed          factor
sigma^2.1   0.1826  0.4273     74     no          Author
sigma^2.2   0.2833  0.5322    112     no  Author/StudyID

Test for Residual Heterogeneity:
QE(df = 270) = 360.8767, p-val = 0.0002

Test of Moderators (coefficient(s)
2,3,4,5,6,7,8,9,10,11,12,13,14,15,16,17,18,19,20,21,22,23,24,25):
QM(df = 24) = 249.5480, p-val < .0001

Model Results:

                                estimate       se     zval    pval      ci.lb    ci.ub
intrcpt                         -51.6096  38.0420  -1.3566  0.1749  -126.1706  22.9514
factor(Anticonvulsants)1          0.5118   0.3494   1.4648  0.1430    -0.1730   1.1967
factor(Antidepressants)1          0.5505   0.3834   1.4357  0.1511    -0.2010   1.3020
factor(Antipsychotics)1           0.1528   0.4180   0.3656  0.7146    -0.6664   0.9721
factor(ContingencyManagement)1    0.7218   0.1523   4.7393  <.0001     0.4233   1.0203  ***
factor(DopamineAgonists)1         0.2873   0.3260   0.8811  0.3782    -0.3517   0.9263
factor(MiscMeds)1                 0.2407   0.3111   0.7738  0.4391    -0.3690   0.8505
factor(Opioid)1                   0.2378   0.2123   1.1203  0.2626    -0.1782   0.6538
factor(Other)1                    0.3651   0.3559   1.0258  0.3050    -0.3324   1.0625
factor(Placebo)1                  0.0880   0.2679   0.3284  0.7426    -0.4371   0.6131
factor(Psychotherapy)1           -0.1259   0.1878  -0.6704  0.5026    -0.4939   0.2422
factor(Psychostimulants)1         0.5854   0.3347   1.7489  0.0803    -0.0706   1.2413    .
factor(Double_Blind)1            -0.2368   0.3003  -0.7885  0.4304    -0.8254   0.3518
factor(Multisite)1                0.0713   0.2989   0.2386  0.8114    -0.5145   0.6572
factor(Randomized)1              -0.1088   0.4311  -0.2523  0.8008    -0.9537   0.7361
factor(BaselineType)2            -2.1771   0.2251  -9.6730  <.0001    -2.6183  -1.7360  ***
factor(BaselineType)3            -2.3125   0.2886  -8.0139  <.0001    -2.8780  -1.7469  ***
factor(CokeFreeOutcome_Type)2     0.0147   0.2115   0.0697  0.9445    -0.3998   0.4293
factor(CokeFreeOutcome_Type)3    -0.5449   0.4656  -1.1702  0.2419    -1.4575   0.3677
factor(CokeFreeOutcome_Type)4    -0.0839   0.2918  -0.2877  0.7736    -0.6559   0.4880
Complete                          2.7488   0.3682   7.4647  <.0001     2.0271   3.4705  ***
FU                                0.0300   0.0143   2.1053  0.0353     0.0021   0.0580    *
Year                              0.0262   0.0191   1.3721  0.1700    -0.0112   0.0636
Proportion_Male                   0.6879   0.4467   1.5399  0.1236    -0.1877   1.5635
Mean_Age                         -0.0330   0.0207  -1.5938  0.1110    -0.0735   0.0076

---
Signif. codes:  0 '***' 0.001 '**' 0.01 '*' 0.05 '.' 0.1 ' ' 1

[1] ICC = 0.392
[1] I2 = 49.3996299622723
```

© 2021 Bentzley BS et al. *JAMA Network Open*.

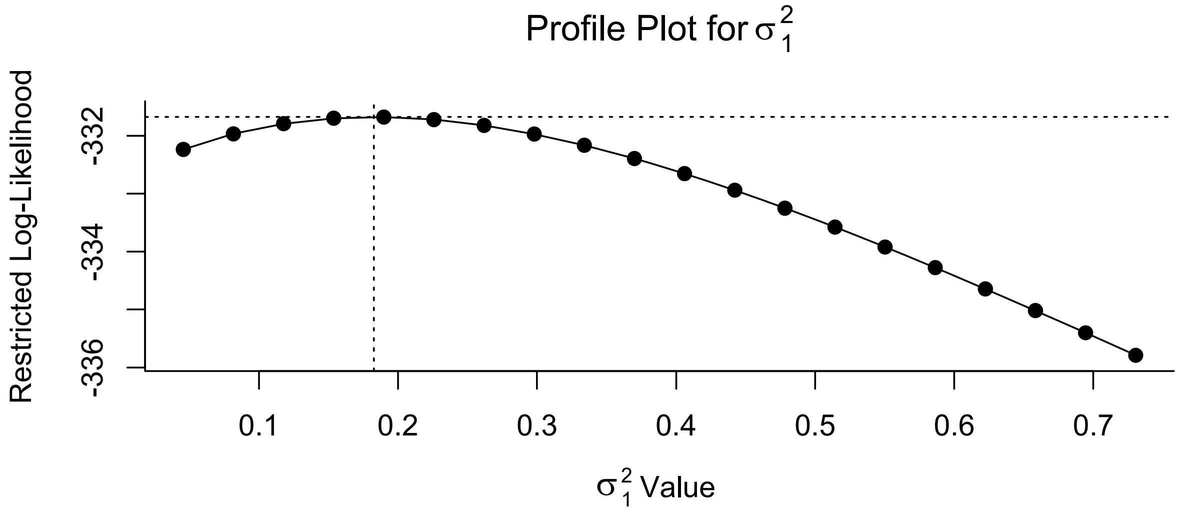

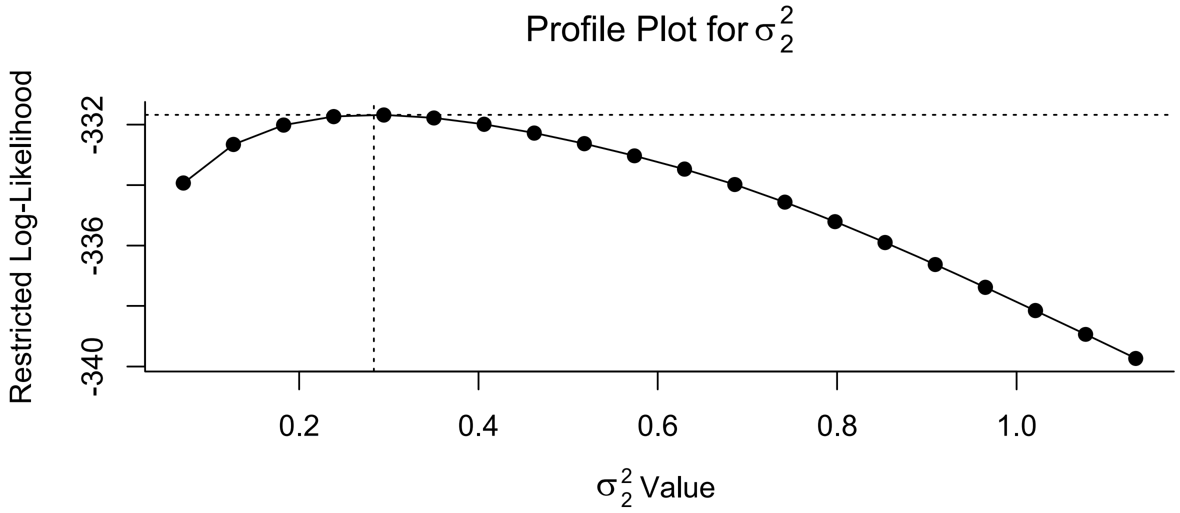

© 2021 Bentzley BS et al. *JAMA Network Open*.

*****Random-effects model, multi-level, all covariates, with imputed data *****

```
***R script:

#Author: Brandon S Bentzley
#Project: Meta analysis of treatments for cocaine use disorders
#Date this R script was created: 04/06/2018
#Date this R script was last updated: 04/07/2018
#Description: Runs MICE to impute missing data to create 5 imputed data sets as .csv files

# store the current directory
initial.dir<-getwd()
# change to the new directory
setwd("/Users/bsbentzley/Documents/Stanford/Malenka/2015.12.03 - DBS cocaine/Ranalysis")
# load the necessary libraries
library(gdata)
library(metafor)
require(mice)
require(lattice)
# set the output file
sink("Output.out")
# set file width
options(width=500)
# load the dataset and define variable data types
mydata = read.csv("DataForImp.csv", colClasses=c(
"Author"="character", "Year"="numeric", "Title"="character", "StudyID"="factor",
"Double_Blind"="factor", "Multisite"="factor", "Randomized"="factor", "SubDsrdr"="character",
"Placebo"="factor", "Psychotherapy"="factor", "ContingencyManagement"="factor", "Other"="factor",
"Opioid"="factor", "MiscMeds"="factor", "Psychostimulants"="factor", "Anticonvulsants"="factor",
"DopamineAgonists"="factor", "Antidepressants"="factor", "Antipsychotics"="factor",
"TreatmentNmbr"= "numeric", "Trtmnt1orMore"="factor", "N_start"="numeric",
"Proportion_Male"="numeric", "Mean_Age"="numeric", "Mean_daysweek"="numeric",
"Proportion_30days"="numeric", "Years_use"="numeric", "ASI_Drug"="numeric",
"TestPosScreen"="factor", "CokeFreeBase"="numeric", "CokeFreeWk1"="numeric",
"BaselineType"="factor", "CokeFreeStart"="numeric", "Complication"="character", "FU"="numeric",
"Complete"="numeric", "CokeFreeLastWk_NotITT"="numeric", "CokeFreeMean_NotITT"="numeric",
"CokeFreeLastWk_ITT"="numeric", "CokeFreeMean_ITT"="numeric", "CokeFreeOutcome_Type"="factor",
"CokeFreeOutcome_ITT"="numeric", "CFS"="numeric", "CPS"="numeric", "CFE"="numeric",
"CPE"="numeric"))

#Use this to output data types for the variables for testing
#print(str(mydata))

#MICE perform multiple imputation on Data set, 5 times, seed makes it reproducible
set.seed(123)
imp <- mice(mydata, m = 5, maxit = 5, seed = 123)

#Show imputation methods used
write.csv(imp$meth, file = "methods.csv")

#Write the imputed data sets to a long CSV file
write.csv(complete(imp, 'long'), file = "Data_imputed_long.csv")

# close the output file
sink()
# unload the libraries
detach("package:gdata")
detach("package:metafor")
# change back to the original directory
setwd(initial.dir)



#Author: Brandon S Bentzley
#Project: Meta-analysis of treatments for cocaine use disorders
#Date this R script was created: 04/06/2018
#Date this R script was last updated: 04/07/2018
#Description: Runs Escalc on 5 imputed data sets
```

© 2021 Bentzley BS et al. *JAMA Network Open*.

```
# store the current directory
initial.dir<-getwd()
# change to the new directory
setwd("/Users/bsbentzley/Documents/Stanford/Malenka/2015.12.03 - DBS cocaine/Ranalysis")
# load the necessary libraries
library(gdata)
library(metafor)
require(mice)
require(lattice)
# set the output file
sink("Output.out")
# set file width
options(width=500)


#Run Escalc for 5 files
mydata = read.csv("Data_Imputed_1.csv")
mydata <- escalc(measure = "OR", ai = CFE, bi = CPE, ci = CFS, di = CPS, data = mydata, add = 1, to
= "only0", append = TRUE)
write.csv(mydata, file = "Data_Imputed_1_EC.csv")

mydata = read.csv("Data_Imputed_2.csv")
mydata <- escalc(measure = "OR", ai = CFE, bi = CPE, ci = CFS, di = CPS, data = mydata, add = 1, to
= "only0", append = TRUE)
write.csv(mydata, file = "Data_Imputed_2_EC.csv")

mydata = read.csv("Data_Imputed_3.csv")
mydata <- escalc(measure = "OR", ai = CFE, bi = CPE, ci = CFS, di = CPS, data = mydata, add = 1, to
= "only0", append = TRUE)
write.csv(mydata, file = "Data_Imputed_3_EC.csv")

mydata = read.csv("Data_Imputed_4.csv")
mydata <- escalc(measure = "OR", ai = CFE, bi = CPE, ci = CFS, di = CPS, data = mydata, add = 1, to
= "only0", append = TRUE)
write.csv(mydata, file = "Data_Imputed_4_EC.csv")

mydata = read.csv("Data_Imputed_5.csv")
mydata <- escalc(measure = "OR", ai = CFE, bi = CPE, ci = CFS, di = CPS, data = mydata, add = 1, to
= "only0", append = TRUE)
write.csv(mydata, file = "Data_Imputed_5_EC.csv")

# close the output file
sink()
# unload the libraries
detach("package:gdata")
detach("package:metafor")
# change back to the original directory
setwd(initial.dir)



#Author: Brandon S Bentzley
#Project: Meta-analysis of treatments for cocaine use disorders
#Date this R script was created: 04/06/2018
#Date this R script was last updated: 08/23/2018
#Description: Runs multilevel analysis using 5 imputed data sets, calculates I2 for each data set,
runs collinearity diagnostics on each imputed data set, reports pooled results, makes funnel plots

# store the current directory
initial.dir<-getwd()
# change to the new directory
setwd("/Users/bsbentzley/Documents/Stanford/Malenka/2015.12.03 - DBS cocaine/Ranalysis")
# load the necessary libraries
library(gdata)
library(metafor)
require(mice)
require(lattice)
library(mctest)
```

© 2021 Bentzley BS et al. *JAMA Network Open*.

```
# set the output file
sink("Output.out")
# set file width
options(width=130)

#Build data frame from long combined CSV file of all imputations
mydata = read.csv("Data_imputed_long.csv", colClasses=c(
"Author"="character", "Year"="numeric", "Title"="character", "StudyID"="factor",
"Double_Blind"="factor", "Multisite"="factor", "Randomized"="factor", "SubDsrdr"="character",
"Placebo"="factor", "Psychotherapy"="factor", "ContingencyManagement"="factor", "Other"="factor",
"Opioid"="factor", "MiscMeds"="factor", "Psychostimulants"="factor", "Anticonvulsants"="factor",
"DopamineAgonists"="factor", "Antidepressants"="factor", "Antipsychotics"="factor",
"TreatmentNmbr"= "numeric", "Trtmnt1orMore"="factor", "N_start"="numeric",
"Proportion_Male"="numeric", "Mean_Age"="numeric", "Mean_daysweek"="numeric",
"Years_use"="numeric", "ASI_Drug"="numeric", "BaselineType"="factor", "CokeFreeStart"="numeric",
"FU"="numeric", "Complete"="numeric", "CokeFreeOutcome_ITT"="numeric", "CFS"="numeric",
"CPS"="numeric", "CFE"="numeric", "CPE"="numeric", "yi"="numeric", "vi"="numeric"))

#Turn mydata into mids object
imp <- as.mids(mydata)

#Perform analysis on mids object and return as mira
fit <- with(imp, rma.mv(yi, vi, random = ~ 1 | Author/StudyID,
    mods = ~ factor(Anticonvulsants) + factor(Antidepressants) + factor(Antipsychotics)
    + factor(ContingencyManagement) + factor(DopamineAgonists) + factor(MiscMeds) + factor(Opioid)
    + factor(Other) + factor(Placebo) + factor(Psychotherapy) + factor(Psychostimulants)
    + factor(Double_Blind) + factor(Multisite) + factor(Randomized)
    + factor(BaselineType) + factor(CokeFreeOutcome_Type)
    + FU + Year + Proportion_Male + Mean_Age + Mean_daysweek + Years_use + ASI_Drug + Complete,
    method = "REML", measure = "OR"))


#Print all model results with calculated I2 and collinearity diagnostics

#Loop through all models
for (model_num in c(1:5)){

#Print results for model #
print(paste("Model",model_num,sep=""), quote = FALSE)
results <- getfit(fit, model_num)
print(results)

#Calc I2 for model #
dataframe <- complete(imp, model_num)
X <- model.matrix(results)
W <- diag(1/dataframe$vi)
    #Only include rows with values (Not NAs)
    rowsInclude =as.numeric(row.names(X))
    W2=W[rowsInclude, rowsInclude]
P <- W2 - W2 %*% X %*% solve(t(X) %*% W2 %*% X) %*% t(X) %*% W2
Isquared<-100 * sum(results$sigma2) / (sum(results$sigma2) + (results$k-results$p)/sum(diag(P)))
print(paste("Model", model_num, "I2 = ", Isquared, sep=""), quote = FALSE)
cat("\n")#new line

#Calc collinear for model #
X <- X[,-c(1)] #for deleting columns; this takes out the intrcp=1 for all groups
Y <- dataframe$yi
coldiag <- mctest(X, Y, type="i") #perform colinearity diagnostics
print(paste("Collinearity diagnostics for Model ", model_num, sep=""), quote = FALSE)
print(coldiag)
cat("\n") #new line

#Make PDF file for graphs
pdf(file=paste("Model_",model_num,"_Funnel_Imputed.pdf",sep=""), width=93.5/25.4, height=93.5/25.4)

#Font size
par(ps=8)

#Make funnel plot
```

© 2021 Bentzley BS et al. *JAMA Network Open*.

```
funnel(results, level=c(90, 95, 99), digits=c(2,2), shade=c("white", "gray", "darkgray"),
atransf=exp, at=log(c(0.01, 0.1, 1, 10, 100)))

dev.off() #turn off PDF

 } #End For Loop


#Pool the results and print them
print(paste0("Pooled Results"), quote = FALSE)
pool.fit <- pool(fit)
print(summary(pool.fit))


# close the output file
sink()
# unload the libraries
detach("package:gdata")
detach("package:metafor")
detach("package:mice")
detach("package:lattice")
detach("package:mctest")
# change back to the original directory
setwd(initial.dir)
```

```
Output 1: (Without completion rate as covariate)

[1] Model1

Multivariate Meta-Analysis Model (k = 402; method: REML)

Variance Components:

            estim    sqrt  nlvls  fixed           factor
sigma^2.1  0.7942  0.8912     98     no           Author
sigma^2.2  0.9012  0.9493    157     no  Author/StudyID

Test for Residual Heterogeneity:
QE(df = 375) = 1150.6193, p-val < .0001

Test of Moderators (coefficient(s)
2,3,4,5,6,7,8,9,10,11,12,13,14,15,16,17,18,19,20,21,22,23,24,25,26,27):
QM(df = 26) = 109.2886, p-val < .0001

Model Results:

                                estimate       se     zval    pval      ci.lb    ci.ub
intrcpt                         -64.1216  45.9724  -1.3948  0.1631  -154.2258  25.9826
factor(Anticonvulsants)1          0.6381   0.3589   1.7777  0.0755    -0.0654   1.3416    .
factor(Antidepressants)1          0.3295   0.3952   0.8337  0.4045    -0.4451   1.1040
factor(Antipsychotics)1          -0.0160   0.4451  -0.0359  0.9713    -0.8884   0.8565
factor(ContingencyManagement)1    0.7748   0.1303   5.9450  <.0001     0.5193   1.0302  ***
factor(DopamineAgonists)1         0.2552   0.3513   0.7265  0.4675    -0.4333   0.9438
factor(MiscMeds)1                 0.3300   0.3142   1.0506  0.2934    -0.2857   0.9458
factor(Opioid)1                   0.5405   0.2637   2.0498  0.0404     0.0237   1.0573    *
factor(Other)1                    0.2946   0.3427   0.8596  0.3900    -0.3771   0.9663
factor(Placebo)1                  0.1647   0.2822   0.5838  0.5593    -0.3883   0.7178
factor(Psychotherapy)1            0.1505   0.2028   0.7418  0.4582    -0.2471   0.5480
factor(Psychostimulants)1         0.8638   0.3393   2.5457  0.0109     0.1987   1.5288    *
factor(Double_Blind)1            -0.7054   0.3626  -1.9455  0.0517    -1.4161   0.0052    .
factor(Multisite)1                0.3496   0.3797   0.9206  0.3573    -0.3947   1.0939
factor(Randomized)1               0.2495   0.4388   0.5685  0.5697    -0.6106   1.1096
factor(BaselineType)2            -1.1135   0.2646  -4.2076  <.0001    -1.6322  -0.5948  ***
factor(BaselineType)3             0.1027   0.2551   0.4026  0.6872    -0.3972   0.6026
factor(CokeFreeOutcome_Type)2     0.1633   0.3100   0.5268  0.5984    -0.4442   0.7708
factor(CokeFreeOutcome_Type)3    -0.0682   0.5105  -0.1335  0.8938    -1.0686   0.9323
factor(CokeFreeOutcome_Type)4     0.4616   0.3397   1.3587  0.1742    -0.2042   1.1274
```

© 2021 Bentzley BS et al. *JAMA Network Open*.

```
FU                                  0.0295   0.0129   2.2935  0.0218     0.0043   0.0548     *
Year                                0.0320   0.0231   1.3854  0.1659    -0.0133   0.0772
Proportion_Male                     1.0443   0.4539   2.3009  0.0214     0.1547   1.9338     *
Mean_Age                           -0.0049   0.0232  -0.2125  0.8317    -0.0503   0.0405
Mean_daysweek                       0.0438   0.0650   0.6734  0.5007    -0.0836   0.1712
Years_use                          -0.0030   0.0215  -0.1391  0.8894    -0.0451   0.0391
ASI_Drug                           -0.6410   0.5890  -1.0884  0.2764    -1.7955   0.5134

---
Signif. codes:  0 '***' 0.001 '**' 0.01 '*' 0.05 '.' 0.1 ' ' 1

[1] ICC = 0.468
[1] Model1I2 = 76.7166905524325

[1] Collinearity diagnostics for Model 1

Call:
imcdiag(x = x, y = y, method = method, corr = FALSE, vif = vif,
    tol = tol, conf = conf, cvif = cvif, leamer = leamer, all = all)


All Individual Multicollinearity Diagnostics Result

                                  VIF    TOL      Wi      Fi Leamer   CVIF Klein
factor(Anticonvulsants)1       1.4714 0.6796  7.0906  7.4057 0.8244 1.8481     0
factor(Antidepressants)1       1.4544 0.6876  6.8346  7.1383 0.8292 1.8267     0
factor(Antipsychotics)1        1.3824 0.7234  5.7509  6.0065 0.8505 1.7362     0
factor(ContingencyManagement)1 1.3664 0.7319  5.5105  5.7554 0.8555 1.7161     0
factor(DopamineAgonists)1      1.5736 0.6355  8.6271  9.0105 0.7972 1.9764     0
factor(MiscMeds)1              2.1914 0.4563 17.9183 18.7145 0.6755 2.7523     1
factor(Opioid)1                1.8416 0.5430 12.6582 13.2207 0.7369 2.3130     1
factor(Other)1                 1.4842 0.6737  7.2830  7.6066 0.8208 1.8642     0
factor(Placebo)1               3.0118 0.3320 30.2570 31.6015 0.5762 3.7827     1
factor(Psychotherapy)1         1.3753 0.7271  5.6445  5.8953 0.8527 1.7273     0
factor(Psychostimulants)1      1.4975 0.6678  7.4823  7.8148 0.8172 1.8808     0
factor(Double_Blind)1          3.2377 0.3089 33.6552 35.1507 0.5558 4.0665     1
factor(Multisite)1             1.2803 0.7811  4.2158  4.4031 0.8838 1.6080     0
factor(Randomized)1            1.3837 0.7227  5.7701  6.0265 0.8501 1.7378     0
factor(BaselineType)2          1.9469 0.5136 14.2417 14.8745 0.7167 2.4453     1
factor(BaselineType)3          1.8524 0.5398 12.8202 13.3898 0.7347 2.3266     1
factor(CokeFreeOutcome_Type)2  2.1671 0.4614 17.5532 18.3332 0.6793 2.7218     1
factor(CokeFreeOutcome_Type)3  1.3123 0.7620  4.6969  4.9056 0.8729 1.6482     0
factor(CokeFreeOutcome_Type)4  1.9311 0.5179 14.0031 14.6253 0.7196 2.4253     1
FU                             1.2264 0.8154  3.4058  3.5571 0.9030 1.5404     0
Year                           1.5038 0.6650  7.5779  7.9146 0.8155 1.8888     0
Proportion_Male                1.4701 0.6802  7.0704  7.3846 0.8248 1.8464     0
Mean_Age                       1.4746 0.6782  7.1376  7.4547 0.8235 1.8520     0
Mean_daysweek                  1.3890 0.7199  5.8506  6.1106 0.8485 1.7445     0
Years_use                      1.4529 0.6883  6.8112  7.1139 0.8296 1.8248     0
ASI_Drug                       1.6284 0.6141  9.4508  9.8707 0.7837 2.0452     1

1 --> COLLINEARITY is detected by the test
0 --> COLLINEARITY is not detected by the test

factor(Anticonvulsants)1 , factor(Antidepressants)1 , factor(Antipsychotics)1 ,
factor(ContingencyManagement)1 , factor(DopamineAgonists)1 , factor(MiscMeds)1 , factor(Other)1 ,
factor(Placebo)1 , factor(Psychotherapy)1 , factor(Psychostimulants)1 , factor(Multisite)1 ,
factor(Randomized)1 , factor(CokeFreeOutcome_Type)2 , factor(CokeFreeOutcome_Type)3 ,
factor(CokeFreeOutcome_Type)4 , Mean_Age , Mean_daysweek , Years_use , ASI_Drug , coefficient(s)
are non-significant may be due to multicollinearity

R-square of y on all x: 0.3712

* use method argument to check which regressors may be the reason of collinearity
===================================

[1] Model2

Multivariate Meta-Analysis Model (k = 402; method: REML)
```

© 2021 Bentzley BS et al. *JAMA Network Open*.

```
Variance Components:

            estim    sqrt  nlvls  fixed          factor
sigma^2.1  0.7885  0.8880     98     no          Author
sigma^2.2  0.5092  0.7136    157     no  Author/StudyID

Test for Residual Heterogeneity:
QE(df = 375) = 1056.8754, p-val < .0001

Test of Moderators (coefficient(s)
2,3,4,5,6,7,8,9,10,11,12,13,14,15,16,17,18,19,20,21,22,23,24,25,26,27):
QM(df = 26) = 224.3326, p-val < .0001

Model Results:

                                estimate       se     zval    pval      ci.lb    ci.ub
intrcpt                         -51.5233  40.1224  -1.2842  0.1991  -130.1618  27.1151
factor(Anticonvulsants)1          0.0356   0.3259   0.1093  0.9130    -0.6031   0.6743
factor(Antidepressants)1          0.3043   0.3645   0.8348  0.4038    -0.4102   1.0188
factor(Antipsychotics)1           0.2369   0.3934   0.6022  0.5470    -0.5341   1.0078
factor(ContingencyManagement)1    0.6994   0.1166   6.0000  <.0001     0.4709   0.9278  ***
factor(DopamineAgonists)1        -0.0170   0.3239  -0.0524  0.9582    -0.6519   0.6179
factor(MiscMeds)1                 0.0464   0.2842   0.1633  0.8703    -0.5107   0.6035
factor(Opioid)1                   0.4609   0.2371   1.9445  0.0518    -0.0037   0.9256    .
factor(Other)1                    0.3778   0.3004   1.2574  0.2086    -0.2111   0.9666
factor(Placebo)1                  0.0514   0.2575   0.1994  0.8419    -0.4533   0.5560
factor(Psychotherapy)1            0.2310   0.1748   1.3215  0.1863    -0.1116   0.5735
factor(Psychostimulants)1         0.4601   0.3114   1.4776  0.1395    -0.1502   1.0705
factor(Double_Blind)1            -0.0499   0.3043  -0.1641  0.8696    -0.6463   0.5464
factor(Multisite)1                0.3000   0.3285   0.9133  0.3611    -0.3439   0.9439
factor(Randomized)1               0.4648   0.3790   1.2262  0.2201    -0.2781   1.2077
factor(BaselineType)2            -1.5385   0.2137  -7.2007  <.0001    -1.9573  -1.1197  ***
factor(BaselineType)3            -1.9259   0.2050  -9.3926  <.0001    -2.3278  -1.5240  ***
factor(CokeFreeOutcome_Type)2    -0.5466   0.2509  -2.1788  0.0293    -1.0382  -0.0549    *
factor(CokeFreeOutcome_Type)3    -1.6466   0.4406  -3.7371  0.0002    -2.5102  -0.7830  ***
factor(CokeFreeOutcome_Type)4    -1.3996   0.3117  -4.4902  <.0001    -2.0105  -0.7887  ***
FU                                0.0109   0.0104   1.0455  0.2958    -0.0095   0.0312
Year                              0.0259   0.0202   1.2809  0.2002    -0.0137   0.0654
Proportion_Male                   0.6477   0.4061   1.5949  0.1107    -0.1482   1.4436
Mean_Age                          0.0052   0.0214   0.2434  0.8077    -0.0367   0.0472
Mean_daysweek                     0.0169   0.0621   0.2723  0.7854    -0.1048   0.1386
Years_use                         0.0075   0.0194   0.3863  0.6993    -0.0306   0.0456
ASI_Drug                         -0.0953   0.5300  -0.1798  0.8573    -1.1340   0.9435

---
Signif. codes:  0 '***' 0.001 '**' 0.01 '*' 0.05 '.' 0.1 ' ' 1

[1] ICC = 0.608
[1] Model2I2 = 75.2205556421797

[1] Collinearity diagnostics for Model 2

Call:
imcdiag(x = x, y = y, method = method, corr = FALSE, vif = vif,
    tol = tol, conf = conf, cvif = cvif, leamer = leamer, all = all)


All Individual Multicollinearity Diagnostics Result

                                  VIF    TOL      Wi      Fi Leamer   CVIF Klein
factor(Anticonvulsants)1       1.4791 0.6761  7.2053  7.5255 0.8223 1.3867     0
factor(Antidepressants)1       1.4268 0.7009  6.4184  6.7036 0.8372 1.3376     0
factor(Antipsychotics)1        1.3826 0.7233  5.7536  6.0093 0.8505 1.2962     0
factor(ContingencyManagement)1 1.3546 0.7382  5.3337  5.5708 0.8592 1.2700     0
factor(DopamineAgonists)1      1.5980 0.6258  8.9941  9.3937 0.7911 1.4982     0
factor(MiscMeds)1              2.1834 0.4580 17.7980 18.5889 0.6768 2.0470     1
factor(Opioid)1                2.0332 0.4918 15.5389 16.2294 0.7013 1.9061     1
factor(Other)1                 1.4681 0.6812  7.0400  7.3529 0.8253 1.3764     0
```

© 2021 Bentzley BS et al. *JAMA Network Open*.

```
factor(Placebo)1               3.0028 0.3330 30.1219 31.4604 0.5771 2.8152     1
factor(Psychotherapy)1         1.3470 0.7424  5.2184  5.4503 0.8616 1.2628     0
factor(Psychostimulants)1      1.4812 0.6751  7.2375  7.5592 0.8217 1.3887     0
factor(Double_Blind)1          2.9449 0.3396 29.2507 30.5506 0.5827 2.7609     1
factor(Multisite)1             1.3926 0.7181  5.9050  6.1674 0.8474 1.3056     0
factor(Randomized)1            1.4196 0.7044  6.3105  6.5909 0.8393 1.3309     0
factor(BaselineType)2          1.9273 0.5189 13.9465 14.5663 0.7203 1.8069     1
factor(BaselineType)3          1.6994 0.5884 10.5191 10.9866 0.7671 1.5932     0
factor(CokeFreeOutcome_Type)2  2.0547 0.4867 15.8621 16.5669 0.6976 1.9263     1
factor(CokeFreeOutcome_Type)3  1.5000 0.6667  7.5193  7.8534 0.8165 1.4062     0
factor(CokeFreeOutcome_Type)4  2.4749 0.4041 22.1820 23.1677 0.6357 2.3202     1
FU                             1.2613 0.7929  3.9293  4.1039 0.8904 1.1825     0
Year                           1.5466 0.6466  8.2210  8.5863 0.8041 1.4500     0
Proportion_Male                1.5213 0.6573  7.8403  8.1887 0.8108 1.4263     0
Mean_Age                       1.6431 0.6086  9.6725 10.1023 0.7801 1.5405     0
Mean_daysweek                  1.4602 0.6848  6.9218  7.2294 0.8275 1.3690     0
Years_use                      1.8347 0.5450 12.5538 13.1117 0.7383 1.7201     0
ASI_Drug                       1.8421 0.5428 12.6658 13.2286 0.7368 1.7271     0

1 --> COLLINEARITY is detected by the test
0 --> COLLINEARITY is not detected by the test

factor(Anticonvulsants)1 , factor(Antidepressants)1 , factor(Antipsychotics)1 ,
factor(DopamineAgonists)1 , factor(MiscMeds)1 , factor(Placebo)1 , factor(Psychotherapy)1 ,
factor(Psychostimulants)1 , factor(Double_Blind)1 , factor(Multisite)1 , factor(Randomized)1 , FU ,
Mean_Age , Mean_daysweek , Years_use , ASI_Drug , coefficient(s) are non-significant may be due to
multicollinearity

R-square of y on all x: 0.4764

* use method argument to check which regressors may be the reason of collinearity
===================================

[1] Model3

Multivariate Meta-Analysis Model (k = 402; method: REML)

Variance Components:

             estim    sqrt  nlvls  fixed          factor
sigma^2.1   0.7224  0.8499     98     no          Author
sigma^2.2   0.5227  0.7230    157     no  Author/StudyID

Test for Residual Heterogeneity:
QE(df = 375) = 1033.4830, p-val < .0001

Test of Moderators (coefficient(s)
2,3,4,5,6,7,8,9,10,11,12,13,14,15,16,17,18,19,20,21,22,23,24,25,26,27):
QM(df = 26) = 182.9648, p-val < .0001

Model Results:

                                estimate       se     zval    pval     ci.lb    ci.ub
intrcpt                         -16.5344  39.5260  -0.4183  0.6757  -94.0039  60.9351
factor(Anticonvulsants)1          0.3824   0.3239   1.1807  0.2377   -0.2524   1.0173
factor(Antidepressants)1          0.1941   0.3515   0.5520  0.5809   -0.4950   0.8831
factor(Antipsychotics)1           0.0704   0.3922   0.1796  0.8575   -0.6983   0.8392
factor(ContingencyManagement)1    0.8235   0.1148   7.1739  <.0001    0.5985   1.0485  ***
factor(DopamineAgonists)1         0.0415   0.3188   0.1301  0.8965   -0.5834   0.6663
factor(MiscMeds)1                 0.1680   0.2794   0.6012  0.5477   -0.3797   0.7156
factor(Opioid)1                   0.6187   0.2219   2.7884  0.0053    0.1838   1.0536   **
factor(Other)1                    0.2808   0.2983   0.9414  0.3465   -0.3038   0.8655
factor(Placebo)1                  0.0392   0.2513   0.1561  0.8759   -0.4533   0.5318
factor(Psychotherapy)1            0.1277   0.1748   0.7307  0.4649   -0.2148   0.4703
factor(Psychostimulants)1         0.3980   0.3063   1.2994  0.1938   -0.2024   0.9984
factor(Double_Blind)1            -0.3986   0.2988  -1.3337  0.1823   -0.9843   0.1871
factor(Multisite)1                0.3555   0.3187   1.1155  0.2646   -0.2691   0.9800
factor(Randomized)1               0.3244   0.3748   0.8656  0.3867   -0.4102   1.0590
factor(BaselineType)2            -1.1621   0.2206  -5.2675  <.0001   -1.5945  -0.7297  ***
```

© 2021 Bentzley BS et al. *JAMA Network Open*.

```
factor(BaselineType)3            -1.2667   0.2037  -6.2199  <.0001   -1.6659  -0.8676  ***
factor(CokeFreeOutcome_Type)2    -0.5263   0.2466  -2.1338  0.0329   -1.0096  -0.0429    *
factor(CokeFreeOutcome_Type)3    -1.6218   0.4636  -3.4983  0.0005   -2.5304  -0.7132  ***
factor(CokeFreeOutcome_Type)4    -1.5978   0.2969  -5.3810  <.0001   -2.1798  -1.0158  ***
FU                                0.0173   0.0102   1.6969  0.0897   -0.0027   0.0374    .
Year                              0.0076   0.0199   0.3839  0.7011   -0.0313   0.0466
Proportion_Male                   1.0586   0.4049   2.6145  0.0089    0.2650   1.8521   **
Mean_Age                          0.0277   0.0212   1.3080  0.1909   -0.0138   0.0692
Mean_daysweek                     0.1330   0.0617   2.1574  0.0310    0.0122   0.2539    *
Years_use                         0.0154   0.0200   0.7705  0.4410   -0.0238   0.0546
ASI_Drug                         -0.5393   0.4576  -1.1784  0.2386   -1.4362   0.3577

---
Signif. codes:  0 '***' 0.001 '**' 0.01 '*' 0.05 '.' 0.1 ' ' 1

[1] ICC = 0.58
[1] Model3I2 = 74.9574674457457

[1] Collinearity diagnostics for Model 3

Call:
imcdiag(x = x, y = y, method = method, corr = FALSE, vif = vif,
    tol = tol, conf = conf, cvif = cvif, leamer = leamer, all = all)


All Individual Multicollinearity Diagnostics Result

                                  VIF    TOL      Wi      Fi Leamer   CVIF Klein
factor(Anticonvulsants)1       1.4736 0.6786  7.1228  7.4393 0.8238 1.7449     0
factor(Antidepressants)1       1.4418 0.6936  6.6448  6.9400 0.8328 1.7072     0
factor(Antipsychotics)1        1.4228 0.7029  6.3584  6.6410 0.8384 1.6847     0
factor(ContingencyManagement)1 1.3709 0.7294  5.5791  5.8270 0.8541 1.6233     0
factor(DopamineAgonists)1      1.5848 0.6310  8.7959  9.1867 0.7943 1.8766     0
factor(MiscMeds)1              2.1846 0.4577 17.8170 18.6088 0.6766 2.5868     1
factor(Opioid)1                1.6472 0.6071  9.7344 10.1669 0.7792 1.9505     0
factor(Other)1                 1.4481 0.6905  6.7400  7.0395 0.8310 1.7147     0
factor(Placebo)1               3.0292 0.3301 30.5198 31.8760 0.5746 3.5869     1
factor(Psychotherapy)1         1.3860 0.7215  5.8050  6.0630 0.8494 1.6411     0
factor(Psychostimulants)1      1.4983 0.6674  7.4950  7.8281 0.8169 1.7742     0
factor(Double_Blind)1          3.1619 0.3163 32.5152 33.9601 0.5624 3.7440     1
factor(Multisite)1             1.3666 0.7318  5.5130  5.7580 0.8554 1.6181     0
factor(Randomized)1            1.4004 0.7141  6.0217  6.2893 0.8450 1.6582     0
factor(BaselineType)2          1.9988 0.5003 15.0220 15.6895 0.7073 2.3668     0
factor(BaselineType)3          2.0102 0.4975 15.1935 15.8687 0.7053 2.3803     0
factor(CokeFreeOutcome_Type)2  1.9675 0.5083 14.5517 15.1983 0.7129 2.3297     0
factor(CokeFreeOutcome_Type)3  1.5997 0.6251  9.0195  9.4203 0.7906 1.8942     0
factor(CokeFreeOutcome_Type)4  2.2493 0.4446 18.7897 19.6247 0.6668 2.6634     1
FU                             1.2327 0.8112  3.4993  3.6548 0.9007 1.4596     0
Year                           1.5482 0.6459  8.2456  8.6121 0.8037 1.8333     0
Proportion_Male                1.4187 0.7049  6.2977  6.5775 0.8396 1.6799     0
Mean_Age                       1.5754 0.6348  8.6536  9.0381 0.7967 1.8654     0
Mean_daysweek                  1.5850 0.6309  8.7987  9.1897 0.7943 1.8768     0
Years_use                      1.9582 0.5107 14.4110 15.0514 0.7146 2.3187     0
ASI_Drug                       1.4401 0.6944  6.6186  6.9127 0.8333 1.7052     0

1 --> COLLINEARITY is detected by the test
0 --> COLLINEARITY is not detected by the test

factor(Anticonvulsants)1 , factor(Antidepressants)1 , factor(Antipsychotics)1 ,
factor(DopamineAgonists)1 , factor(MiscMeds)1 , factor(Other)1 , factor(Placebo)1 ,
factor(Psychotherapy)1 , factor(Psychostimulants)1 , factor(Double_Blind)1 , factor(Multisite)1 ,
factor(Randomized)1 , Year , Mean_Age , Mean_daysweek , ASI_Drug , coefficient(s) are non-
significant may be due to multicollinearity

R-square of y on all x: 0.5165

* use method argument to check which regressors may be the reason of collinearity
===================================
```

© 2021 Bentzley BS et al. *JAMA Network Open*.

```
[1] Model4

Multivariate Meta-Analysis Model (k = 402; method: REML)

Variance Components:

            estim    sqrt  nlvls  fixed           factor
sigma^2.1  0.7882  0.8878     98     no           Author
sigma^2.2  0.6228  0.7892    157     no  Author/StudyID

Test for Residual Heterogeneity:
QE(df = 375) = 1029.4430, p-val < .0001

Test of Moderators (coefficient(s)
2,3,4,5,6,7,8,9,10,11,12,13,14,15,16,17,18,19,20,21,22,23,24,25,26,27):
QM(df = 26) = 124.7149, p-val < .0001

Model Results:

                                estimate       se     zval    pval      ci.lb    ci.ub
intrcpt                         -47.7313  43.8140  -1.0894  0.2760  -133.6051  38.1426
factor(Anticonvulsants)1          0.2162   0.3529   0.6127  0.5401    -0.4755   0.9080
factor(Antidepressants)1          0.2804   0.3838   0.7306  0.4650    -0.4718   1.0325
factor(Antipsychotics)1           0.0800   0.4139   0.1932  0.8468    -0.7312   0.8911
factor(ContingencyManagement)1    0.7042   0.1315   5.3571  <.0001     0.4466   0.9619  ***
factor(DopamineAgonists)1         0.0209   0.3354   0.0623  0.9503    -0.6365   0.6783
factor(MiscMeds)1                 0.0309   0.3029   0.1021  0.9187    -0.5628   0.6246
factor(Opioid)1                   0.3601   0.2385   1.5094  0.1312    -0.1075   0.8276
factor(Other)1                    0.2921   0.3320   0.8796  0.3791    -0.3587   0.9428
factor(Placebo)1                 -0.0696   0.2687  -0.2591  0.7955    -0.5963   0.4570
factor(Psychotherapy)1            0.1775   0.1974   0.8993  0.3685    -0.2094   0.5645
factor(Psychostimulants)1         0.5749   0.3245   1.7717  0.0764    -0.0611   1.2108    .
factor(Double_Blind)1            -0.4629   0.3257  -1.4211  0.1553    -1.1013   0.1755
factor(Multisite)1                0.3194   0.3622   0.8821  0.3777    -0.3904   1.0292
factor(Randomized)1              -0.1804   0.4164  -0.4333  0.6648    -0.9966   0.6358
factor(BaselineType)2            -1.6199   0.2492  -6.4996  <.0001    -2.1084  -1.1314  ***
factor(BaselineType)3            -1.0147   0.2577  -3.9369  <.0001    -1.5199  -0.5096  ***
factor(CokeFreeOutcome_Type)2     0.1029   0.2889   0.3561  0.7218    -0.4634   0.6691
factor(CokeFreeOutcome_Type)3    -0.2487   0.4632  -0.5369  0.5914    -1.1564   0.6591
factor(CokeFreeOutcome_Type)4     0.7213   0.3210   2.2472  0.0246     0.0922   1.3504    *
FU                                0.0284   0.0121   2.3568  0.0184     0.0048   0.0520    *
Year                              0.0245   0.0220   1.1122  0.2661    -0.0187   0.0676
Proportion_Male                   0.7542   0.4321   1.7455  0.0809    -0.0926   1.6011    .
Mean_Age                         -0.0239   0.0218  -1.0960  0.2731    -0.0666   0.0188
Mean_daysweek                     0.1085   0.0649   1.6719  0.0945    -0.0187   0.2358    .
Years_use                         0.0100   0.0206   0.4858  0.6271    -0.0303   0.0503
ASI_Drug                          0.3502   0.8464   0.4137  0.6791    -1.3088   2.0091

---
Signif. codes:  0 '***' 0.001 '**' 0.01 '*' 0.05 '.' 0.1 ' ' 1

[1] ICC = 0.559
[1] Model4I2 = 72.938820015205

[1] Collinearity diagnostics for Model 4

Call:
imcdiag(x = x, y = y, method = method, corr = FALSE, vif = vif,
    tol = tol, conf = conf, cvif = cvif, leamer = leamer, all = all)


All Individual Multicollinearity Diagnostics Result

                                   VIF    TOL     Wi      Fi Leamer   CVIF Klein
factor(Anticonvulsants)1        1.5346 0.6516 8.0399  8.3972 0.8072 1.8364     0
factor(Antidepressants)1        1.4619 0.6840 6.9477  7.2564 0.8271 1.7495     0
factor(Antipsychotics)1         1.3989 0.7149 5.9989  6.2655 0.8455 1.6740     0
factor(ContingencyManagement)1 1.3502 0.7406 5.2672  5.5012 0.8606 1.6158     0
factor(DopamineAgonists)1       1.5876 0.6299 8.8376  9.2304 0.7936 1.8999     0
```

© 2021 Bentzley BS et al. *JAMA Network Open*.

```
factor(MiscMeds)1               2.2347 0.4475 18.5698 19.3950 0.6689 2.6743     1
factor(Opioid)1                 1.5657 0.6387  8.5089  8.8870 0.7992 1.8738     0
factor(Other)1                  1.4914 0.6705  7.3904  7.7188 0.8189 1.7848     0
factor(Placebo)1                3.1049 0.3221 31.6580 33.0647 0.5675 3.7157     1
factor(Psychotherapy)1          1.3835 0.7228  5.7679  6.0242 0.8502 1.6557     0
factor(Psychostimulants)1       1.4867 0.6726  7.3203  7.6456 0.8201 1.7792     0
factor(Double_Blind)1           2.9565 0.3382 29.4264 30.7341 0.5816 3.5381     1
factor(Multisite)1              1.4532 0.6881  6.8159  7.1187 0.8295 1.7390     0
factor(Randomized)1             1.4402 0.6943  6.6210  6.9152 0.8333 1.7235     0
factor(BaselineType)2           1.8836 0.5309 13.2895 13.8801 0.7286 2.2541     1
factor(BaselineType)3           1.7258 0.5794 10.9159 11.4009 0.7612 2.0653     1
factor(CokeFreeOutcome_Type)2   2.5299 0.3953 23.0093 24.0317 0.6287 3.0275     1
factor(CokeFreeOutcome_Type)3   1.3343 0.7495  5.0273  5.2507 0.8657 1.5967     0
factor(CokeFreeOutcome_Type)4   2.1595 0.4631 17.4389 18.2138 0.6805 2.5843     1
FU                              1.2653 0.7903  3.9909  4.1682 0.8890 1.5143     0
Year                            1.6411 0.6094  9.6418 10.0702 0.7806 1.9639     0
Proportion_Male                 1.4072 0.7106  6.1249  6.3971 0.8430 1.6841     0
Mean_Age                        1.3932 0.7178  5.9133  6.1760 0.8472 1.6672     0
Mean_daysweek                   1.2982 0.7703  4.4850  4.6843 0.8777 1.5536     0
Years_use                       1.2326 0.8113  3.4990  3.6545 0.9007 1.4751     0
ASI_Drug                        1.9410 0.5152 14.1530 14.7820 0.7178 2.3229     1

1 --> COLLINEARITY is detected by the test
0 --> COLLINEARITY is not detected by the test

factor(Anticonvulsants)1 , factor(Antidepressants)1 , factor(Antipsychotics)1 ,
factor(ContingencyManagement)1 , factor(DopamineAgonists)1 , factor(MiscMeds)1 , factor(Other)1 ,
factor(Placebo)1 , factor(Psychotherapy)1 , factor(Psychostimulants)1 , factor(Multisite)1 ,
factor(Randomized)1 , factor(CokeFreeOutcome_Type)2 , factor(CokeFreeOutcome_Type)3 ,
factor(CokeFreeOutcome_Type)4 , Mean_Age , Years_use , ASI_Drug , coefficient(s) are non-
significant may be due to multicollinearity

R-square of y on all x: 0.4127

* use method argument to check which regressors may be the reason of collinearity
===================================

[1] Model5

Multivariate Meta-Analysis Model (k = 402; method: REML)

Variance Components:

            estim    sqrt  nlvls  fixed           factor
sigma^2.1  0.2789  0.5281     98     no           Author
sigma^2.2  0.9800  0.9899    157     no  Author/StudyID

Test for Residual Heterogeneity:
QE(df = 375) = 1023.1494, p-val < .0001

Test of Moderators (coefficient(s)
2,3,4,5,6,7,8,9,10,11,12,13,14,15,16,17,18,19,20,21,22,23,24,25,26,27):
QM(df = 26) = 149.9986, p-val < .0001

Model Results:

                                estimate       se     zval    pval      ci.lb    ci.ub
intrcpt                         -39.6270  42.3165  -0.9364  0.3490  -122.5657  43.3118
factor(Anticonvulsants)1          0.3909   0.3480   1.1231  0.2614    -0.2913   1.0730
factor(Antidepressants)1          0.4241   0.3853   1.1009  0.2710    -0.3310   1.1792
factor(Antipsychotics)1          -0.2520   0.4037  -0.6242  0.5325    -1.0433   0.5393
factor(ContingencyManagement)1    0.7851   0.1250   6.2800  <.0001     0.5401   1.0301  ***
factor(DopamineAgonists)1        -0.0828   0.3344  -0.2476  0.8045    -0.7382   0.5727
factor(MiscMeds)1                 0.0809   0.2970   0.2724  0.7853    -0.5012   0.6630
factor(Opioid)1                   0.6644   0.2303   2.8849  0.0039     0.2130   1.1158   **
factor(Other)1                    0.2392   0.3060   0.7817  0.4344    -0.3606   0.8390
factor(Placebo)1                 -0.0392   0.2660  -0.1474  0.8828    -0.5605   0.4821
factor(Psychotherapy)1            0.1495   0.1838   0.8134  0.4160    -0.2107   0.5096
factor(Psychostimulants)1         0.4791   0.3224   1.4859  0.1373    -0.1529   1.1110
```

© 2021 Bentzley BS et al. *JAMA Network Open*.

```
factor(Double_Blind)1              0.0709   0.3085   0.2299  0.8182    -0.5337   0.6755
factor(Multisite)1                -0.0422   0.3538  -0.1192  0.9052    -0.7356   0.6513
factor(Randomized)1               -0.4997   0.4054  -1.2327  0.2177    -1.2943   0.2948
factor(BaselineType)2             -1.2367   0.2258  -5.4774  <.0001    -1.6792  -0.7942  ***
factor(BaselineType)3             -0.2764   0.2334  -1.1842  0.2363    -0.7340   0.1811
factor(CokeFreeOutcome_Type)2     -0.5184   0.3013  -1.7204  0.0854    -1.1090   0.0722    .
factor(CokeFreeOutcome_Type)3     -1.3126   0.4623  -2.8396  0.0045    -2.2187  -0.4066   **
factor(CokeFreeOutcome_Type)4      0.7580   0.3240   2.3393  0.0193     0.1229   1.3931    *
FU                                 0.0202   0.0118   1.7148  0.0864    -0.0029   0.0432    .
Year                               0.0197   0.0213   0.9278  0.3535    -0.0219   0.0614
Proportion_Male                    0.7853   0.4270   1.8390  0.0659    -0.0516   1.6222    .
Mean_Age                           0.0085   0.0213   0.4015  0.6880    -0.0332   0.0503
Mean_daysweek                      0.0553   0.0683   0.8092  0.4184    -0.0786   0.1892
Years_use                          0.0104   0.0199   0.5226  0.6013    -0.0286   0.0494
ASI_Drug                           0.2461   0.5257   0.4681  0.6397    -0.7843   1.2765

---
Signif. codes:  0 '***' 0.001 '**' 0.01 '*' 0.05 '.' 0.1 ' ' 1

[1] ICC = 0.222
[1] Model5I2 = 72.6886552758625

[1] Collinearity diagnostics for Model 5

Call:
imcdiag(x = x, y = y, method = method, corr = FALSE, vif = vif,
    tol = tol, conf = conf, cvif = cvif, leamer = leamer, all = all)


All Individual Multicollinearity Diagnostics Result

                                 VIF    TOL      Wi      Fi Leamer   CVIF Klein
factor(Anticonvulsants)1      1.5184 0.6586  7.7961  8.1425 0.8115 2.2490     0
factor(Antidepressants)1      1.4336 0.6976  6.5211  6.8109 0.8352 2.1234     0
factor(Antipsychotics)1       1.4263 0.7011  6.4119  6.6968 0.8373 2.1126     0
factor(ContingencyManagement)1 1.3736 0.7280  5.6196  5.8693 0.8532 2.0346     0
factor(DopamineAgonists)1     1.5800 0.6329  8.7228  9.1104 0.7956 2.3402     0
factor(MiscMeds)1             2.2206 0.4503 18.3579 19.1736 0.6711 3.2891     1
factor(Opioid)1               1.5609 0.6407  8.4359  8.8107 0.8004 2.3120     0
factor(Other)1                1.4845 0.6736  7.2873  7.6111 0.8207 2.1989     0
factor(Placebo)1              3.0791 0.3248 31.2698 32.6593 0.5699 4.5607     1
factor(Psychotherapy)1        1.4048 0.7119  6.0878  6.3583 0.8437 2.0807     0
factor(Psychostimulants)1     1.4848 0.6735  7.2920  7.6160 0.8207 2.1993     0
factor(Double_Blind)1         2.8373 0.3524 27.6327 28.8606 0.5937 4.2025     1
factor(Multisite)1            1.3446 0.7437  5.1823  5.4126 0.8624 1.9916     0
factor(Randomized)1           1.4173 0.7055  6.2769  6.5559 0.8400 2.0994     0
factor(BaselineType)2         1.9907 0.5023 14.8994 15.5615 0.7088 2.9485     1
factor(BaselineType)3         1.9884 0.5029 14.8660 15.5266 0.7092 2.9452     1
factor(CokeFreeOutcome_Type)2 2.9980 0.3336 30.0493 31.3846 0.5775 4.4405     1
factor(CokeFreeOutcome_Type)3 1.3292 0.7523  4.9513  5.1713 0.8674 1.9688     0
factor(CokeFreeOutcome_Type)4 2.0528 0.4872 15.8334 16.5370 0.6980 3.0405     1
FU                            1.2250 0.8163  3.3847  3.5351 0.9035 1.8145     0
Year                          1.6017 0.6243  9.0493  9.4515 0.7902 2.3724     0
Proportion_Male               1.4141 0.7072  6.2274  6.5041 0.8409 2.0945     0
Mean_Age                      1.4013 0.7136  6.0355  6.3037 0.8448 2.0756     0
Mean_daysweek                 1.3987 0.7150  5.9964  6.2629 0.8455 2.0717     0
Years_use                     1.7174 0.5823 10.7892 11.2687 0.7631 2.5437     0
ASI_Drug                      2.2857 0.4375 19.3375 20.1968 0.6614 3.3856     1

1 --> COLLINEARITY is detected by the test
0 --> COLLINEARITY is not detected by the test

factor(Anticonvulsants)1 , factor(Antidepressants)1 , factor(DopamineAgonists)1 , factor(MiscMeds)1
, factor(Other)1 , factor(Placebo)1 , factor(Psychotherapy)1 , factor(Psychostimulants)1 ,
factor(Double_Blind)1 , factor(Multisite)1 , factor(CokeFreeOutcome_Type)2 , Year , Mean_Age ,
Mean_daysweek , Years_use , ASI_Drug , coefficient(s) are non-significant may be due to
multicollinearity

R-square of y on all x: 0.4451
```
© 2021 Bentzley BS et al. *JAMA Network Open*.

```
* use method argument to check which regressors may be the reason of collinearity
===================================

[1] Pooled Results
                                          est          se           t         df     Pr(>|t|)
lo 95       hi 95 nmis
intrcpt                        -43.907529397 46.62440419 -0.94172848  93.765559 3.487516e-01 -
1.364844e+02 48.66933572   NA
factor(Anticonvulsants)1         0.332647014  0.42119681  0.78976623  30.372545 4.357870e-01 -
5.271094e-01  1.19240342   NA
factor(Antidepressants)1         0.306467265  0.38726472  0.79136376 277.406870 4.294079e-01 -
4.558836e-01  1.06881815   NA
factor(Antipsychotics)1          0.023853718  0.45466371  0.05246453  83.513078 9.582838e-01 -
8.803720e-01  0.92807939   NA
factor(ContingencyManagement)1   0.757399884  0.13719332  5.52067619  84.120914 3.673800e-07
4.845817e-01  1.03021811   NA
factor(DopamineAgonists)1        0.043565170  0.36104169  0.12066520 114.433769 9.041678e-01 -
6.716266e-01  0.75875690   NA
factor(MiscMeds)1                0.131248988  0.32511825  0.40369615  93.885178 6.873542e-01 -
5.142912e-01  0.77678920   NA
factor(Opioid)1                  0.528921867  0.27365336  1.93281698  56.122529 5.831050e-02 -
1.924528e-02  1.07708902   NA
factor(Other)1                   0.296889670  0.32118685  0.92435188 335.535127 3.559674e-01 -
3.349039e-01  0.92868323   NA
factor(Placebo)1                 0.029299990  0.28359370  0.10331679 143.980508 9.178553e-01 -
5.312449e-01  0.58984487   NA
factor(Psychotherapy)1           0.167235651  0.19207922  0.87065976 286.691709 3.846684e-01 -
2.108287e-01  0.54530002   NA
factor(Psychostimulants)1        0.555174471  0.37890957  1.46518989  42.255480 1.502708e-01 -
2.093591e-01  1.31970799   NA
factor(Double_Blind)1           -0.309188061  0.47224852 -0.65471473  12.771146 5.242683e-01 -
1.331281e+00  0.71290439   NA
factor(Multisite)1               0.256476576  0.39503559  0.64924929  65.212156 5.184575e-01 -
5.324153e-01  1.04536847   NA
factor(Randomized)1              0.071709436  0.59575588  0.12036715  12.654782 9.060823e-01 -
1.218921e+00  1.36233995   NA
factor(BaselineType)2           -1.334140571  0.34474420 -3.86994348  13.022345 1.927259e-03 -
2.078785e+00 -0.58949589   NA
factor(BaselineType)3           -0.876220704  0.91178991 -0.96098969   3.846426 3.929943e-01 -
3.448120e+00  1.69567847   NA
factor(CokeFreeOutcome_Type)2   -0.265012317  0.48781970 -0.54325875   8.339523 6.011674e-01 -
1.382004e+00  0.85197980   NA
factor(CokeFreeOutcome_Type)3   -0.979574713  0.95892697 -1.02153213   6.406533 3.440181e-01 -
3.290360e+00  1.33121103   NA
factor(CokeFreeOutcome_Type)4   -0.211317791  1.33442960 -0.15835814   3.715219 8.824049e-01 -
4.031024e+00  3.60838801   NA
FU                               0.021258733  0.01433818  1.48266594  27.831663 1.493991e-01 -
8.119705e-03  0.05063717    0
Year                             0.021932369  0.02353582  0.93187184  88.077402 3.539501e-01 -
2.483956e-02  0.06870430    0
Proportion_Male                  0.858014017  0.47046869  1.82374307  85.613488 7.168031e-02 -
7.730703e-02  1.79333506   11
Mean_Age                         0.002526762  0.03004749  0.08409231  16.258369 9.340098e-01 -
6.108889e-02  0.06614242   10
Mean_daysweek                    0.071504944  0.08308718  0.86060136  22.647279 3.984773e-01 -
1.005222e-01  0.24353213  128
Years_use                        0.008061793  0.02161866  0.37290907 151.840409 7.097362e-01 -
3.465041e-02  0.05077400  166
ASI_Drug                        -0.135866567  0.77879113 -0.17445829  22.854495 8.630415e-01 -
1.747487e+00  1.47575337  273
                                      fmi     lambda
intrcpt                        0.18945253 0.17234616
factor(Anticonvulsants)1       0.37935369 0.33978748
factor(Antidepressants)1       0.06211933 0.05538185
factor(Antipsychotics)1        0.20513723 0.18632682
factor(ContingencyManagement)1 0.20413435 0.18543470
factor(DopamineAgonists)1      0.16397259 0.14948762
factor(MiscMeds)1              0.18928363 0.17219526
```

© 2021 Bentzley BS et al. *JAMA Network Open*.

```
factor(Opioid)1                0.26486070 0.23912167
factor(Other)1                 0.03525675 0.02952337
factor(Placebo)1               0.13653674 0.12462530
factor(Psychotherapy)1         0.05799971 0.05145103
factor(Psychostimulants)1      0.31406283 0.28234722
factor(Double_Blind)1          0.59701515 0.53848918
factor(Multisite)1             0.24114551 0.21822360
factor(Randomized)1            0.59967295 0.54103752
factor(BaselineType)2          0.59138227 0.53310133
factor(BaselineType)3          0.95410440 0.93516443
factor(CokeFreeOutcome_Type)2  0.72719960 0.66878111
factor(CokeFreeOutcome_Type)3  0.81198787 0.76121860
factor(CokeFreeOutcome_Type)4  0.95987787 0.94285974
FU                             0.39819821 0.35645231
Year                           0.19784341 0.17983309
Proportion_Male                0.20171385 0.18328055
Mean_Age                       0.52950055 0.47497634
Mean_daysweek                  0.44539624 0.39848989
Years_use                      0.13042098 0.11904206
ASI_Drug                       0.44322847 0.39654783
```

Output 2: (With completion rate as covariate)

```
[1] Model1

Multivariate Meta-Analysis Model (k = 402; method: REML)

Variance Components:

            estim    sqrt  nlvls  fixed          factor
sigma^2.1  0.2787  0.5279     98     no          Author
sigma^2.2  1.0922  1.0451    157     no  Author/StudyID

Test for Residual Heterogeneity:
QE(df = 374) = 949.7756, p-val < .0001

Test of Moderators (coefficient(s)
2,3,4,5,6,7,8,9,10,11,12,13,14,15,16,17,18,19,20,21,22,23,24,25,26,27,28):
QM(df = 27) = 174.8822, p-val < .0001

Model Results:

                                estimate       se     zval    pval      ci.lb    ci.ub
intrcpt                         -64.4895  42.8486  -1.5051  0.1323  -148.4711  19.4922
factor(Anticonvulsants)1          0.4189   0.3502   1.1960  0.2317    -0.2676   1.1053
factor(Antidepressants)1          0.1757   0.3884   0.4525  0.6509    -0.5854   0.9369
factor(Antipsychotics)1          -0.3218   0.4379  -0.7350  0.4624    -1.1801   0.5364
factor(ContingencyManagement)1    0.6804   0.1303   5.2205  <.0001     0.4249   0.9358  ***
factor(DopamineAgonists)1         0.1018   0.3423   0.2973  0.7662    -0.5692   0.7727
factor(MiscMeds)1                 0.0375   0.3076   0.1220  0.9029    -0.5653   0.6403
factor(Opioid)1                   0.1267   0.2552   0.4965  0.6195    -0.3735   0.6270
factor(Other)1                   -0.0205   0.3382  -0.0605  0.9518    -0.6834   0.6425
factor(Placebo)1                 -0.1149   0.2744  -0.4189  0.6753    -0.6527   0.4228
factor(Psychotherapy)1           -0.0773   0.2017  -0.3830  0.7017    -0.4726   0.3181
factor(Psychostimulants)1         0.5585   0.3323   1.6805  0.0929    -0.0929   1.2098    .
factor(Double_Blind)1            -0.4477   0.3416  -1.3107  0.1900    -1.1172   0.2218
factor(Multisite)1                0.3364   0.3644   0.9231  0.3559    -0.3778   1.0505
factor(Randomized)1               0.0818   0.4154   0.1970  0.8438    -0.7324   0.8960
factor(BaselineType)2            -1.2333   0.2561  -4.8152  <.0001    -1.7353  -0.7313  ***
factor(BaselineType)3            -0.0828   0.2509  -0.3298  0.7415    -0.5746   0.4091
factor(CokeFreeOutcome_Type)2     0.5681   0.3025   1.8779  0.0604    -0.0248   1.1610    .
factor(CokeFreeOutcome_Type)3     0.4853   0.5035   0.9638  0.3351    -0.5016   1.4722
factor(CokeFreeOutcome_Type)4     0.6928   0.3318   2.0883  0.0368     0.0426   1.3431    *
FU                                0.0351   0.0124   2.8380  0.0045     0.0108   0.0593   **
Year                              0.0318   0.0215   1.4766  0.1398    -0.0104   0.0739
Proportion_Male                   1.0264   0.4433   2.3154  0.0206     0.1576   1.8952    *
Mean_Age                         -0.0173   0.0221  -0.7831  0.4336    -0.0605   0.0260
Mean_daysweek                     0.0240   0.0630   0.3803  0.7037    -0.0996   0.1476
```

© 2021 Bentzley BS et al. *JAMA Network Open*.

```
Years_use                          -0.0187   0.0209  -0.8961  0.3702    -0.0597   0.0222
ASI_Drug                           -0.4087   0.5778  -0.7072  0.4794    -1.5412   0.7239
Complete                            2.7472   0.3763   7.3001  <.0001     2.0096   3.4847  ***

---
Signif. codes:  0 '***' 0.001 '**' 0.01 '*' 0.05 '.' 0.1 ' ' 1

[1] ICC = 0.203
[1] Model1I2 = 72.6574118961228

[1] Collinearity diagnostics for Model 1

Call:
imcdiag(x = x, y = y, method = method, corr = FALSE, vif = vif,
    tol = tol, conf = conf, cvif = cvif, leamer = leamer, all = all)


All Individual Multicollinearity Diagnostics Result

                                   VIF    TOL      Wi      Fi Leamer   CVIF Klein
factor(Anticonvulsants)1        1.4833 0.6742  6.9714  7.2696 0.8211 2.3390     0
factor(Antidepressants)1        1.4544 0.6876  6.5544  6.8347 0.8292 2.2934     0
factor(Antipsychotics)1         1.3825 0.7233  5.5167  5.7526 0.8505 2.1799     0
factor(ContingencyManagement)1  1.3702 0.7298  5.3397  5.5681 0.8543 2.1606     0
factor(DopamineAgonists)1       1.5746 0.6351  8.2876  8.6421 0.7969 2.4829     0
factor(MiscMeds)1               2.2211 0.4502 17.6117 18.3650 0.6710 3.5022     1
factor(Opioid)1                 2.1712 0.4606 16.8919 17.6144 0.6787 3.4235     1
factor(Other)1                  1.5040 0.6649  7.2694  7.5804 0.8154 2.3715     0
factor(Placebo)1                3.0421 0.3287 29.4533 30.7132 0.5733 4.7968     1
factor(Psychotherapy)1          1.3786 0.7254  5.4602  5.6937 0.8517 2.1737     0
factor(Psychostimulants)1       1.4977 0.6677  7.1786  7.4857 0.8171 2.3616     0
factor(Double_Blind)1           3.2920 0.3038 33.0573 34.4713 0.5512 5.1908     1
factor(Multisite)1              1.2862 0.7775  4.1284  4.3050 0.8817 2.0281     0
factor(Randomized)1             1.3882 0.7204  5.5989  5.8384 0.8487 2.1889     0
factor(BaselineType)2           1.9551 0.5115 13.7762 14.3654 0.7152 3.0829     1
factor(BaselineType)3           1.8839 0.5308 12.7479 13.2932 0.7286 2.9705     0
factor(CokeFreeOutcome_Type)2   2.2468 0.4451 17.9825 18.7517 0.6671 3.5428     1
factor(CokeFreeOutcome_Type)3   1.3664 0.7318  5.2851  5.5112 0.8555 2.1546     0
factor(CokeFreeOutcome_Type)4   1.9602 0.5101 13.8493 14.4416 0.7142 3.0909     1
FU                              1.2306 0.8126  3.3262  3.4685 0.9014 1.9404     0
Year                            1.5316 0.6529  7.6672  7.9952 0.8080 2.4150     0
Proportion_Male                 1.4848 0.6735  6.9921  7.2912 0.8207 2.3412     0
Mean_Age                        1.4792 0.6760  6.9120  7.2077 0.8222 2.3325     0
Mean_daysweek                   1.3896 0.7196  5.6190  5.8594 0.8483 2.1911     0
Years_use                       1.4658 0.6822  6.7183  7.0057 0.8260 2.3113     0
ASI_Drug                        1.6405 0.6096  9.2386  9.6338 0.7807 2.5868     0
Complete                        1.5170 0.6592  7.4567  7.7757 0.8119 2.3920     0

1 --> COLLINEARITY is detected by the test
0 --> COLLINEARITY is not detected by the test

factor(Anticonvulsants)1 , factor(Antidepressants)1 , factor(Antipsychotics)1 ,
factor(DopamineAgonists)1 , factor(MiscMeds)1 , factor(Opioid)1 , factor(Other)1 , factor(Placebo)1
, factor(Psychotherapy)1 , factor(Psychostimulants)1 , factor(Multisite)1 , factor(Randomized)1 ,
factor(CokeFreeOutcome_Type)3 , Proportion_Male , Mean_Age , Mean_daysweek , Years_use , ASI_Drug ,
coefficient(s) are non-significant may be due to multicollinearity

R-square of y on all x: 0.4799

* use method argument to check which regressors may be the reason of collinearity
===================================

[1] Model2

Multivariate Meta-Analysis Model (k = 402; method: REML)

Variance Components:

            estim    sqrt  nlvls  fixed          factor
```

© 2021 Bentzley BS et al. *JAMA Network Open*.

```
sigma^2.1  0.5918  0.7693     98     no          Author
sigma^2.2  0.4885  0.6989    157     no  Author/StudyID

Test for Residual Heterogeneity:
QE(df = 374) = 878.8091, p-val < .0001

Test of Moderators (coefficient(s)
2,3,4,5,6,7,8,9,10,11,12,13,14,15,16,17,18,19,20,21,22,23,24,25,26,27,28):
QM(df = 27) = 304.9178, p-val < .0001

Model Results:

                                 estimate       se     zval    pval      ci.lb    ci.ub
intrcpt                          -53.6869  37.8771  -1.4174  0.1564  -127.9245  20.5508
factor(Anticonvulsants)1          -0.1631   0.3192  -0.5111  0.6093    -0.7887   0.4624
factor(Antidepressants)1           0.2361   0.3584   0.6587  0.5101    -0.4664   0.9386
factor(Antipsychotics)1           -0.0255   0.3874  -0.0658  0.9475    -0.7848   0.7338
factor(ContingencyManagement)1     0.5391   0.1175   4.5898  <.0001     0.3089   0.7693  ***
factor(DopamineAgonists)1         -0.1367   0.3174  -0.4307  0.6667    -0.7587   0.4853
factor(MiscMeds)1                 -0.2277   0.2793  -0.8152  0.4150    -0.7752   0.3198
factor(Opioid)1                    0.0421   0.2335   0.1804  0.8568    -0.4154   0.4997
factor(Other)1                     0.1667   0.2948   0.5654  0.5718    -0.4111   0.7444
factor(Placebo)1                  -0.2042   0.2523  -0.8095  0.4183    -0.6986   0.2902
factor(Psychotherapy)1             0.0377   0.1731   0.2176  0.8278    -0.3015   0.3768
factor(Psychostimulants)1          0.2153   0.3062   0.7029  0.4821    -0.3850   0.8155
factor(Double_Blind)1              0.2227   0.2914   0.7643  0.4447    -0.3484   0.7938
factor(Multisite)1                 0.1259   0.3123   0.4030  0.6869    -0.4863   0.7381
factor(Randomized)1                0.4320   0.3601   1.1996  0.2303    -0.2738   1.1378
factor(BaselineType)2             -1.5897   0.2073  -7.6702  <.0001    -1.9959  -1.1835  ***
factor(BaselineType)3             -1.9878   0.2006  -9.9111  <.0001    -2.3808  -1.5947  ***
factor(CokeFreeOutcome_Type)2     -0.2246   0.2434  -0.9227  0.3562    -0.7016   0.2525
factor(CokeFreeOutcome_Type)3     -0.8915   0.4305  -2.0707  0.0384    -1.7353  -0.0477    *
factor(CokeFreeOutcome_Type)4     -1.0962   0.3017  -3.6330  0.0003    -1.6875  -0.5048  ***
FU                                 0.0121   0.0100   1.2177  0.2233    -0.0074   0.0316
Year                               0.0266   0.0191   1.3974  0.1623    -0.0107   0.0640
Proportion_Male                    0.6070   0.3966   1.5305  0.1259    -0.1703   1.3843
Mean_Age                          -0.0157   0.0207  -0.7555  0.4500    -0.0563   0.0250
Mean_daysweek                      0.0027   0.0599   0.0444  0.9646    -0.1147   0.1201
Years_use                          0.0006   0.0188   0.0301  0.9760    -0.0362   0.0374
ASI_Drug                          -0.1133   0.5166  -0.2194  0.8263    -1.1258   0.8991
Complete                           2.7060   0.3305   8.1880  <.0001     2.0583   3.3538  ***

---
Signif. codes:  0 '***' 0.001 '**' 0.01 '*' 0.05 '.' 0.1 ' ' 1

[1] ICC = 0.548
[1] Model2I2 = 71.5879261626329

[1] Collinearity diagnostics for Model 2

Call:
imcdiag(x = x, y = y, method = method, corr = FALSE, vif = vif,
    tol = tol, conf = conf, cvif = cvif, leamer = leamer, all = all)


All Individual Multicollinearity Diagnostics Result

                                  VIF    TOL      Wi      Fi Leamer   CVIF Klein
factor(Anticonvulsants)1       1.4952 0.6688  7.1417  7.4472 0.8178 1.5336     0
factor(Antidepressants)1       1.4268 0.7009  6.1552  6.4185 0.8372 1.4634     0
factor(Antipsychotics)1        1.3828 0.7232  5.5216  5.7578 0.8504 1.4183     0
factor(ContingencyManagement)1 1.3585 0.7361  5.1702  5.3914 0.8580 1.3934     0
factor(DopamineAgonists)1      1.5988 0.6255  8.6367  9.0062 0.7909 1.6399     0
factor(MiscMeds)1              2.2159 0.4513 17.5374 18.2875 0.6718 2.2728     0
factor(Opioid)1                2.3580 0.4241 19.5859 20.4236 0.6512 2.4185     1
factor(Other)1                 1.4895 0.6714  7.0603  7.3622 0.8194 1.5278     0
factor(Placebo)1               3.0393 0.3290 29.4128 30.6708 0.5736 3.1173     1
factor(Psychotherapy)1         1.3510 0.7402  5.0626  5.2791 0.8603 1.3857     0
factor(Psychostimulants)1      1.4826 0.6745  6.9608  7.2585 0.8213 1.5207     0
```

© 2021 Bentzley BS et al. *JAMA Network Open*.

```
factor(Double_Blind)1          3.0571 0.3271 29.6693 30.9384 0.5719 3.1356     1
factor(Multisite)1             1.3982 0.7152  5.7438  5.9894 0.8457 1.4341     0
factor(Randomized)1            1.4222 0.7031  6.0897  6.3502 0.8385 1.4587     0
factor(BaselineType)2          1.9306 0.5180 13.4224 13.9966 0.7197 1.9802     0
factor(BaselineType)3          1.7228 0.5804 10.4252 10.8711 0.7619 1.7671     0
factor(CokeFreeOutcome_Type)2  2.1205 0.4716 16.1608 16.8520 0.6867 2.1749     0
factor(CokeFreeOutcome_Type)3  1.5603 0.6409  8.0806  8.4263 0.8006 1.6003     0
factor(CokeFreeOutcome_Type)4  2.5241 0.3962 21.9829 22.9232 0.6294 2.5890     1
FU                             1.2642 0.7910  3.8102  3.9732 0.8894 1.2966     0
Year                           1.5696 0.6371  8.2160  8.5674 0.7982 1.6099     0
Proportion_Male                1.5333 0.6522  7.6913  8.0203 0.8076 1.5726     0
Mean_Age                       1.6699 0.5988  9.6621 10.0754 0.7738 1.7128     0
Mean_daysweek                  1.4628 0.6836  6.6745  6.9599 0.8268 1.5003     0
Years_use                      1.8348 0.5450 12.0397 12.5546 0.7383 1.8819     0
ASI_Drug                       1.8447 0.5421 12.1827 12.7038 0.7363 1.8920     0
Complete                       1.5074 0.6634  7.3179  7.6309 0.8145 1.5461     0

1 --> COLLINEARITY is detected by the test
0 --> COLLINEARITY is not detected by the test

factor(Anticonvulsants)1 , factor(Antidepressants)1 , factor(Antipsychotics)1 ,
factor(DopamineAgonists)1 , factor(MiscMeds)1 , factor(Opioid)1 , factor(Other)1 , factor(Placebo)1
, factor(Psychotherapy)1 , factor(Psychostimulants)1 , factor(Double_Blind)1 , factor(Multisite)1 ,
factor(Randomized)1 , FU , Proportion_Male , Mean_daysweek , Years_use , ASI_Drug , coefficient(s)
are non-significant may be due to multicollinearity

R-square of y on all x: 0.5673

* use method argument to check which regressors may be the reason of collinearity
===================================

[1] Model3

Multivariate Meta-Analysis Model (k = 402; method: REML)

Variance Components:

            estim    sqrt  nlvls  fixed          factor
sigma^2.1  0.5716  0.7560     98     no          Author
sigma^2.2  0.5447  0.7380    157     no  Author/StudyID

Test for Residual Heterogeneity:
QE(df = 374) = 875.5325, p-val < .0001

Test of Moderators (coefficient(s)
2,3,4,5,6,7,8,9,10,11,12,13,14,15,16,17,18,19,20,21,22,23,24,25,26,27,28):
QM(df = 27) = 253.7804, p-val < .0001

Model Results:

                                estimate       se     zval    pval     ci.lb    ci.ub
intrcpt                          -8.2694  38.3076  -0.2159  0.8291  -83.3509  66.8121
factor(Anticonvulsants)1          0.1396   0.3210   0.4348  0.6637   -0.4896   0.7688
factor(Antidepressants)1          0.0981   0.3490   0.2812  0.7786   -0.5860   0.7823
factor(Antipsychotics)1          -0.2276   0.3905  -0.5828  0.5600   -0.9929   0.5378
factor(ContingencyManagement)1    0.6967   0.1156   6.0295  <.0001    0.4702   0.9232  ***
factor(DopamineAgonists)1        -0.1179   0.3160  -0.3730  0.7092   -0.7371   0.5014
factor(MiscMeds)1                -0.1623   0.2791  -0.5816  0.5608   -0.7093   0.3847
factor(Opioid)1                   0.1490   0.2253   0.6612  0.5085   -0.2926   0.5905
factor(Other)1                    0.0367   0.2963   0.1238  0.9014   -0.5441   0.6175
factor(Placebo)1                 -0.2478   0.2503  -0.9900  0.3222   -0.7383   0.2428
factor(Psychotherapy)1           -0.0811   0.1752  -0.4631  0.6433   -0.4245   0.2622
factor(Psychostimulants)1         0.1496   0.3049   0.4907  0.6237   -0.4480   0.7472
factor(Double_Blind)1            -0.1547   0.2929  -0.5282  0.5974   -0.7288   0.4194
factor(Multisite)1                0.2394   0.3122   0.7668  0.4432   -0.3725   0.8513
factor(Randomized)1               0.2369   0.3644   0.6500  0.5157   -0.4774   0.9512
factor(BaselineType)2            -1.2602   0.2179  -5.7830  <.0001   -1.6874  -0.8331  ***
factor(BaselineType)3            -1.4074   0.2027  -6.9449  <.0001   -1.8046  -1.0102  ***
factor(CokeFreeOutcome_Type)2    -0.1989   0.2459  -0.8088  0.4186   -0.6810   0.2831
```

© 2021 Bentzley BS et al. *JAMA Network Open*.

```
factor(CokeFreeOutcome_Type)3    -0.7863   0.4652  -1.6901  0.0910   -1.6981   0.1255    .
factor(CokeFreeOutcome_Type)4    -1.2362   0.2965  -4.1695  <.0001   -1.8173  -0.6551  ***
FU                                0.0196   0.0100   1.9557  0.0505   -0.0000   0.0392    .
Year                              0.0031   0.0193   0.1589  0.8738   -0.0347   0.0408
Proportion_Male                   0.9844   0.4008   2.4561  0.0140    0.1988   1.7699    *
Mean_Age                          0.0149   0.0207   0.7194  0.4719   -0.0257   0.0556
Mean_daysweek                     0.1549   0.0606   2.5570  0.0106    0.0362   0.2736    *
Years_use                         0.0044   0.0197   0.2234  0.8232   -0.0342   0.0430
ASI_Drug                         -0.3329   0.4529  -0.7349  0.4624   -1.2206   0.5549
Complete                          2.6701   0.3339   7.9979  <.0001    2.0158   3.3245  ***

---
Signif. codes:  0 '***' 0.001 '**' 0.01 '*' 0.05 '.' 0.1 ' ' 1

[1] ICC = 0.512
[1] Model3I2 = 72.7824694740359

[1] Collinearity diagnostics for Model 3

Call:
imcdiag(x = x, y = y, method = method, corr = FALSE, vif = vif,
    tol = tol, conf = conf, cvif = cvif, leamer = leamer, all = all)


All Individual Multicollinearity Diagnostics Result

                                   VIF    TOL      Wi      Fi Leamer   CVIF Klein
factor(Anticonvulsants)1        1.4893 0.6714  7.0575  7.3593 0.8194 2.2391     0
factor(Antidepressants)1        1.4420 0.6935  6.3745  6.6472 0.8328 2.1679     0
factor(Antipsychotics)1         1.4228 0.7028  6.0981  6.3589 0.8384 2.1391     0
factor(ContingencyManagement)1  1.3772 0.7261  5.4411  5.6738 0.8521 2.0706     0
factor(DopamineAgonists)1       1.5852 0.6308  8.4410  8.8021 0.7942 2.3833     0
factor(MiscMeds)1               2.2172 0.4510 17.5560 18.3070 0.6716 3.3334     0
factor(Opioid)1                 2.0919 0.4780 15.7481 16.4217 0.6914 3.1449     0
factor(Other)1                  1.4725 0.6791  6.8153  7.1069 0.8241 2.2138     0
factor(Placebo)1                3.0632 0.3265 29.7572 31.0301 0.5714 4.6052     1
factor(Psychotherapy)1          1.3894 0.7197  5.6160  5.8562 0.8484 2.0888     0
factor(Psychostimulants)1       1.5006 0.6664  7.2202  7.5291 0.8163 2.2560     0
factor(Double_Blind)1           3.2153 0.3110 31.9508 33.3174 0.5577 4.8339     1
factor(Multisite)1              1.3705 0.7296  5.3441  5.5726 0.8542 2.0605     0
factor(Randomized)1             1.4024 0.7130  5.8045  6.0528 0.8444 2.1085     0
factor(BaselineType)2           2.0056 0.4986 14.5039 15.1243 0.7061 3.0152     0
factor(BaselineType)3           2.0617 0.4850 15.3133 15.9683 0.6964 3.0996     0
factor(CokeFreeOutcome_Type)2   2.0371 0.4909 14.9588 15.5987 0.7006 3.0627     0
factor(CokeFreeOutcome_Type)3   1.6676 0.5997  9.6290 10.0409 0.7744 2.5071     0
factor(CokeFreeOutcome_Type)4   2.3050 0.4338 18.8219 19.6270 0.6587 3.4653     0
FU                              1.2399 0.8065  3.4599  3.6079 0.8981 1.8641     0
Year                            1.5890 0.6293  8.4953  8.8587 0.7933 2.3889     0
Proportion_Male                 1.4287 0.6999  6.1834  6.4478 0.8366 2.1479     0
Mean_Age                        1.5845 0.6311  8.4306  8.7912 0.7944 2.3822     0
Mean_daysweek                   1.6131 0.6199  8.8435  9.2217 0.7873 2.4252     0
Years_use                       1.9593 0.5104 13.8356 14.4274 0.7144 2.9456     0
ASI_Drug                        1.4401 0.6944  6.3479  6.6194 0.8333 2.1651     0
Complete                        1.5483 0.6459  7.9080  8.2463 0.8037 2.3277     0

1 --> COLLINEARITY is detected by the test
0 --> COLLINEARITY is not detected by the test

factor(Anticonvulsants)1 , factor(Antidepressants)1 , factor(Antipsychotics)1 ,
factor(DopamineAgonists)1 , factor(MiscMeds)1 , factor(Opioid)1 , factor(Other)1 , factor(Placebo)1
, factor(Psychotherapy)1 , factor(Psychostimulants)1 , factor(Double_Blind)1 , factor(Multisite)1 ,
factor(Randomized)1 , factor(CokeFreeOutcome_Type)2 , Year , Mean_Age , Mean_daysweek , ASI_Drug ,
coefficient(s) are non-significant may be due to multicollinearity

R-square of y on all x: 0.5821

* use method argument to check which regressors may be the reason of collinearity
===================================
```

© 2021 Bentzley BS et al. *JAMA Network Open*.

```
[1] Model4

Multivariate Meta-Analysis Model (k = 402; method: REML)

Variance Components:

            estim    sqrt  nlvls  fixed          factor
sigma^2.1  0.4262  0.6529     98     no          Author
sigma^2.2  0.7074  0.8411    157     no  Author/StudyID

Test for Residual Heterogeneity:
QE(df = 374) = 848.2921, p-val < .0001

Test of Moderators (coefficient(s)
2,3,4,5,6,7,8,9,10,11,12,13,14,15,16,17,18,19,20,21,22,23,24,25,26,27,28):
QM(df = 27) = 190.1784, p-val < .0001

Model Results:

                                estimate       se     zval    pval      ci.lb    ci.ub
intrcpt                         -27.8369  41.5269  -0.6703  0.5026  -109.2282  53.5544
factor(Anticonvulsants)1         -0.0367   0.3461  -0.1062  0.9154    -0.7151   0.6416
factor(Antidepressants)1          0.1737   0.3788   0.4584  0.6466    -0.5688   0.9161
factor(Antipsychotics)1          -0.2175   0.4084  -0.5326  0.5943    -1.0180   0.5830
factor(ContingencyManagement)1    0.6132   0.1313   4.6694  <.0001     0.3558   0.8706  ***
factor(DopamineAgonists)1        -0.1425   0.3290  -0.4332  0.6648    -0.7873   0.5022
factor(MiscMeds)1                -0.2456   0.2982  -0.8235  0.4102    -0.8301   0.3389
factor(Opioid)1                  -0.1019   0.2362  -0.4316  0.6661    -0.5648   0.3610
factor(Other)1                   -0.0203   0.3276  -0.0620  0.9505    -0.6624   0.6217
factor(Placebo)1                 -0.3359   0.2634  -1.2751  0.2023    -0.8522   0.1804
factor(Psychotherapy)1           -0.0670   0.1969  -0.3406  0.7334    -0.4529   0.3188
factor(Psychostimulants)1         0.3143   0.3194   0.9840  0.3251    -0.3117   0.9403
factor(Double_Blind)1            -0.1646   0.3097  -0.5316  0.5950    -0.7716   0.4424
factor(Multisite)1                0.0493   0.3500   0.1409  0.8880    -0.6367   0.7353
factor(Randomized)1              -0.2831   0.3972  -0.7128  0.4760    -1.0615   0.4954
factor(BaselineType)2            -1.6361   0.2411  -6.7869  <.0001    -2.1086  -1.1636  ***
factor(BaselineType)3            -0.9993   0.2525  -3.9573  <.0001    -1.4942  -0.5044  ***
factor(CokeFreeOutcome_Type)2     0.5004   0.2848   1.7572  0.0789    -0.0578   1.0586    .
factor(CokeFreeOutcome_Type)3     0.0131   0.4475   0.0293  0.9767    -0.8640   0.8902
factor(CokeFreeOutcome_Type)4     1.0985   0.3163   3.4733  0.0005     0.4786   1.7184  ***
FU                                0.0324   0.0116   2.7899  0.0053     0.0096   0.0552   **
Year                              0.0139   0.0209   0.6647  0.5062    -0.0270   0.0548
Proportion_Male                   0.6748   0.4233   1.5944  0.1108    -0.1547   1.5044
Mean_Age                         -0.0287   0.0207  -1.3862  0.1657    -0.0692   0.0119
Mean_daysweek                     0.1681   0.0636   2.6445  0.0082     0.0435   0.2927   **
Years_use                         0.0044   0.0196   0.2256  0.8215    -0.0341   0.0429
ASI_Drug                          0.5253   0.8381   0.6269  0.5308    -1.1172   2.1679
Complete                          2.6427   0.3704   7.1347  <.0001     1.9167   3.3687  ***

---
Signif. codes:  0 '***' 0.001 '**' 0.01 '*' 0.05 '.' 0.1 ' ' 1

[1] ICC = 0.376
[1] Model4I2 = 68.3514959518097

[1] Collinearity diagnostics for Model 4

Call:
imcdiag(x = x, y = y, method = method, corr = FALSE, vif = vif,
    tol = tol, conf = conf, cvif = cvif, leamer = leamer, all = all)


All Individual Multicollinearity Diagnostics Result

                                  VIF    TOL      Wi      Fi Leamer   CVIF Klein
factor(Anticonvulsants)1       1.5457 0.6470  7.8700  8.2066 0.8043 2.2177     0
factor(Antidepressants)1       1.4642 0.6830  6.6946  6.9809 0.8264 2.1008     0
factor(Antipsychotics)1        1.4013 0.7136  5.7873  6.0348 0.8448 2.0106     0
factor(ContingencyManagement)1 1.3508 0.7403  5.0591  5.2755 0.8604 1.9381     0
```

© 2021 Bentzley BS et al. *JAMA Network Open*.

```
factor(DopamineAgonists)1      1.5890 0.6293  8.4948  8.8582 0.7933 2.2799     0
factor(MiscMeds)1              2.2594 0.4426 18.1640 18.9410 0.6653 3.2418     1
factor(Opioid)1                1.9329 0.5174 13.4552 14.0307 0.7193 2.7734     0
factor(Other)1                 1.5120 0.6614  7.3853  7.7012 0.8132 2.1695     0
factor(Placebo)1               3.1213 0.3204 30.5951 31.9037 0.5660 4.4785     1
factor(Psychotherapy)1         1.3892 0.7198  5.6138  5.8540 0.8484 1.9933     0
factor(Psychostimulants)1      1.4870 0.6725  7.0247  7.3252 0.8200 2.1337     0
factor(Double_Blind)1          3.0489 0.3280 29.5516 30.8157 0.5727 4.3747     1
factor(Multisite)1             1.4895 0.6714  7.0597  7.3616 0.8194 2.1371     0
factor(Randomized)1            1.4450 0.6921  6.4177  6.6922 0.8319 2.0733     0
factor(BaselineType)2          1.8839 0.5308 12.7492 13.2945 0.7286 2.7031     0
factor(BaselineType)3          1.7260 0.5794 10.4715 10.9194 0.7612 2.4765     0
factor(CokeFreeOutcome_Type)2  2.6571 0.3764 23.9005 24.9228 0.6135 3.8125     1
factor(CokeFreeOutcome_Type)3  1.3493 0.7411  5.0377  5.2532 0.8609 1.9360     0
factor(CokeFreeOutcome_Type)4  2.2290 0.4486 17.7260 18.4842 0.6698 3.1982     1
FU                             1.2697 0.7876  3.8903  4.0567 0.8875 1.8218     0
Year                           1.7230 0.5804 10.4281 10.8741 0.7618 2.4722     0
Proportion_Male                1.4179 0.7053  6.0273  6.2851 0.8398 2.0344     0
Mean_Age                       1.3934 0.7177  5.6740  5.9167 0.8472 1.9993     0
Mean_daysweek                  1.3241 0.7552  4.6742  4.8741 0.8690 1.8998     0
Years_use                      1.2401 0.8064  3.4628  3.6109 0.8980 1.7793     0
ASI_Drug                       1.9449 0.5142 13.6289 14.2118 0.7170 2.7906     0
Complete                       1.5654 0.6388  8.1550  8.5038 0.7993 2.2461     0

1 --> COLLINEARITY is detected by the test
0 --> COLLINEARITY is not detected by the test

factor(Anticonvulsants)1 , factor(Antidepressants)1 , factor(Antipsychotics)1 ,
factor(ContingencyManagement)1 , factor(DopamineAgonists)1 , factor(MiscMeds)1 , factor(Opioid)1 ,
factor(Other)1 , factor(Psychotherapy)1 , factor(Psychostimulants)1 , factor(Double_Blind)1 ,
factor(Multisite)1 , factor(Randomized)1 , factor(CokeFreeOutcome_Type)3 , Year , Proportion_Male ,
Mean_Age , Years_use , ASI_Drug , coefficient(s) are non-significant may be due to
multicollinearity

R-square of y on all x: 0.5084

* use method argument to check which regressors may be the reason of collinearity
===================================

[1] Model5

Multivariate Meta-Analysis Model (k = 402; method: REML)

Variance Components:

            estim    sqrt  nlvls  fixed           factor
sigma^2.1  0.1245  0.3529     98     no           Author
sigma^2.2  1.0443  1.0219    157     no  Author/StudyID

Test for Residual Heterogeneity:
QE(df = 374) = 929.9673, p-val < .0001

Test of Moderators (coefficient(s)
2,3,4,5,6,7,8,9,10,11,12,13,14,15,16,17,18,19,20,21,22,23,24,25,26,27,28):
QM(df = 27) = 198.4783, p-val < .0001

Model Results:

                                estimate       se     zval    pval     ci.lb    ci.ub
intrcpt                         -16.2073  41.0720  -0.3946  0.6931  -96.7070  64.2923
factor(Anticonvulsants)1          0.2205   0.3448   0.6395  0.5225   -0.4553   0.8963
factor(Antidepressants)1          0.3921   0.3822   1.0259  0.3049   -0.3570   1.1413
factor(Antipsychotics)1          -0.4160   0.4011  -1.0372  0.2996   -1.2021   0.3701
factor(ContingencyManagement)1    0.6595   0.1262   5.2272  <.0001    0.4122   0.9068  ***
factor(DopamineAgonists)1        -0.1726   0.3309  -0.5216  0.6019   -0.8213   0.4760
factor(MiscMeds)1                -0.1214   0.2947  -0.4122  0.6802   -0.6990   0.4561
factor(Opioid)1                   0.3590   0.2298   1.5623  0.1182   -0.0914   0.8094
factor(Other)1                    0.0644   0.3042   0.2118  0.8322   -0.5318   0.6607
factor(Placebo)1                 -0.2158   0.2633  -0.8196  0.4124   -0.7318   0.3002
```

© 2021 Bentzley BS et al. *JAMA Network Open*.

```
factor(Psychotherapy)1            -0.0003   0.1838  -0.0016  0.9987   -0.3605   0.3599
factor(Psychostimulants)1          0.3049   0.3193   0.9551  0.3395   -0.3208   0.9306
factor(Double_Blind)1              0.4158   0.3025   1.3747  0.1692   -0.1770   1.0087
factor(Multisite)1                -0.3247   0.3479  -0.9333  0.3506   -1.0065   0.3571
factor(Randomized)1               -0.6251   0.3957  -1.5799  0.1141   -1.4007   0.1504
factor(BaselineType)2             -1.2107   0.2232  -5.4241  <.0001   -1.6482  -0.7732  ***
factor(BaselineType)3             -0.3264   0.2317  -1.4086  0.1590   -0.7805   0.1278
factor(CokeFreeOutcome_Type)2     -0.2757   0.2989  -0.9225  0.3563   -0.8615   0.3101
factor(CokeFreeOutcome_Type)3     -1.1853   0.4558  -2.6003  0.0093   -2.0788  -0.2919   **
factor(CokeFreeOutcome_Type)4      0.9762   0.3214   3.0371  0.0024    0.3462   1.6062   **
FU                                 0.0244   0.0115   2.1194  0.0341    0.0018   0.0470    *
Year                               0.0074   0.0206   0.3605  0.7185   -0.0330   0.0479
Proportion_Male                    0.7174   0.4220   1.6998  0.0892   -0.1098   1.5445    .
Mean_Age                           0.0034   0.0208   0.1639  0.8698   -0.0374   0.0442
Mean_daysweek                      0.1000   0.0676   1.4781  0.1394   -0.0326   0.2325
Years_use                          0.0079   0.0195   0.4030  0.6870   -0.0304   0.0462
ASI_Drug                           0.1406   0.5208   0.2700  0.7872   -0.8801   1.1613
Complete                           2.3499   0.3653   6.4322  <.0001    1.6338   3.0659  ***

---
Signif. codes:  0 '***' 0.001 '**' 0.01 '*' 0.05 '.' 0.1 ' ' 1

[1] ICC = 0.107
[1] Model5I2 = 71.1384534650972

[1] Collinearity diagnostics for Model 5

Call:
imcdiag(x = x, y = y, method = method, corr = FALSE, vif = vif,
    tol = tol, conf = conf, cvif = cvif, leamer = leamer, all = all)


All Individual Multicollinearity Diagnostics Result

                                   VIF    TOL      Wi      Fi Leamer   CVIF Klein
factor(Anticonvulsants)1        1.5278 0.6545  7.6126  7.9383 0.8090 2.5635     0
factor(Antidepressants)1        1.4345 0.6971  6.2673  6.5354 0.8349 2.4070     0
factor(Antipsychotics)1         1.4288 0.6999  6.1848  6.4493 0.8366 2.3974     0
factor(ContingencyManagement)1  1.3794 0.7249  5.4728  5.7069 0.8514 2.3146     0
factor(DopamineAgonists)1       1.5824 0.6319  8.4003  8.7596 0.7949 2.6552     0
factor(MiscMeds)1               2.2391 0.4466 17.8713 18.6358 0.6683 3.7570     1
factor(Opioid)1                 1.8683 0.5352 12.5238 13.0594 0.7316 3.1349     0
factor(Other)1                  1.4926 0.6700  7.1047  7.4086 0.8185 2.5044     0
factor(Placebo)1                3.0955 0.3230 30.2238 31.5166 0.5684 5.1940     1
factor(Psychotherapy)1          1.4080 0.7102  5.8842  6.1359 0.8428 2.3624     0
factor(Psychostimulants)1       1.4849 0.6734  6.9944  7.2936 0.8206 2.4916     0
factor(Double_Blind)1           2.9566 0.3382 28.2199 29.4270 0.5816 4.9609     1
factor(Multisite)1              1.3628 0.7338  5.2330  5.4568 0.8566 2.2867     0
factor(Randomized)1             1.4199 0.7043  6.0563  6.3154 0.8392 2.3825     0
factor(BaselineType)2           1.9921 0.5020 14.3096 14.9216 0.7085 3.3426     1
factor(BaselineType)3           2.0068 0.4983 14.5207 15.1418 0.7059 3.3672     1
factor(CokeFreeOutcome_Type)2   3.0369 0.3293 29.3777 30.6343 0.5738 5.0956     1
factor(CokeFreeOutcome_Type)3   1.3292 0.7523  4.7486  4.9517 0.8674 2.2303     0
factor(CokeFreeOutcome_Type)4   2.1059 0.4749 15.9501 16.6324 0.6891 3.5335     1
FU                              1.2335 0.8107  3.3681  3.5122 0.9004 2.0697     0
Year                            1.6533 0.6049  9.4224  9.8254 0.7777 2.7741     0
Proportion_Male                 1.4196 0.7044  6.0524  6.3113 0.8393 2.3820     0
Mean_Age                        1.4054 0.7115  5.8469  6.0970 0.8435 2.3581     0
Mean_daysweek                   1.4031 0.7127  5.8143  6.0630 0.8442 2.3543     0
Years_use                       1.7186 0.5819 10.3648 10.8081 0.7628 2.8837     0
ASI_Drug                        2.2982 0.4351 18.7233 19.5242 0.6596 3.8561     1
Complete                        1.5202 0.6578  7.5026  7.8235 0.8111 2.5507     0

1 --> COLLINEARITY is detected by the test
0 --> COLLINEARITY is not detected by the test

factor(Anticonvulsants)1 , factor(Antidepressants)1 , factor(Antipsychotics)1 ,
factor(DopamineAgonists)1 , factor(MiscMeds)1 , factor(Other)1 , factor(Placebo)1 ,
factor(Psychotherapy)1 , factor(Psychostimulants)1 , factor(Double_Blind)1 , factor(Multisite)1 ,
```

© 2021 Bentzley BS et al. *JAMA Network Open*.

```
factor(CokeFreeOutcome_Type)2 , Year , Mean_Age , Mean_daysweek , Years_use , ASI_Drug ,
coefficient(s) are non-significant may be due to multicollinearity

R-square of y on all x: 0.485

* use method argument to check which regressors may be the reason of collinearity
====================================

[1] Pooled Results
                                          est          se           t         df     Pr(>|t|)
lo 95       hi 95 nmis
intrcpt                         -34.097987842 48.27044216 -0.70639477  37.857740 4.842677e-01 -
1.318284e+02 63.63247374   NA
factor(Anticonvulsants)1          0.115818029  0.41784647  0.27717843  28.572466 7.836386e-01 -
7.393297e-01  0.97096580   NA
factor(Antidepressants)1          0.215154030  0.39085017  0.55047700 190.047517 5.826387e-01 -
5.558077e-01  0.98611578   NA
factor(Antipsychotics)1          -0.241687050  0.43553775 -0.55491642 132.476182 5.798876e-01 -
1.103195e+00  0.61982103   NA
factor(ContingencyManagement)1    0.637781370  0.14243518  4.47769554  56.536803 3.711791e-05
3.525092e-01  0.92305355   NA
factor(DopamineAgonists)1        -0.093583011  0.34910282 -0.26806719 148.500996 7.890196e-01 -
7.834338e-01  0.59626774   NA
factor(MiscMeds)1                -0.143916495  0.31716930 -0.45375292 111.326331 6.508900e-01 -
7.723883e-01  0.48455534   NA
factor(Opioid)1                   0.114973780  0.29958962  0.38377091  24.931905 7.044016e-01 -
5.021281e-01  0.73207561   NA
factor(Other)1                    0.045406318  0.32394027  0.14016880 247.431394 8.886407e-01 -
5.926257e-01  0.68343839   NA
factor(Placebo)1                 -0.223713055  0.27510297 -0.81319754 180.765257 4.171746e-01 -
7.665392e-01  0.31911305   NA
factor(Psychotherapy)1           -0.037615979  0.19541312 -0.19249464 201.850777 8.475483e-01 -
4.229289e-01  0.34769691   NA
factor(Psychostimulants)1         0.308500622  0.35939394  0.85839127  62.444429 3.939594e-01 -
4.098159e-01  1.02681718   NA
factor(Double_Blind)1            -0.025703282  0.48596819 -0.05289087  10.410016 9.588201e-01 -
1.102755e+00  1.05134819   NA
factor(Multisite)1                0.085255912  0.43772651  0.19476982  22.104767 8.473522e-01 -
8.222839e-01  0.99279573   NA
factor(Randomized)1              -0.031506711  0.60374099 -0.05218581  10.728214 9.593396e-01 -
1.364450e+00  1.30143704   NA
factor(BaselineType)2            -1.386003553  0.32400133 -4.27777123  14.899684 6.701933e-04 -
2.077001e+00 -0.69500589   NA
factor(BaselineType)3            -0.960720049  0.88377489 -1.08706421   3.880078 3.398770e-01 -
3.444673e+00  1.52323300   NA
factor(CokeFreeOutcome_Type)2     0.073855531  0.53841487  0.13717216   6.854665 8.948367e-01 -
1.204786e+00  1.35249699   NA
factor(CokeFreeOutcome_Type)3    -0.472941367  0.89066435 -0.53099842   6.946993 6.119662e-01 -
2.582290e+00  1.63640720   NA
factor(CokeFreeOutcome_Type)4     0.087029672  1.30305671  0.06678886   3.728623 9.501759e-01 -
3.637086e+00  3.81114559   NA
FU                                0.024718703  0.01514238  1.63241843  17.316998 1.206413e-01 -
7.184438e-03  0.05662184    0
Year                              0.016551678  0.02435471  0.67960890  36.660928 5.010210e-01 -
3.281106e-02  0.06591442    0
Proportion_Male                   0.801982262  0.46672377  1.71832316  75.035505 8.986159e-02 -
1.277720e-01  1.73173648   11
Mean_Age                         -0.008654739  0.02844550 -0.30425684  17.678523 7.644845e-01 -
6.849452e-02  0.05118504   10
Mean_daysweek                     0.089927630  0.10339815  0.86972182   9.430174 4.060454e-01 -
1.423588e-01  0.32221409  128
Years_use                        -0.000291333  0.02290343 -0.01272006  49.225874 9.899025e-01 -
4.631215e-02  0.04572948  166
ASI_Drug                         -0.037782728  0.72778119 -0.05191495  32.349712 9.589157e-01 -
1.519596e+00  1.44403079  273
Complete                          2.623178930  0.39553540  6.63197011  80.469772 3.508446e-09
1.836109e+00  3.41024892    7
                                      fmi     lambda
intrcpt                        0.33472115 0.30047938
```

© 2021 Bentzley BS et al. *JAMA Network Open*.

```
factor(Anticonvulsants)1       0.39240330 0.35131125
factor(Antidepressants)1       0.10506271 0.09569397
factor(Antipsychotics)1        0.14616246 0.13336861
factor(ContingencyManagement)1 0.26358948 0.23799159
factor(DopamineAgonists)1      0.13284467 0.12124400
factor(MiscMeds)1              0.16728655 0.15245986
factor(Opioid)1                0.42285236 0.37833981
factor(Other)1                 0.07547906 0.06803619
factor(Placebo)1               0.11063222 0.10084632
factor(Psychotherapy)1         0.09837507 0.08948553
factor(Psychostimulants)1      0.24776678 0.22405364
factor(Double_Blind)1          0.65788407 0.59791642
factor(Multisite)1             0.45115128 0.40364171
factor(Randomized)1            0.64871599 0.58881190
factor(BaselineType)2          0.55326797 0.49707415
factor(BaselineType)3          0.95246607 0.93298526
factor(CokeFreeOutcome_Type)2  0.79012727 0.73668826
factor(CokeFreeOutcome_Type)3  0.78580860 0.73190359
factor(CokeFreeOutcome_Type)4  0.95924459 0.94200685
FU                             0.51266287 0.45945141
Year                           0.34096501 0.30596667
Proportion_Male                0.22019423 0.19968260
Mean_Age                       0.50724306 0.45448119
Mean_daysweek                  0.68840926 0.62866131
Years_use                      0.28677969 0.25837917
ASI_Drug                       0.36610943 0.32809467
Complete                       0.21022826 0.19084016
```

© 2021 Bentzley BS et al. *JAMA Network Open*.

Egger's Test of Bias

```
#Author: Brandon S Bentzley
#Project: Meta analysis of treatments for cocaine use disorders
#Date this R script was last updated: 02/15/2021
#Description: Egger tests for bias, aka regression of funnel plots


# store the current directory
initial.dir<-getwd()
# change to the new directory
setwd("/Users/bsbentzley/Documents/Stanford/STAAR/2015.12.03 - DBS cocaine/Ranalysis")
# load the necessary libraries
library(gdata)
library(metafor)
# set the output file
sink("Output.out")
# set file width
options(width=120)
# load the dataset and define variable data types
mydata = read.csv("Data_imputed_long.csv", colClasses=c(
"Author"="character", "Year"="numeric", "Title"="character", "StudyID"="factor",
"Double_Blind"="factor", "Multisite"="factor", "Randomized"="factor", "SubDsrdr"="character",
"Treatments"="character", "Placebo"="factor", "Psychotherapy"="factor",
"ContingencyManagement"="factor", "Other"="factor", "Opioid"="factor", "MiscMeds"="factor",
"Psychostimulants"="factor", "Anticonvulsants"="factor", "DopamineAgonists"="factor",
"Antidepressants"="factor", "Antipsychotics"="factor", "TreatmentNmbr"= "numeric",
"Trtmnt1orMore"="factor", "N_start"="numeric", "Proportion_Male"="numeric", "Mean_Age"="numeric",
"Mean_daysweek"="numeric", "Proportion_30days"="numeric", "Years_use"="numeric",
"ASI_Drug"="numeric", "TestPosScreen"="factor", "CokeFreeBase"="numeric", "CokeFreeWk1"="numeric",
"BaselineType"="factor", "CokeFreeStart"="numeric", "Complication"="character", "FU"="numeric",
"Complete"="numeric", "CokeFreeLastWk_NotITT"="numeric", "CokeFreeMean_NotITT"="numeric",
"CokeFreeLastWk_ITT"="numeric", "CokeFreeMean_ITT"="numeric", "CokeFreeOutcome_Type"="factor",
"CokeFreeOutcome_ITT"="numeric", "CFS"="numeric", "CPS"="numeric", "CFE"="numeric",
"CPE"="numeric", "yi"="numeric", "vi"="numeric"))

#Loop through all treatment groups
for (i in c(1:11)){

#Select only data from a particular treatment category
if (i==1) {TRT="Placebo"
mydata_sub<-subset(mydata, Placebo==1)}
if (i==2) {TRT="Psychotherapy"
mydata_sub<-subset(mydata, Psychotherapy==1)}
if (i==3) {TRT="ContingencyManagement"
mydata_sub<-subset(mydata, ContingencyManagement==1)}
if (i==4) {TRT="Other"
mydata_sub<-subset(mydata, Other==1)}
if (i==5) {TRT="Opioid"
mydata_sub<-subset(mydata, Opioid==1)}
if (i==6) {TRT="MiscMeds"
mydata_sub<-subset(mydata, MiscMeds==1)}
if (i==7) {TRT="Psychostimulants"
mydata_sub<-subset(mydata, Psychostimulants==1)}
if (i==8) {TRT="Anticonvulsants"
mydata_sub<-subset(mydata, Anticonvulsants==1)}
if (i==9) {TRT="DopamineAgonists"
```

© 2021 Bentzley BS et al. *JAMA Network Open*.

```
mydata_sub<-subset(mydata, DopamineAgonists==1)}
if (i==10) {TRT="Antidepressants"
mydata_sub<-subset(mydata, Antidepressants==1)}
if (i==11) {TRT="Antipsychotics"
mydata_sub<-subset(mydata, Antipsychotics==1)}


    #Loop through non-imputed and imputed data
    for (j in c(0:5)){

    #subset of each dataset,  #0 has native missing data, 1-5 are imputed datasets
    mydata_subsub<-subset(mydata_sub, .imp==j)

    ###Multi-level mixed effects models for Egger Tests
    test.egger = rma.mv(yi,vi, mod = vi, random = ~ 1 | Author/StudyID, data = mydata_subsub)

    ####Egger test for bias
    print(TRT)
    print(j) #0 has native missing data, 1-5 are imputed datasets
    print(test.egger)
    }

} #End For Loop

#Multilevel full analysis,

#Loop through non-imputed and imputed data for full dataset with all treatment categories
for (k in c(0:5)){

#subset of each dataset
mydata_subfull<-subset(mydata, .imp==k)
test.egger = rma.mv(yi,vi, mod = vi, random = ~ 1 | Author/StudyID, data = mydata_subfull)


####Egger test for bias
print("Full Model")
print(k)  #0 has native missing data, 1-5 are imputed datasets
print(test.egger)
} # End for loop

# close the output file
sink()
# unload the libraries
detach("package:gdata")
detach("package:metafor")
# change back to the original directory
setwd(initial.dir)

*******

[1] "Placebo"
[1] 0
```

© 2021 Bentzley BS et al. *JAMA Network Open*.

```
Multivariate Meta-Analysis Model (k = 78; method: REML)

Variance Components:

            estim    sqrt  nlvls  fixed          factor
sigma^2.1  0.8280  0.9100     42     no          Author
sigma^2.2  0.3591  0.5992     65     no  Author/StudyID

Test for Residual Heterogeneity:
QE(df = 76) = 228.3277, p-val < .0001

Test of Moderators (coefficient(s) 2):
QM(df = 1) = 16.6029, p-val < .0001

Model Results:

         estimate      se     zval    pval    ci.lb    ci.ub
intrcpt   -0.6630  0.3154  -2.1024  0.0355  -1.2811  -0.0449    *
mods       1.3385  0.3285   4.0747  <.0001   0.6946   1.9823  ***

---
Signif. codes:  0 '***' 0.001 '**' 0.01 '*' 0.05 '.' 0.1 ' ' 1

[1] "Placebo"
[1] 1

Multivariate Meta-Analysis Model (k = 96; method: REML)

Variance Components:

            estim    sqrt  nlvls  fixed          factor
sigma^2.1  1.1237  1.0600     51     no          Author
sigma^2.2  0.2659  0.5157     80     no  Author/StudyID

Test for Residual Heterogeneity:
QE(df = 94) = 290.0080, p-val < .0001

Test of Moderators (coefficient(s) 2):
QM(df = 1) = 21.1484, p-val < .0001

Model Results:

         estimate      se     zval    pval    ci.lb   ci.ub
intrcpt   -0.4157  0.3041  -1.3671  0.1716  -1.0117  0.1803
mods       1.3361  0.2905   4.5987  <.0001   0.7666  1.9055  ***

---
Signif. codes:  0 '***' 0.001 '**' 0.01 '*' 0.05 '.' 0.1 ' ' 1

[1] "Placebo"
[1] 2

Multivariate Meta-Analysis Model (k = 96; method: REML)
```

© 2021 Bentzley BS et al. *JAMA Network Open*.

```
Variance Components:

            estim    sqrt  nlvls  fixed          factor
sigma^2.1  0.8309  0.9115     51     no          Author
sigma^2.2  0.8295  0.9107     80     no  Author/StudyID

Test for Residual Heterogeneity:
QE(df = 94) = 315.0832, p-val < .0001

Test of Moderators (coefficient(s) 2):
QM(df = 1) = 17.5368, p-val < .0001

Model Results:

         estimate      se     zval    pval    ci.lb    ci.ub
intrcpt   -0.7618  0.3209  -2.3737  0.0176  -1.3908  -0.1328    *
mods       1.3485  0.3220   4.1877  <.0001   0.7174   1.9797  ***

---
Signif. codes:  0 '***' 0.001 '**' 0.01 '*' 0.05 '.' 0.1 ' ' 1

[1] "Placebo"
[1] 3

Multivariate Meta-Analysis Model (k = 96; method: REML)

Variance Components:

            estim    sqrt  nlvls  fixed          factor
sigma^2.1  1.2684  1.1262     51     no          Author
sigma^2.2  0.5300  0.7280     80     no  Author/StudyID

Test for Residual Heterogeneity:
QE(df = 94) = 343.3167, p-val < .0001

Test of Moderators (coefficient(s) 2):
QM(df = 1) = 12.0580, p-val = 0.0005

Model Results:

         estimate      se     zval    pval    ci.lb    ci.ub
intrcpt   -0.8671  0.3154  -2.7496  0.0060  -1.4852  -0.2490   **
mods       1.1144  0.3209   3.4725  0.0005   0.4854   1.7434  ***

---
Signif. codes:  0 '***' 0.001 '**' 0.01 '*' 0.05 '.' 0.1 ' ' 1

[1] "Placebo"
[1] 4

Multivariate Meta-Analysis Model (k = 96; method: REML)
```

© 2021 Bentzley BS et al. *JAMA Network Open*.

```
Variance Components:

            estim    sqrt  nlvls  fixed          factor
sigma^2.1  0.9780  0.9889     51     no          Author
sigma^2.2  0.3440  0.5865     80     no  Author/StudyID

Test for Residual Heterogeneity:
QE(df = 94) = 283.9711, p-val < .0001

Test of Moderators (coefficient(s) 2):
QM(df = 1) = 24.4018, p-val < .0001

Model Results:

         estimate      se     zval    pval    ci.lb   ci.ub
intrcpt   -0.5411  0.3143  -1.7214  0.0852  -1.1571  0.0750    .
mods       1.5006  0.3038   4.9398  <.0001   0.9052  2.0960  ***

---
Signif. codes:  0 '***' 0.001 '**' 0.01 '*' 0.05 '.' 0.1 ' ' 1

[1] "Placebo"
[1] 5

Multivariate Meta-Analysis Model (k = 96; method: REML)

Variance Components:

            estim    sqrt  nlvls  fixed          factor
sigma^2.1  0.7510  0.8666     51     no          Author
sigma^2.2  0.5964  0.7723     80     no  Author/StudyID

Test for Residual Heterogeneity:
QE(df = 94) = 280.9826, p-val < .0001

Test of Moderators (coefficient(s) 2):
QM(df = 1) = 20.2187, p-val < .0001

Model Results:

         estimate      se     zval    pval    ci.lb   ci.ub
intrcpt   -0.5832  0.3069  -1.9001  0.0574  -1.1847  0.0184    .
mods       1.3662  0.3038   4.4965  <.0001   0.7707  1.9618  ***

---
Signif. codes:  0 '***' 0.001 '**' 0.01 '*' 0.05 '.' 0.1 ' ' 1

[1] "Psychotherapy"
[1] 0

Multivariate Meta-Analysis Model (k = 259; method: REML)

Variance Components:
```

© 2021 Bentzley BS et al. *JAMA Network Open*.

```
            estim    sqrt  nlvls  fixed          factor
sigma^2.1  1.2076  1.0989     65     no          Author
sigma^2.2  0.5730  0.7570     98     no  Author/StudyID

Test for Residual Heterogeneity:
QE(df = 257) = 889.8962, p-val < .0001

Test of Moderators (coefficient(s) 2):
QM(df = 1) = 1.6025, p-val = 0.2055

Model Results:

         estimate      se    zval    pval    ci.lb   ci.ub
intrcpt    0.5313  0.2373  2.2389  0.0252   0.0662  0.9964  *
mods       0.2844  0.2246  1.2659  0.2055  -0.1559  0.7247

---
Signif. codes:  0 '***' 0.001 '**' 0.01 '*' 0.05 '.' 0.1 ' ' 1

[1] "Psychotherapy"
[1] 1

Multivariate Meta-Analysis Model (k = 330; method: REML)

Variance Components:

            estim    sqrt  nlvls  fixed          factor
sigma^2.1  1.1582  1.0762     82     no          Author
sigma^2.2  0.7329  0.8561    131     no  Author/StudyID

Test for Residual Heterogeneity:
QE(df = 328) = 1145.1182, p-val < .0001

Test of Moderators (coefficient(s) 2):
QM(df = 1) = 22.4099, p-val < .0001

Model Results:

         estimate      se    zval    pval    ci.lb   ci.ub
intrcpt    0.4171  0.2208  1.8886  0.0589  -0.0158  0.8499    .
mods       0.9017  0.1905  4.7339  <.0001   0.5284  1.2750  ***

---
Signif. codes:  0 '***' 0.001 '**' 0.01 '*' 0.05 '.' 0.1 ' ' 1

[1] "Psychotherapy"
[1] 2

Multivariate Meta-Analysis Model (k = 330; method: REML)

Variance Components:
```

```
            estim    sqrt  nlvls  fixed          factor
sigma^2.1  0.9096  0.9537     82     no          Author
sigma^2.2  0.9705  0.9851    131     no  Author/StudyID

Test for Residual Heterogeneity:
QE(df = 328) = 1389.6902, p-val < .0001

Test of Moderators (coefficient(s) 2):
QM(df = 1) = 18.5778, p-val < .0001

Model Results:

         estimate      se     zval    pval    ci.lb   ci.ub
intrcpt   -0.2354  0.2016  -1.1675  0.2430  -0.6306  0.1598
mods       0.8408  0.1951   4.3102  <.0001   0.4584  1.2231  ***

---
Signif. codes:  0 '***' 0.001 '**' 0.01 '*' 0.05 '.' 0.1 ' ' 1

[1] "Psychotherapy"
[1] 3

Multivariate Meta-Analysis Model (k = 330; method: REML)

Variance Components:

            estim    sqrt  nlvls  fixed          factor
sigma^2.1  1.1551  1.0748     82     no          Author
sigma^2.2  1.1943  1.0929    131     no  Author/StudyID

Test for Residual Heterogeneity:
QE(df = 328) = 1432.3839, p-val < .0001

Test of Moderators (coefficient(s) 2):
QM(df = 1) = 0.7444, p-val = 0.3883

Model Results:

         estimate      se    zval    pval    ci.lb   ci.ub
intrcpt    0.0599  0.2181  0.2748  0.7835  -0.3676  0.4874
mods       0.1821  0.2110  0.8628  0.3883  -0.2316  0.5957

---
Signif. codes:  0 '***' 0.001 '**' 0.01 '*' 0.05 '.' 0.1 ' ' 1

[1] "Psychotherapy"
[1] 4

Multivariate Meta-Analysis Model (k = 330; method: REML)

Variance Components:

            estim    sqrt  nlvls  fixed          factor
```

© 2021 Bentzley BS et al. *JAMA Network Open*.

```
sigma^2.1  1.2841  1.1332     82     no          Author
sigma^2.2  0.6489  0.8056    131     no  Author/StudyID

Test for Residual Heterogeneity:
QE(df = 328) = 1109.1718, p-val < .0001

Test of Moderators (coefficient(s) 2):
QM(df = 1) = 14.1316, p-val = 0.0002

Model Results:

         estimate      se    zval    pval   ci.lb   ci.ub
intrcpt    0.6045  0.2303  2.6252  0.0087  0.1532  1.0558   **
mods       0.7468  0.1987  3.7592  0.0002  0.3574  1.1361  ***

---
Signif. codes:  0 '***' 0.001 '**' 0.01 '*' 0.05 '.' 0.1 ' ' 1

[1] "Psychotherapy"
[1] 5

Multivariate Meta-Analysis Model (k = 330; method: REML)

Variance Components:

            estim    sqrt  nlvls  fixed          factor
sigma^2.1  0.6891  0.8301     82     no          Author
sigma^2.2  1.2129  1.1013    131     no  Author/StudyID

Test for Residual Heterogeneity:
QE(df = 328) = 1108.9843, p-val < .0001

Test of Moderators (coefficient(s) 2):
QM(df = 1) = 6.8123, p-val = 0.0091

Model Results:

         estimate      se    zval    pval    ci.lb   ci.ub
intrcpt    0.4230  0.2168  1.9508  0.0511  -0.0020  0.8480   .
mods       0.5309  0.2034  2.6100  0.0091   0.1322  0.9296  **

---
Signif. codes:  0 '***' 0.001 '**' 0.01 '*' 0.05 '.' 0.1 ' ' 1

[1] "ContingencyManagement"
[1] 0

Multivariate Meta-Analysis Model (k = 69; method: REML)

Variance Components:

            estim    sqrt  nlvls  fixed          factor
sigma^2.1  0.4707  0.6861     27     no          Author
```

© 2021 Bentzley BS et al. *JAMA Network Open*.

```
sigma^2.2  0.3542  0.5951     38     no  Author/StudyID

Test for Residual Heterogeneity:
QE(df = 67) = 207.4040, p-val < .0001

Test of Moderators (coefficient(s) 2):
QM(df = 1) = 8.0721, p-val = 0.0045

Model Results:

         estimate      se    zval    pval    ci.lb   ci.ub
intrcpt    0.5007  0.3091  1.6201  0.1052  -0.1051  1.1065
mods       1.1203  0.3943  2.8411  0.0045   0.3475  1.8932  **

---
Signif. codes:  0 '***' 0.001 '**' 0.01 '*' 0.05 '.' 0.1 ' ' 1

[1] "ContingencyManagement"
[1] 1

Multivariate Meta-Analysis Model (k = 88; method: REML)

Variance Components:

            estim    sqrt  nlvls  fixed          factor
sigma^2.1  0.1424  0.3773     33     no          Author
sigma^2.2  0.7093  0.8422     50     no  Author/StudyID

Test for Residual Heterogeneity:
QE(df = 86) = 256.0774, p-val < .0001

Test of Moderators (coefficient(s) 2):
QM(df = 1) = 29.9044, p-val < .0001

Model Results:

         estimate      se    zval    pval    ci.lb   ci.ub
intrcpt    0.2374  0.2748  0.8639  0.3877  -0.3012  0.7759
mods       1.7693  0.3236  5.4685  <.0001   1.1352  2.4035  ***

---
Signif. codes:  0 '***' 0.001 '**' 0.01 '*' 0.05 '.' 0.1 ' ' 1

[1] "ContingencyManagement"
[1] 2

Multivariate Meta-Analysis Model (k = 88; method: REML)

Variance Components:

            estim    sqrt  nlvls  fixed          factor
sigma^2.1  0.2943  0.5425     33     no          Author
sigma^2.2  0.7926  0.8903     50     no  Author/StudyID
```

© 2021 Bentzley BS et al. *JAMA Network Open*.

```
Test for Residual Heterogeneity:
QE(df = 86) = 360.1599, p-val < .0001

Test of Moderators (coefficient(s) 2):
QM(df = 1) = 14.4169, p-val = 0.0001

Model Results:

         estimate      se     zval    pval    ci.lb   ci.ub
intrcpt   -0.0172  0.2875  -0.0597  0.9524  -0.5807  0.5464
mods       1.4391  0.3790   3.7970  0.0001   0.6962  2.1819  ***

---
Signif. codes:  0 ‘***’ 0.001 ‘**’ 0.01 ‘*’ 0.05 ‘.’ 0.1 ‘ ’ 1

[1] "ContingencyManagement"
[1] 3

Multivariate Meta-Analysis Model (k = 88; method: REML)

Variance Components:

            estim    sqrt  nlvls  fixed          factor
sigma^2.1  0.3173  0.5633     33     no          Author
sigma^2.2  0.9476  0.9734     50     no  Author/StudyID

Test for Residual Heterogeneity:
QE(df = 86) = 374.3820, p-val < .0001

Test of Moderators (coefficient(s) 2):
QM(df = 1) = 10.8216, p-val = 0.0010

Model Results:

         estimate      se     zval    pval    ci.lb   ci.ub
intrcpt   -0.0411  0.2982  -0.1379  0.8903  -0.6256  0.5433
mods       1.3105  0.3984   3.2896  0.0010   0.5297  2.0913  **

---
Signif. codes:  0 ‘***’ 0.001 ‘**’ 0.01 ‘*’ 0.05 ‘.’ 0.1 ‘ ’ 1

[1] "ContingencyManagement"
[1] 4

Multivariate Meta-Analysis Model (k = 88; method: REML)

Variance Components:

            estim    sqrt  nlvls  fixed          factor
sigma^2.1  0.2403  0.4902     33     no          Author
sigma^2.2  0.4994  0.7067     50     no  Author/StudyID
```

© 2021 Bentzley BS et al. *JAMA Network Open*.

```
Test for Residual Heterogeneity:
QE(df = 86) = 231.2155, p-val < .0001

Test of Moderators (coefficient(s) 2):
QM(df = 1) = 32.2073, p-val < .0001

Model Results:

         estimate      se    zval    pval    ci.lb   ci.ub
intrcpt    0.2693  0.2769  0.9726  0.3307  -0.2734  0.8119
mods       1.7952  0.3163  5.6751  <.0001   1.1752  2.4152  ***

---
Signif. codes:  0 '***' 0.001 '**' 0.01 '*' 0.05 '.' 0.1 ' ' 1

[1] "ContingencyManagement"
[1] 5

Multivariate Meta-Analysis Model (k = 88; method: REML)

Variance Components:

            estim    sqrt  nlvls  fixed          factor
sigma^2.1  0.1260  0.3549     33     no          Author
sigma^2.2  0.6342  0.7964     50     no  Author/StudyID

Test for Residual Heterogeneity:
QE(df = 86) = 239.9328, p-val < .0001

Test of Moderators (coefficient(s) 2):
QM(df = 1) = 26.9386, p-val < .0001

Model Results:

         estimate      se    zval    pval    ci.lb   ci.ub
intrcpt    0.1012  0.2610  0.3879  0.6981  -0.4102  0.6127
mods       1.6962  0.3268  5.1902  <.0001   1.0557  2.3368  ***

---
Signif. codes:  0 '***' 0.001 '**' 0.01 '*' 0.05 '.' 0.1 ' ' 1

[1] "Other"
[1] 0

Multivariate Meta-Analysis Model (k = 21; method: REML)

Variance Components:

            estim    sqrt  nlvls  fixed          factor
sigma^2.1  0.0000  0.0001     11     no          Author
sigma^2.2  1.0571  1.0282     12     no  Author/StudyID

Test for Residual Heterogeneity:
```

© 2021 Bentzley BS et al. *JAMA Network Open*.

```
QE(df = 19) = 52.1176, p-val < .0001

Test of Moderators (coefficient(s) 2):
QM(df = 1) = 2.5727, p-val = 0.1087

Model Results:

         estimate      se    zval    pval    ci.lb   ci.ub
intrcpt    0.7616  0.5430  1.4026  0.1607  -0.3027  1.8259
mods       0.8248  0.5142  1.6040  0.1087  -0.1831  1.8326

---
Signif. codes:  0 '***' 0.001 '**' 0.01 '*' 0.05 '.' 0.1 ' ' 1

[1] "Other"
[1] 1

Multivariate Meta-Analysis Model (k = 26; method: REML)

Variance Components:

            estim    sqrt  nlvls  fixed          factor
sigma^2.1  0.0000  0.0001     14     no          Author
sigma^2.2  0.9366  0.9678     15     no  Author/StudyID

Test for Residual Heterogeneity:
QE(df = 24) = 56.2187, p-val = 0.0002

Test of Moderators (coefficient(s) 2):
QM(df = 1) = 4.1038, p-val = 0.0428

Model Results:

         estimate      se    zval    pval    ci.lb   ci.ub
intrcpt    0.8301  0.5165  1.6072  0.1080  -0.1822  1.8424
mods       0.9901  0.4888  2.0258  0.0428   0.0322  1.9480  *

---
Signif. codes:  0 '***' 0.001 '**' 0.01 '*' 0.05 '.' 0.1 ' ' 1

[1] "Other"
[1] 2

Multivariate Meta-Analysis Model (k = 26; method: REML)

Variance Components:

            estim    sqrt  nlvls  fixed          factor
sigma^2.1  0.0000  0.0003     14     no          Author
sigma^2.2  2.7994  1.6731     15     no  Author/StudyID

Test for Residual Heterogeneity:
QE(df = 24) = 130.9743, p-val < .0001
```

© 2021 Bentzley BS et al. *JAMA Network Open*.

```
Test of Moderators (coefficient(s) 2):
QM(df = 1) = 4.8216, p-val = 0.0281

Model Results:

         estimate      se     zval    pval    ci.lb   ci.ub
intrcpt   -0.2932  0.6478  -0.4525  0.6509  -1.5628  0.9765
mods       1.3142  0.5985   2.1958  0.0281   0.1412  2.4872  *

---
Signif. codes:  0 '***' 0.001 '**' 0.01 '*' 0.05 '.' 0.1 ' ' 1

[1] "Other"
[1] 3

Multivariate Meta-Analysis Model (k = 26; method: REML)

Variance Components:

            estim    sqrt  nlvls  fixed          factor
sigma^2.1  0.0000  0.0004     14     no          Author
sigma^2.2  2.8013  1.6737     15     no  Author/StudyID

Test for Residual Heterogeneity:
QE(df = 24) = 130.7994, p-val < .0001

Test of Moderators (coefficient(s) 2):
QM(df = 1) = 4.8297, p-val = 0.0280

Model Results:

         estimate      se     zval    pval    ci.lb   ci.ub
intrcpt   -0.2944  0.6479  -0.4544  0.6495  -1.5643  0.9755
mods       1.3154  0.5985   2.1977  0.0280   0.1423  2.4885  *

---
Signif. codes:  0 '***' 0.001 '**' 0.01 '*' 0.05 '.' 0.1 ' ' 1

[1] "Other"
[1] 4

Multivariate Meta-Analysis Model (k = 26; method: REML)

Variance Components:

            estim    sqrt  nlvls  fixed          factor
sigma^2.1  0.0000  0.0001     14     no          Author
sigma^2.2  0.9366  0.9678     15     no  Author/StudyID

Test for Residual Heterogeneity:
QE(df = 24) = 56.2187, p-val = 0.0002
```

© 2021 Bentzley BS et al. *JAMA Network Open*.

```
Test of Moderators (coefficient(s) 2):
QM(df = 1) = 4.1038, p-val = 0.0428

Model Results:

         estimate      se    zval    pval    ci.lb   ci.ub
intrcpt    0.8301  0.5165  1.6072  0.1080  -0.1822  1.8424
mods       0.9901  0.4888  2.0258  0.0428   0.0322  1.9480  *

---
Signif. codes:  0 ‘***’ 0.001 ‘**’ 0.01 ‘*’ 0.05 ‘.’ 0.1 ‘ ’ 1

[1] "Other"
[1] 5

Multivariate Meta-Analysis Model (k = 26; method: REML)

Variance Components:

            estim    sqrt  nlvls  fixed          factor
sigma^2.1  0.0000  0.0002     14     no          Author
sigma^2.2  2.2431  1.4977     15     no  Author/StudyID

Test for Residual Heterogeneity:
QE(df = 24) = 111.1455, p-val < .0001

Test of Moderators (coefficient(s) 2):
QM(df = 1) = 4.4686, p-val = 0.0345

Model Results:

         estimate      se    zval    pval    ci.lb   ci.ub
intrcpt    0.0831  0.6231  0.1333  0.8939  -1.1383  1.3044
mods       1.2124  0.5735  2.1139  0.0345   0.0883  2.3365  *

---
Signif. codes:  0 ‘***’ 0.001 ‘**’ 0.01 ‘*’ 0.05 ‘.’ 0.1 ‘ ’ 1

[1] "Opioid"
[1] 0

Multivariate Meta-Analysis Model (k = 142; method: REML)

Variance Components:

            estim    sqrt  nlvls  fixed          factor
sigma^2.1  0.5448  0.7381     32     no          Author
sigma^2.2  0.1551  0.3939     49     no  Author/StudyID

Test for Residual Heterogeneity:
QE(df = 140) = 257.9966, p-val < .0001

Test of Moderators (coefficient(s) 2):
```

© 2021 Bentzley BS et al. *JAMA Network Open*.

```
QM(df = 1) = 12.5663, p-val = 0.0004

Model Results:

         estimate      se    zval    pval    ci.lb   ci.ub
intrcpt    0.5269  0.2689  1.9599  0.0500  -0.0000  1.0539    .
mods       1.0012  0.2824  3.5449  0.0004   0.4476  1.5547  ***

---
Signif. codes:  0 '***' 0.001 '**' 0.01 '*' 0.05 '.' 0.1 ' ' 1

[1] "Opioid"
[1] 1

Multivariate Meta-Analysis Model (k = 159; method: REML)

Variance Components:

            estim    sqrt  nlvls  fixed          factor
sigma^2.1  0.6177  0.7859     34     no          Author
sigma^2.2  0.1197  0.3460     55     no  Author/StudyID

Test for Residual Heterogeneity:
QE(df = 157) = 283.7641, p-val < .0001

Test of Moderators (coefficient(s) 2):
QM(df = 1) = 16.2211, p-val < .0001

Model Results:

         estimate      se    zval    pval   ci.lb   ci.ub
intrcpt    0.5507  0.2626  2.0967  0.0360  0.0359  1.0655    *
mods       1.0537  0.2616  4.0275  <.0001  0.5409  1.5665  ***

---
Signif. codes:  0 '***' 0.001 '**' 0.01 '*' 0.05 '.' 0.1 ' ' 1

[1] "Opioid"
[1] 2

Multivariate Meta-Analysis Model (k = 159; method: REML)

Variance Components:

            estim    sqrt  nlvls  fixed          factor
sigma^2.1  1.4455  1.2023     34     no          Author
sigma^2.2  0.3765  0.6136     55     no  Author/StudyID

Test for Residual Heterogeneity:
QE(df = 157) = 529.1676, p-val < .0001

Test of Moderators (coefficient(s) 2):
QM(df = 1) = 4.4702, p-val = 0.0345
```

© 2021 Bentzley BS et al. *JAMA Network Open*.

```
Model Results:

         estimate      se    zval    pval    ci.lb   ci.ub
intrcpt    0.4851  0.3507  1.3833  0.1666  -0.2022  1.1724
mods       0.7043  0.3331  2.1143  0.0345   0.0514  1.3572  *

---
Signif. codes:  0 '***' 0.001 '**' 0.01 '*' 0.05 '.' 0.1 ' ' 1

[1] "Opioid"
[1] 3

Multivariate Meta-Analysis Model (k = 159; method: REML)

Variance Components:

            estim    sqrt  nlvls  fixed          factor
sigma^2.1  1.4872  1.2195     34     no          Author
sigma^2.2  0.3012  0.5488     55     no  Author/StudyID

Test for Residual Heterogeneity:
QE(df = 157) = 523.1105, p-val < .0001

Test of Moderators (coefficient(s) 2):
QM(df = 1) = 4.5844, p-val = 0.0323

Model Results:

         estimate      se    zval    pval    ci.lb   ci.ub
intrcpt    0.5070  0.3427  1.4792  0.1391  -0.1648  1.1787
mods       0.6859  0.3203  2.1411  0.0323   0.0580  1.3137  *

---
Signif. codes:  0 '***' 0.001 '**' 0.01 '*' 0.05 '.' 0.1 ' ' 1

[1] "Opioid"
[1] 4

Multivariate Meta-Analysis Model (k = 159; method: REML)

Variance Components:

            estim    sqrt  nlvls  fixed          factor
sigma^2.1  0.6361  0.7976     34     no          Author
sigma^2.2  0.1204  0.3470     55     no  Author/StudyID

Test for Residual Heterogeneity:
QE(df = 157) = 281.1605, p-val < .0001

Test of Moderators (coefficient(s) 2):
QM(df = 1) = 15.2348, p-val < .0001
```

© 2021 Bentzley BS et al. *JAMA Network Open*.

```
Model Results:

         estimate      se    zval    pval   ci.lb   ci.ub
intrcpt    0.5584  0.2711  2.0602  0.0394  0.0272  1.0897    *
mods       1.0515  0.2694  3.9032  <.0001  0.5235  1.5796  ***

---
Signif. codes:  0 '***' 0.001 '**' 0.01 '*' 0.05 '.' 0.1 ' ' 1

[1] "Opioid"
[1] 5

Multivariate Meta-Analysis Model (k = 159; method: REML)

Variance Components:

            estim    sqrt  nlvls  fixed          factor
sigma^2.1  0.5072  0.7122     34     no          Author
sigma^2.2  0.7522  0.8673     55     no  Author/StudyID

Test for Residual Heterogeneity:
QE(df = 157) = 375.3041, p-val < .0001

Test of Moderators (coefficient(s) 2):
QM(df = 1) = 3.7491, p-val = 0.0528

Model Results:

         estimate      se    zval    pval    ci.lb   ci.ub
intrcpt    0.7365  0.3189  2.3099  0.0209   0.1116  1.3615  *
mods       0.6184  0.3194  1.9363  0.0528  -0.0076  1.2444  .

---
Signif. codes:  0 '***' 0.001 '**' 0.01 '*' 0.05 '.' 0.1 ' ' 1

[1] "MiscMeds"
[1] 0

Multivariate Meta-Analysis Model (k = 38; method: REML)

Variance Components:

            estim    sqrt  nlvls  fixed          factor
sigma^2.1  0.8811  0.9387     21     no          Author
sigma^2.2  0.1883  0.4339     26     no  Author/StudyID

Test for Residual Heterogeneity:
QE(df = 36) = 80.7060, p-val < .0001

Test of Moderators (coefficient(s) 2):
QM(df = 1) = 2.1600, p-val = 0.1416

Model Results:
```

```
         estimate      se    zval    pval    ci.lb   ci.ub
intrcpt    0.0951  0.4744  0.2004  0.8412  -0.8347  1.0249
mods       0.6615  0.4501  1.4697  0.1416  -0.2207  1.5437

---
Signif. codes:  0 '***' 0.001 '**' 0.01 '*' 0.05 '.' 0.1 ' ' 1

[1] "MiscMeds"
[1] 1

Multivariate Meta-Analysis Model (k = 54; method: REML)

Variance Components:

            estim    sqrt  nlvls  fixed          factor
sigma^2.1  1.0832  1.0408     27     no          Author
sigma^2.2  0.0000  0.0001     37     no  Author/StudyID

Test for Residual Heterogeneity:
QE(df = 52) = 105.2566, p-val < .0001

Test of Moderators (coefficient(s) 2):
QM(df = 1) = 5.2179, p-val = 0.0224

Model Results:

         estimate      se    zval    pval    ci.lb   ci.ub
intrcpt    0.2081  0.4311  0.4828  0.6293  -0.6368  1.0530
mods       0.8411  0.3682  2.2843  0.0224   0.1194  1.5627  *

---
Signif. codes:  0 '***' 0.001 '**' 0.01 '*' 0.05 '.' 0.1 ' ' 1

[1] "MiscMeds"
[1] 2

Multivariate Meta-Analysis Model (k = 54; method: REML)

Variance Components:

            estim    sqrt  nlvls  fixed          factor
sigma^2.1  1.1464  1.0707     27     no          Author
sigma^2.2  1.4405  1.2002     37     no  Author/StudyID

Test for Residual Heterogeneity:
QE(df = 52) = 217.4944, p-val < .0001

Test of Moderators (coefficient(s) 2):
QM(df = 1) = 1.6396, p-val = 0.2004

Model Results:
```

© 2021 Bentzley BS et al. *JAMA Network Open*.

```
         estimate      se     zval    pval    ci.lb   ci.ub
intrcpt   -0.2861  0.5820  -0.4915  0.6230  -1.4268  0.8546
mods       0.6710  0.5241   1.2805  0.2004  -0.3561  1.6982

---
Signif. codes:  0 '***' 0.001 '**' 0.01 '*' 0.05 '.' 0.1 ' ' 1

[1] "MiscMeds"
[1] 3

Multivariate Meta-Analysis Model (k = 54; method: REML)

Variance Components:

            estim    sqrt  nlvls  fixed          factor
sigma^2.1  0.7540  0.8683     27     no          Author
sigma^2.2  1.3655  1.1685     37     no  Author/StudyID

Test for Residual Heterogeneity:
QE(df = 52) = 198.3370, p-val < .0001

Test of Moderators (coefficient(s) 2):
QM(df = 1) = 1.2338, p-val = 0.2667

Model Results:

         estimate      se     zval    pval    ci.lb   ci.ub
intrcpt   -0.4512  0.5282  -0.8543  0.3929  -1.4864  0.5840
mods       0.5621  0.5060   1.1108  0.2667  -0.4297  1.5539

---
Signif. codes:  0 '***' 0.001 '**' 0.01 '*' 0.05 '.' 0.1 ' ' 1

[1] "MiscMeds"
[1] 4

Multivariate Meta-Analysis Model (k = 54; method: REML)

Variance Components:

            estim    sqrt  nlvls  fixed          factor
sigma^2.1  1.1857  1.0889     27     no          Author
sigma^2.2  0.0000  0.0001     37     no  Author/StudyID

Test for Residual Heterogeneity:
QE(df = 52) = 106.1010, p-val < .0001

Test of Moderators (coefficient(s) 2):
QM(df = 1) = 3.7190, p-val = 0.0538

Model Results:

         estimate      se    zval    pval    ci.lb   ci.ub
```

© 2021 Bentzley BS et al. *JAMA Network Open*.

```
intrcpt    0.3099  0.4626  0.6699  0.5029  -0.5968  1.2167
mods       0.7585  0.3933  1.9285  0.0538  -0.0124  1.5294  .

---
Signif. codes:  0 '***' 0.001 '**' 0.01 '*' 0.05 '.' 0.1 ' ' 1

[1] "MiscMeds"
[1] 5

Multivariate Meta-Analysis Model (k = 54; method: REML)

Variance Components:

            estim    sqrt  nlvls  fixed          factor
sigma^2.1  0.0000  0.0001     27     no          Author
sigma^2.2  1.1331  1.0645     37     no  Author/StudyID

Test for Residual Heterogeneity:
QE(df = 52) = 123.3111, p-val < .0001

Test of Moderators (coefficient(s) 2):
QM(df = 1) = 4.7770, p-val = 0.0288

Model Results:

         estimate      se     zval    pval    ci.lb   ci.ub
intrcpt   -0.1702  0.4545  -0.3744  0.7081  -1.0609  0.7206
mods       0.9198  0.4208   2.1856  0.0288   0.0950  1.7446  *

---
Signif. codes:  0 '***' 0.001 '**' 0.01 '*' 0.05 '.' 0.1 ' ' 1

[1] "Psychostimulants"
[1] 0

Multivariate Meta-Analysis Model (k = 19; method: REML)

Variance Components:

            estim    sqrt  nlvls  fixed          factor
sigma^2.1  0.4836  0.6954      8     no          Author
sigma^2.2  0.5910  0.7688     13     no  Author/StudyID

Test for Residual Heterogeneity:
QE(df = 17) = 46.3431, p-val = 0.0002

Test of Moderators (coefficient(s) 2):
QM(df = 1) = 3.0608, p-val = 0.0802

Model Results:

         estimate      se     zval    pval    ci.lb   ci.ub
intrcpt   -0.4426  0.6702  -0.6604  0.5090  -1.7561  0.8709
```

© 2021 Bentzley BS et al. *JAMA Network Open*.

```
mods       1.3591  0.7768   1.7495  0.0802  -0.1635  2.8817  .

---
Signif. codes:  0 ‘***’ 0.001 ‘**’ 0.01 ‘*’ 0.05 ‘.’ 0.1 ‘ ’ 1

[1] "Psychostimulants"
[1] 1

Multivariate Meta-Analysis Model (k = 24; method: REML)

Variance Components:

            estim    sqrt  nlvls  fixed          factor
sigma^2.1  0.3924  0.6264     10     no          Author
sigma^2.2  0.4654  0.6822     17     no  Author/StudyID

Test for Residual Heterogeneity:
QE(df = 22) = 49.2371, p-val = 0.0007

Test of Moderators (coefficient(s) 2):
QM(df = 1) = 5.9121, p-val = 0.0150

Model Results:

         estimate      se     zval    pval    ci.lb   ci.ub
intrcpt   -0.4893  0.5752  -0.8506  0.3950  -1.6168  0.6382
mods       1.3659  0.5617   2.4315  0.0150   0.2649  2.4669  *

---
Signif. codes:  0 ‘***’ 0.001 ‘**’ 0.01 ‘*’ 0.05 ‘.’ 0.1 ‘ ’ 1

[1] "Psychostimulants"
[1] 2

Multivariate Meta-Analysis Model (k = 24; method: REML)

Variance Components:

            estim    sqrt  nlvls  fixed          factor
sigma^2.1  1.9887  1.4102     10     no          Author
sigma^2.2  0.8559  0.9252     17     no  Author/StudyID

Test for Residual Heterogeneity:
QE(df = 22) = 82.9868, p-val < .0001

Test of Moderators (coefficient(s) 2):
QM(df = 1) = 0.0195, p-val = 0.8890

Model Results:

         estimate      se     zval    pval    ci.lb   ci.ub
intrcpt   -0.0579  0.9239  -0.0627  0.9500  -1.8686  1.7528
mods       0.1183  0.8475   0.1396  0.8890  -1.5427  1.7793
```

© 2021 Bentzley BS et al. *JAMA Network Open*.

```
---
Signif. codes:  0 '***' 0.001 '**' 0.01 '*' 0.05 '.' 0.1 ' ' 1

[1] "Psychostimulants"
[1] 3

Multivariate Meta-Analysis Model (k = 24; method: REML)

Variance Components:

            estim    sqrt  nlvls  fixed          factor
sigma^2.1  1.9826  1.4081     10     no          Author
sigma^2.2  0.9826  0.9913     17     no  Author/StudyID

Test for Residual Heterogeneity:
QE(df = 22) = 84.3098, p-val < .0001

Test of Moderators (coefficient(s) 2):
QM(df = 1) = 0.0528, p-val = 0.8183

Model Results:

         estimate      se     zval    pval    ci.lb   ci.ub
intrcpt    0.1815  0.9131   0.1987  0.8425  -1.6081  1.9710
mods      -0.1870  0.8141  -0.2297  0.8183  -1.7826  1.4086

---
Signif. codes:  0 '***' 0.001 '**' 0.01 '*' 0.05 '.' 0.1 ' ' 1

[1] "Psychostimulants"
[1] 4

Multivariate Meta-Analysis Model (k = 24; method: REML)

Variance Components:

            estim    sqrt  nlvls  fixed          factor
sigma^2.1  0.3924  0.6264     10     no          Author
sigma^2.2  0.4654  0.6822     17     no  Author/StudyID

Test for Residual Heterogeneity:
QE(df = 22) = 49.2371, p-val = 0.0007

Test of Moderators (coefficient(s) 2):
QM(df = 1) = 5.9121, p-val = 0.0150

Model Results:

         estimate      se     zval    pval    ci.lb   ci.ub
intrcpt   -0.4893  0.5752  -0.8506  0.3950  -1.6168  0.6382
mods       1.3659  0.5617   2.4315  0.0150   0.2649  2.4669  *
```

© 2021 Bentzley BS et al. *JAMA Network Open*.

```
---
Signif. codes:  0 '***' 0.001 '**' 0.01 '*' 0.05 '.' 0.1 ' ' 1

[1] "Psychostimulants"
[1] 5

Multivariate Meta-Analysis Model (k = 24; method: REML)

Variance Components:

            estim    sqrt  nlvls  fixed          factor
sigma^2.1  0.4382  0.6620     10     no          Author
sigma^2.2  2.0622  1.4360     17     no  Author/StudyID

Test for Residual Heterogeneity:
QE(df = 22) = 80.8123, p-val < .0001

Test of Moderators (coefficient(s) 2):
QM(df = 1) = 0.0752, p-val = 0.7839

Model Results:

         estimate      se    zval    pval    ci.lb   ci.ub
intrcpt    0.0604  0.8123  0.0743  0.9407  -1.5317  1.6524
mods       0.2115  0.7711  0.2742  0.7839  -1.2998  1.7228

---
Signif. codes:  0 '***' 0.001 '**' 0.01 '*' 0.05 '.' 0.1 ' ' 1

[1] "Anticonvulsants"
[1] 0

Multivariate Meta-Analysis Model (k = 17; method: REML)

Variance Components:

            estim    sqrt  nlvls  fixed          factor
sigma^2.1  0.3704  0.6086     12     no          Author
sigma^2.2  0.0000  0.0001     14     no  Author/StudyID

Test for Residual Heterogeneity:
QE(df = 15) = 29.5457, p-val = 0.0137

Test of Moderators (coefficient(s) 2):
QM(df = 1) = 15.7017, p-val < .0001

Model Results:

         estimate      se     zval    pval    ci.lb    ci.ub
intrcpt   -1.3255  0.5299  -2.5012  0.0124  -2.3641  -0.2868    *
mods       2.7236  0.6873   3.9625  <.0001   1.3764   4.0707  ***

---
```

© 2021 Bentzley BS et al. *JAMA Network Open*.

```
Signif. codes:  0 '***' 0.001 '**' 0.01 '*' 0.05 '.' 0.1 ' ' 1

[1] "Anticonvulsants"
[1] 1

Multivariate Meta-Analysis Model (k = 20; method: REML)

Variance Components:

            estim    sqrt  nlvls  fixed          factor
sigma^2.1  0.3970  0.6301     14     no          Author
sigma^2.2  0.0000  0.0001     16     no  Author/StudyID

Test for Residual Heterogeneity:
QE(df = 18) = 32.9050, p-val = 0.0171

Test of Moderators (coefficient(s) 2):
QM(df = 1) = 21.3924, p-val < .0001

Model Results:

         estimate      se     zval    pval    ci.lb    ci.ub
intrcpt   -1.3947  0.5320  -2.6215  0.0088  -2.4375  -0.3519   **
mods       2.9675  0.6416   4.6252  <.0001   1.7100   4.2250  ***

---
Signif. codes:  0 '***' 0.001 '**' 0.01 '*' 0.05 '.' 0.1 ' ' 1

[1] "Anticonvulsants"
[1] 2

Multivariate Meta-Analysis Model (k = 20; method: REML)

Variance Components:

            estim    sqrt  nlvls  fixed          factor
sigma^2.1  0.9235  0.9610     14     no          Author
sigma^2.2  0.0000  0.0001     16     no  Author/StudyID

Test for Residual Heterogeneity:
QE(df = 18) = 47.2131, p-val = 0.0002

Test of Moderators (coefficient(s) 2):
QM(df = 1) = 14.9264, p-val = 0.0001

Model Results:

         estimate      se     zval    pval    ci.lb    ci.ub
intrcpt   -1.8601  0.6100  -3.0491  0.0023  -3.0557  -0.6644   **
mods       3.0847  0.7984   3.8635  0.0001   1.5198   4.6496  ***

---
Signif. codes:  0 '***' 0.001 '**' 0.01 '*' 0.05 '.' 0.1 ' ' 1
```

© 2021 Bentzley BS et al. *JAMA Network Open*.

```
[1] "Anticonvulsants"
[1] 3

Multivariate Meta-Analysis Model (k = 20; method: REML)

Variance Components:

            estim    sqrt  nlvls  fixed          factor
sigma^2.1  0.9034  0.9505     14     no          Author
sigma^2.2  0.0000  0.0001     16     no  Author/StudyID

Test for Residual Heterogeneity:
QE(df = 18) = 46.2202, p-val = 0.0003

Test of Moderators (coefficient(s) 2):
QM(df = 1) = 14.8811, p-val = 0.0001

Model Results:

         estimate      se     zval    pval    ci.lb    ci.ub
intrcpt   -1.8256  0.6035  -3.0248  0.0025  -3.0085  -0.6427   **
mods       3.0539  0.7917   3.8576  0.0001   1.5023   4.6055  ***

---
Signif. codes:  0 '***' 0.001 '**' 0.01 '*' 0.05 '.' 0.1 ' ' 1

[1] "Anticonvulsants"
[1] 4

Multivariate Meta-Analysis Model (k = 20; method: REML)

Variance Components:

            estim    sqrt  nlvls  fixed          factor
sigma^2.1  0.3970  0.6301     14     no          Author
sigma^2.2  0.0000  0.0001     16     no  Author/StudyID

Test for Residual Heterogeneity:
QE(df = 18) = 32.9050, p-val = 0.0171

Test of Moderators (coefficient(s) 2):
QM(df = 1) = 21.3924, p-val < .0001

Model Results:

         estimate      se     zval    pval    ci.lb    ci.ub
intrcpt   -1.3947  0.5320  -2.6215  0.0088  -2.4375  -0.3519   **
mods       2.9675  0.6416   4.6252  <.0001   1.7100   4.2250  ***

---
Signif. codes:  0 '***' 0.001 '**' 0.01 '*' 0.05 '.' 0.1 ' ' 1
```

© 2021 Bentzley BS et al. *JAMA Network Open*.

```
[1] "Anticonvulsants"
[1] 5

Multivariate Meta-Analysis Model (k = 20; method: REML)

Variance Components:

            estim    sqrt  nlvls  fixed          factor 
sigma^2.1  0.3970  0.6301     14     no          Author 
sigma^2.2  0.0000  0.0001     16     no  Author/StudyID 

Test for Residual Heterogeneity:
QE(df = 18) = 32.9050, p-val = 0.0171

Test of Moderators (coefficient(s) 2):
QM(df = 1) = 21.3924, p-val < .0001

Model Results:

         estimate      se     zval    pval    ci.lb    ci.ub      
intrcpt   -1.3947  0.5320  -2.6215  0.0088  -2.4375  -0.3519   ** 
mods       2.9675  0.6416   4.6252  <.0001   1.7100   4.2250  *** 

---
Signif. codes:  0 '***' 0.001 '**' 0.01 '*' 0.05 '.' 0.1 ' ' 1

[1] "DopamineAgonists"
[1] 0

Multivariate Meta-Analysis Model (k = 18; method: REML)

Variance Components:

            estim    sqrt  nlvls  fixed          factor 
sigma^2.1  0.0000  0.0001      9     no          Author 
sigma^2.2  0.2556  0.5056     10     no  Author/StudyID 

Test for Residual Heterogeneity:
QE(df = 16) = 22.3523, p-val = 0.1322

Test of Moderators (coefficient(s) 2):
QM(df = 1) = 4.2579, p-val = 0.0391

Model Results:

         estimate      se     zval    pval    ci.lb    ci.ub    
intrcpt   -0.9012  0.3685  -2.4452  0.0145  -1.6235  -0.1788  * 
mods       1.1913  0.5773   2.0635  0.0391   0.0598   2.3228  * 

---
Signif. codes:  0 '***' 0.001 '**' 0.01 '*' 0.05 '.' 0.1 ' ' 1

[1] "DopamineAgonists"
```

© 2021 Bentzley BS et al. *JAMA Network Open*.

```
[1] 1

Multivariate Meta-Analysis Model (k = 20; method: REML)

Variance Components:

            estim    sqrt  nlvls  fixed          factor
sigma^2.1  0.2785  0.5278     10     no          Author
sigma^2.2  0.0000  0.0001     12     no  Author/StudyID

Test for Residual Heterogeneity:
QE(df = 18) = 26.2047, p-val = 0.0952

Test of Moderators (coefficient(s) 2):
QM(df = 1) = 10.7469, p-val = 0.0010

Model Results:

         estimate      se     zval    pval    ci.lb    ci.ub
intrcpt   -1.0335  0.3528  -2.9297  0.0034  -1.7249  -0.3421  **
mods       1.6644  0.5077   3.2783  0.0010   0.6693   2.6595  **

---
Signif. codes:  0 '***' 0.001 '**' 0.01 '*' 0.05 '.' 0.1 ' ' 1

[1] "DopamineAgonists"
[1] 2

Multivariate Meta-Analysis Model (k = 20; method: REML)

Variance Components:

            estim    sqrt  nlvls  fixed          factor
sigma^2.1  0.0000  0.0000     10     no          Author
sigma^2.2  0.8971  0.9471     12     no  Author/StudyID

Test for Residual Heterogeneity:
QE(df = 18) = 40.0693, p-val = 0.0020

Test of Moderators (coefficient(s) 2):
QM(df = 1) = 1.9548, p-val = 0.1621

Model Results:

         estimate      se     zval    pval    ci.lb    ci.ub
intrcpt   -1.0620  0.4838  -2.1951  0.0282  -2.0102  -0.1138  *
mods       0.9904  0.7084   1.3981  0.1621  -0.3980   2.3788

---
Signif. codes:  0 '***' 0.001 '**' 0.01 '*' 0.05 '.' 0.1 ' ' 1

[1] "DopamineAgonists"
[1] 3
```

© 2021 Bentzley BS et al. *JAMA Network Open*.

```
Multivariate Meta-Analysis Model (k = 20; method: REML)

Variance Components:

            estim    sqrt  nlvls  fixed          factor
sigma^2.1  0.0000  0.0000     10     no          Author
sigma^2.2  0.9545  0.9770     12     no  Author/StudyID

Test for Residual Heterogeneity:
QE(df = 18) = 41.2222, p-val = 0.0014

Test of Moderators (coefficient(s) 2):
QM(df = 1) = 1.8024, p-val = 0.1794

Model Results:

         estimate      se     zval    pval    ci.lb    ci.ub
intrcpt   -1.0616  0.4944  -2.1471  0.0318  -2.0307  -0.0925  *
mods       0.9642  0.7182   1.3425  0.1794  -0.4434   2.3719

---
Signif. codes:  0 '***' 0.001 '**' 0.01 '*' 0.05 '.' 0.1 ' ' 1

[1] "DopamineAgonists"
[1] 4

Multivariate Meta-Analysis Model (k = 20; method: REML)

Variance Components:

            estim    sqrt  nlvls  fixed          factor
sigma^2.1  0.2785  0.5278     10     no          Author
sigma^2.2  0.0000  0.0001     12     no  Author/StudyID

Test for Residual Heterogeneity:
QE(df = 18) = 26.2047, p-val = 0.0952

Test of Moderators (coefficient(s) 2):
QM(df = 1) = 10.7469, p-val = 0.0010

Model Results:

         estimate      se     zval    pval    ci.lb    ci.ub
intrcpt   -1.0335  0.3528  -2.9297  0.0034  -1.7249  -0.3421  **
mods       1.6644  0.5077   3.2783  0.0010   0.6693   2.6595  **

---
Signif. codes:  0 '***' 0.001 '**' 0.01 '*' 0.05 '.' 0.1 ' ' 1

[1] "DopamineAgonists"
[1] 5
```

© 2021 Bentzley BS et al. *JAMA Network Open*.

```
Multivariate Meta-Analysis Model (k = 20; method: REML)

Variance Components:

            estim    sqrt  nlvls  fixed          factor
sigma^2.1  0.2785  0.5278     10     no          Author
sigma^2.2  0.0000  0.0001     12     no  Author/StudyID

Test for Residual Heterogeneity:
QE(df = 18) = 26.2047, p-val = 0.0952

Test of Moderators (coefficient(s) 2):
QM(df = 1) = 10.7469, p-val = 0.0010

Model Results:

         estimate      se     zval    pval    ci.lb    ci.ub
intrcpt   -1.0335  0.3528  -2.9297  0.0034  -1.7249  -0.3421  **
mods       1.6644  0.5077   3.2783  0.0010   0.6693   2.6595  **

---
Signif. codes:  0 '***' 0.001 '**' 0.01 '*' 0.05 '.' 0.1 ' ' 1

[1] "Antidepressants"
[1] 0

Multivariate Meta-Analysis Model (k = 15; method: REML)

Variance Components:

            estim    sqrt  nlvls  fixed          factor
sigma^2.1  0.0000  0.0001      8     no          Author
sigma^2.2  0.0000  0.0001      8     no  Author/StudyID

Test for Residual Heterogeneity:
QE(df = 13) = 14.8644, p-val = 0.3159

Test of Moderators (coefficient(s) 2):
QM(df = 1) = 39.2060, p-val < .0001

Model Results:

         estimate      se     zval    pval    ci.lb    ci.ub
intrcpt   -1.7957  0.5371  -3.3433  0.0008  -2.8483  -0.7430  ***
mods       3.3421  0.5338   6.2615  <.0001   2.2960   4.3882  ***

---
Signif. codes:  0 '***' 0.001 '**' 0.01 '*' 0.05 '.' 0.1 ' ' 1

[1] "Antidepressants"
[1] 1

Multivariate Meta-Analysis Model (k = 18; method: REML)
```

© 2021 Bentzley BS et al. *JAMA Network Open*.

```
Variance Components:

            estim    sqrt  nlvls  fixed          factor
sigma^2.1  0.0088  0.0939     11     no          Author
sigma^2.2  0.0088  0.0939     11     no  Author/StudyID

Test for Residual Heterogeneity:
QE(df = 16) = 16.4389, p-val = 0.4228

Test of Moderators (coefficient(s) 2):
QM(df = 1) = 44.6331, p-val < .0001

Model Results:

         estimate      se     zval    pval    ci.lb    ci.ub
intrcpt   -1.4394  0.4231  -3.4018  0.0007  -2.2687  -0.6101  ***
mods       2.9627  0.4435   6.6808  <.0001   2.0935   3.8319  ***

---
Signif. codes:  0 '***' 0.001 '**' 0.01 '*' 0.05 '.' 0.1 ' ' 1

[1] "Antidepressants"
[1] 2

Multivariate Meta-Analysis Model (k = 18; method: REML)

Variance Components:

            estim    sqrt  nlvls  fixed          factor
sigma^2.1  0.2311  0.4808     11     no          Author
sigma^2.2  0.2311  0.4808     11     no  Author/StudyID

Test for Residual Heterogeneity:
QE(df = 16) = 24.3265, p-val = 0.0826

Test of Moderators (coefficient(s) 2):
QM(df = 1) = 21.7454, p-val < .0001

Model Results:

         estimate      se     zval    pval    ci.lb    ci.ub
intrcpt   -2.1752  0.7425  -2.9296  0.0034  -3.6305  -0.7199   **
mods       3.4345  0.7365   4.6632  <.0001   1.9910   4.8781  ***

---
Signif. codes:  0 '***' 0.001 '**' 0.01 '*' 0.05 '.' 0.1 ' ' 1

[1] "Antidepressants"
[1] 3

Multivariate Meta-Analysis Model (k = 18; method: REML)
```

© 2021 Bentzley BS et al. *JAMA Network Open*.

```
Variance Components:

            estim    sqrt  nlvls  fixed          factor
sigma^2.1  0.5339  0.7307     11     no          Author
sigma^2.2  0.5339  0.7307     11     no  Author/StudyID

Test for Residual Heterogeneity:
QE(df = 16) = 32.9936, p-val = 0.0074

Test of Moderators (coefficient(s) 2):
QM(df = 1) = 17.3226, p-val < .0001

Model Results:

         estimate      se     zval    pval    ci.lb    ci.ub
intrcpt   -2.4412  0.7974  -3.0615  0.0022  -4.0041  -0.8784   **
mods       3.4176  0.8211   4.1620  <.0001   1.8082   5.0270  ***

---
Signif. codes:  0 '***' 0.001 '**' 0.01 '*' 0.05 '.' 0.1 ' ' 1

[1] "Antidepressants"
[1] 4

Multivariate Meta-Analysis Model (k = 18; method: REML)

Variance Components:

            estim    sqrt  nlvls  fixed          factor
sigma^2.1  0.0000  0.0000     11     no          Author
sigma^2.2  0.0000  0.0000     11     no  Author/StudyID

Test for Residual Heterogeneity:
QE(df = 16) = 15.8653, p-val = 0.4624

Test of Moderators (coefficient(s) 2):
QM(df = 1) = 39.8996, p-val < .0001

Model Results:

         estimate      se     zval    pval    ci.lb    ci.ub
intrcpt   -1.7312  0.5327  -3.2497  0.0012  -2.7753  -0.6871   **
mods       3.2204  0.5098   6.3166  <.0001   2.2212   4.2197  ***

---
Signif. codes:  0 '***' 0.001 '**' 0.01 '*' 0.05 '.' 0.1 ' ' 1

[1] "Antidepressants"
[1] 5

Multivariate Meta-Analysis Model (k = 18; method: REML)

Variance Components:
```

© 2021 Bentzley BS et al. *JAMA Network Open*.

```
            estim    sqrt  nlvls  fixed          factor
sigma^2.1  0.0000  0.0000     11     no          Author
sigma^2.2  0.0000  0.0000     11     no  Author/StudyID

Test for Residual Heterogeneity:
QE(df = 16) = 15.8653, p-val = 0.4624

Test of Moderators (coefficient(s) 2):
QM(df = 1) = 39.8996, p-val < .0001

Model Results:

         estimate      se     zval    pval    ci.lb    ci.ub
intrcpt   -1.7312  0.5327  -3.2497  0.0012  -2.7753  -0.6871   **
mods       3.2204  0.5098   6.3166  <.0001   2.2212   4.2197  ***

---
Signif. codes:  0 ‘***’ 0.001 ‘**’ 0.01 ‘*’ 0.05 ‘.’ 0.1 ‘ ’ 1

[1] "Antipsychotics"
[1] 0

Multivariate Meta-Analysis Model (k = 11; method: REML)

Variance Components:

            estim    sqrt  nlvls  fixed          factor
sigma^2.1  0.6061  0.7785      7     no          Author
sigma^2.2  0.0000  0.0000      8     no  Author/StudyID

Test for Residual Heterogeneity:
QE(df = 9) = 15.9240, p-val = 0.0685

Test of Moderators (coefficient(s) 2):
QM(df = 1) = 0.0759, p-val = 0.7830

Model Results:

         estimate      se     zval    pval    ci.lb   ci.ub
intrcpt   -0.4668  0.7468  -0.6250  0.5320  -1.9305  0.9970
mods      -0.2106  0.7644  -0.2755  0.7830  -1.7087  1.2876

---
Signif. codes:  0 ‘***’ 0.001 ‘**’ 0.01 ‘*’ 0.05 ‘.’ 0.1 ‘ ’ 1

[1] "Antipsychotics"
[1] 1

Multivariate Meta-Analysis Model (k = 12; method: REML)

Variance Components:
```

© 2021 Bentzley BS et al. *JAMA Network Open*.

```
            estim    sqrt  nlvls  fixed          factor
sigma^2.1  2.5846  1.6077      8     no          Author
sigma^2.2  0.0000  0.0000      9     no  Author/StudyID

Test for Residual Heterogeneity:
QE(df = 10) = 32.3451, p-val = 0.0004

Test of Moderators (coefficient(s) 2):
QM(df = 1) = 0.2031, p-val = 0.6522

Model Results:

         estimate      se     zval    pval    ci.lb   ci.ub
intrcpt    0.3107  1.0188   0.3049  0.7604  -1.6861  2.3075
mods      -0.3920  0.8699  -0.4507  0.6522  -2.0969  1.3129

---
Signif. codes:  0 ‘***’ 0.001 ‘**’ 0.01 ‘*’ 0.05 ‘.’ 0.1 ‘ ’ 1

[1] "Antipsychotics"
[1] 2

Multivariate Meta-Analysis Model (k = 12; method: REML)

Variance Components:

            estim    sqrt  nlvls  fixed          factor
sigma^2.1  0.3001  0.5478      8     no          Author
sigma^2.2  0.0000  0.0000      9     no  Author/StudyID

Test for Residual Heterogeneity:
QE(df = 10) = 15.9437, p-val = 0.1013

Test of Moderators (coefficient(s) 2):
QM(df = 1) = 0.0138, p-val = 0.9064

Model Results:

         estimate      se     zval    pval    ci.lb   ci.ub
intrcpt   -0.8338  0.5416  -1.5396  0.1237  -1.8953  0.2277
mods       0.0772  0.6567   0.1176  0.9064  -1.2099  1.3643

---
Signif. codes:  0 ‘***’ 0.001 ‘**’ 0.01 ‘*’ 0.05 ‘.’ 0.1 ‘ ’ 1

[1] "Antipsychotics"
[1] 3

Multivariate Meta-Analysis Model (k = 12; method: REML)

Variance Components:

            estim    sqrt  nlvls  fixed          factor
```

© 2021 Bentzley BS et al. *JAMA Network Open*.

```
sigma^2.1  0.3001  0.5478      8     no          Author
sigma^2.2  0.0000  0.0000      9     no  Author/StudyID

Test for Residual Heterogeneity:
QE(df = 10) = 15.9437, p-val = 0.1013

Test of Moderators (coefficient(s) 2):
QM(df = 1) = 0.0138, p-val = 0.9064

Model Results:

         estimate      se     zval    pval    ci.lb   ci.ub
intrcpt   -0.8338  0.5416  -1.5396  0.1237  -1.8953  0.2277
mods       0.0772  0.6567   0.1176  0.9064  -1.2099  1.3643

---
Signif. codes:  0 '***' 0.001 '**' 0.01 '*' 0.05 '.' 0.1 ' ' 1

[1] "Antipsychotics"
[1] 4

Multivariate Meta-Analysis Model (k = 12; method: REML)

Variance Components:

            estim    sqrt  nlvls  fixed          factor
sigma^2.1  1.8713  1.3679      8     no          Author
sigma^2.2  0.0000  0.0000      9     no  Author/StudyID

Test for Residual Heterogeneity:
QE(df = 10) = 38.0970, p-val < .0001

Test of Moderators (coefficient(s) 2):
QM(df = 1) = 0.9041, p-val = 0.3417

Model Results:

         estimate      se     zval    pval    ci.lb   ci.ub
intrcpt    0.5121  0.8768   0.5841  0.5592  -1.2064  2.2307
mods      -0.7907  0.8315  -0.9509  0.3417  -2.4205  0.8391

---
Signif. codes:  0 '***' 0.001 '**' 0.01 '*' 0.05 '.' 0.1 ' ' 1

[1] "Antipsychotics"
[1] 5

Multivariate Meta-Analysis Model (k = 12; method: REML)

Variance Components:

            estim    sqrt  nlvls  fixed          factor
sigma^2.1  0.5057  0.7111      8     no          Author
```

© 2021 Bentzley BS et al. *JAMA Network Open*.

```
sigma^2.2  0.0000  0.0000      9     no  Author/StudyID

Test for Residual Heterogeneity:
QE(df = 10) = 18.7211, p-val = 0.0440

Test of Moderators (coefficient(s) 2):
QM(df = 1) = 0.2555, p-val = 0.6133

Model Results:

         estimate      se     zval    pval    ci.lb   ci.ub
intrcpt   -0.2947  0.5895  -0.4998  0.6172  -1.4501  0.8608
mods      -0.3438  0.6802  -0.5054  0.6133  -1.6769  0.9893

---
Signif. codes:  0 '***' 0.001 '**' 0.01 '*' 0.05 '.' 0.1 ' ' 1

[1] "Full Model"
[1] 0

Multivariate Meta-Analysis Model (k = 314; method: REML)

Variance Components:

            estim    sqrt  nlvls  fixed          factor
sigma^2.1  0.9313  0.9650     79     no          Author
sigma^2.2  0.6442  0.8026    120     no  Author/StudyID

Test for Residual Heterogeneity:
QE(df = 312) = 1056.9190, p-val < .0001

Test of Moderators (coefficient(s) 2):
QM(df = 1) = 2.4776, p-val = 0.1155

Model Results:

         estimate      se    zval    pval    ci.lb   ci.ub
intrcpt    0.4636  0.2063  2.2476  0.0246   0.0593  0.8679  *
mods       0.3130  0.1989  1.5740  0.1155  -0.0767  0.7028

---
Signif. codes:  0 '***' 0.001 '**' 0.01 '*' 0.05 '.' 0.1 ' ' 1

[1] "Full Model"
[1] 1

Multivariate Meta-Analysis Model (k = 402; method: REML)

Variance Components:

            estim    sqrt  nlvls  fixed          factor
sigma^2.1  0.9940  0.9970     98     no          Author
sigma^2.2  0.7667  0.8756    157     no  Author/StudyID
```

© 2021 Bentzley BS et al. *JAMA Network Open*.

```
Test for Residual Heterogeneity:
QE(df = 400) = 1356.3866, p-val < .0001

Test of Moderators (coefficient(s) 2):
QM(df = 1) = 24.2349, p-val < .0001

Model Results:

         estimate      se    zval    pval   ci.lb   ci.ub
intrcpt    0.3899  0.1967  1.9824  0.0474  0.0044  0.7755    *
mods       0.8445  0.1716  4.9229  <.0001  0.5083  1.1808  ***

---
Signif. codes:  0 '***' 0.001 '**' 0.01 '*' 0.05 '.' 0.1 ' ' 1

[1] "Full Model"
[1] 2

Multivariate Meta-Analysis Model (k = 402; method: REML)

Variance Components:

            estim    sqrt  nlvls  fixed          factor
sigma^2.1  0.9341  0.9665     98     no          Author
sigma^2.2  0.9837  0.9918    157     no  Author/StudyID

Test for Residual Heterogeneity:
QE(df = 400) = 1717.7091, p-val < .0001

Test of Moderators (coefficient(s) 2):
QM(df = 1) = 15.8142, p-val < .0001

Model Results:

         estimate      se     zval    pval    ci.lb   ci.ub
intrcpt   -0.1829  0.1875  -0.9756  0.3293  -0.5503  0.1845
mods       0.7109  0.1788   3.9767  <.0001   0.3605  1.0613  ***

---
Signif. codes:  0 '***' 0.001 '**' 0.01 '*' 0.05 '.' 0.1 ' ' 1

[1] "Full Model"
[1] 3

Multivariate Meta-Analysis Model (k = 402; method: REML)

Variance Components:

            estim    sqrt  nlvls  fixed          factor
sigma^2.1  1.0729  1.0358     98     no          Author
sigma^2.2  1.1675  1.0805    157     no  Author/StudyID
```

© 2021 Bentzley BS et al. *JAMA Network Open*.

```
Test for Residual Heterogeneity:
QE(df = 400) = 1767.5876, p-val < .0001

Test of Moderators (coefficient(s) 2):
QM(df = 1) = 1.0675, p-val = 0.3015

Model Results:

         estimate      se    zval    pval    ci.lb   ci.ub
intrcpt    0.0389  0.1978  0.1967  0.8441  -0.3489  0.4267
mods       0.1963  0.1900  1.0332  0.3015  -0.1761  0.5687

---
Signif. codes:  0 '***' 0.001 '**' 0.01 '*' 0.05 '.' 0.1 ' ' 1

[1] "Full Model"
[1] 4

Multivariate Meta-Analysis Model (k = 402; method: REML)

Variance Components:

            estim    sqrt  nlvls  fixed          factor
sigma^2.1  1.1213  1.0589     98     no          Author
sigma^2.2  0.6921  0.8319    157     no  Author/StudyID

Test for Residual Heterogeneity:
QE(df = 400) = 1310.6344, p-val < .0001

Test of Moderators (coefficient(s) 2):
QM(df = 1) = 14.5114, p-val = 0.0001

Model Results:

         estimate      se    zval    pval   ci.lb   ci.ub
intrcpt    0.5723  0.2068  2.7669  0.0057  0.1669  0.9776   **
mods       0.6885  0.1807  3.8094  0.0001  0.3343  1.0428  ***

---
Signif. codes:  0 '***' 0.001 '**' 0.01 '*' 0.05 '.' 0.1 ' ' 1

[1] "Full Model"
[1] 5

Multivariate Meta-Analysis Model (k = 402; method: REML)

Variance Components:

            estim    sqrt  nlvls  fixed          factor
sigma^2.1  0.5233  0.7234     98     no          Author
sigma^2.2  1.3207  1.1492    157     no  Author/StudyID

Test for Residual Heterogeneity:
```

```
QE(df = 400) = 1355.9961, p-val < .0001

Test of Moderators (coefficient(s) 2):
QM(df = 1) = 7.4209, p-val = 0.0064

Model Results:

         estimate      se    zval    pval   ci.lb   ci.ub
intrcpt    0.3985  0.1964  2.0291  0.0425  0.0136  0.7834   *
mods       0.5050  0.1854  2.7241  0.0064  0.1417  0.8684  **

---
Signif. codes:  0 '***' 0.001 '**' 0.01 '*' 0.05 '.' 0.1 ' ' 1
```

© 2021 Bentzley BS et al. *JAMA Network Open*.

eMethods 3. Model Outputs in Data Sets (Subsetted by Treatment Category)

```
[1] "Placebo"
[1] 0

Mixed-Effects Model (k = 77; tau^2 estimator: REML)

tau^2 (estimated amount of residual heterogeneity):     1.2507 (SE = 0.3318)
tau (square root of estimated tau^2 value):             1.1183
I^2 (residual heterogeneity / unaccounted variability): 68.72%
H^2 (unaccounted variability / sampling variability):   3.20
R^2 (amount of heterogeneity accounted for):            38.12%

Test for Residual Heterogeneity:
QE(df = 71) = 201.5267, p-val < .0001

Test of Moderators (coefficient(s) 2,3,4,5,6):
QM(df = 5) = 31.8170, p-val < .0001

Model Results:

                 estimate       se     zval    pval      ci.lb    ci.ub
intrcpt          -67.2407  71.1746  -0.9447  0.3448  -206.7404  72.2590
FU                -0.0229   0.0445  -0.5158  0.6060    -0.1101   0.0642
Year               0.0343   0.0360   0.9524  0.3409    -0.0363   0.1048
Proportion_Male   -0.3931   1.0174  -0.3864  0.6992    -2.3872   1.6009
Mean_Age          -0.0663   0.0420  -1.5780  0.1146    -0.1486   0.0160
Complete           3.3005   0.6761   4.8815  <.0001     1.9753   4.6257  ***

---
Signif. codes:  0 '***' 0.001 '**' 0.01 '*' 0.05 '.' 0.1 ' ' 1


[1] "Psychotherapy"
[1] 0

Mixed-Effects Model (k = 246; tau^2 estimator: REML)

tau^2 (estimated amount of residual heterogeneity):     0.9985 (SE = 0.1483)
tau (square root of estimated tau^2 value):             0.9992
I^2 (residual heterogeneity / unaccounted variability): 68.46%
H^2 (unaccounted variability / sampling variability):   3.17
R^2 (amount of heterogeneity accounted for):            44.29%

Test for Residual Heterogeneity:
QE(df = 240) = 650.7375, p-val < .0001

Test of Moderators (coefficient(s) 2,3,4,5,6):
QM(df = 5) = 110.1070, p-val < .0001

Model Results:

                 estimate       se     zval    pval      ci.lb     ci.ub
```

© 2021 Bentzley BS et al. *JAMA Network Open*.

```
intrcpt          -93.8624  35.5015  -2.6439  0.0082  -163.4441  -24.2807   **
FU                 0.0173   0.0148   1.1721  0.2412    -0.0117    0.0463
Year               0.0468   0.0179   2.6107  0.0090     0.0117    0.0820   **
Proportion_Male    0.0331   0.5195   0.0637  0.9492    -0.9850    1.0512
Mean_Age          -0.0521   0.0249  -2.0894  0.0367    -0.1009   -0.0032    *
Complete           3.5270   0.3595   9.8098  <.0001     2.8223    4.2317  ***

---
Signif. codes:  0 '***' 0.001 '**' 0.01 '*' 0.05 '.' 0.1 ' ' 1


[1] "ContingencyManagement"
[1] 0

Mixed-Effects Model (k = 62; tau^2 estimator: REML)

tau^2 (estimated amount of residual heterogeneity):     0.6040 (SE = 0.2047)
tau (square root of estimated tau^2 value):             0.7772
I^2 (residual heterogeneity / unaccounted variability): 59.98%
H^2 (unaccounted variability / sampling variability):   2.50
R^2 (amount of heterogeneity accounted for):            35.17%

Test for Residual Heterogeneity:
QE(df = 56) = 134.9547, p-val < .0001

Test of Moderators (coefficient(s) 2,3,4,5,6):
QM(df = 5) = 24.4281, p-val = 0.0002

Model Results:

                 estimate       se     zval    pval    ci.lb     ci.ub
intrcpt          171.9289  71.3211   2.4106  0.0159  32.1421  311.7158   *
FU                 0.0538   0.0230   2.3386  0.0194   0.0087    0.0989   *
Year              -0.0864   0.0359  -2.4102  0.0159  -0.1567   -0.0161   *
Proportion_Male    0.6389   0.9249   0.6908  0.4897  -1.1739    2.4517
Mean_Age           0.0051   0.0439   0.1167  0.9071  -0.0809    0.0912
Complete           1.6922   0.6446   2.6249  0.0087   0.4287    2.9556  **

---
Signif. codes:  0 '***' 0.001 '**' 0.01 '*' 0.05 '.' 0.1 ' ' 1


[1] "Other"
[1] 0

Mixed-Effects Model (k = 18; tau^2 estimator: REML)

tau^2 (estimated amount of residual heterogeneity):     1.0731 (SE = 0.6586)
tau (square root of estimated tau^2 value):             1.0359
I^2 (residual heterogeneity / unaccounted variability): 80.40%
H^2 (unaccounted variability / sampling variability):   5.10
R^2 (amount of heterogeneity accounted for):            34.10%
```

© 2021 Bentzley BS et al. *JAMA Network Open*.

```
Test for Residual Heterogeneity:
QE(df = 12) = 33.7279, p-val = 0.0007

Test of Moderators (coefficient(s) 2,3,4,5,6):
QM(df = 5) = 8.6743, p-val = 0.1228

Model Results:

                 estimate        se     zval    pval      ci.lb     ci.ub
intrcpt           93.7221  323.5241   0.2897  0.7721  -540.3735  727.8176
FU                 0.0467    0.0996   0.4694  0.6388    -0.1485    0.2420
Year              -0.0442    0.1645  -0.2685  0.7883    -0.3667    0.2783
Proportion_Male   -4.2648    2.7354  -1.5591  0.1190    -9.6261    1.0966
Mean_Age          -0.1248    0.1747  -0.7146  0.4748    -0.4672    0.2175
Complete           4.2506    2.3611   1.8003  0.0718    -0.3771    8.8783  .

---
Signif. codes:  0 '***' 0.001 '**' 0.01 '*' 0.05 '.' 0.1 ' ' 1


[1] "Opioid"
[1] 0

Mixed-Effects Model (k = 136; tau^2 estimator: REML)

tau^2 (estimated amount of residual heterogeneity):     0.9458 (SE = 0.1993)
tau (square root of estimated tau^2 value):             0.9725
I^2 (residual heterogeneity / unaccounted variability): 63.92%
H^2 (unaccounted variability / sampling variability):   2.77
R^2 (amount of heterogeneity accounted for):            0.81%

Test for Residual Heterogeneity:
QE(df = 130) = 339.7699, p-val < .0001

Test of Moderators (coefficient(s) 2,3,4,5,6):
QM(df = 5) = 5.9468, p-val = 0.3114

Model Results:

                 estimate       se     zval    pval      ci.lb    ci.ub
intrcpt          -27.3325  47.3302  -0.5775  0.5636  -120.0981  65.4330
FU                -0.0182   0.0160  -1.1410  0.2539    -0.0495   0.0131
Year               0.0147   0.0239   0.6155  0.5382    -0.0321   0.0615
Proportion_Male    0.5005   0.6413   0.7804  0.4352    -0.7565   1.7574
Mean_Age          -0.0446   0.0392  -1.1358  0.2560    -0.1215   0.0323
Complete           0.8222   0.7258   1.1328  0.2573    -0.6004   2.2447

---
Signif. codes:  0 '***' 0.001 '**' 0.01 '*' 0.05 '.' 0.1 ' ' 1


[1] "MiscMeds"
[1] 0
```

© 2021 Bentzley BS et al. *JAMA Network Open*.

```
Mixed-Effects Model (k = 37; tau^2 estimator: REML)

tau^2 (estimated amount of residual heterogeneity):     1.0429 (SE = 0.4521)
tau (square root of estimated tau^2 value):             1.0212
I^2 (residual heterogeneity / unaccounted variability): 68.96%
H^2 (unaccounted variability / sampling variability):   3.22
R^2 (amount of heterogeneity accounted for):            0.00%

Test for Residual Heterogeneity:
QE(df = 31) = 78.6164, p-val < .0001

Test of Moderators (coefficient(s) 2,3,4,5,6):
QM(df = 5) = 5.6360, p-val = 0.3433

Model Results:

                  estimate        se     zval    pval      ci.lb     ci.ub
intrcpt          -133.5635  124.7241  -1.0709  0.2842  -378.0182  110.8913
FU                 -0.0270    0.0890  -0.3031  0.7618    -0.2015    0.1475
Year                0.0689    0.0630   1.0947  0.2736    -0.0545    0.1923
Proportion_Male    -2.8305    1.9968  -1.4175  0.1563    -6.7442    1.0832
Mean_Age           -0.0602    0.0710  -0.8471  0.3969    -0.1994    0.0791
Complete            0.7056    1.2845   0.5493  0.5828    -1.8119    3.2232

---
Signif. codes:  0 '***' 0.001 '**' 0.01 '*' 0.05 '.' 0.1 ' ' 1


[1] "Psychostimulants"
[1] 0

Mixed-Effects Model (k = 19; tau^2 estimator: REML)

tau^2 (estimated amount of residual heterogeneity):     0.2349 (SE = 0.2926)
tau (square root of estimated tau^2 value):             0.4847
I^2 (residual heterogeneity / unaccounted variability): 31.36%
H^2 (unaccounted variability / sampling variability):   1.46
R^2 (amount of heterogeneity accounted for):            83.31%

Test for Residual Heterogeneity:
QE(df = 13) = 23.3007, p-val = 0.0382

Test of Moderators (coefficient(s) 2,3,4,5,6):
QM(df = 5) = 24.8851, p-val = 0.0001

Model Results:

                  estimate        se     zval    pval      ci.lb    ci.ub
intrcpt          -138.9877  112.7082  -1.2332  0.2175  -359.8916  81.9162
FU                 -0.0531    0.0420  -1.2633  0.2065    -0.1354   0.0293
Year                0.0714    0.0573   1.2447  0.2133    -0.0410   0.1837
Proportion_Male    -4.5710    3.1437  -1.4540  0.1459   -10.7325   1.5906
```

© 2021 Bentzley BS et al. *JAMA Network Open*.

```
Mean_Age           -0.0426    0.0590  -0.7220  0.4703    -0.1583   0.0731
Complete            3.4549    1.5874   2.1764  0.0295     0.3436   6.5662  *

---
Signif. codes:  0 '***' 0.001 '**' 0.01 '*' 0.05 '.' 0.1 ' ' 1


[1] "Anticonvulsants"
[1] 0

Mixed-Effects Model (k = 17; tau^2 estimator: REML)

tau^2 (estimated amount of residual heterogeneity):     0.1374 (SE = 0.3008)
tau (square root of estimated tau^2 value):             0.3707
I^2 (residual heterogeneity / unaccounted variability): 18.75%
H^2 (unaccounted variability / sampling variability):   1.23
R^2 (amount of heterogeneity accounted for):            92.71%

Test for Residual Heterogeneity:
QE(df = 11) = 10.8030, p-val = 0.4599

Test of Moderators (coefficient(s) 2,3,4,5,6):
QM(df = 5) = 42.7143, p-val < .0001

Model Results:

                 estimate       se     zval    pval      ci.lb      ci.ub
intrcpt         -450.2186  93.9553  -4.7918  <.0001  -634.3675  -266.0696  ***
FU                -0.1105   0.1346  -0.8213  0.4115    -0.3743     0.1532
Year               0.2263   0.0475   4.7680  <.0001     0.1333     0.3193  ***
Proportion_Male    2.2222   2.4267   0.9157  0.3598    -2.5340     6.9783
Mean_Age          -0.1005   0.0682  -1.4738  0.1405    -0.2342     0.0332
Complete           0.3519   1.0986   0.3203  0.7487    -1.8013     2.5052

---
Signif. codes:  0 '***' 0.001 '**' 0.01 '*' 0.05 '.' 0.1 ' ' 1


[1] "DopamineAgonists"
[1] 0

Mixed-Effects Model (k = 18; tau^2 estimator: REML)

tau^2 (estimated amount of residual heterogeneity):     0.0022 (SE = 0.1692)
tau (square root of estimated tau^2 value):             0.0470
I^2 (residual heterogeneity / unaccounted variability): 0.46%
H^2 (unaccounted variability / sampling variability):   1.00
R^2 (amount of heterogeneity accounted for):            99.39%

Test for Residual Heterogeneity:
QE(df = 12) = 13.3007, p-val = 0.3476

Test of Moderators (coefficient(s) 2,3,4,5,6):
```

© 2021 Bentzley BS et al. *JAMA Network Open*.

```
QM(df = 5) = 18.8892, p-val = 0.0020

Model Results:

                  estimate       se     zval    pval      ci.lb     ci.ub    
intrcpt          -211.1726  85.1506  -2.4800  0.0131  -378.0647  -44.2805   * 
FU                  0.0491   0.0797   0.6164  0.5376    -0.1070    0.2052     
Year                0.1063   0.0430   2.4747  0.0133     0.0221    0.1905   * 
Proportion_Male    -4.0561   1.9347  -2.0965  0.0360    -7.8480   -0.2642   * 
Mean_Age           -0.0587   0.0454  -1.2945  0.1955    -0.1476    0.0302     
Complete            5.8243   2.0009   2.9108  0.0036     1.9026    9.7461  ** 

---
Signif. codes:  0 '***' 0.001 '**' 0.01 '*' 0.05 '.' 0.1 ' ' 1


[1] "Antidepressants"
[1] 0

Mixed-Effects Model (k = 15; tau^2 estimator: REML)

tau^2 (estimated amount of residual heterogeneity):     0.7642 (SE = 0.8718)
tau (square root of estimated tau^2 value):             0.8742
I^2 (residual heterogeneity / unaccounted variability): 41.66%
H^2 (unaccounted variability / sampling variability):   1.71
R^2 (amount of heterogeneity accounted for):            58.42%

Test for Residual Heterogeneity:
QE(df = 9) = 15.0497, p-val = 0.0896

Test of Moderators (coefficient(s) 2,3,4,5,6):
QM(df = 5) = 14.1740, p-val = 0.0145

Model Results:

                  estimate        se     zval    pval      ci.lb     ci.ub    
intrcpt          -253.1938  193.7648  -1.3067  0.1913  -632.9658  126.5781    
FU                 -0.6719    0.3315  -2.0268  0.0427    -1.3217   -0.0221  * 
Year                0.1345    0.1005   1.3381  0.1809    -0.0625    0.3316    
Proportion_Male     1.4309    1.3665   1.0471  0.2950    -1.2474    4.1091    
Mean_Age           -0.2401    0.3421  -0.7016  0.4829    -0.9106    0.4305    
Complete            3.3947    2.8201   1.2038  0.2287    -2.1325    8.9219    

---
Signif. codes:  0 '***' 0.001 '**' 0.01 '*' 0.05 '.' 0.1 ' ' 1


[1] "Antipsychotics"
[1] 0

Mixed-Effects Model (k = 11; tau^2 estimator: REML)

tau^2 (estimated amount of residual heterogeneity):     0.0409 (SE = 0.4647)
```

© 2021 Bentzley BS et al. *JAMA Network Open*.

```
tau (square root of estimated tau^2 value):             0.2022
I^2 (residual heterogeneity / unaccounted variability): 5.14%
H^2 (unaccounted variability / sampling variability):   1.05
R^2 (amount of heterogeneity accounted for):            89.77%

Test for Residual Heterogeneity:
QE(df = 5) = 5.4069, p-val = 0.3683

Test of Moderators (coefficient(s) 2,3,4,5,6):
QM(df = 5) = 9.9983, p-val = 0.0753

Model Results:

                  estimate        se     zval    pval      ci.lb     ci.ub
intrcpt          -201.4559  275.3147  -0.7317  0.4643  -741.0628  338.1510
FU                  0.0087    0.0381   0.2286  0.8192    -0.0660    0.0834
Year                0.0998    0.1399   0.7132  0.4758    -0.1744    0.3740
Proportion_Male     3.9847    2.8952   1.3763  0.1687    -1.6897    9.6591
Mean_Age           -0.0911    0.1947  -0.4677  0.6400    -0.4727    0.2905
Complete            2.8446    2.1043   1.3518  0.1764    -1.2797    6.9689

---
Signif. codes:  0 '***' 0.001 '**' 0.01 '*' 0.05 '.' 0.1 ' ' 1
```

© 2021 Bentzley BS et al. *JAMA Network Open*.

eFigure 1. Forest Plot Without Imputed Data: Anticonvulsants

Group ID, Study ID, Author, Year	N	Treatment Categories	Odds Ratio [95% CI]
10, 5, Baldacara, L, 2016	30	Anticonvulsants, Psychotherapy	59.18 [7.10, 493.42]
15, 8, Brodie, J, 2009	50	Anticonvulsants, Psychotherapy	27.00 [3.44, 211.82]
16, 9, Brodie, J, 2003	20	Anticonvulsants	12.67 [1.40, 114.42]
37, 18, Crosby, R, 1996	20	Anticonvulsants, Psychotherapy	0.20 [0.05, 0.83]
91, 39, Gonzalez, G, 2007	26	Anticonvulsants, Psychotherapy, Opioid	1.00 [0.18, 5.48]
92, 39, Gonzalez, G, 2007	25	Anticonvulsants, Psychotherapy, Opioid	2.85 [0.64, 12.64]
94, 40, Gonzalez, G, 2003	15	Anticonvulsants, Psychotherapy, Opioid	1.00 [0.22, 4.56]
95, 40, Gonzalez, G, 2003	15	Anticonvulsants, Psychotherapy, Opioid	1.00 [0.22, 4.56]
114, 46, Halikas, J, 1997	49	Anticonvulsants, Psychotherapy	0.69 [0.29, 1.61]
115, 46, Halikas, J, 1997	50	Anticonvulsants, Psychotherapy	0.13 [0.05, 0.35]
146, 58, Kampman, K, 2004	20	Anticonvulsants, Psychotherapy	1.22 [0.35, 4.24]
196, 79, Mancino, M, 2014	34	Antidepressants, Anticonvulsants, Psychotherapy	10.00 [1.18, 84.78]
250, 100, Myrick, H, 2001	30	Anticonvulsants	1.00 [0.23, 4.43]
315, 122, Reid, M, 2005	17	Anticonvulsants, Psychotherapy	4.80 [0.48, 47.68]
323, 126, Salloum, I, 2007	7	Anticonvulsants, Psychotherapy	1.00 [0.12, 8.31]
387, 151, Somoza, E, 2013	92	Anticonvulsants, Psychotherapy	8.65 [1.06, 70.61]
402, 157, Winhusen, T, 2007	70	Anticonvulsants, Psychotherapy	1.00 [0.39, 2.58]
RE Model for All Studies (N = 570, Q = 62.77, df = 16, p = 1.78e–07; I^2 = 77.3%)			1.82 [0.84, 3.91]

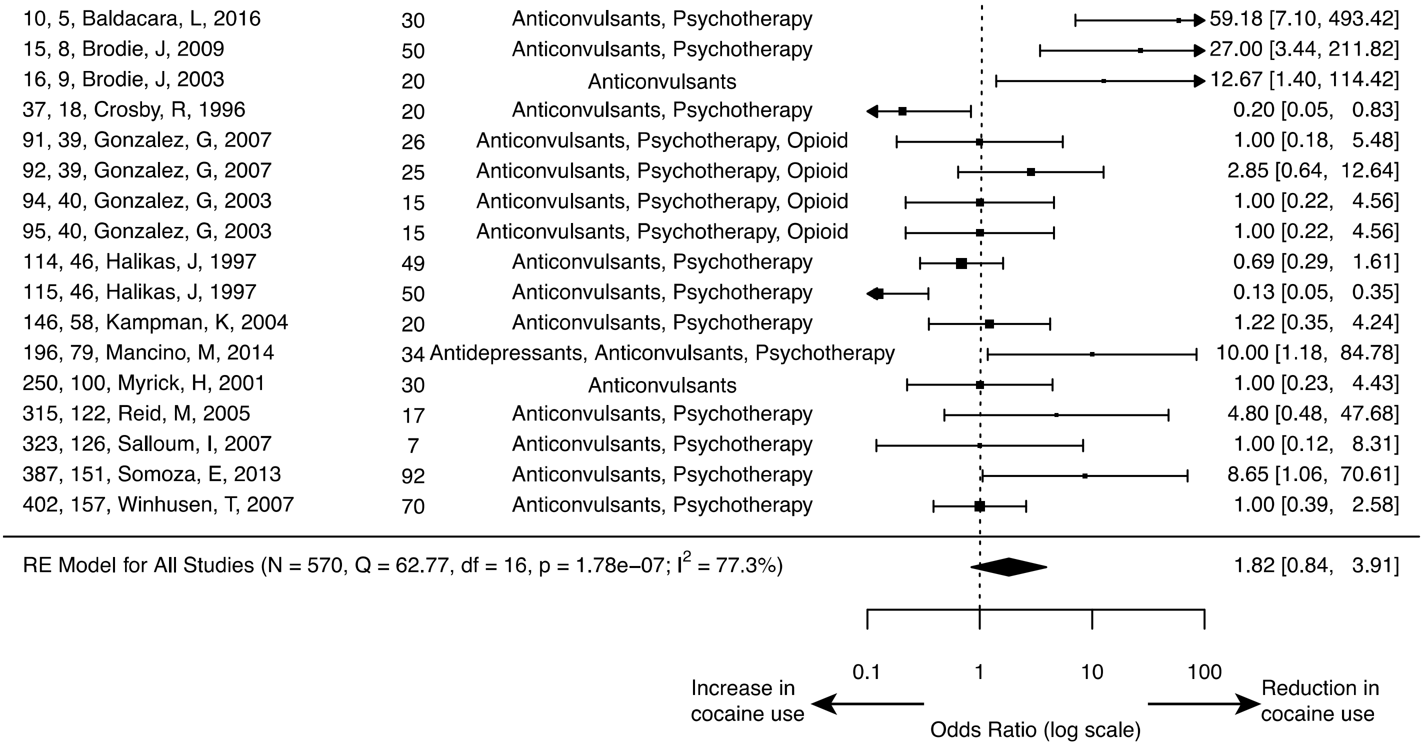

© 2021 Bentzley BS et al. *JAMA Network Open*.

eFigure 2. Forest Plot Without Imputed Data: Antidepressants

Group ID, Study ID, Author, Year	N	Treatment Categories	Odds Ratio [95% CI]
3, 2, Avants, S, 1998	6	Antidepressants, Psychotherapy, Opioid	7.00 [0.57, 86.32]
68, 29, Feingold, A, 2002	52	Antidepressants, Psychotherapy, Opioid	18.55 [2.34, 146.99]
69, 29, Feingold, A, 2002	57	Antidepressants, Psychotherapy, Opioid	23.48 [3.01, 183.37]
166, 66, Kosten, T, 2003	40	Antidepressants, CM, Opioid	12.81 [1.56, 105.36]
167, 66, Kosten, T, 2003	40	Antidepressants, Opioid	6.83 [0.79, 59.48]
196, 79, Mancino, M, 2014	34	Antidepressants, Anticonvulsants, Psychotherapy	10.00 [1.18, 84.78]
197, 79, Mancino, M, 2014	35	Antidepressants, Psychotherapy	9.93 [1.17, 84.04]
257, 102, Oliveto, A, 1999	35	Antidepressants, Psychotherapy, Opioid	24.55 [3.03, 198.96]
258, 102, Oliveto, A, 1999	24	Antidepressants, Psychotherapy, Opioid	15.63 [1.82, 134.04]
259, 102, Oliveto, A, 1999	10	Antidepressants, Psychotherapy, Opioid	2.20 [0.17, 28.14]
260, 102, Oliveto, A, 1999	21	Antidepressants, Psychotherapy, Opioid	6.11 [0.65, 57.15]
296, 115, Poling, J, 2006	27	Antidepressants, CM, Psychotherapy, Opioid	34.46 [4.12, 288.45]
297, 115, Poling, J, 2006	30	Antidepressants, Psychotherapy, Opioid	14.09 [1.68, 118.22]
326, 128, Schmitz, J, 2001	34	Antidepressants, Psychotherapy	0.32 [0.10, 1.03]
352, 137, Shoptaw, S, 2008	37	Antidepressants, Psychotherapy	0.38 [0.13, 1.10]
RE Model for All Studies (N = 482, Q = 54.07, df = 14, p = 1.25e−06; I^2 = 66.4%)			6.32 [2.66, 15.00]

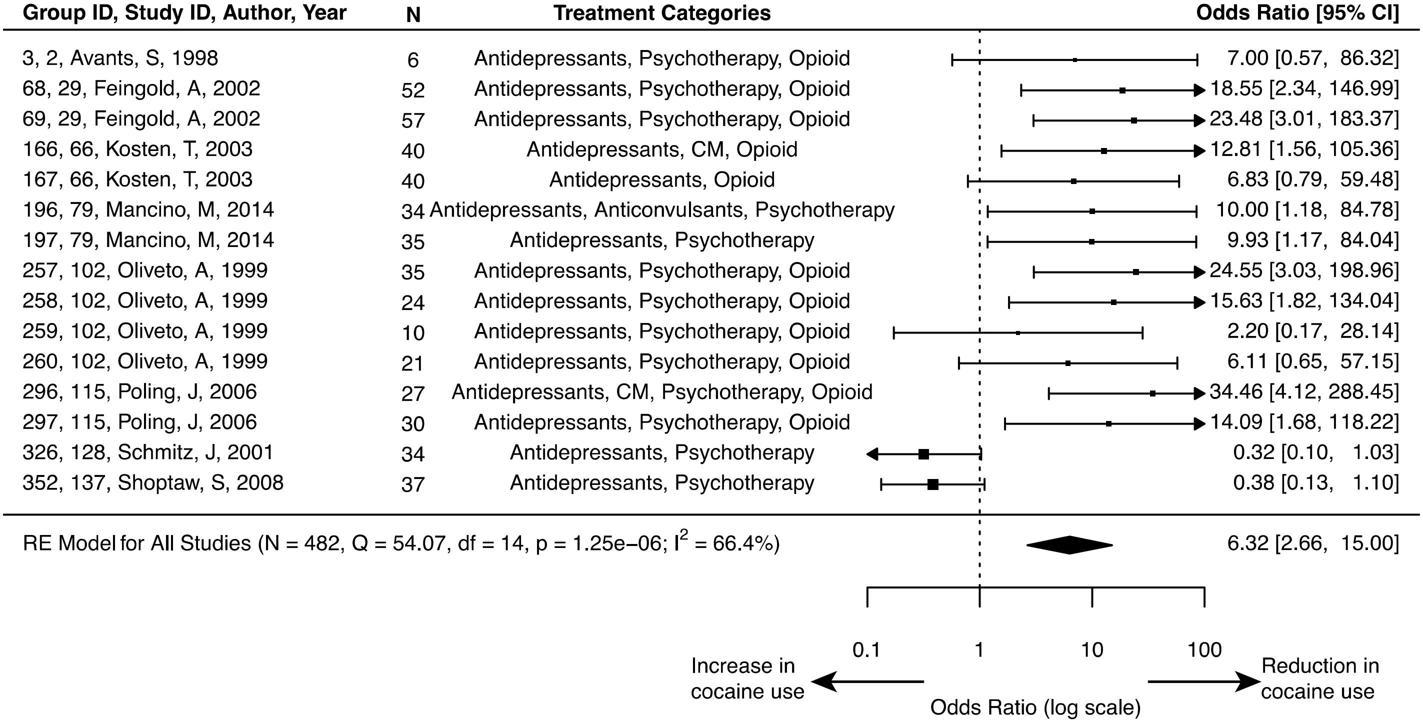

© 2021 Bentzley BS et al. *JAMA Network Open*.

eFigure 3. Forest Plot Without Imputed Data: Antipsychotics

Group ID, Study ID, Author, Year	N	Treatment Categories	Odds Ratio [95% CI]
11, 6, Beresford, T, 2005	9	Antipsychotics	1.56 [0.24, 10.03]
19, 11, Brown, E, 2002	17	Antipsychotics	0.13 [0.03, 0.59]
102, 42, Grabowski, J, 2004	32	Antipsychotics, Psychotherapy, Opioid	0.16 [0.05, 0.53]
103, 42, Grabowski, J, 2004	31	Antipsychotics, Psychotherapy, Opioid	0.66 [0.18, 2.36]
110, 45, Grabowski, J, 2000	30	Antipsychotics, Psychotherapy	0.37 [0.08, 1.58]
111, 45, Grabowski, J, 2000	38	Antipsychotics, Psychotherapy	0.05 [0.01, 0.42]
112, 45, Grabowski, J, 2000	12	Antipsychotics, Psychotherapy	0.19 [0.02, 2.00]
144, 57, Kampman, K, 2003	15	Antipsychotics, Psychotherapy	0.29 [0.03, 3.12]
314, 122, Reid, M, 2005	16	Antipsychotics, Psychotherapy	2.12 [0.18, 25.78]
324, 127, Sayers, S, 2005	12	Antipsychotics	1.50 [0.25, 8.84]
389, 152, Tapp, A, 2015	29	Antipsychotics, Psychotherapy	0.80 [0.21, 2.98]
RE Model for All Studies (N = 241, Q = 16.00, df = 10, p = 9.96e−02; I^2 = 36.1%)			0.40 [0.21, 0.76]

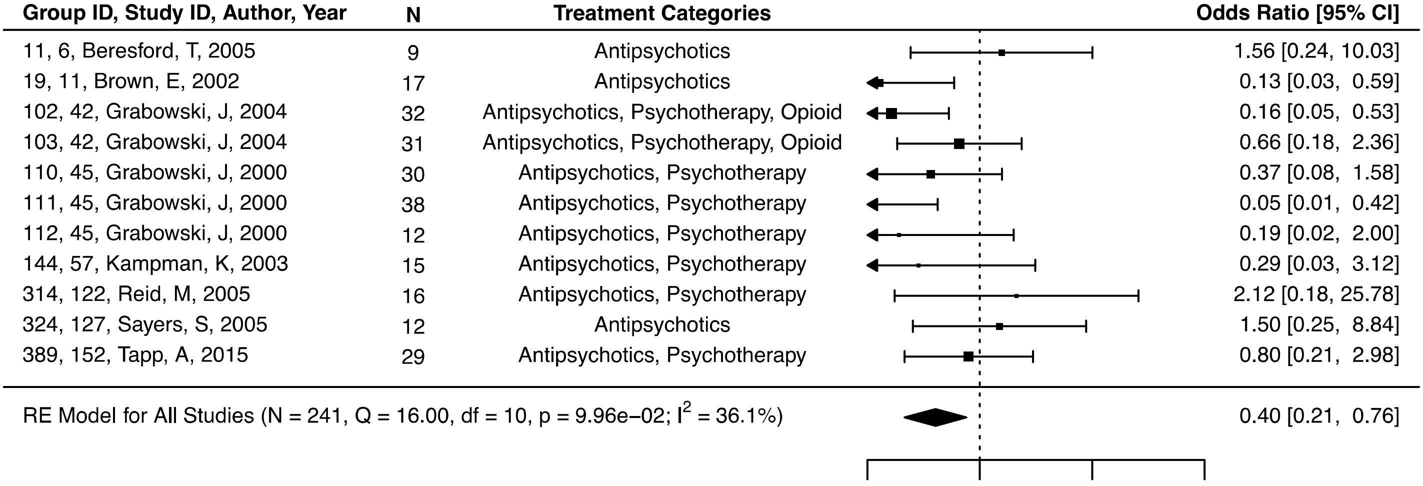

© 2021 Bentzley BS et al. *JAMA Network Open*.

eFigure 4. Forest Plot Without Imputed Data: Contingency Management

(1 of 2)

Group ID, Study ID, Author, Year	N	Treatment Categories	Odds Ratio [95% CI]
43, 21, Dallery, J, 2001	15	CM, Psychotherapy, Opioid	3.50 [0.32, 38.23]
44, 22, Donlin, W, 2008	111	CM, Opioid	11.05 [5.23, 23.37]
45, 23, Dunn, K, 2014	16	CM	9.00 [0.94, 86.52]
56, 27, Epstein, D, 2003	49	CM, Psychotherapy, Opioid	6.76 [1.81, 25.23]
57, 27, Epstein, D, 2003	47	CM, Other, Opioid	3.94 [1.30, 11.97]
62, 28, Epstein, D, 2009	49	CM, Psychotherapy, Opioid	2.91 [1.07, 7.89]
63, 28, Epstein, D, 2009	47	CM, Psychotherapy, Opioid	2.34 [0.80, 6.89]
64, 28, Epstein, D, 2009	48	CM, Psychotherapy, Opioid	2.66 [0.97, 7.29]
65, 28, Epstein, D, 2009	47	CM, Psychotherapy, Opioid	2.61 [0.90, 7.61]
83, 36, Ghitza, U, 2007	20	CM, Opioid, Psychotherapy	2.10 [0.18, 25.01]
84, 36, Ghitza, U, 2007	36	CM, Opioid, Psychotherapy	6.94 [0.79, 60.71]
85, 36, Ghitza, U, 2007	20	CM, Opioid, Psychotherapy	6.18 [0.66, 58.03]
121, 50, Higgins, S, 2003	49	CM, Psychotherapy	3.91 [1.63, 9.37]
122, 50, Higgins, S, 2003	51	CM	1.61 [0.73, 3.54]
125, 51, Holtyn, A, 2014	33	CM, Opioid, Other	2.51 [0.90, 7.00]
130, 53, Jones, H, 2004	58	Placebo, CM, Psychotherapy	14.75 [1.85, 117.51]
132, 53, Jones, H, 2004	45	MiscMeds, CM, Psychotherapy	9.44 [1.13, 78.78]
154, 62, Katz, E, 2002	40	CM, Psychotherapy, Opioid	22.78 [2.84, 182.64]
158, 64, Kirby, K, 2013	62	CM, Opioid, Psychotherapy	22.79 [2.93, 177.34]
159, 64, Kirby, K, 2013	68	CM, Opioid, Psychotherapy	36.00 [4.71, 275.45]
164, 66, Kosten, T, 2003	40	Placebo, CM, Opioid	11.18 [1.35, 92.81]
166, 66, Kosten, T, 2003	40	Antidepressants, CM, Opioid	12.81 [1.56, 105.36]
186, 75, Liu, S, 2014	20	Placebo, Psychotherapy, CM	0.62 [0.16, 2.43]
187, 75, Liu, S, 2014	13	Placebo, Psychotherapy, CM	0.28 [0.02, 3.10]
188, 75, Liu, S, 2014	26	DA, Psychotherapy, CM	0.68 [0.20, 2.32]
189, 75, Liu, S, 2014	12	DA, Psychotherapy, CM	0.60 [0.08, 4.45]
227, 91, Miguel, A, 2016	33	Psychotherapy, CM	0.89 [0.34, 2.33]
228, 92, Milby, J, 2000	56	CM, Psychotherapy	1.95 [0.91, 4.17]
230, 93, Milby, J, 2008	103	CM, Psychotherapy	1.24 [0.65, 2.34]
231, 93, Milby, J, 2008	103	CM	1.26 [0.69, 2.28]
240, 97, Mooney, M, 2009	27	CM, Placebo, Psychotherapy	0.50 [0.13, 1.95]
241, 97, Mooney, M, 2009	30	Psychostimulants, CM, Psychotherapy	0.26 [0.06, 1.08]
242, 97, Mooney, M, 2009	25	Psychostimulants, CM, Psychotherapy	1.00 [0.29, 3.44]
261, 103, Oliveto, A, 2005	35	CM, Psychotherapy, Opioid	11.57 [1.38, 96.81]
262, 103, Oliveto, A, 2005	35	CM, Psychotherapy, Opioid	17.28 [2.11, 141.51]
269, 105, Petitjean, S, 2014	29	CM, Psychotherapy	2.45 [0.82, 7.31]
273, 107, Petry, N, 2012	147	CM, Psychotherapy	0.42 [0.26, 0.69]
274, 107, Petry, N, 2012	153	CM, Psychotherapy	0.35 [0.21, 0.58]
279, 109, Petry, N, 2002	19	CM, Psychotherapy, Opioid	1.54 [0.42, 5.64]
281, 110, Petry, N, 2005	40	CM, Psychotherapy, Opioid	2.22 [0.86, 5.74]
284, 111, Petry, N, 2004	45	CM, Psychotherapy	0.11 [0.04, 0.32]
285, 111, Petry, N, 2004	38	CM, Psychotherapy	0.19 [0.07, 0.50]
286, 112, Petry, N, 2007	28	CM, Psychotherapy, Opioid	0.75 [0.26, 2.15]
287, 112, Petry, N, 2007	27	CM, Psychotherapy, Opioid	1.16 [0.40, 3.37]
294, 115, Poling, J, 2006	25	Placebo, CM, Psychotherapy, Opioid	10.95 [1.26, 95.06]
296, 115, Poling, J, 2006	27	Antidepressants, CM, Psychotherapy, Opioid	34.46 [4.12, 288.45]
300, 117, Preston, K, 2008	29	CM, Psychotherapy, Opioid	1.25 [0.34, 4.68]
301, 117, Preston, K, 2008	38	CM, Psychotherapy, Opioid	1.92 [0.51, 7.19]

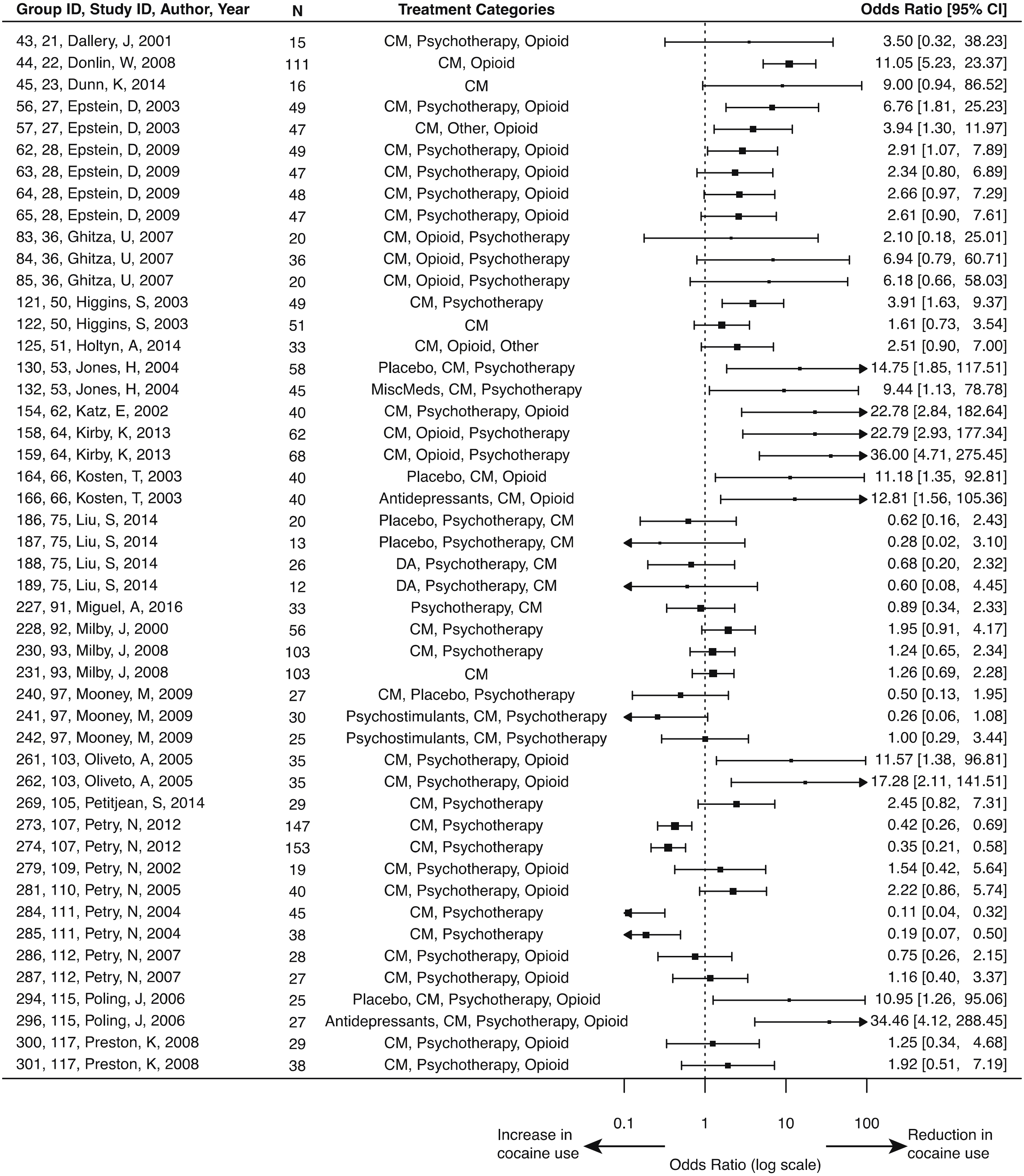

© 2021 Bentzley BS et al. *JAMA Network Open*.

Contingency Management (2 of 2)

Group ID, Study ID, Author, Year	N	Treatment Categories	Odds Ratio [95% CI]
302, 118, Preston, K, 2001	49	CM, Psychotherapy, Opioid	2.55 [0.81, 7.99]
303, 118, Preston, K, 2001	46	CM, Psychotherapy, Opioid	2.44 [0.88, 6.76]
306, 120, Rawson, R, 2002	27	CM, Psychotherapy, Opioid	39.67 [4.73, 332.84]
307, 120, Rawson, R, 2002	26	CM, Psychotherapy, Opioid	23.40 [2.78, 196.84]
319, 125, Rowan−Szal, G, 2005	13	CM, Psychotherapy, Opioid	3.89 [0.72, 21.06]
320, 125, Rowan−Szal, G, 2005	17	CM, Psychotherapy, Opioid	6.67 [1.38, 32.28]
329, 129, Schmitz, J, 2008	27	Placebo, Psychotherapy, CM	0.17 [0.02, 1.57]
332, 129, Schmitz, J, 2008	23	DA, Psychotherapy, CM	1.00 [0.28, 3.51]
337, 131, Schottenfeld, R, 2005	40	CM, Psychotherapy, Opioid	2.22 [0.86, 5.74]
338, 131, Schottenfeld, R, 2005	39	CM, Psychotherapy, Opioid	1.18 [0.38, 3.65]
364, 142, Sigmon, S, 2004	14	CM, Psychotherapy, Opioid	15.00 [2.26, 99.64]
365, 142, Sigmon, S, 2004	13	CM, Psychotherapy, Opioid	4.71 [0.73, 30.28]
367, 143, Silverman, K, 2007	28	CM, Psychotherapy, Opioid	6.16 [1.19, 31.82]
369, 144, Silverman, K, 2004	26	CM, Psychotherapy, Opioid	7.50 [2.01, 28.05]
373, 146, Silverman, K, 1998	20	CM, Psychotherapy, Opioid	3.78 [0.83, 17.25]
374, 146, Silverman, K, 1998	20	CM, Psychotherapy, Opioid	6.93 [1.53, 31.38]
376, 147, Silverman, K, 1996	19	CM, Psychotherapy, Opioid	18.18 [2.05, 161.38]
378, 148, Silverman, K, 1999	29	CM, Psychotherapy, Opioid	18.95 [2.28, 157.76]
380, 148, Silverman, K, 1999	29	CM, Psychotherapy, Opioid	5.77 [0.63, 52.61]
396, 155, Wardle, M, 2017	40	Placebo, CM, Psychotherapy	1.89 [0.61, 5.82]
397, 155, Wardle, M, 2017	45	DA, CM, Psychotherapy	1.36 [0.46, 4.03]
RE Model for All Studies (N = 2744, Q = 289.36, df = 68, p = 4.30e−29; I^2 = 76.7%)			2.52 [1.85, 3.44]

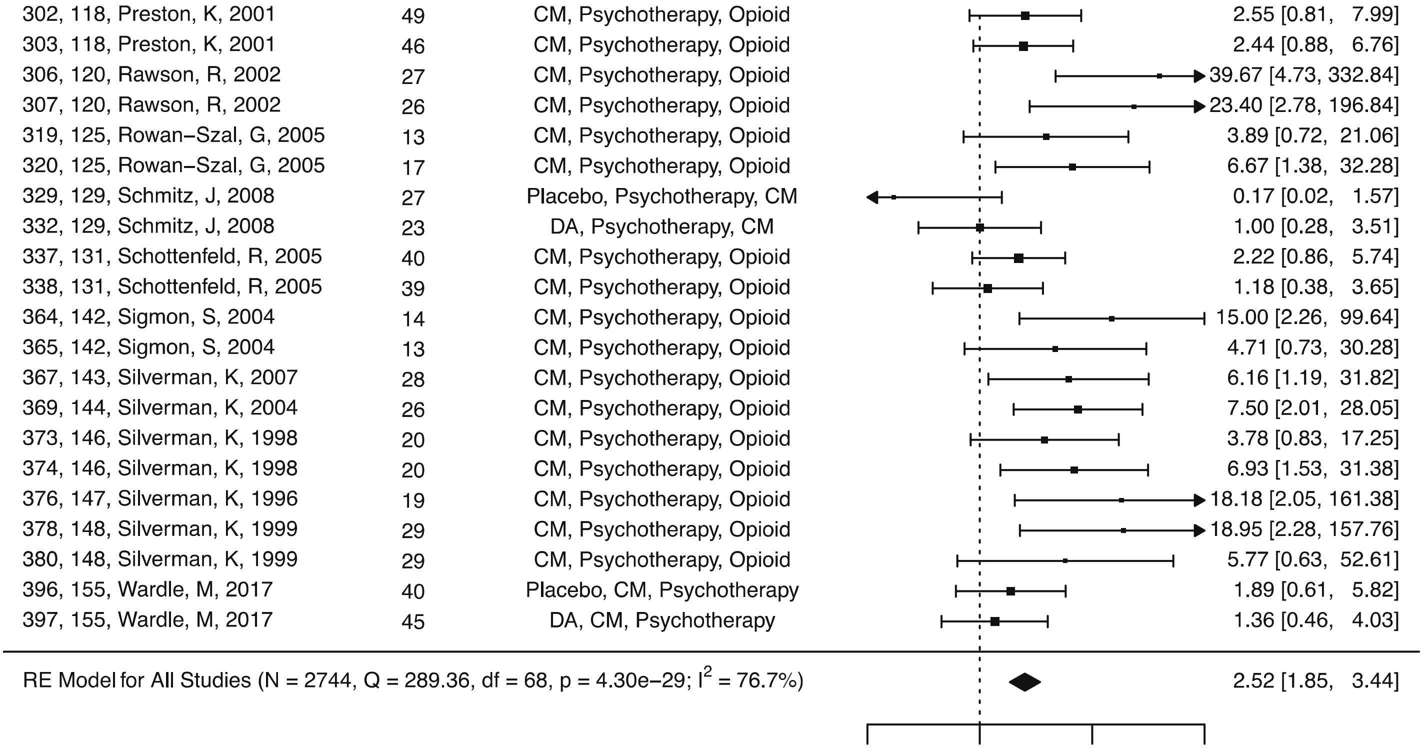

© 2021 Bentzley BS et al. *JAMA Network Open*.

eFigure 5. Forest Plot Without Imputed Data: Dopamine Agonists

Group ID, Study ID, Author, Year	N	Treatment Categories	Odds Ratio [95% CI]
71, 30, Focchi, G, 2005	22	DA	2.67 [0.76, 9.37]
117, 47, Handelsman, L, 1997	24	DA, Psychotherapy, Opioid	0.40 [0.10, 1.57]
140, 56, Kampman, K, 2006	50	DA, MiscMeds, Psychotherapy	5.43 [0.61, 48.15]
141, 56, Kampman, K, 2006	50	DA, Psychotherapy	7.93 [0.94, 66.98]
188, 75, Liu, S, 2014	26	DA, Psychotherapy, CM	0.68 [0.20, 2.32]
189, 75, Liu, S, 2014	12	DA, Psychotherapy, CM	0.60 [0.08, 4.45]
194, 78, Malcolm, R, 2001	84	DA	0.45 [0.21, 0.93]
195, 78, Malcolm, R, 2001	79	DA	0.28 [0.10, 0.75]
247, 99, Mooney, M, 2007	31	DA, Psychotherapy	0.50 [0.16, 1.62]
248, 99, Mooney, M, 2007	43	DA, Psychotherapy	0.34 [0.11, 0.98]
249, 99, Mooney, M, 2007	39	DA, Psychotherapy	0.37 [0.12, 1.21]
330, 129, Schmitz, J, 2008	25	DA	0.43 [0.09, 1.97]
331, 129, Schmitz, J, 2008	28	DA, Psychotherapy	0.46 [0.08, 2.75]
332, 129, Schmitz, J, 2008	23	DA, Psychotherapy, CM	1.00 [0.28, 3.51]
350, 136, Shoptaw, S, 2002	34	DA, Psychotherapy	0.22 [0.08, 0.62]
356, 139, Shoptaw, S, 2005	15	DA, Psychotherapy	8.73 [0.92, 82.96]
357, 139, Shoptaw, S, 2005	15	DA, Psychotherapy	1.00 [0.06, 17.41]
397, 155, Wardle, M, 2017	45	DA, CM, Psychotherapy	1.36 [0.46, 4.03]
RE Model for All Studies (N = 645, Q = 32.28, df = 17, p = 1.39e−02; I^2 = 45.6%)			0.68 [0.44, 1.04]

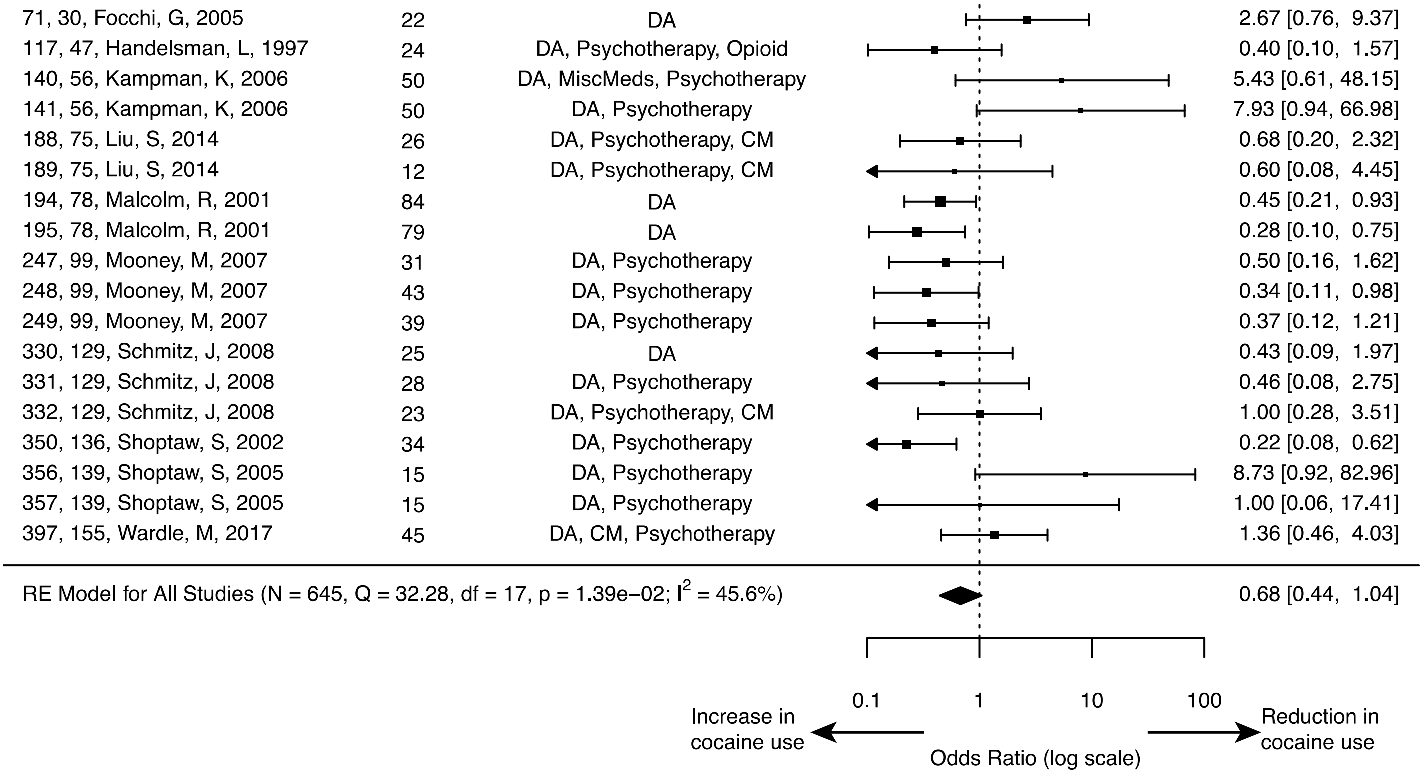

© 2021 Bentzley BS et al. *JAMA Network Open*.

eFigure 6. Forest Plot Without Imputed Data: Miscellaneous Medications

Group ID, Study ID, Author, Year	N	Treatment Categories	Odds Ratio [95% CI]
18, 10, Brown, E, 2015	61	MiscMeds, Psychotherapy	22.91 [2.94, 178.44]
54, 26, Elkashef, A, 2006	150	MiscMeds, Psychotherapy	11.78 [1.50, 92.42]
81, 35, George, T, 2000	11	MiscMeds, Psychotherapy, Opioid	2.57 [0.36, 18.33]
87, 37, Gilgun–Sherki, Y, 2016	70	MiscMeds	0.73 [0.24, 2.21]
88, 37, Gilgun–Sherki, Y, 2016	69	MiscMeds	1.29 [0.48, 3.50]
127, 52, Johnson, B, 2006	16	MiscMeds, Psychotherapy	0.43 [0.07, 2.76]
128, 52, Johnson, B, 2006	12	MiscMeds, Psychotherapy	0.14 [0.01, 1.39]
129, 52, Johnson, B, 2006	19	MiscMeds, Psychotherapy	4.80 [0.48, 47.68]
132, 53, Jones, H, 2004	45	MiscMeds, CM, Psychotherapy	9.44 [1.13, 78.78]
133, 53, Jones, H, 2004	56	MiscMeds, Psychotherapy	5.38 [0.61, 47.54]
135, 54, Kablinger, A, 2012	15	MiscMeds, MiscMeds	11.20 [1.19, 105.13]
136, 54, Kablinger, A, 2012	15	MiscMeds, MiscMeds	6.67 [0.69, 64.77]
140, 56, Kampman, K, 2006	50	DA, MiscMeds, Psychotherapy	5.43 [0.61, 48.15]
142, 56, Kampman, K, 2006	50	MiscMeds, Psychotherapy	6.77 [1.42, 32.37]
169, 67, Kosten, T, 2002	20	MiscMeds, Opioid	0.44 [0.07, 2.76]
171, 68, Kosten, T, 2013	34	MiscMeds, Psychotherapy, Opioid	1.61 [0.53, 4.88]
181, 73, Licata, S, 2011	15	MiscMeds, Psychotherapy	0.46 [0.04, 5.75]
183, 74, Ling, W, 2016	102	Placebo, MiscMeds, Psychotherapy	1.17 [0.67, 2.04]
184, 74, Ling, W, 2016	100	Opioid, MiscMeds, Psychotherapy	1.04 [0.60, 1.81]
185, 74, Ling, W, 2016	100	Opioid, MiscMeds, Psychotherapy	1.85 [1.05, 3.26]
192, 77, Malcolm, R, 2005	60	MiscMeds, Psychotherapy	0.20 [0.09, 0.47]
210, 84, Margolin, A, 1996	10	MiscMeds, Opioid	0.27 [0.02, 3.09]
266, 104, Oliveto, A, 2011	37	MiscMeds, Psychotherapy, Opioid	1.18 [0.38, 3.68]
267, 104, Oliveto, A, 2011	38	MiscMeds, Psychotherapy, Opioid	1.24 [0.34, 4.46]
268, 104, Oliveto, A, 2011	39	MiscMeds, Psychotherapy, Opioid	2.16 [0.71, 6.59]
293, 114, Plebani, J, 2012	18	MiscMeds	2.60 [0.65, 10.38]
299, 116, Poling, J, 2010	13	MiscMeds, Psychotherapy, Opioid	9.33 [0.96, 90.94]
311, 121, Reid, M, 2005	10	MiscMeds, Psychotherapy	2.20 [0.17, 28.14]
313, 122, Reid, M, 2005	15	MiscMeds, MiscMeds, Psychotherapy	2.13 [0.17, 26.03]
354, 138, Shoptaw, S, 2004	11	MiscMeds, Psychotherapy	0.27 [0.02, 3.08]
358, 139, Shoptaw, S, 2005	15	MiscMeds, Psychotherapy	3.43 [0.32, 36.83]
360, 140, Shoptaw, S, 2003	35	MiscMeds, Psychotherapy	0.52 [0.19, 1.43]
362, 141, Shorter, D, 2013	8	MiscMeds, Psychotherapy	2.25 [0.17, 29.77]
363, 141, Shorter, D, 2013	9	MiscMeds, Psychotherapy	3.75 [0.32, 43.31]
382, 149, Sofuoglu, M, 2017	37	MiscMeds, Psychotherapy, Opioid	40.00 [4.98, 320.98]
383, 149, Sofuoglu, M, 2017	35	MiscMeds, Psychotherapy, Opioid	21.91 [2.70, 178.08]
385, 150, Sofuoglu, M, 2007	30	MiscMeds, Psychotherapy, Opioid	8.68 [1.00, 75.30]
395, 154, Walsh, S, 2013	25	MiscMeds	0.35 [0.06, 1.99]
RE Model for All Studies (N = 1455, Q = 94.15, df = 37, p = 7.03e–07; I^2 = 67.6%)			1.87 [1.23, 2.85]

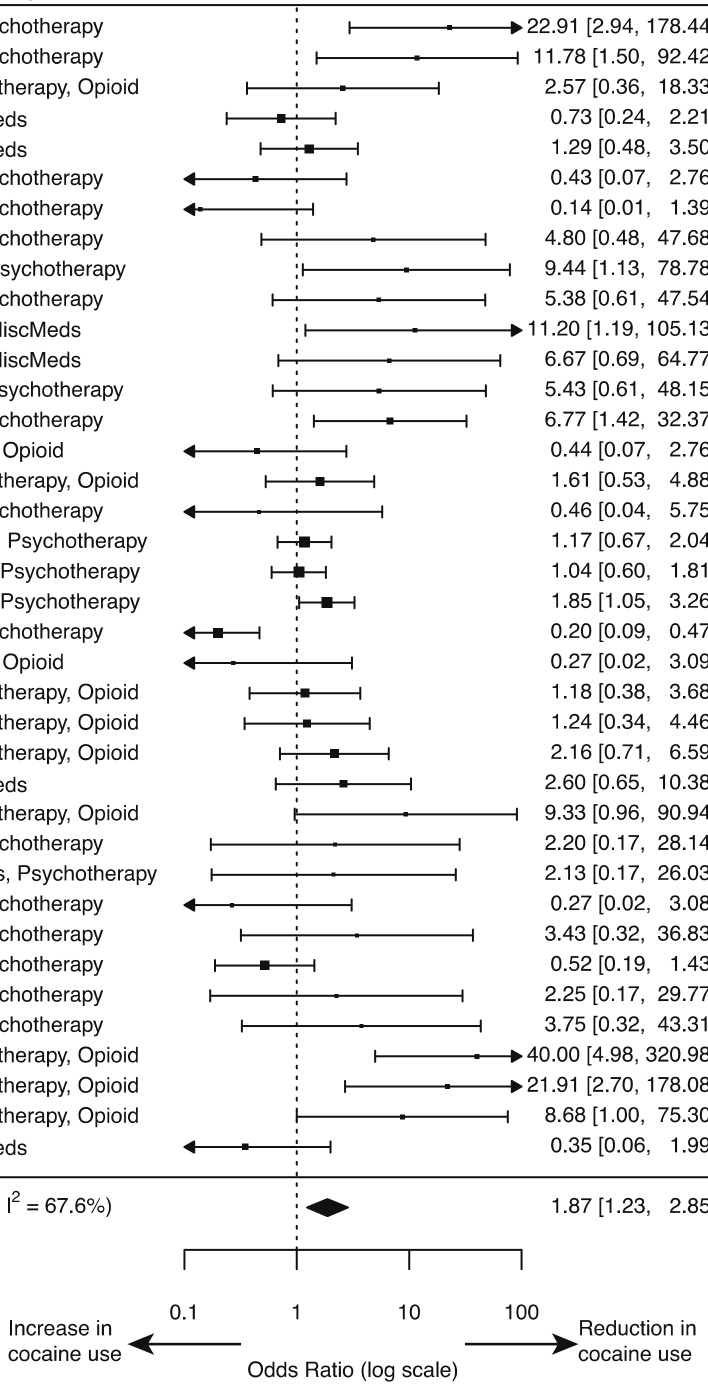

© 2021 Bentzley BS et al. *JAMA Network Open*.

eFigure 7. Forest Plot Without Imputed Data: Opioids

(1 of 3)

Group ID, Study ID, Author, Year	N	Treatment Categories	Odds Ratio [95% CI]
3, 2, Avants, S, 1998	6	Antidepressants, Psychotherapy, Opioid	7.00 [0.57, 86.32]
4, 3, Avants, S, 2000	28	Other, Opioid	5.20 [0.99, 27.23]
5, 3, Avants, S, 2000	27	Other, Opioid	14.74 [1.74, 124.83]
6, 3, Avants, S, 2000	27	Other, Opioid	3.57 [0.65, 19.59]
43, 21, Dallery, J, 2001	15	CM, Psychotherapy, Opioid	3.50 [0.32, 38.23]
44, 22, Donlin, W, 2008	111	CM, Opioid	11.05 [5.23, 23.37]
48, 24, Dursteler−MacFarland, K, 2013	17	Placebo, Psychotherapy, Opioid	3.37 [0.32, 35.79]
49, 24, Dursteler−MacFarland, K, 2013	15	Placebo, Opioid	6.67 [0.69, 64.77]
50, 24, Dursteler−MacFarland, K, 2013	15	Psychostimulants, Psychotherapy, Opioid	3.43 [0.32, 36.83]
51, 24, Dursteler−MacFarland, K, 2013	15	Psychostimulants, Opioid	4.92 [0.49, 49.61]
56, 27, Epstein, D, 2003	49	CM, Psychotherapy, Opioid	6.76 [1.81, 25.23]
57, 27, Epstein, D, 2003	47	CM, Other, Opioid	3.94 [1.30, 11.97]
58, 27, Epstein, D, 2003	49	Other, Opioid	3.28 [0.63, 17.12]
59, 27, Epstein, D, 2003	48	Psychotherapy, Opioid	1.47 [0.43, 5.00]
60, 28, Epstein, D, 2009	30	Opioid	1.00 [0.23, 4.43]
61, 28, Epstein, D, 2009	31	Opioid	1.00 [0.23, 4.42]
62, 28, Epstein, D, 2009	49	CM, Psychotherapy, Opioid	2.91 [1.07, 7.89]
63, 28, Epstein, D, 2009	47	CM, Psychotherapy, Opioid	2.34 [0.80, 6.89]
64, 28, Epstein, D, 2009	48	CM, Psychotherapy, Opioid	2.66 [0.97, 7.29]
65, 28, Epstein, D, 2009	47	CM, Psychotherapy, Opioid	2.61 [0.90, 7.61]
66, 29, Feingold, A, 2002	52	Placebo, Psychotherapy, Opioid	16.80 [2.11, 133.76]
67, 29, Feingold, A, 2002	57	Placebo, Psychotherapy, Opioid	19.77 [2.52, 155.42]
68, 29, Feingold, A, 2002	52	Antidepressants, Psychotherapy, Opioid	18.55 [2.34, 146.99]
69, 29, Feingold, A, 2002	57	Antidepressants, Psychotherapy, Opioid	23.48 [3.01, 183.37]
80, 35, George, T, 2000	9	Placebo, Psychotherapy, Opioid	6.40 [0.55, 74.89]
81, 35, George, T, 2000	11	MiscMeds, Psychotherapy, Opioid	2.57 [0.36, 18.33]
82, 36, Ghitza, U, 2007	40	Opioid, Psychotherapy	3.15 [0.31, 31.62]
83, 36, Ghitza, U, 2007	20	CM, Opioid, Psychotherapy	2.10 [0.18, 25.01]
84, 36, Ghitza, U, 2007	36	CM, Opioid, Psychotherapy	6.94 [0.79, 60.71]
85, 36, Ghitza, U, 2007	20	CM, Opioid, Psychotherapy	6.18 [0.66, 58.03]
90, 39, Gonzalez, G, 2007	25	Placebo, Psychotherapy, Opioid	2.04 [0.51, 8.12]
91, 39, Gonzalez, G, 2007	26	Anticonvulsants, Psychotherapy, Opioid	1.00 [0.18, 5.48]
92, 39, Gonzalez, G, 2007	25	Anticonvulsants, Psychotherapy, Opioid	2.85 [0.64, 12.64]
93, 40, Gonzalez, G, 2003	15	Placebo, Psychotherapy, Opioid	0.76 [0.18, 3.24]
94, 40, Gonzalez, G, 2003	15	Anticonvulsants, Psychotherapy, Opioid	1.00 [0.22, 4.56]
95, 40, Gonzalez, G, 2003	15	Anticonvulsants, Psychotherapy, Opioid	1.00 [0.22, 4.56]
98, 42, Grabowski, J, 2004	40	Placebo, Psychotherapy, Opioid	0.16 [0.05, 0.46]
99, 42, Grabowski, J, 2004	33	Placebo, Psychotherapy, Opioid	0.11 [0.03, 0.40]
100, 42, Grabowski, J, 2004	26	Psychostimulants, Psychotherapy, Opioid	1.00 [0.28, 3.63]
101, 42, Grabowski, J, 2004	28	Psychostimulants, Psychotherapy, Opioid	0.72 [0.23, 2.22]
102, 42, Grabowski, J, 2004	32	Antipsychotics, Psychotherapy, Opioid	0.16 [0.05, 0.53]
103, 42, Grabowski, J, 2004	31	Antipsychotics, Psychotherapy, Opioid	0.66 [0.18, 2.36]
116, 47, Handelsman, L, 1997	26	Placebo, Psychotherapy, Opioid	2.82 [0.64, 12.44]
117, 47, Handelsman, L, 1997	24	DA, Psychotherapy, Opioid	0.40 [0.10, 1.57]
123, 51, Holtyn, A, 2014	30	Opioid, Other	1.00 [0.32, 3.14]
124, 51, Holtyn, A, 2014	35	Opioid, Other	2.53 [0.90, 7.12]
125, 51, Holtyn, A, 2014	33	CM, Opioid, Other	2.51 [0.90, 7.00]
154, 62, Katz, E, 2002	40	CM, Psychotherapy, Opioid	22.78 [2.84, 182.64]
155, 62, Katz, E, 2002	40	Psychotherapy, Opioid	12.81 [1.56, 105.36]

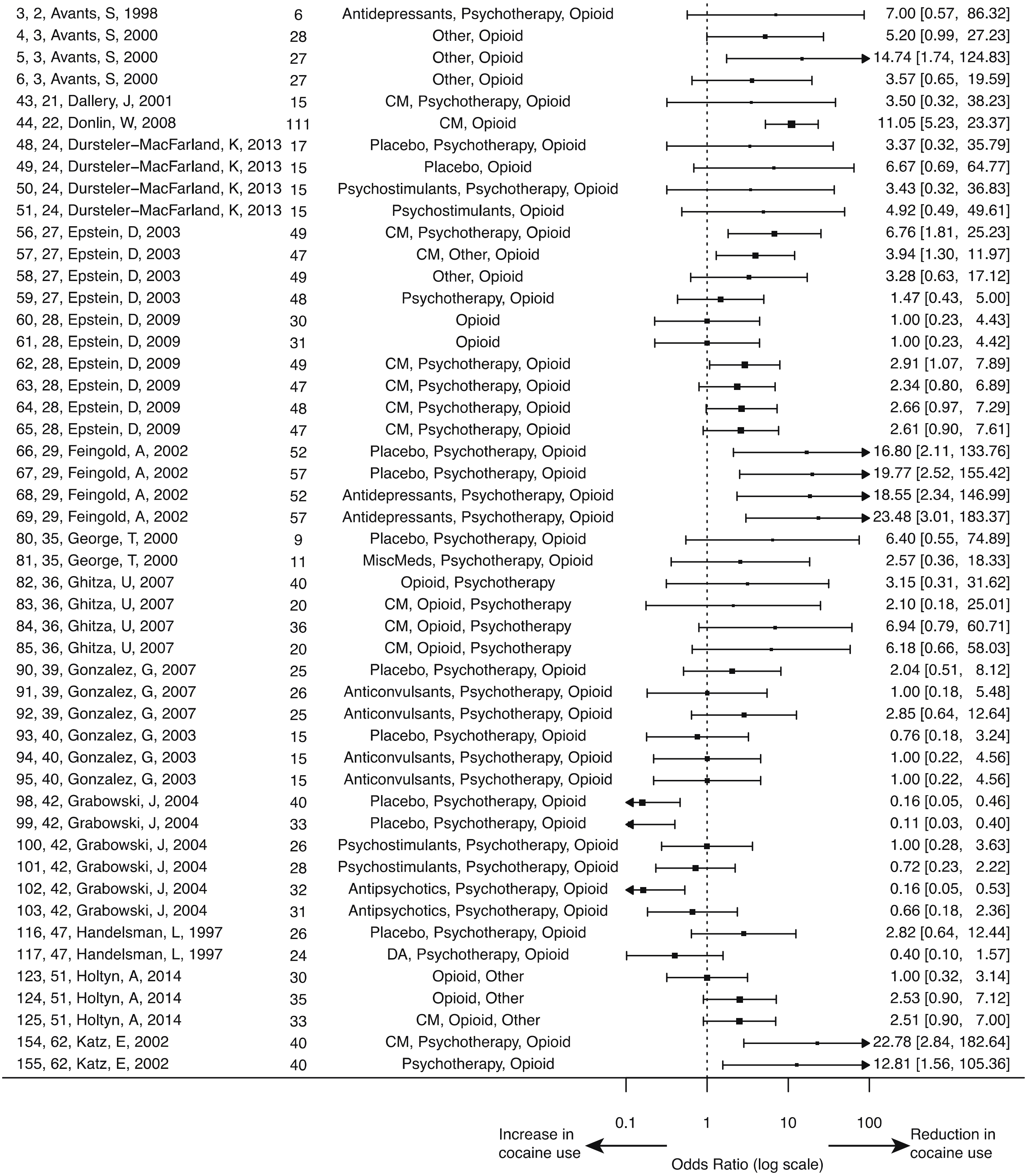

© 2021 Bentzley BS et al. *JAMA Network Open*.

Opioids (2 of 3)

Group ID, Study ID, Author, Year	N	Treatment Categories	Odds Ratio [95% CI]
158, 64, Kirby, K, 2013	62	CM, Opioid, Psychotherapy	22.79 [2.93, 177.34]
159, 64, Kirby, K, 2013	68	CM, Opioid, Psychotherapy	36.00 [4.71, 275.45]
164, 66, Kosten, T, 2003	40	Placebo, CM, Opioid	11.18 [1.35, 92.81]
165, 66, Kosten, T, 2003	40	Placebo, Opioid	8.20 [0.96, 69.93]
166, 66, Kosten, T, 2003	40	Antidepressants, CM, Opioid	12.81 [1.56, 105.36]
167, 66, Kosten, T, 2003	40	Antidepressants, Opioid	6.83 [0.79, 59.48]
168, 67, Kosten, T, 2002	19	Placebo, Opioid	1.95 [0.52, 7.31]
169, 67, Kosten, T, 2002	20	MiscMeds, Opioid	0.44 [0.07, 2.76]
170, 68, Kosten, T, 2013	40	Placebo, Psychotherapy, Opioid	1.16 [0.40, 3.40]
171, 68, Kosten, T, 2013	34	MiscMeds, Psychotherapy, Opioid	1.61 [0.53, 4.88]
184, 74, Ling, W, 2016	100	Opioid, MiscMeds, Psychotherapy	1.04 [0.60, 1.81]
185, 74, Ling, W, 2016	100	Opioid, MiscMeds, Psychotherapy	1.85 [1.05, 3.26]
190, 76, Magura, S, 1994	43	Psychotherapy, Opioid	2.70 [0.49, 14.74]
210, 84, Margolin, A, 1996	10	MiscMeds, Opioid	0.27 [0.02, 3.09]
213, 86, Martell, B, 2009	57	Placebo, Psychotherapy, Opioid	25.46 [3.27, 198.39]
214, 86, Martell, B, 2009	57	Other, Psychotherapy, Opioid	23.48 [3.01, 183.37]
236, 96, Montoya, I, 2004	46	Opioid, Psychotherapy	13.97 [1.72, 113.19]
237, 96, Montoya, I, 2004	44	Opioid, Psychotherapy	10.95 [1.33, 90.40]
238, 96, Montoya, I, 2004	43	Opioid, Psychotherapy	17.88 [2.22, 143.70]
239, 96, Montoya, I, 2004	46	Opioid, Psychotherapy	21.36 [2.69, 169.74]
253, 102, Oliveto, A, 1999	34	Placebo, Psychotherapy, Opioid	10.00 [1.18, 84.78]
254, 102, Oliveto, A, 1999	31	Placebo, Psychotherapy, Opioid	10.24 [1.20, 87.35]
255, 102, Oliveto, A, 1999	11	Placebo, Psychotherapy, Opioid	2.18 [0.17, 27.56]
256, 102, Oliveto, A, 1999	14	Placebo, Psychotherapy, Opioid	6.82 [0.69, 66.90]
257, 102, Oliveto, A, 1999	35	Antidepressants, Psychotherapy, Opioid	24.55 [3.03, 198.96]
258, 102, Oliveto, A, 1999	24	Antidepressants, Psychotherapy, Opioid	15.63 [1.82, 134.04]
259, 102, Oliveto, A, 1999	10	Antidepressants, Psychotherapy, Opioid	2.20 [0.17, 28.14]
260, 102, Oliveto, A, 1999	21	Antidepressants, Psychotherapy, Opioid	6.11 [0.65, 57.15]
261, 103, Oliveto, A, 2005	35	CM, Psychotherapy, Opioid	11.57 [1.38, 96.81]
262, 103, Oliveto, A, 2005	35	CM, Psychotherapy, Opioid	17.28 [2.11, 141.51]
263, 103, Oliveto, A, 2005	35	Opioid, Psychotherapy	13.33 [1.61, 110.56]
264, 103, Oliveto, A, 2005	35	Opioid, Psychotherapy	11.57 [1.38, 96.81]
265, 104, Oliveto, A, 2011	38	Placebo, Psychotherapy, Opioid	2.46 [0.81, 7.46]
266, 104, Oliveto, A, 2011	37	MiscMeds, Psychotherapy, Opioid	1.18 [0.38, 3.68]
267, 104, Oliveto, A, 2011	38	MiscMeds, Psychotherapy, Opioid	1.24 [0.34, 4.46]
268, 104, Oliveto, A, 2011	39	MiscMeds, Psychotherapy, Opioid	2.16 [0.71, 6.59]
279, 109, Petry, N, 2002	19	CM, Psychotherapy, Opioid	1.54 [0.42, 5.64]
280, 109, Petry, N, 2002	23	Psychotherapy, Opioid	0.34 [0.10, 1.14]
281, 110, Petry, N, 2005	40	CM, Psychotherapy, Opioid	2.22 [0.86, 5.74]
282, 110, Petry, N, 2005	37	Psychotherapy, Opioid	0.44 [0.12, 1.61]
286, 112, Petry, N, 2007	28	CM, Psychotherapy, Opioid	0.75 [0.26, 2.15]
287, 112, Petry, N, 2007	27	CM, Psychotherapy, Opioid	1.16 [0.40, 3.37]
288, 112, Petry, N, 2007	19	Psychotherapy, Opioid	0.61 [0.15, 2.44]
294, 115, Poling, J, 2006	25	Placebo, CM, Psychotherapy, Opioid	10.95 [1.26, 95.06]
295, 115, Poling, J, 2006	24	Placebo, Psychotherapy, Opioid	13.24 [1.53, 114.30]
296, 115, Poling, J, 2006	27	Antidepressants, CM, Psychotherapy, Opioid	34.46 [4.12, 288.45]
297, 115, Poling, J, 2006	30	Antidepressants, Psychotherapy, Opioid	14.09 [1.68, 118.22]
298, 116, Poling, J, 2010	18	Placebo, Psychotherapy, Opioid	10.23 [1.12, 93.34]
299, 116, Poling, J, 2010	13	MiscMeds, Psychotherapy, Opioid	9.33 [0.96, 90.94]

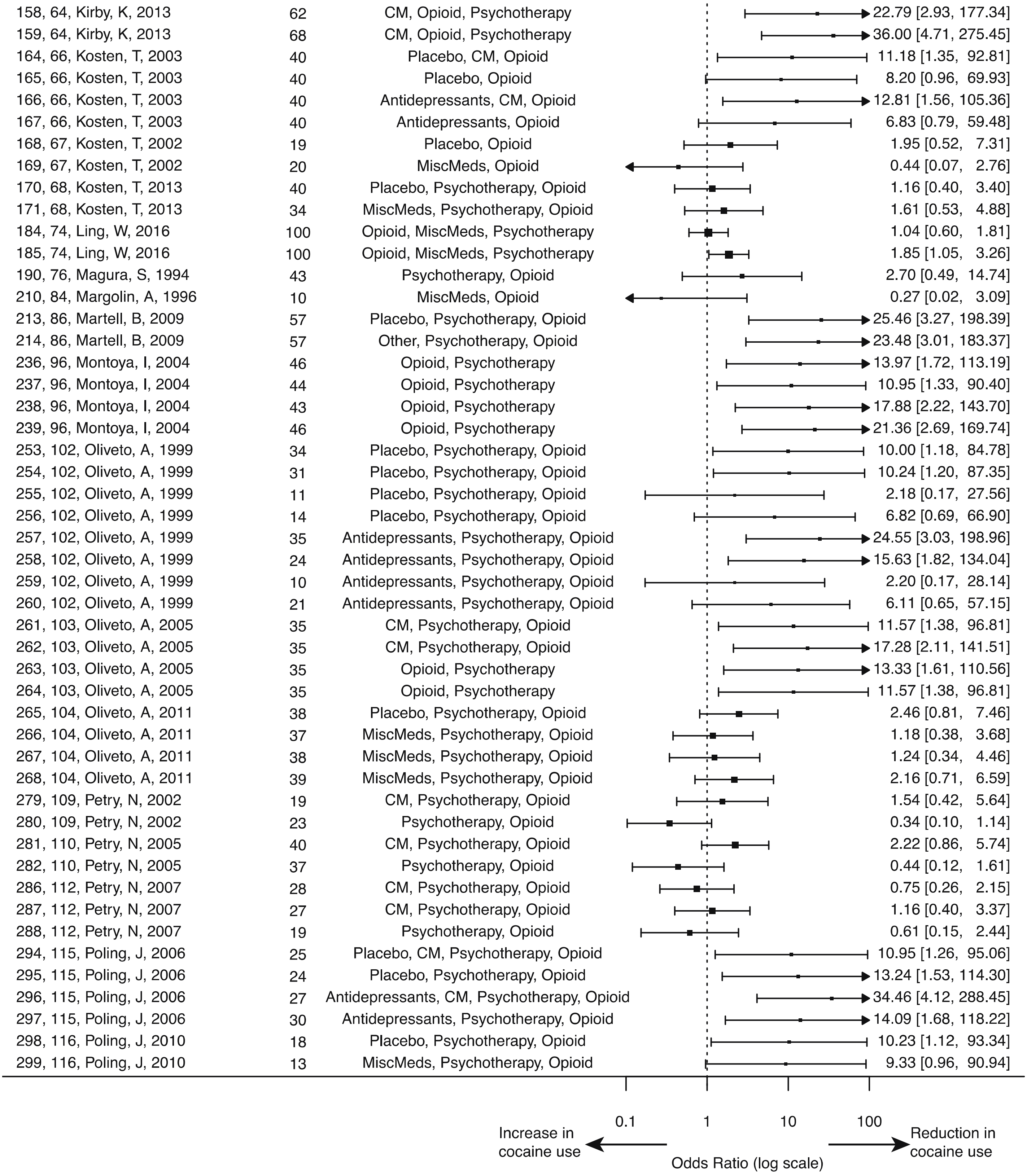

© 2021 Bentzley BS et al. *JAMA Network Open*.

Opioids (3 of 3)

Group ID, Study ID, Author, Year	N	Treatment Categories	Odds Ratio [95% CI]
300, 117, Preston, K, 2008	29	CM, Psychotherapy, Opioid	1.25 [0.34, 4.68]
301, 117, Preston, K, 2008	38	CM, Psychotherapy, Opioid	1.92 [0.51, 7.19]
302, 118, Preston, K, 2001	49	CM, Psychotherapy, Opioid	2.55 [0.81, 7.99]
303, 118, Preston, K, 2001	46	CM, Psychotherapy, Opioid	2.44 [0.88, 6.76]
306, 120, Rawson, R, 2002	27	CM, Psychotherapy, Opioid	39.67 [4.73, 332.84]
307, 120, Rawson, R, 2002	26	CM, Psychotherapy, Opioid	23.40 [2.78, 196.84]
308, 120, Rawson, R, 2002	28	Psychotherapy, Opioid	19.33 [2.31, 161.57]
309, 120, Rawson, R, 2002	27	Psychotherapy, Opioid	8.91 [1.02, 77.91]
317, 124, Rosenblum, A, 1999	140	Psychotherapy, Opioid	1.59 [0.96, 2.63]
318, 124, Rosenblum, A, 1999	58	Psychotherapy, Opioid	1.36 [0.63, 2.93]
319, 125, Rowan−Szal, G, 2005	13	CM, Psychotherapy, Opioid	3.89 [0.72, 21.06]
320, 125, Rowan−Szal, G, 2005	17	CM, Psychotherapy, Opioid	6.67 [1.38, 32.28]
321, 125, Rowan−Szal, G, 2005	15	Psychotherapy, Opioid	1.00 [0.20, 5.04]
322, 125, Rowan−Szal, G, 2005	16	Psychotherapy, Opioid	4.33 [0.88, 21.30]
333, 130, Schottenfeld, R, 1997	28	Opioid, Psychotherapy	1.18 [0.38, 3.71]
334, 130, Schottenfeld, R, 1997	29	Opioid, Psychotherapy	0.54 [0.18, 1.62]
335, 130, Schottenfeld, R, 1997	30	Opioid, Psychotherapy	0.50 [0.15, 1.62]
336, 130, Schottenfeld, R, 1997	29	Opioid, Psychotherapy	0.16 [0.04, 0.67]
337, 131, Schottenfeld, R, 2005	40	CM, Psychotherapy, Opioid	2.22 [0.86, 5.74]
338, 131, Schottenfeld, R, 2005	39	CM, Psychotherapy, Opioid	1.18 [0.38, 3.65]
339, 131, Schottenfeld, R, 2005	40	Opioid, Psychotherapy	2.73 [0.92, 8.13]
340, 131, Schottenfeld, R, 2005	43	Opioid, Psychotherapy	1.36 [0.46, 4.06]
347, 135, Shearer, J, 2003	14	Placebo, Opioid	1.00 [0.16, 6.08]
348, 135, Shearer, J, 2003	16	Psychostimulants, Opioid	11.67 [1.23, 110.95]
364, 142, Sigmon, S, 2004	14	CM, Psychotherapy, Opioid	15.00 [2.26, 99.64]
365, 142, Sigmon, S, 2004	13	CM, Psychotherapy, Opioid	4.71 [0.73, 30.28]
366, 142, Sigmon, S, 2004	15	Psychotherapy, Opioid	4.57 [0.90, 23.14]
367, 143, Silverman, K, 2007	28	CM, Psychotherapy, Opioid	6.16 [1.19, 31.82]
368, 143, Silverman, K, 2007	28	Psychotherapy, Opioid	2.17 [0.36, 12.92]
369, 144, Silverman, K, 2004	26	CM, Psychotherapy, Opioid	7.50 [2.01, 28.05]
370, 144, Silverman, K, 2004	26	Psychotherapy, Opioid	4.55 [0.47, 43.78]
373, 146, Silverman, K, 1998	20	CM, Psychotherapy, Opioid	3.78 [0.83, 17.25]
374, 146, Silverman, K, 1998	20	CM, Psychotherapy, Opioid	6.93 [1.53, 31.38]
375, 146, Silverman, K, 1998	19	Psychotherapy, Opioid	2.27 [0.36, 14.19]
376, 147, Silverman, K, 1996	19	CM, Psychotherapy, Opioid	18.18 [2.05, 161.38]
377, 147, Silverman, K, 1996	18	Psychotherapy, Opioid	2.12 [0.18, 25.78]
378, 148, Silverman, K, 1999	29	CM, Psychotherapy, Opioid	18.95 [2.28, 157.76]
379, 148, Silverman, K, 1999	29	Psychotherapy, Opioid	2.07 [0.18, 24.07]
380, 148, Silverman, K, 1999	29	CM, Psychotherapy, Opioid	5.77 [0.63, 52.61]
381, 149, Sofuoglu, M, 2017	34	Placebo, Psychotherapy, Opioid	17.50 [2.13, 143.64]
382, 149, Sofuoglu, M, 2017	37	MiscMeds, Psychotherapy, Opioid	40.00 [4.98, 320.98]
383, 149, Sofuoglu, M, 2017	35	MiscMeds, Psychotherapy, Opioid	21.91 [2.70, 178.08]
384, 150, Sofuoglu, M, 2007	15	Placebo, Psychotherapy, Opioid	8.73 [0.92, 82.96]
385, 150, Sofuoglu, M, 2007	30	MiscMeds, Psychotherapy, Opioid	8.68 [1.00, 75.30]
RE Model for All Studies (N = 4686, Q = 369.32, df = 141, p = 2.31e−22; I^2 = 65.2%)			3.05 [2.46, 3.78]

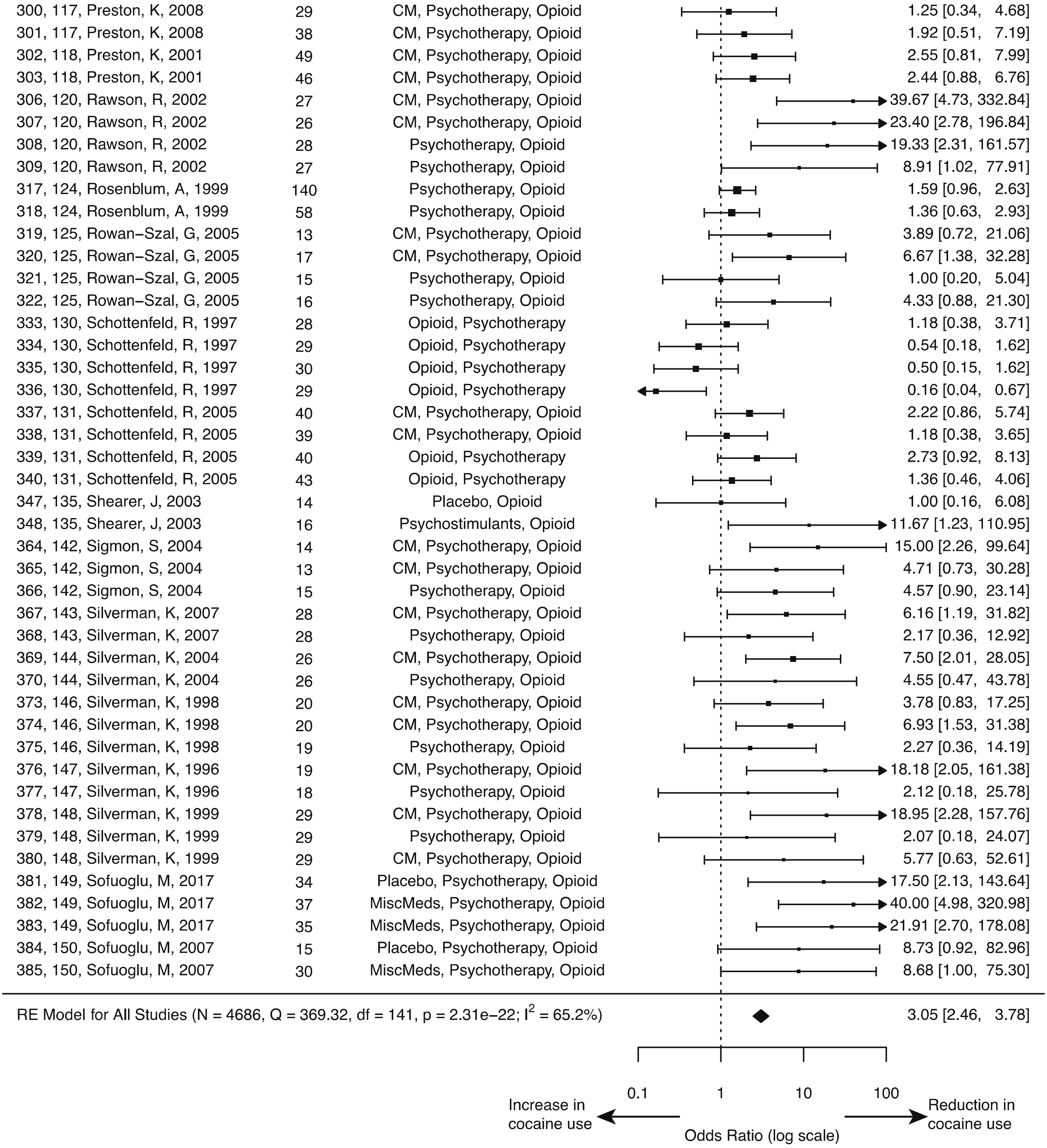

© 2021 Bentzley BS et al. *JAMA Network Open*.

eFigure 8. Forest Plot Without Imputed Data: Other Interventions

Group ID, Study ID, Author, Year	N	Treatment Categories	Odds Ratio [95% CI]
4, 3, Avants, S, 2000	28	Other, Opioid	5.20 [0.99, 27.23]
5, 3, Avants, S, 2000	27	Other, Opioid	14.74 [1.74, 124.83]
6, 3, Avants, S, 2000	27	Other, Opioid	3.57 [0.65, 19.59]
12, 7, Bernstein, J, 2005	344	Other	55.57 [7.62, 405.04]
46, 23, Dunn, K, 2014	15	Other	2.67 [0.52, 13.66]
47, 23, Dunn, K, 2014	15	Other	6.67 [0.69, 64.77]
57, 27, Epstein, D, 2003	47	CM, Other, Opioid	3.94 [1.30, 11.97]
58, 27, Epstein, D, 2003	49	Other, Opioid	3.28 [0.63, 17.12]
89, 38, Goncalves−Ferreira, A, 2016	1	Other	4.00 [0.13, 119.23]
118, 48, Haney, M, 2010	10	Other	1.00 [0.06, 18.08]
123, 51, Holtyn, A, 2014	30	Opioid, Other	1.00 [0.32, 3.14]
124, 51, Holtyn, A, 2014	35	Opioid, Other	2.53 [0.90, 7.12]
125, 51, Holtyn, A, 2014	33	CM, Opioid, Other	2.51 [0.90, 7.00]
173, 69, Kosten, T, 2014	152	Other, Psychotherapy	45.00 [6.08, 333.22]
204, 82, Margolin, A, 2002	222	Other, Psychotherapy	0.44 [0.27, 0.71]
205, 82, Margolin, A, 2002	203	Other, Psychotherapy	0.56 [0.35, 0.89]
206, 82, Margolin, A, 2002	195	Other, Psychotherapy	0.51 [0.31, 0.83]
211, 85, Martell, B, 2005	8	Other, Psychotherapy	11.67 [0.92, 147.56]
212, 85, Martell, B, 2005	10	Other, Psychotherapy	1.00 [0.17, 5.98]
214, 86, Martell, B, 2009	57	Other, Psychotherapy, Opioid	23.48 [3.01, 183.37]
215, 87, Maude−Griffin, P, 1998	69	Other	4.29 [1.60, 11.48]
RE Model for All Studies (N = 1577, Q = 105.41, df = 20, p = 1.34e−13; I^2 = 82.3%)			2.97 [1.61, 5.46]

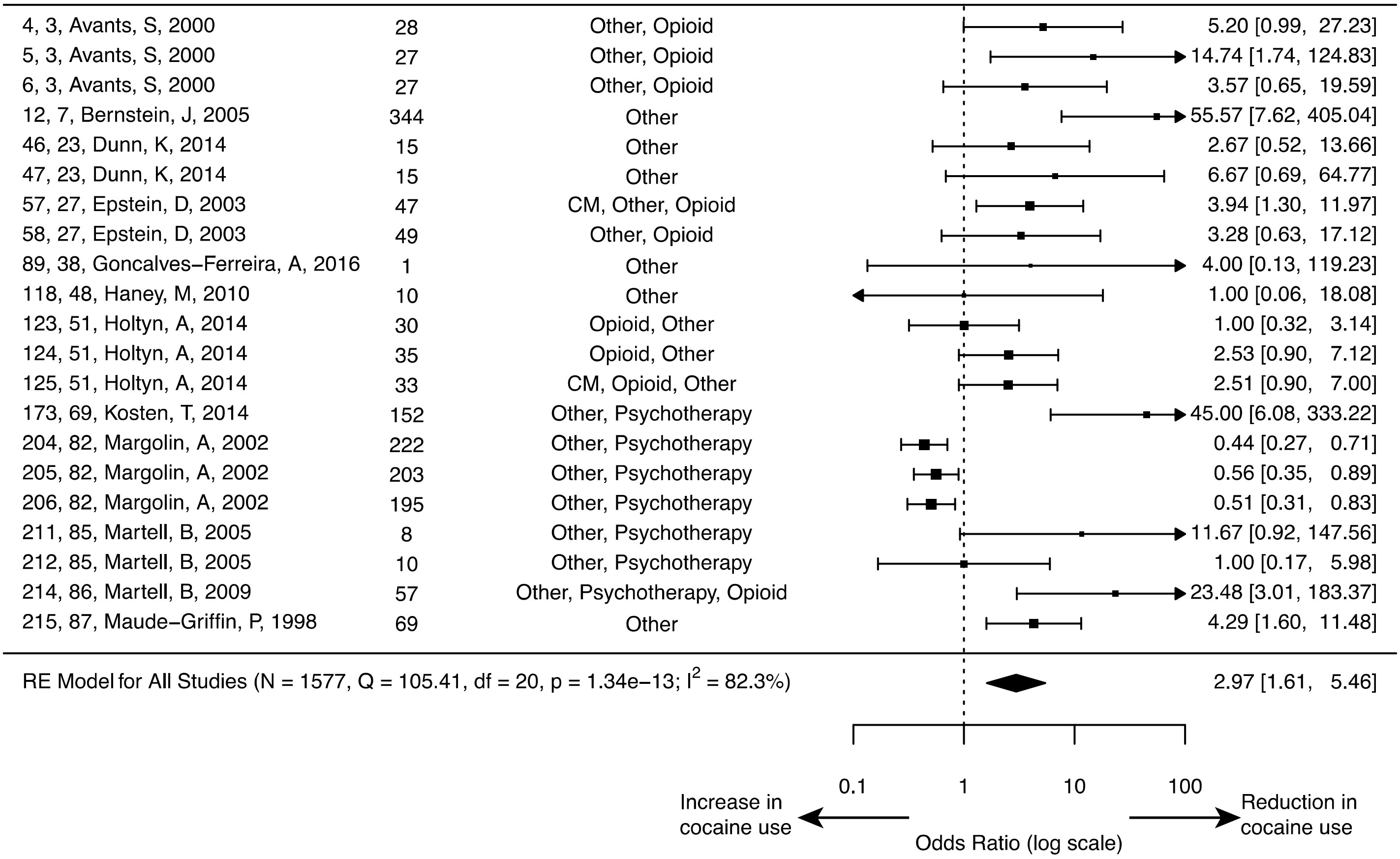

© 2021 Bentzley BS et al. *JAMA Network Open*.

eFigure 9. Forest Plot Without Imputed Data: Placebo

(1 of 2)

Group ID, Study ID, Author, Year	N	Treatment Categories	Odds Ratio [95% CI]
9, 5, Baldacara, L, 2016	30	Placebo, Psychotherapy	5.74 [0.63, 52.23]
14, 8, Brodie, J, 2009	53	Placebo, Psychotherapy	6.61 [0.77, 56.88]
17, 10, Brown, E, 2015	61	Placebo, Psychotherapy	19.37 [2.47, 151.87]
36, 18, Crosby, R, 1996	24	Placebo, Psychotherapy	0.12 [0.03, 0.52]
38, 19, Dackis, C, 2005	32	Placebo	1.19 [0.37, 3.79]
40, 20, Dackis, C, 2012	75	Placebo, Psychotherapy	2.41 [0.86, 6.73]
48, 24, Dursteler−MacFarland, K, 2013	17	Placebo, Psychotherapy, Opioid	3.37 [0.32, 35.79]
49, 24, Dursteler−MacFarland, K, 2013	15	Placebo, Opioid	6.67 [0.69, 64.77]
55, 26, Elkashef, A, 2006	150	Placebo, Psychotherapy	16.53 [2.16, 126.82]
66, 29, Feingold, A, 2002	52	Placebo, Psychotherapy, Opioid	16.80 [2.11, 133.76]
67, 29, Feingold, A, 2002	57	Placebo, Psychotherapy, Opioid	19.77 [2.52, 155.42]
70, 30, Focchi, G, 2005	20	Placebo	2.79 [0.77, 10.04]
80, 35, George, T, 2000	9	Placebo, Psychotherapy, Opioid	6.40 [0.55, 74.89]
86, 37, Gilgun−Sherki, Y, 2016	69	Placebo	0.40 [0.10, 1.63]
90, 39, Gonzalez, G, 2007	25	Placebo, Psychotherapy, Opioid	2.04 [0.51, 8.12]
93, 40, Gonzalez, G, 2003	15	Placebo, Psychotherapy, Opioid	0.76 [0.18, 3.24]
98, 42, Grabowski, J, 2004	40	Placebo, Psychotherapy, Opioid	0.16 [0.05, 0.46]
99, 42, Grabowski, J, 2004	33	Placebo, Psychotherapy, Opioid	0.11 [0.03, 0.40]
104, 43, Grabowski, J, 2001	40	Placebo, Psychotherapy	0.47 [0.08, 2.75]
107, 44, Grabowski, J, 1997	24	Placebo, Psychotherapy	0.67 [0.19, 2.34]
109, 45, Grabowski, J, 2000	45	Placebo, Psychotherapy	0.06 [0.01, 0.45]
113, 46, Halikas, J, 1997	51	Placebo, Psychotherapy	0.39 [0.16, 0.99]
116, 47, Handelsman, L, 1997	26	Placebo, Psychotherapy, Opioid	2.82 [0.64, 12.44]
126, 52, Johnson, B, 2006	16	Placebo, Psychotherapy	1.00 [0.06, 17.33]
130, 53, Jones, H, 2004	58	Placebo, CM, Psychotherapy	14.75 [1.85, 117.51]
131, 53, Jones, H, 2004	40	Placebo, Psychotherapy	5.54 [0.62, 49.63]
134, 54, Kablinger, A, 2012	15	Placebo	3.43 [0.32, 36.83]
139, 56, Kampman, K, 2006	49	Placebo, Psychotherapy	2.09 [0.36, 11.97]
143, 57, Kampman, K, 2003	15	Placebo, Psychotherapy	2.00 [0.38, 10.51]
145, 58, Kampman, K, 2004	20	Placebo, Psychotherapy	0.11 [0.02, 0.61]
164, 66, Kosten, T, 2003	40	Placebo, CM, Opioid	11.18 [1.35, 92.81]
165, 66, Kosten, T, 2003	40	Placebo, Opioid	8.20 [0.96, 69.93]
168, 67, Kosten, T, 2002	19	Placebo, Opioid	1.95 [0.52, 7.31]
170, 68, Kosten, T, 2013	40	Placebo, Psychotherapy, Opioid	1.16 [0.40, 3.40]
172, 69, Kosten, T, 2014	148	Placebo, Psychotherapy	47.05 [6.36, 348.34]
176, 71, Levin, F, 2015	43	Placebo, Psychotherapy	1.00 [0.23, 4.29]
179, 72, Levin, F, 2007	53	Placebo, Psychotherapy	0.35 [0.15, 0.82]
182, 73, Licata, S, 2011	14	Placebo, Psychotherapy	0.61 [0.09, 4.37]
183, 74, Ling, W, 2016	102	Placebo, MiscMeds, Psychotherapy	1.17 [0.67, 2.04]
186, 75, Liu, S, 2014	20	Placebo, Psychotherapy, CM	0.62 [0.16, 2.43]
187, 75, Liu, S, 2014	13	Placebo, Psychotherapy, CM	0.28 [0.02, 3.10]
191, 77, Malcolm, R, 2005	56	Placebo, Psychotherapy	0.26 [0.10, 0.65]
193, 78, Malcolm, R, 2001	92	Placebo	0.54 [0.29, 1.02]
213, 86, Martell, B, 2009	57	Placebo, Psychotherapy, Opioid	25.46 [3.27, 198.39]
240, 97, Mooney, M, 2009	27	CM, Placebo, Psychotherapy	0.50 [0.13, 1.95]
243, 98, Mooney, M, 2015	21	Placebo, Psychotherapy	0.12 [0.01, 1.15]
245, 99, Mooney, M, 2007	36	Placebo, Psychotherapy	0.31 [0.10, 0.95]
246, 99, Mooney, M, 2007	40	Placebo, Psychotherapy	0.47 [0.15, 1.41]

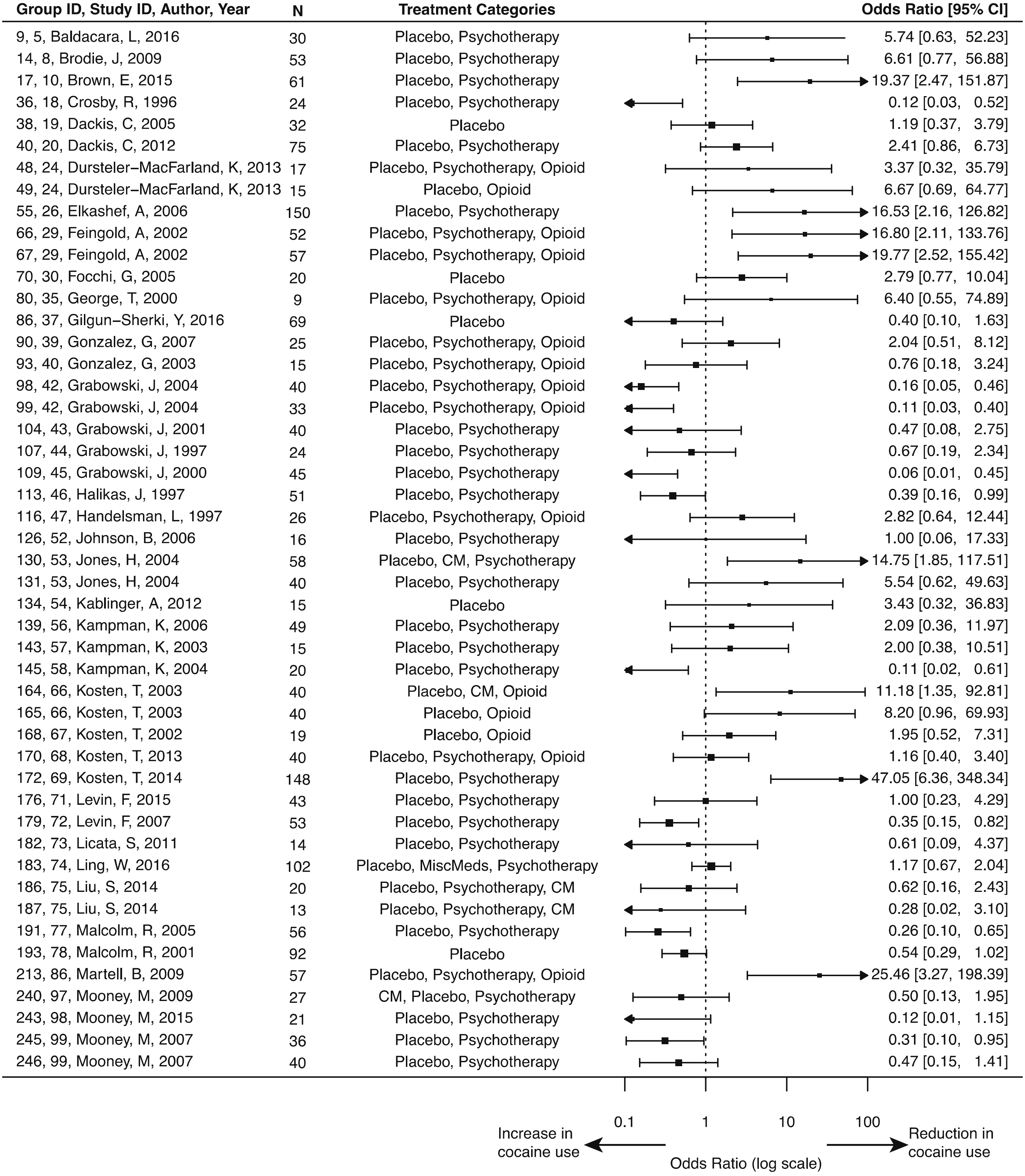

© 2021 Bentzley BS et al. *JAMA Network Open*.

Placebo (2 of 2)

Group ID, Study ID, Author, Year	N	Treatment Categories	Odds Ratio [95% CI]
246, 99, Mooney, M, 2007	40	Placebo, Psychotherapy	0.47 [0.15, 1.41]
253, 102, Oliveto, A, 1999	34	Placebo, Psychotherapy, Opioid	10.00 [1.18, 84.78]
254, 102, Oliveto, A, 1999	31	Placebo, Psychotherapy, Opioid	10.24 [1.20, 87.35]
255, 102, Oliveto, A, 1999	11	Placebo, Psychotherapy, Opioid	2.18 [0.17, 27.56]
256, 102, Oliveto, A, 1999	14	Placebo, Psychotherapy, Opioid	6.82 [0.69, 66.90]
265, 104, Oliveto, A, 2011	38	Placebo, Psychotherapy, Opioid	2.46 [0.81, 7.46]
292, 114, Plebani, J, 2012	19	Placebo	1.26 [0.33, 4.84]
294, 115, Poling, J, 2006	25	Placebo, CM, Psychotherapy, Opioid	10.95 [1.26, 95.06]
295, 115, Poling, J, 2006	24	Placebo, Psychotherapy, Opioid	13.24 [1.53, 114.30]
298, 116, Poling, J, 2010	18	Placebo, Psychotherapy, Opioid	10.23 [1.12, 93.34]
310, 121, Reid, M, 2005	13	Placebo, Psychotherapy	5.09 [0.50, 52.29]
312, 122, Reid, M, 2005	15	Placebo, Psychotherapy	1.00 [0.06, 17.41]
325, 128, Schmitz, J, 2001	34	Placebo, Psychotherapy	0.10 [0.02, 0.49]
327, 129, Schmitz, J, 2008	27	Placebo	0.55 [0.12, 2.58]
328, 129, Schmitz, J, 2008	31	Placebo, Psychotherapy	0.14 [0.02, 1.24]
329, 129, Schmitz, J, 2008	27	Placebo, Psychotherapy, CM	0.17 [0.02, 1.57]
341, 132, Schubiner, H, 2002	24	Placebo, Psychotherapy	9.21 [1.04, 81.36]
347, 135, Shearer, J, 2003	14	Placebo, Opioid	1.00 [0.16, 6.08]
349, 136, Shoptaw, S, 2002	35	Placebo, Psychotherapy	0.07 [0.02, 0.27]
351, 137, Shoptaw, S, 2008	33	Placebo, Psychotherapy	0.13 [0.04, 0.45]
353, 138, Shoptaw, S, 2004	12	Placebo, Psychotherapy	7.00 [1.04, 46.95]
355, 139, Shoptaw, S, 2005	15	Placebo, Psychotherapy	2.13 [0.17, 26.03]
359, 140, Shoptaw, S, 2003	35	Placebo, Psychotherapy	0.16 [0.05, 0.47]
361, 141, Shorter, D, 2013	13	Placebo, Psychotherapy	2.15 [0.17, 26.67]
381, 149, Sofuoglu, M, 2017	34	Placebo, Psychotherapy, Opioid	17.50 [2.13, 143.64]
384, 150, Sofuoglu, M, 2007	15	Placebo, Psychotherapy, Opioid	8.73 [0.92, 82.96]
386, 151, Somoza, E, 2013	94	Placebo, Psychotherapy	6.33 [0.75, 53.64]
388, 152, Tapp, A, 2015	31	Placebo, Psychotherapy	0.13 [0.03, 0.52]
394, 154, Walsh, S, 2013	25	Placebo	0.29 [0.08, 1.09]
396, 155, Wardle, M, 2017	40	Placebo, CM, Psychotherapy	1.89 [0.61, 5.82]
401, 157, Winhusen, T, 2007	71	Placebo, Psychotherapy	0.54 [0.20, 1.46]
RE Model for All Studies (N = 2889, Q = 292.56, df = 77, p = 7.34e−27; I^2 = 78.4%)			1.33 [0.92, 1.92]

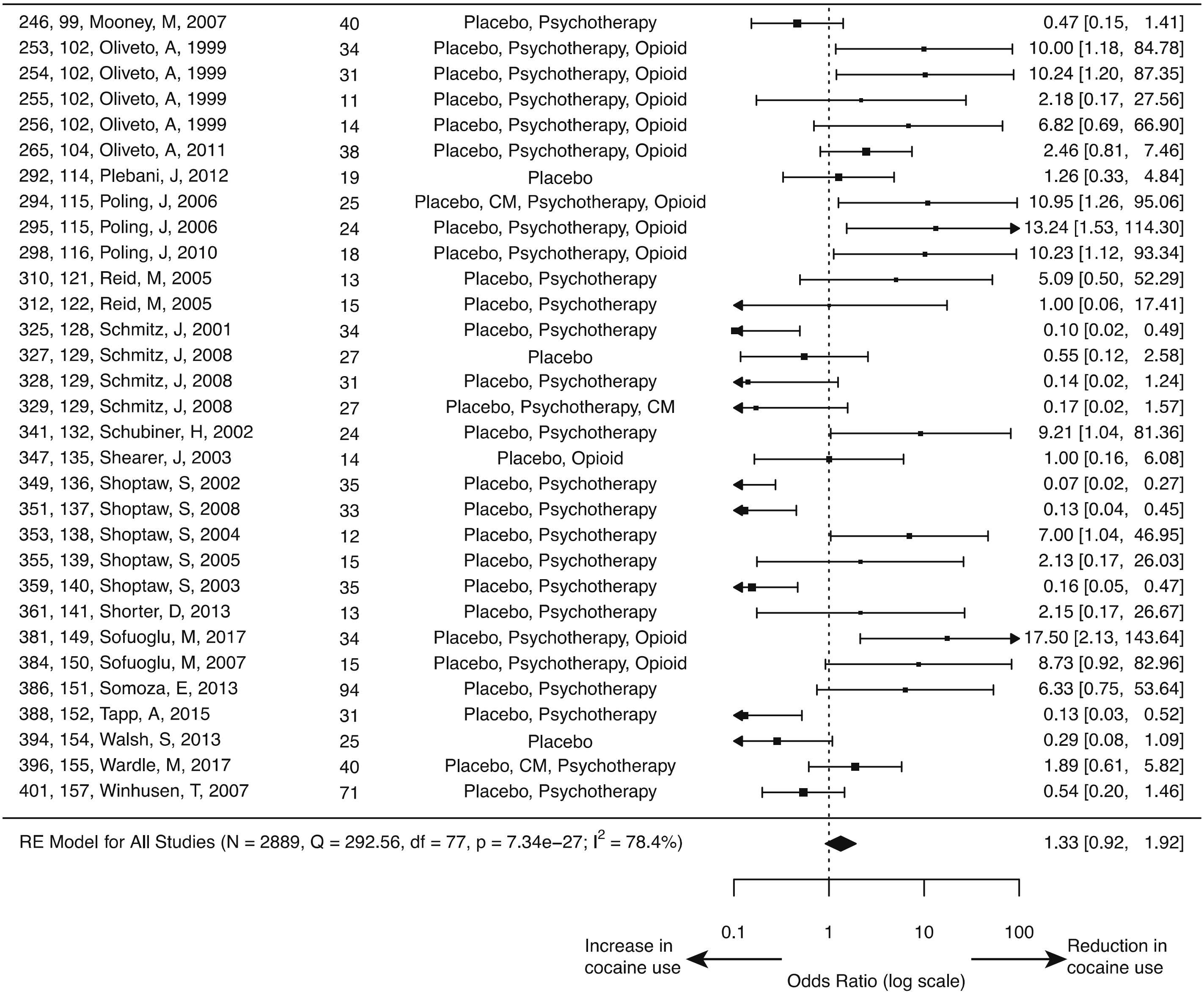

© 2021 Bentzley BS et al. *JAMA Network Open*.

eFigure 10. Forest Plot Without Imputed Data: Psychostimulants

Group ID, Study ID, Author, Year	N	Treatment Categories	Odds Ratio [95% CI]
39, 19, Dackis, C, 2005	30	Psychostimulants	1.32 [0.47, 3.72]
41, 20, Dackis, C, 2012	70	Psychostimulants, Psychotherapy	29.24 [3.81, 224.41]
42, 20, Dackis, C, 2012	65	Psychostimulants, Psychotherapy	14.40 [1.82, 114.25]
50, 24, Dursteler–MacFarland, K, 2013	15	Psychostimulants, Psychotherapy, Opioid	3.43 [0.32, 36.83]
51, 24, Dursteler–MacFarland, K, 2013	15	Psychostimulants, Opioid	4.92 [0.49, 49.61]
100, 42, Grabowski, J, 2004	26	Psychostimulants, Psychotherapy, Opioid	1.00 [0.28, 3.63]
101, 42, Grabowski, J, 2004	28	Psychostimulants, Psychotherapy, Opioid	0.72 [0.23, 2.22]
105, 43, Grabowski, J, 2001	42	Psychostimulants, Psychotherapy	0.50 [0.17, 1.43]
106, 43, Grabowski, J, 2001	46	Psychostimulants, Psychotherapy	0.16 [0.04, 0.60]
108, 44, Grabowski, J, 1997	25	Psychostimulants, Psychotherapy	0.35 [0.08, 1.55]
177, 71, Levin, F, 2015	43	Psychostimulants, Psychotherapy	3.65 [1.26, 10.56]
178, 71, Levin, F, 2015	40	Psychostimulants, Psychotherapy	2.33 [0.72, 7.59]
180, 72, Levin, F, 2007	53	Psychostimulants, Psychotherapy	0.39 [0.17, 0.90]
241, 97, Mooney, M, 2009	30	Psychostimulants, CM, Psychotherapy	0.26 [0.06, 1.08]
242, 97, Mooney, M, 2009	25	Psychostimulants, CM, Psychotherapy	1.00 [0.29, 3.44]
244, 98, Mooney, M, 2015	22	Psychostimulants, Psychotherapy	0.16 [0.02, 1.52]
251, 101, Nuijten, M, 2015	30	Psychostimulants, Psychotherapy	4.26 [0.81, 22.53]
342, 132, Schubiner, H, 2002	24	Psychostimulants, Psychotherapy	7.50 [0.83, 67.49]
348, 135, Shearer, J, 2003	16	Psychostimulants, Opioid	11.67 [1.23, 110.95]
RE Model for All Studies (N = 645, Q = 60.46, df = 18, p = 1.73e–06; I^2 = 74.1%)			1.39 [0.73, 2.65]

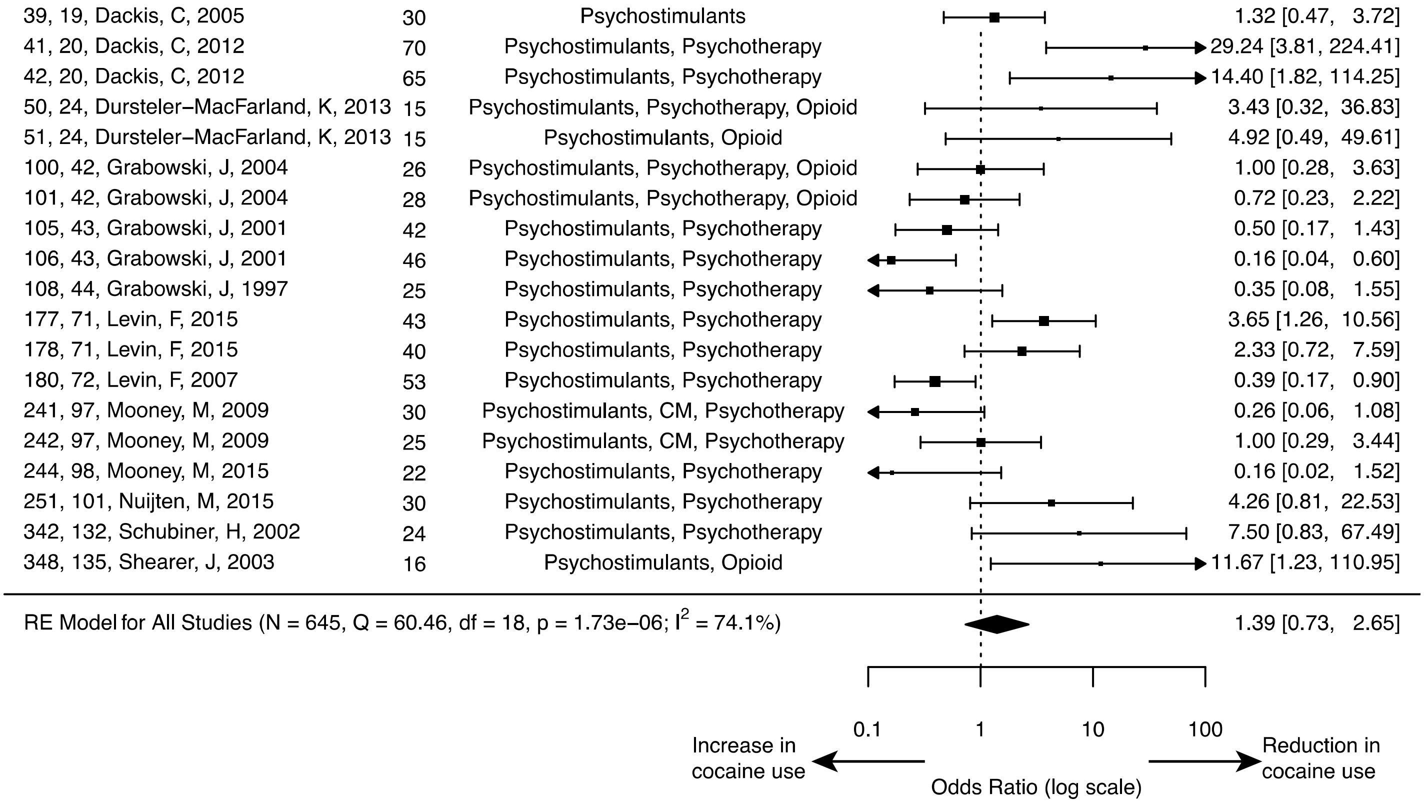

© 2021 Bentzley BS et al. *JAMA Network Open*.

eFigure 11. Forest Plot Without Imputed Data: Psychotherapy

(1 of 6)

Group ID, Study ID, Author, Year	N	Treatment Categories	Odds Ratio [95% CI]
3, 2, Avants, S, 1998	6	Antidepressants, Psychotherapy, Opioid	7.00 [0.57, 86.32]
9, 5, Baldacara, L, 2016	30	Placebo, Psychotherapy	5.74 [0.63, 52.23]
10, 5, Baldacara, L, 2016	30	Anticonvulsants, Psychotherapy	59.18 [7.10, 493.42]
13, 7, Bernstein, J, 2005	376	Psychotherapy	87.19 [12.04, 631.15]
14, 8, Brodie, J, 2009	53	Placebo, Psychotherapy	6.61 [0.77, 56.88]
15, 8, Brodie, J, 2009	50	Anticonvulsants, Psychotherapy	27.00 [3.44, 211.82]
17, 10, Brown, E, 2015	61	Placebo, Psychotherapy	19.37 [2.47, 151.87]
18, 10, Brown, E, 2015	61	MiscMeds, Psychotherapy	22.91 [2.94, 178.44]
32, 16, Covi, L, 2002	26	Psychotherapy	0.49 [0.12, 1.95]
33, 16, Covi, L, 2002	21	Psychotherapy	0.23 [0.06, 0.88]
34, 16, Covi, L, 2002	21	Psychotherapy	0.63 [0.16, 2.42]
36, 18, Crosby, R, 1996	24	Placebo, Psychotherapy	0.12 [0.03, 0.52]
37, 18, Crosby, R, 1996	20	Anticonvulsants, Psychotherapy	0.20 [0.05, 0.83]
40, 20, Dackis, C, 2012	75	Placebo, Psychotherapy	2.41 [0.86, 6.73]
41, 20, Dackis, C, 2012	70	Psychostimulants, Psychotherapy	29.24 [3.81, 224.41]
42, 20, Dackis, C, 2012	65	Psychostimulants, Psychotherapy	14.40 [1.82, 114.25]
43, 21, Dallery, J, 2001	15	CM, Psychotherapy, Opioid	3.50 [0.32, 38.23]
48, 24, Dursteler−MacFarland, K, 2013	17	Placebo, Psychotherapy, Opioid	3.37 [0.32, 35.79]
50, 24, Dursteler−MacFarland, K, 2013	15	Psychostimulants, Psychotherapy, Opioid	3.43 [0.32, 36.83]
54, 26, Elkashef, A, 2006	150	MiscMeds, Psychotherapy	11.78 [1.50, 92.42]
55, 26, Elkashef, A, 2006	150	Placebo, Psychotherapy	16.53 [2.16, 126.82]
56, 27, Epstein, D, 2003	49	CM, Psychotherapy, Opioid	6.76 [1.81, 25.23]
59, 27, Epstein, D, 2003	48	Psychotherapy, Opioid	1.47 [0.43, 5.00]
62, 28, Epstein, D, 2009	49	CM, Psychotherapy, Opioid	2.91 [1.07, 7.89]
63, 28, Epstein, D, 2009	47	CM, Psychotherapy, Opioid	2.34 [0.80, 6.89]
64, 28, Epstein, D, 2009	48	CM, Psychotherapy, Opioid	2.66 [0.97, 7.29]
65, 28, Epstein, D, 2009	47	CM, Psychotherapy, Opioid	2.61 [0.90, 7.61]
66, 29, Feingold, A, 2002	52	Placebo, Psychotherapy, Opioid	16.80 [2.11, 133.76]
67, 29, Feingold, A, 2002	57	Placebo, Psychotherapy, Opioid	19.77 [2.52, 155.42]
68, 29, Feingold, A, 2002	52	Antidepressants, Psychotherapy, Opioid	18.55 [2.34, 146.99]
69, 29, Feingold, A, 2002	57	Antidepressants, Psychotherapy, Opioid	23.48 [3.01, 183.37]
80, 35, George, T, 2000	9	Placebo, Psychotherapy, Opioid	6.40 [0.55, 74.89]
81, 35, George, T, 2000	11	MiscMeds, Psychotherapy, Opioid	2.57 [0.36, 18.33]
82, 36, Ghitza, U, 2007	40	Opioid, Psychotherapy	3.15 [0.31, 31.62]
83, 36, Ghitza, U, 2007	20	CM, Opioid, Psychotherapy	2.10 [0.18, 25.01]
84, 36, Ghitza, U, 2007	36	CM, Opioid, Psychotherapy	6.94 [0.79, 60.71]
85, 36, Ghitza, U, 2007	20	CM, Opioid, Psychotherapy	6.18 [0.66, 58.03]
90, 39, Gonzalez, G, 2007	25	Placebo, Psychotherapy, Opioid	2.04 [0.51, 8.12]
91, 39, Gonzalez, G, 2007	26	Anticonvulsants, Psychotherapy, Opioid	1.00 [0.18, 5.48]
92, 39, Gonzalez, G, 2007	25	Anticonvulsants, Psychotherapy, Opioid	2.85 [0.64, 12.64]
93, 40, Gonzalez, G, 2003	15	Placebo, Psychotherapy, Opioid	0.76 [0.18, 3.24]
94, 40, Gonzalez, G, 2003	15	Anticonvulsants, Psychotherapy, Opioid	1.00 [0.22, 4.56]
95, 40, Gonzalez, G, 2003	15	Anticonvulsants, Psychotherapy, Opioid	1.00 [0.22, 4.56]
98, 42, Grabowski, J, 2004	40	Placebo, Psychotherapy, Opioid	0.16 [0.05, 0.46]
99, 42, Grabowski, J, 2004	33	Placebo, Psychotherapy, Opioid	0.11 [0.03, 0.40]
100, 42, Grabowski, J, 2004	26	Psychostimulants, Psychotherapy, Opioid	1.00 [0.28, 3.63]
101, 42, Grabowski, J, 2004	28	Psychostimulants, Psychotherapy, Opioid	0.72 [0.23, 2.22]
102, 42, Grabowski, J, 2004	32	Antipsychotics, Psychotherapy, Opioid	0.16 [0.05, 0.53]
103, 42, Grabowski, J, 2004	31	Antipsychotics, Psychotherapy, Opioid	0.66 [0.18, 2.36]

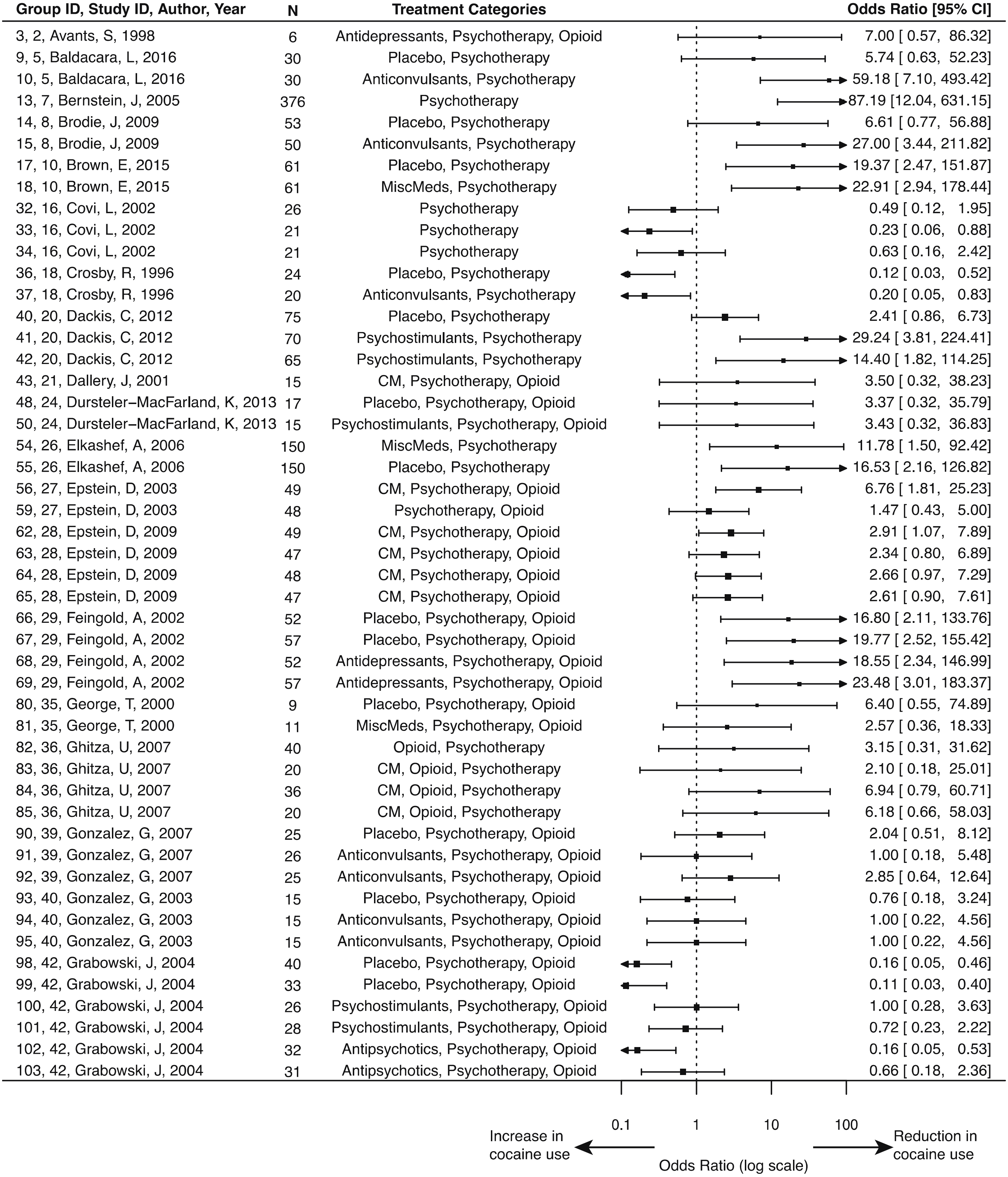

© 2021 Bentzley BS et al. *JAMA Network Open*.

Psychotherapy (2 of 6)

Group ID, Study ID, Author, Year	N	Treatment Categories	Odds Ratio [95% CI]
104, 43, Grabowski, J, 2001	40	Placebo, Psychotherapy	0.47 [0.08, 2.75]
105, 43, Grabowski, J, 2001	42	Psychostimulants, Psychotherapy	0.50 [0.17, 1.43]
106, 43, Grabowski, J, 2001	46	Psychostimulants, Psychotherapy	0.16 [0.04, 0.60]
107, 44, Grabowski, J, 1997	24	Placebo, Psychotherapy	0.67 [0.19, 2.34]
108, 44, Grabowski, J, 1997	25	Psychostimulants, Psychotherapy	0.35 [0.08, 1.55]
109, 45, Grabowski, J, 2000	45	Placebo, Psychotherapy	0.06 [0.01, 0.45]
110, 45, Grabowski, J, 2000	30	Antipsychotics, Psychotherapy	0.37 [0.08, 1.58]
111, 45, Grabowski, J, 2000	38	Antipsychotics, Psychotherapy	0.05 [0.01, 0.42]
112, 45, Grabowski, J, 2000	12	Antipsychotics, Psychotherapy	0.19 [0.02, 2.00]
113, 46, Halikas, J, 1997	51	Placebo, Psychotherapy	0.39 [0.16, 0.99]
114, 46, Halikas, J, 1997	49	Anticonvulsants, Psychotherapy	0.69 [0.29, 1.61]
115, 46, Halikas, J, 1997	50	Anticonvulsants, Psychotherapy	0.13 [0.05, 0.35]
116, 47, Handelsman, L, 1997	26	Placebo, Psychotherapy, Opioid	2.82 [0.64, 12.44]
117, 47, Handelsman, L, 1997	24	DA, Psychotherapy, Opioid	0.40 [0.10, 1.57]
121, 50, Higgins, S, 2003	49	CM, Psychotherapy	3.91 [1.63, 9.37]
126, 52, Johnson, B, 2006	16	Placebo, Psychotherapy	1.00 [0.06, 17.33]
127, 52, Johnson, B, 2006	16	MiscMeds, Psychotherapy	0.43 [0.07, 2.76]
128, 52, Johnson, B, 2006	12	MiscMeds, Psychotherapy	0.14 [0.01, 1.39]
129, 52, Johnson, B, 2006	19	MiscMeds, Psychotherapy	4.80 [0.48, 47.68]
130, 53, Jones, H, 2004	58	Placebo, CM, Psychotherapy	14.75 [1.85, 117.51]
131, 53, Jones, H, 2004	40	Placebo, Psychotherapy	5.54 [0.62, 49.63]
132, 53, Jones, H, 2004	45	MiscMeds, CM, Psychotherapy	9.44 [1.13, 78.78]
133, 53, Jones, H, 2004	56	MiscMeds, Psychotherapy	5.38 [0.61, 47.54]
139, 56, Kampman, K, 2006	49	Placebo, Psychotherapy	2.09 [0.36, 11.97]
140, 56, Kampman, K, 2006	50	DA, MiscMeds, Psychotherapy	5.43 [0.61, 48.15]
141, 56, Kampman, K, 2006	50	DA, Psychotherapy	7.93 [0.94, 66.98]
142, 56, Kampman, K, 2006	50	MiscMeds, Psychotherapy	6.77 [1.42, 32.37]
143, 57, Kampman, K, 2003	15	Placebo, Psychotherapy	2.00 [0.38, 10.51]
144, 57, Kampman, K, 2003	15	Antipsychotics, Psychotherapy	0.29 [0.03, 3.12]
145, 58, Kampman, K, 2004	20	Placebo, Psychotherapy	0.11 [0.02, 0.61]
146, 58, Kampman, K, 2004	20	Anticonvulsants, Psychotherapy	1.22 [0.35, 4.24]
154, 62, Katz, E, 2002	40	CM, Psychotherapy, Opioid	22.78 [2.84, 182.64]
155, 62, Katz, E, 2002	40	Psychotherapy, Opioid	12.81 [1.56, 105.36]
158, 64, Kirby, K, 2013	62	CM, Opioid, Psychotherapy	22.79 [2.93, 177.34]
159, 64, Kirby, K, 2013	68	CM, Opioid, Psychotherapy	36.00 [4.71, 275.45]
170, 68, Kosten, T, 2013	40	Placebo, Psychotherapy, Opioid	1.16 [0.40, 3.40]
171, 68, Kosten, T, 2013	34	MiscMeds, Psychotherapy, Opioid	1.61 [0.53, 4.88]
172, 69, Kosten, T, 2014	148	Placebo, Psychotherapy	47.05 [6.36, 348.34]
173, 69, Kosten, T, 2014	152	Other, Psychotherapy	45.00 [6.08, 333.22]
176, 71, Levin, F, 2015	43	Placebo, Psychotherapy	1.00 [0.23, 4.29]
177, 71, Levin, F, 2015	43	Psychostimulants, Psychotherapy	3.65 [1.26, 10.56]
178, 71, Levin, F, 2015	40	Psychostimulants, Psychotherapy	2.33 [0.72, 7.59]
179, 72, Levin, F, 2007	53	Placebo, Psychotherapy	0.35 [0.15, 0.82]
180, 72, Levin, F, 2007	53	Psychostimulants, Psychotherapy	0.39 [0.17, 0.90]
181, 73, Licata, S, 2011	15	MiscMeds, Psychotherapy	0.46 [0.04, 5.75]
182, 73, Licata, S, 2011	14	Placebo, Psychotherapy	0.61 [0.09, 4.37]
183, 74, Ling, W, 2016	102	Placebo, MiscMeds, Psychotherapy	1.17 [0.67, 2.04]
184, 74, Ling, W, 2016	100	Opioid, MiscMeds, Psychotherapy	1.04 [0.60, 1.81]
185, 74, Ling, W, 2016	100	Opioid, MiscMeds, Psychotherapy	1.85 [1.05, 3.26]

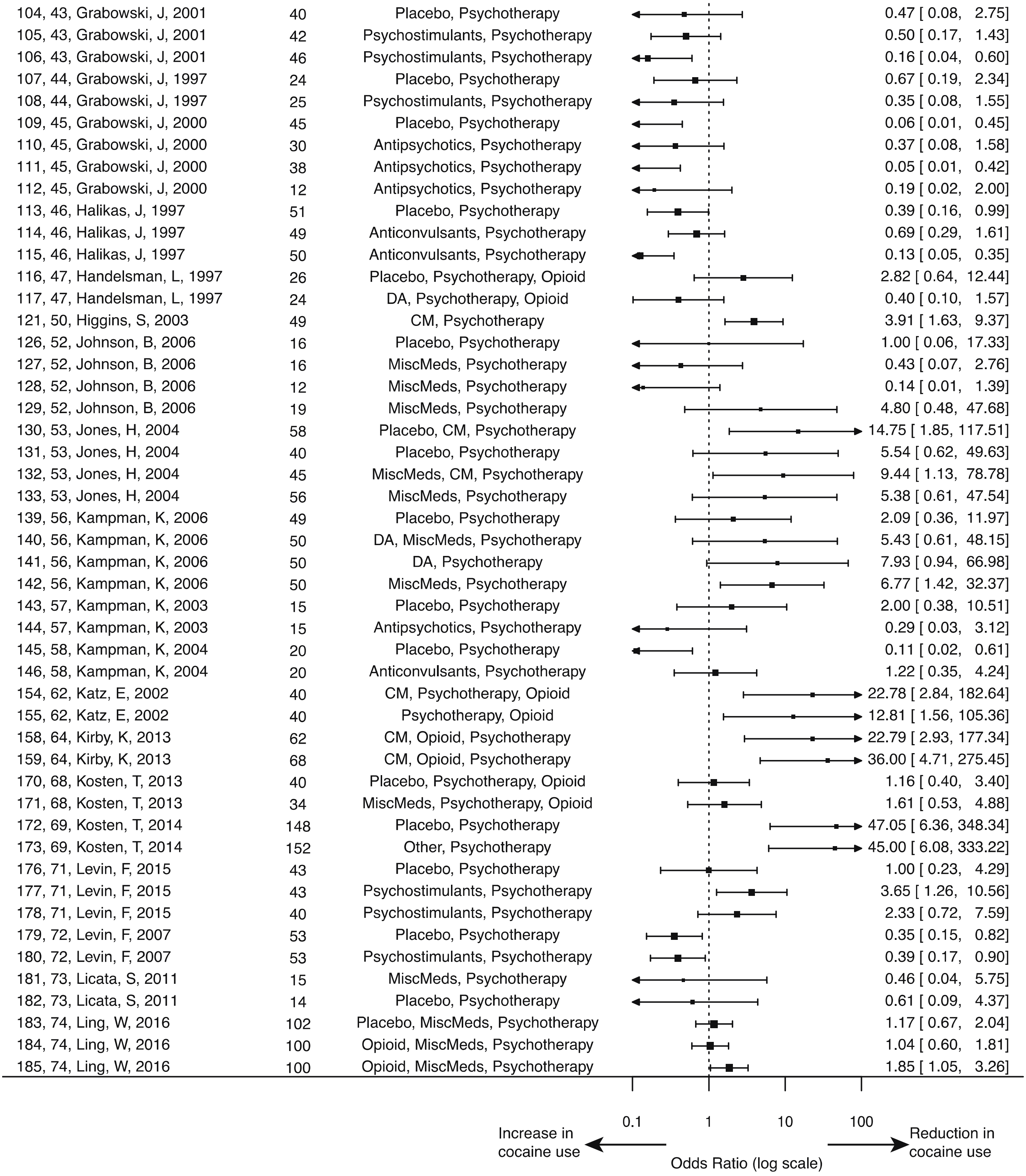

© 2021 Bentzley BS et al. *JAMA Network Open*.

Psychotherapy (3 of 6)

Group ID, Study ID, Author, Year	N	Treatment Categories	Odds Ratio [95% CI]
186, 75, Liu, S, 2014	20	Placebo, Psychotherapy, CM	0.62 [0.16, 2.43]
187, 75, Liu, S, 2014	13	Placebo, Psychotherapy, CM	0.28 [0.02, 3.10]
188, 75, Liu, S, 2014	26	DA, Psychotherapy, CM	0.68 [0.20, 2.32]
189, 75, Liu, S, 2014	12	DA, Psychotherapy, CM	0.60 [0.08, 4.45]
190, 76, Magura, S, 1994	43	Psychotherapy, Opioid	2.70 [0.49, 14.74]
191, 77, Malcolm, R, 2005	56	Placebo, Psychotherapy	0.26 [0.10, 0.65]
192, 77, Malcolm, R, 2005	60	MiscMeds, Psychotherapy	0.20 [0.09, 0.47]
196, 79, Mancino, M, 2014	34	Antidepressants, Anticonvulsants, Psychotherapy	10.00 [1.18, 84.78]
197, 79, Mancino, M, 2014	35	Antidepressants, Psychotherapy	9.93 [1.17, 84.04]
198, 79, Mancino, M, 2014	38	Psychotherapy	5.57 [0.62, 50.03]
204, 82, Margolin, A, 2002	222	Other, Psychotherapy	0.44 [0.27, 0.71]
205, 82, Margolin, A, 2002	203	Other, Psychotherapy	0.56 [0.35, 0.89]
206, 82, Margolin, A, 2002	195	Other, Psychotherapy	0.51 [0.31, 0.83]
211, 85, Martell, B, 2005	8	Other, Psychotherapy	11.67 [0.92, 147.56]
212, 85, Martell, B, 2005	10	Other, Psychotherapy	1.00 [0.17, 5.98]
213, 86, Martell, B, 2009	57	Placebo, Psychotherapy, Opioid	25.46 [3.27, 198.39]
214, 86, Martell, B, 2009	57	Other, Psychotherapy, Opioid	23.48 [3.01, 183.37]
216, 87, Maude−Griffin, P, 1998	59	Psychotherapy	4.07 [1.64, 10.12]
224, 90, McKee, S, 2007	31	Psychotherapy	0.59 [0.21, 1.62]
225, 90, McKee, S, 2007	35	Psychotherapy, Psychotherapy	0.34 [0.13, 0.91]
226, 91, Miguel, A, 2016	32	Psychotherapy	0.09 [0.02, 0.36]
227, 91, Miguel, A, 2016	33	Psychotherapy, CM	0.89 [0.34, 2.33]
228, 92, Milby, J, 2000	56	CM, Psychotherapy	1.95 [0.91, 4.17]
229, 92, Milby, J, 2000	54	Psychotherapy	0.28 [0.12, 0.64]
230, 93, Milby, J, 2008	103	CM, Psychotherapy	1.24 [0.65, 2.34]
236, 96, Montoya, I, 2004	46	Opioid, Psychotherapy	13.97 [1.72, 113.19]
237, 96, Montoya, I, 2004	44	Opioid, Psychotherapy	10.95 [1.33, 90.40]
238, 96, Montoya, I, 2004	43	Opioid, Psychotherapy	17.88 [2.22, 143.70]
239, 96, Montoya, I, 2004	46	Opioid, Psychotherapy	21.36 [2.69, 169.74]
240, 97, Mooney, M, 2009	27	CM, Placebo, Psychotherapy	0.50 [0.13, 1.95]
241, 97, Mooney, M, 2009	30	Psychostimulants, CM, Psychotherapy	0.26 [0.06, 1.08]
242, 97, Mooney, M, 2009	25	Psychostimulants, CM, Psychotherapy	1.00 [0.29, 3.44]
243, 98, Mooney, M, 2015	21	Placebo, Psychotherapy	0.12 [0.01, 1.15]
244, 98, Mooney, M, 2015	22	Psychostimulants, Psychotherapy	0.16 [0.02, 1.52]
245, 99, Mooney, M, 2007	36	Placebo, Psychotherapy	0.31 [0.10, 0.95]
246, 99, Mooney, M, 2007	40	Placebo, Psychotherapy	0.47 [0.15, 1.41]
247, 99, Mooney, M, 2007	31	DA, Psychotherapy	0.50 [0.16, 1.62]
248, 99, Mooney, M, 2007	43	DA, Psychotherapy	0.34 [0.11, 0.98]
249, 99, Mooney, M, 2007	39	DA, Psychotherapy	0.37 [0.12, 1.21]
251, 101, Nuijten, M, 2015	30	Psychostimulants, Psychotherapy	4.26 [0.81, 22.53]
252, 101, Nuijten, M, 2015	35	Psychotherapy	1.17 [0.39, 3.49]
253, 102, Oliveto, A, 1999	34	Placebo, Psychotherapy, Opioid	10.00 [1.18, 84.78]
254, 102, Oliveto, A, 1999	31	Placebo, Psychotherapy, Opioid	10.24 [1.20, 87.35]
255, 102, Oliveto, A, 1999	11	Placebo, Psychotherapy, Opioid	2.18 [0.17, 27.56]
256, 102, Oliveto, A, 1999	14	Placebo, Psychotherapy, Opioid	6.82 [0.69, 66.90]
257, 102, Oliveto, A, 1999	35	Antidepressants, Psychotherapy, Opioid	24.55 [3.03, 198.96]
258, 102, Oliveto, A, 1999	24	Antidepressants, Psychotherapy, Opioid	15.63 [1.82, 134.04]
259, 102, Oliveto, A, 1999	10	Antidepressants, Psychotherapy, Opioid	2.20 [0.17, 28.14]
260, 102, Oliveto, A, 1999	21	Antidepressants, Psychotherapy, Opioid	6.11 [0.65, 57.15]

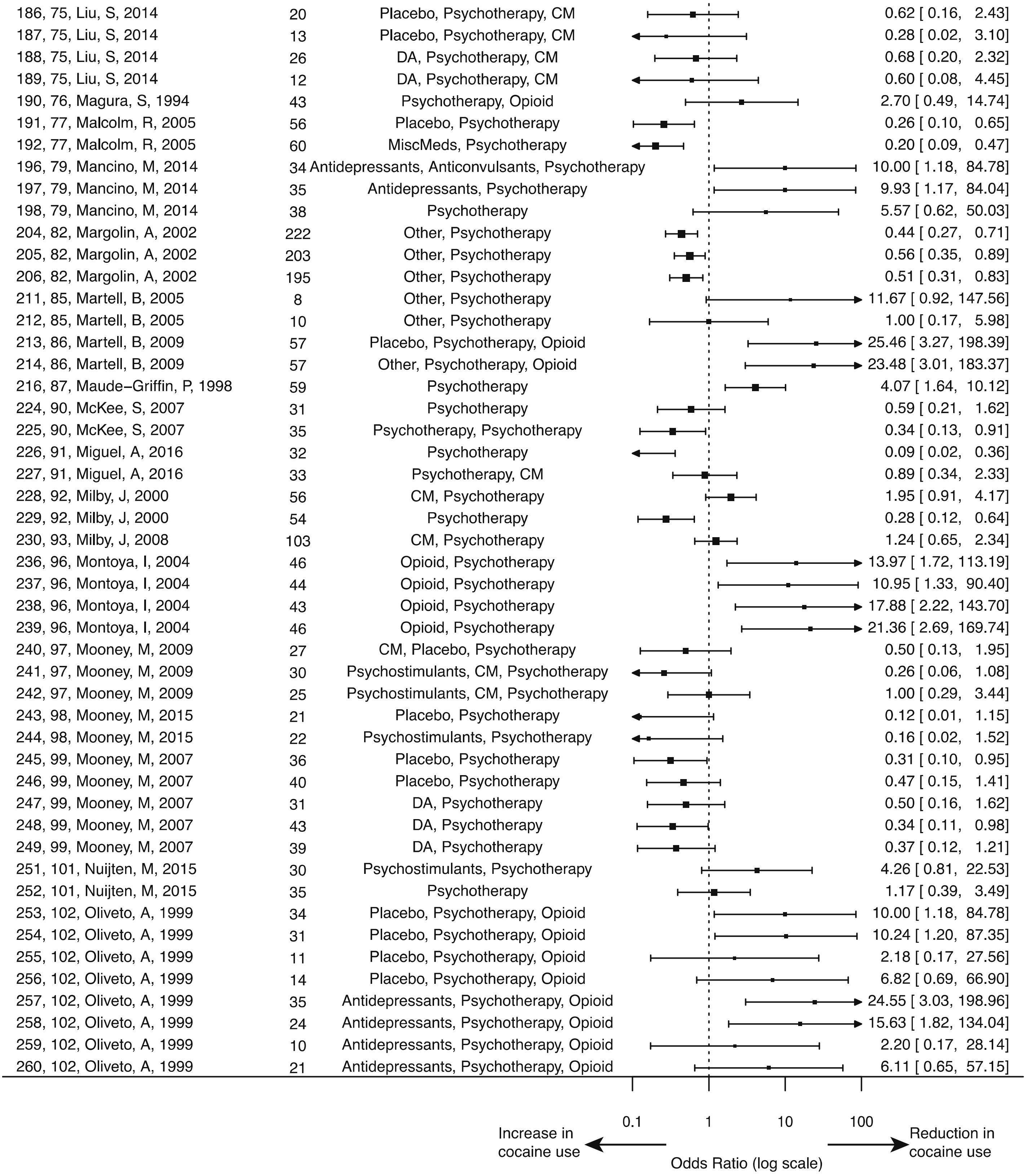

© 2021 Bentzley BS et al. *JAMA Network Open*.

Psychotherapy (4 of 6)

Group ID, Study ID, Author, Year	N	Treatment Categories	Odds Ratio [95% CI]
261, 103, Oliveto, A, 2005	35	CM, Psychotherapy, Opioid	11.57 [1.38, 96.81]
262, 103, Oliveto, A, 2005	35	CM, Psychotherapy, Opioid	17.28 [2.11, 141.51]
263, 103, Oliveto, A, 2005	35	Opioid, Psychotherapy	13.33 [1.61, 110.56]
264, 103, Oliveto, A, 2005	35	Opioid, Psychotherapy	11.57 [1.38, 96.81]
265, 104, Oliveto, A, 2011	38	Placebo, Psychotherapy, Opioid	2.46 [0.81, 7.46]
266, 104, Oliveto, A, 2011	37	MiscMeds, Psychotherapy, Opioid	1.18 [0.38, 3.68]
267, 104, Oliveto, A, 2011	38	MiscMeds, Psychotherapy, Opioid	1.24 [0.34, 4.46]
268, 104, Oliveto, A, 2011	39	MiscMeds, Psychotherapy, Opioid	2.16 [0.71, 6.59]
269, 105, Petitjean, S, 2014	29	CM, Psychotherapy	2.45 [0.82, 7.31]
270, 105, Petitjean, S, 2014	31	Psychotherapy	0.75 [0.27, 2.14]
273, 107, Petry, N, 2012	147	CM, Psychotherapy	0.42 [0.26, 0.69]
274, 107, Petry, N, 2012	153	CM, Psychotherapy	0.35 [0.21, 0.58]
275, 107, Petry, N, 2012	142	Psychotherapy	0.23 [0.14, 0.38]
279, 109, Petry, N, 2002	19	CM, Psychotherapy, Opioid	1.54 [0.42, 5.64]
280, 109, Petry, N, 2002	23	Psychotherapy, Opioid	0.34 [0.10, 1.14]
281, 110, Petry, N, 2005	40	CM, Psychotherapy, Opioid	2.22 [0.86, 5.74]
282, 110, Petry, N, 2005	37	Psychotherapy, Opioid	0.44 [0.12, 1.61]
283, 111, Petry, N, 2004	37	Psychotherapy	0.05 [0.01, 0.19]
284, 111, Petry, N, 2004	45	CM, Psychotherapy	0.11 [0.04, 0.32]
285, 111, Petry, N, 2004	38	CM, Psychotherapy	0.19 [0.07, 0.50]
286, 112, Petry, N, 2007	28	CM, Psychotherapy, Opioid	0.75 [0.26, 2.15]
287, 112, Petry, N, 2007	27	CM, Psychotherapy, Opioid	1.16 [0.40, 3.37]
288, 112, Petry, N, 2007	19	Psychotherapy, Opioid	0.61 [0.15, 2.44]
294, 115, Poling, J, 2006	25	Placebo, CM, Psychotherapy, Opioid	10.95 [1.26, 95.06]
295, 115, Poling, J, 2006	24	Placebo, Psychotherapy, Opioid	13.24 [1.53, 114.30]
296, 115, Poling, J, 2006	27	Antidepressants, CM, Psychotherapy, Opioid	34.46 [4.12, 288.45]
297, 115, Poling, J, 2006	30	Antidepressants, Psychotherapy, Opioid	14.09 [1.68, 118.22]
298, 116, Poling, J, 2010	18	Placebo, Psychotherapy, Opioid	10.23 [1.12, 93.34]
299, 116, Poling, J, 2010	13	MiscMeds, Psychotherapy, Opioid	9.33 [0.96, 90.94]
300, 117, Preston, K, 2008	29	CM, Psychotherapy, Opioid	1.25 [0.34, 4.68]
301, 117, Preston, K, 2008	38	CM, Psychotherapy, Opioid	1.92 [0.51, 7.19]
302, 118, Preston, K, 2001	49	CM, Psychotherapy, Opioid	2.55 [0.81, 7.99]
303, 118, Preston, K, 2001	46	CM, Psychotherapy, Opioid	2.44 [0.88, 6.76]
306, 120, Rawson, R, 2002	27	CM, Psychotherapy, Opioid	39.67 [4.73, 332.84]
307, 120, Rawson, R, 2002	26	CM, Psychotherapy, Opioid	23.40 [2.78, 196.84]
308, 120, Rawson, R, 2002	28	Psychotherapy, Opioid	19.33 [2.31, 161.57]
309, 120, Rawson, R, 2002	27	Psychotherapy, Opioid	8.91 [1.02, 77.91]
310, 121, Reid, M, 2005	13	Placebo, Psychotherapy	5.09 [0.50, 52.29]
311, 121, Reid, M, 2005	10	MiscMeds, Psychotherapy	2.20 [0.17, 28.14]
312, 122, Reid, M, 2005	15	Placebo, Psychotherapy	1.00 [0.06, 17.41]
313, 122, Reid, M, 2005	15	MiscMeds, MiscMeds, Psychotherapy	2.13 [0.17, 26.03]
314, 122, Reid, M, 2005	16	Antipsychotics, Psychotherapy	2.12 [0.18, 25.78]
315, 122, Reid, M, 2005	17	Anticonvulsants, Psychotherapy	4.80 [0.48, 47.68]
317, 124, Rosenblum, A, 1999	140	Psychotherapy, Opioid	1.59 [0.96, 2.63]
318, 124, Rosenblum, A, 1999	58	Psychotherapy, Opioid	1.36 [0.63, 2.93]
319, 125, Rowan−Szal, G, 2005	13	CM, Psychotherapy, Opioid	3.89 [0.72, 21.06]
320, 125, Rowan−Szal, G, 2005	17	CM, Psychotherapy, Opioid	6.67 [1.38, 32.28]
321, 125, Rowan−Szal, G, 2005	15	Psychotherapy, Opioid	1.00 [0.20, 5.04]
322, 125, Rowan−Szal, G, 2005	16	Psychotherapy, Opioid	4.33 [0.88, 21.30]

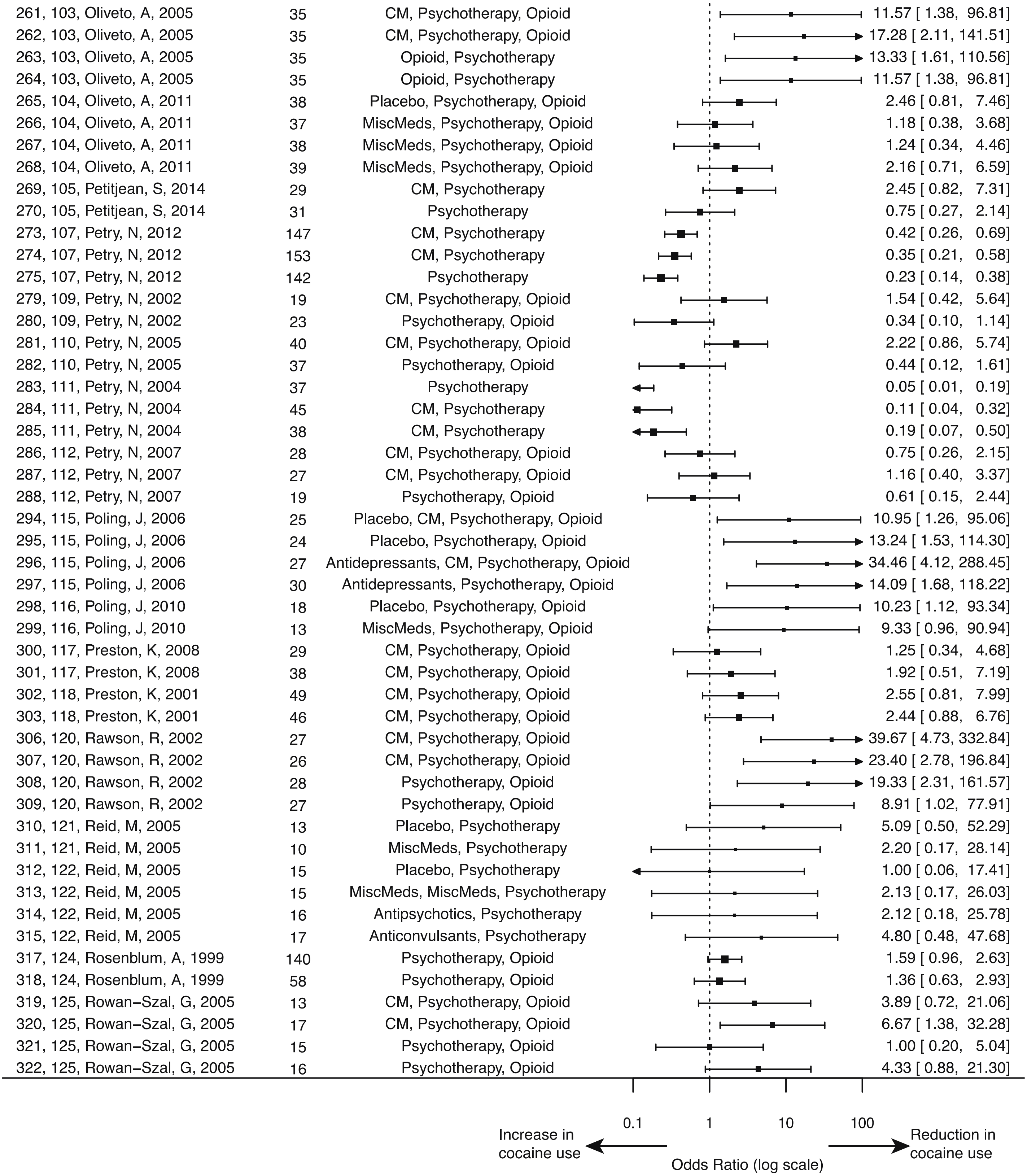

© 2021 Bentzley BS et al. *JAMA Network Open*.

Psychotherapy (5 of 6)

Group ID, Study ID, Author, Year	N	Treatment Categories	Odds Ratio [95% CI]
323, 126, Salloum, I, 2007	7	Anticonvulsants, Psychotherapy	1.00 [0.12, 8.31]
325, 128, Schmitz, J, 2001	34	Placebo, Psychotherapy	0.10 [0.02, 0.49]
326, 128, Schmitz, J, 2001	34	Antidepressants, Psychotherapy	0.32 [0.10, 1.03]
328, 129, Schmitz, J, 2008	31	Placebo, Psychotherapy	0.14 [0.02, 1.24]
329, 129, Schmitz, J, 2008	27	Placebo, Psychotherapy, CM	0.17 [0.02, 1.57]
331, 129, Schmitz, J, 2008	28	DA, Psychotherapy	0.46 [0.08, 2.75]
332, 129, Schmitz, J, 2008	23	DA, Psychotherapy, CM	1.00 [0.28, 3.51]
333, 130, Schottenfeld, R, 1997	28	Opioid, Psychotherapy	1.18 [0.38, 3.71]
334, 130, Schottenfeld, R, 1997	29	Opioid, Psychotherapy	0.54 [0.18, 1.62]
335, 130, Schottenfeld, R, 1997	30	Opioid, Psychotherapy	0.50 [0.15, 1.62]
336, 130, Schottenfeld, R, 1997	29	Opioid, Psychotherapy	0.16 [0.04, 0.67]
337, 131, Schottenfeld, R, 2005	40	CM, Psychotherapy, Opioid	2.22 [0.86, 5.74]
338, 131, Schottenfeld, R, 2005	39	CM, Psychotherapy, Opioid	1.18 [0.38, 3.65]
339, 131, Schottenfeld, R, 2005	40	Opioid, Psychotherapy	2.73 [0.92, 8.13]
340, 131, Schottenfeld, R, 2005	43	Opioid, Psychotherapy	1.36 [0.46, 4.06]
341, 132, Schubiner, H, 2002	24	Placebo, Psychotherapy	9.21 [1.04, 81.36]
342, 132, Schubiner, H, 2002	24	Psychostimulants, Psychotherapy	7.50 [0.83, 67.49]
349, 136, Shoptaw, S, 2002	35	Placebo, Psychotherapy	0.07 [0.02, 0.27]
350, 136, Shoptaw, S, 2002	34	DA, Psychotherapy	0.22 [0.08, 0.62]
351, 137, Shoptaw, S, 2008	33	Placebo, Psychotherapy	0.13 [0.04, 0.45]
352, 137, Shoptaw, S, 2008	37	Antidepressants, Psychotherapy	0.38 [0.13, 1.10]
353, 138, Shoptaw, S, 2004	12	Placebo, Psychotherapy	7.00 [1.04, 46.95]
354, 138, Shoptaw, S, 2004	11	MiscMeds, Psychotherapy	0.27 [0.02, 3.08]
355, 139, Shoptaw, S, 2005	15	Placebo, Psychotherapy	2.13 [0.17, 26.03]
356, 139, Shoptaw, S, 2005	15	DA, Psychotherapy	8.73 [0.92, 82.96]
357, 139, Shoptaw, S, 2005	15	DA, Psychotherapy	1.00 [0.06, 17.41]
358, 139, Shoptaw, S, 2005	15	MiscMeds, Psychotherapy	3.43 [0.32, 36.83]
359, 140, Shoptaw, S, 2003	35	Placebo, Psychotherapy	0.16 [0.05, 0.47]
360, 140, Shoptaw, S, 2003	35	MiscMeds, Psychotherapy	0.52 [0.19, 1.43]
361, 141, Shorter, D, 2013	13	Placebo, Psychotherapy	2.15 [0.17, 26.67]
362, 141, Shorter, D, 2013	8	MiscMeds, Psychotherapy	2.25 [0.17, 29.77]
363, 141, Shorter, D, 2013	9	MiscMeds, Psychotherapy	3.75 [0.32, 43.31]
364, 142, Sigmon, S, 2004	14	CM, Psychotherapy, Opioid	15.00 [2.26, 99.64]
365, 142, Sigmon, S, 2004	13	CM, Psychotherapy, Opioid	4.71 [0.73, 30.28]
366, 142, Sigmon, S, 2004	15	Psychotherapy, Opioid	4.57 [0.90, 23.14]
367, 143, Silverman, K, 2007	28	CM, Psychotherapy, Opioid	6.16 [1.19, 31.82]
368, 143, Silverman, K, 2007	28	Psychotherapy, Opioid	2.17 [0.36, 12.92]
369, 144, Silverman, K, 2004	26	CM, Psychotherapy, Opioid	7.50 [2.01, 28.05]
370, 144, Silverman, K, 2004	26	Psychotherapy, Opioid	4.55 [0.47, 43.78]
373, 146, Silverman, K, 1998	20	CM, Psychotherapy, Opioid	3.78 [0.83, 17.25]
374, 146, Silverman, K, 1998	20	CM, Psychotherapy, Opioid	6.93 [1.53, 31.38]
375, 146, Silverman, K, 1998	19	Psychotherapy, Opioid	2.27 [0.36, 14.19]
376, 147, Silverman, K, 1996	19	CM, Psychotherapy, Opioid	18.18 [2.05, 161.38]
377, 147, Silverman, K, 1996	18	Psychotherapy, Opioid	2.12 [0.18, 25.78]
378, 148, Silverman, K, 1999	29	CM, Psychotherapy, Opioid	18.95 [2.28, 157.76]
379, 148, Silverman, K, 1999	29	Psychotherapy, Opioid	2.07 [0.18, 24.07]
380, 148, Silverman, K, 1999	29	CM, Psychotherapy, Opioid	5.77 [0.63, 52.61]

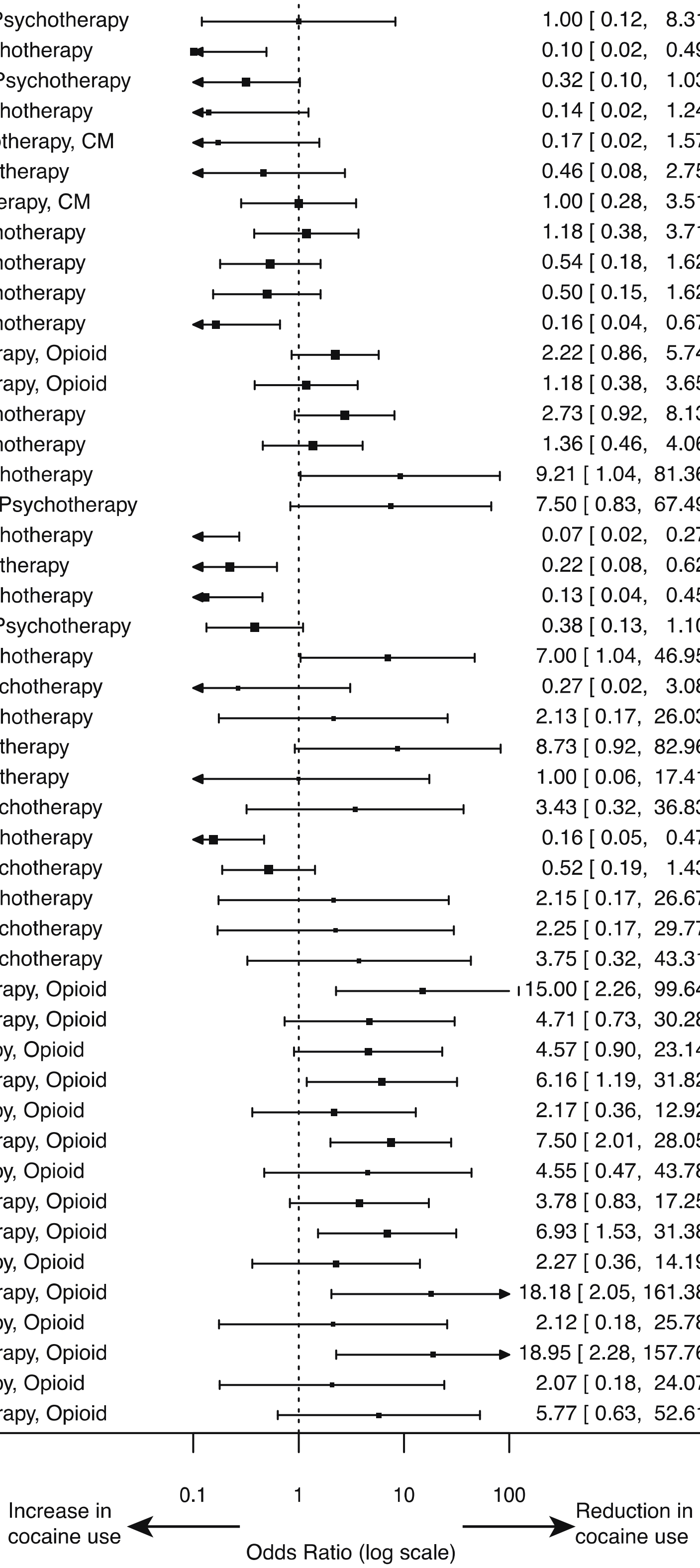

© 2021 Bentzley BS et al. *JAMA Network Open*.

Psychotherapy (6 of 6)

Group ID, Study ID, Author, Year	N	Treatment Categories	Odds Ratio [95% CI]
381, 149, Sofuoglu, M, 2017	34	Placebo, Psychotherapy, Opioid	17.50 [2.13, 143.64]
382, 149, Sofuoglu, M, 2017	37	MiscMeds, Psychotherapy, Opioid	40.00 [4.98, 320.98]
383, 149, Sofuoglu, M, 2017	35	MiscMeds, Psychotherapy, Opioid	21.91 [2.70, 178.08]
384, 150, Sofuoglu, M, 2007	15	Placebo, Psychotherapy, Opioid	8.73 [0.92, 82.96]
385, 150, Sofuoglu, M, 2007	30	MiscMeds, Psychotherapy, Opioid	8.68 [1.00, 75.30]
386, 151, Somoza, E, 2013	94	Placebo, Psychotherapy	6.33 [0.75, 53.64]
387, 151, Somoza, E, 2013	92	Anticonvulsants, Psychotherapy	8.65 [1.06, 70.61]
388, 152, Tapp, A, 2015	31	Placebo, Psychotherapy	0.13 [0.03, 0.52]
389, 152, Tapp, A, 2015	29	Antipsychotics, Psychotherapy	0.80 [0.21, 2.98]
396, 155, Wardle, M, 2017	40	Placebo, CM, Psychotherapy	1.89 [0.61, 5.82]
397, 155, Wardle, M, 2017	45	DA, CM, Psychotherapy	1.36 [0.46, 4.03]
398, 156, Weinstein, S, 1997	144	Psychotherapy	0.18 [0.11, 0.32]
399, 156, Weinstein, S, 1997	142	Psychotherapy	0.01 [0.00, 0.05]
400, 156, Weinstein, S, 1997	137	Psychotherapy	0.01 [0.00, 0.05]
401, 157, Winhusen, T, 2007	71	Placebo, Psychotherapy	0.54 [0.20, 1.46]
402, 157, Winhusen, T, 2007	70	Anticonvulsants, Psychotherapy	1.00 [0.39, 2.58]
RE Model for All Studies (N = 10818, Q = 1159.82, df = 258, p = 2.40e–114; I^2 = 82.8%)			1.62 [1.32, 1.98]

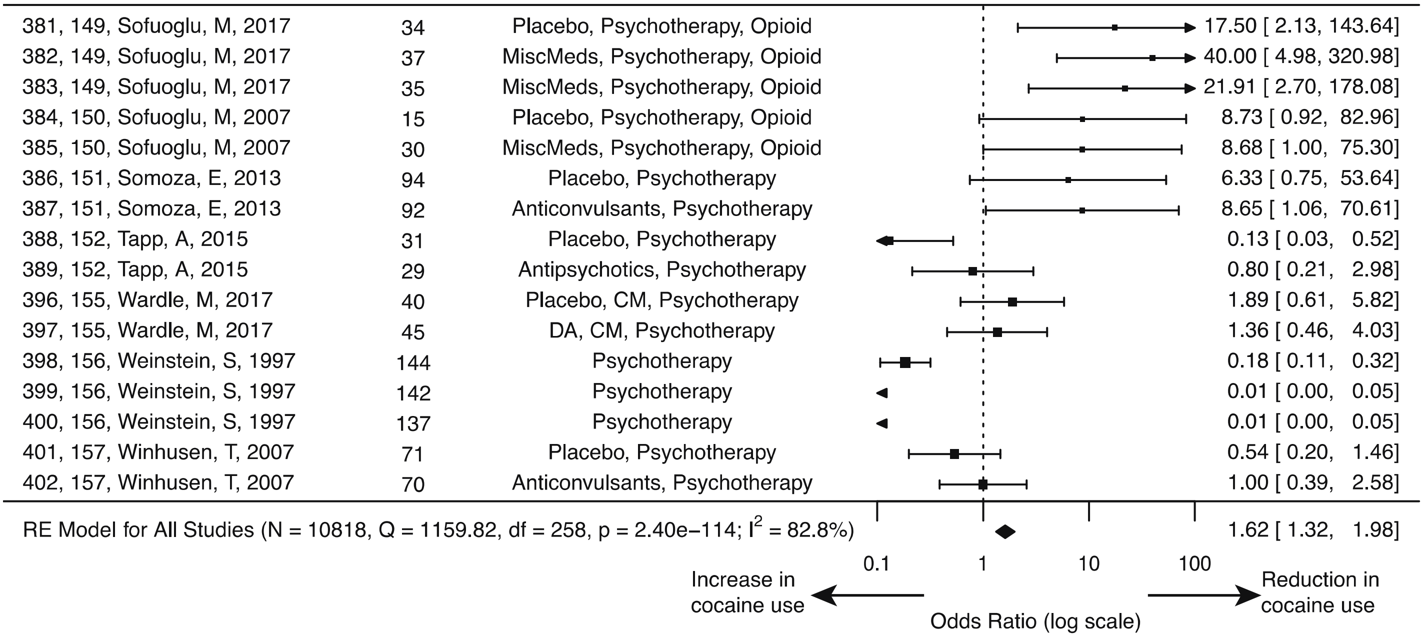

© 2021 Bentzley BS et al. *JAMA Network Open*.

eFigure 12. Funnel Plots (Full Models)

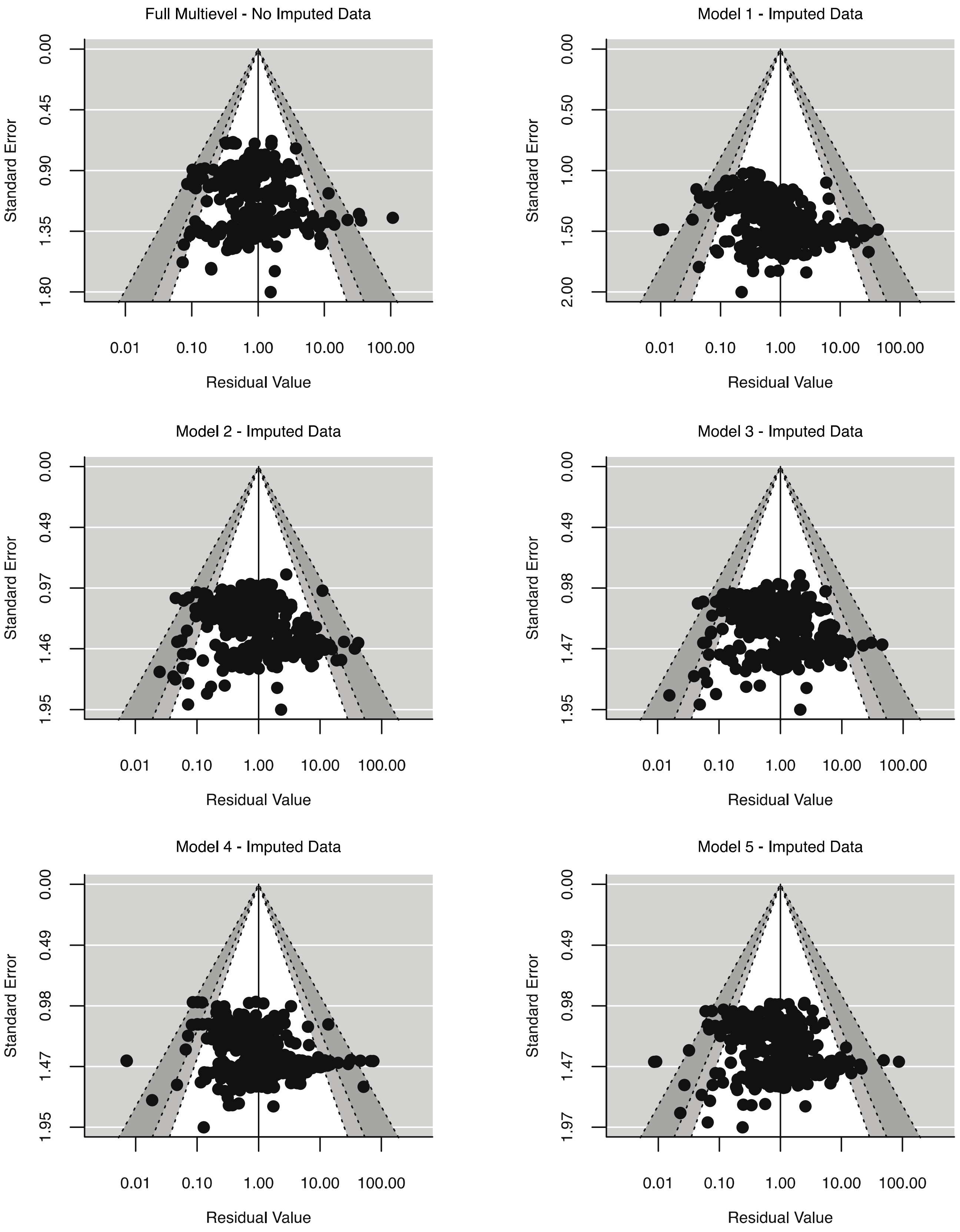

© 2021 Bentzley BS et al. *JAMA Network Open*.

eFigure 13. Funnel Plots (By Treatment Category)

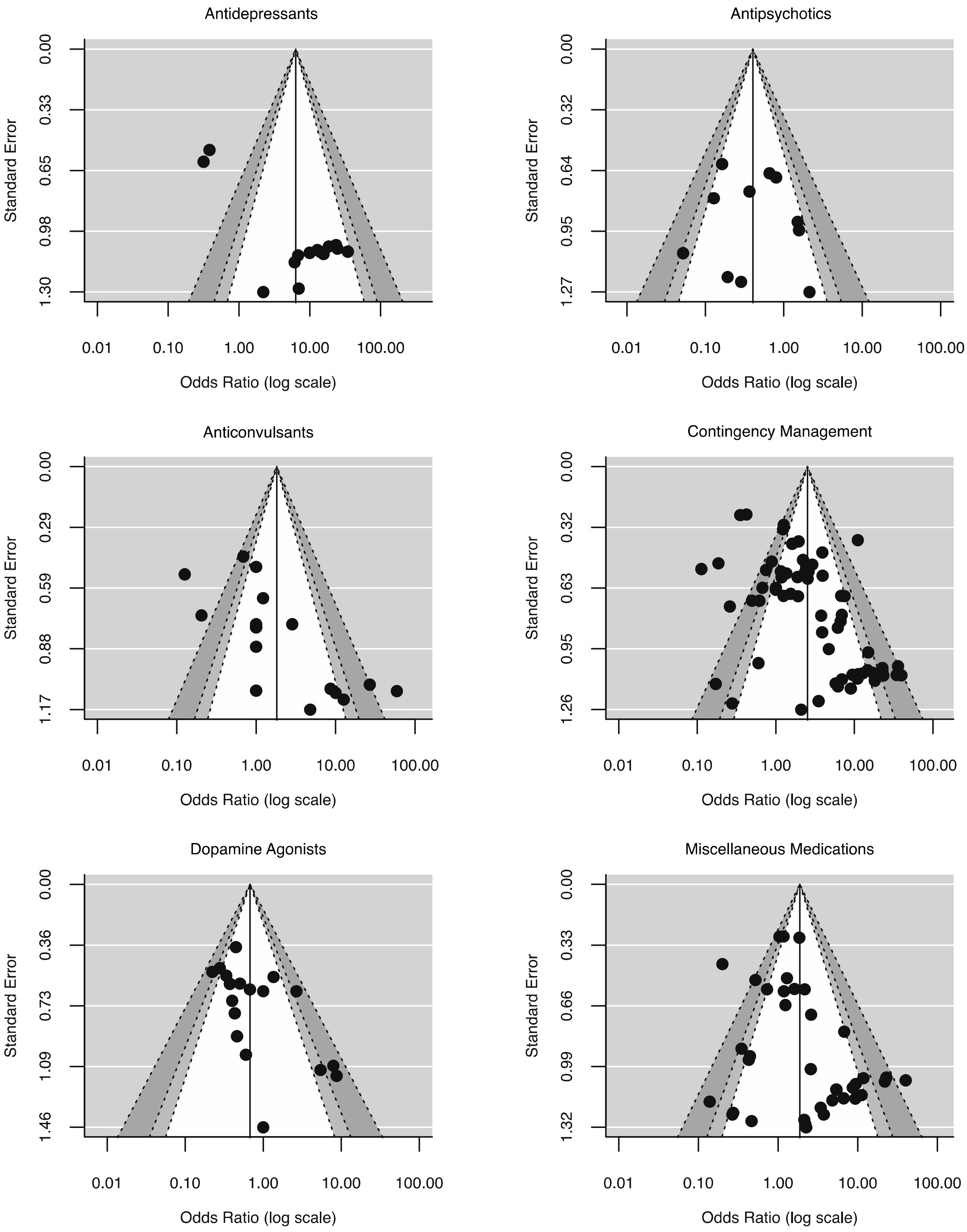

© 2021 Bentzley BS et al. *JAMA Network Open*.

Funnel Plots (By Treatment Category)

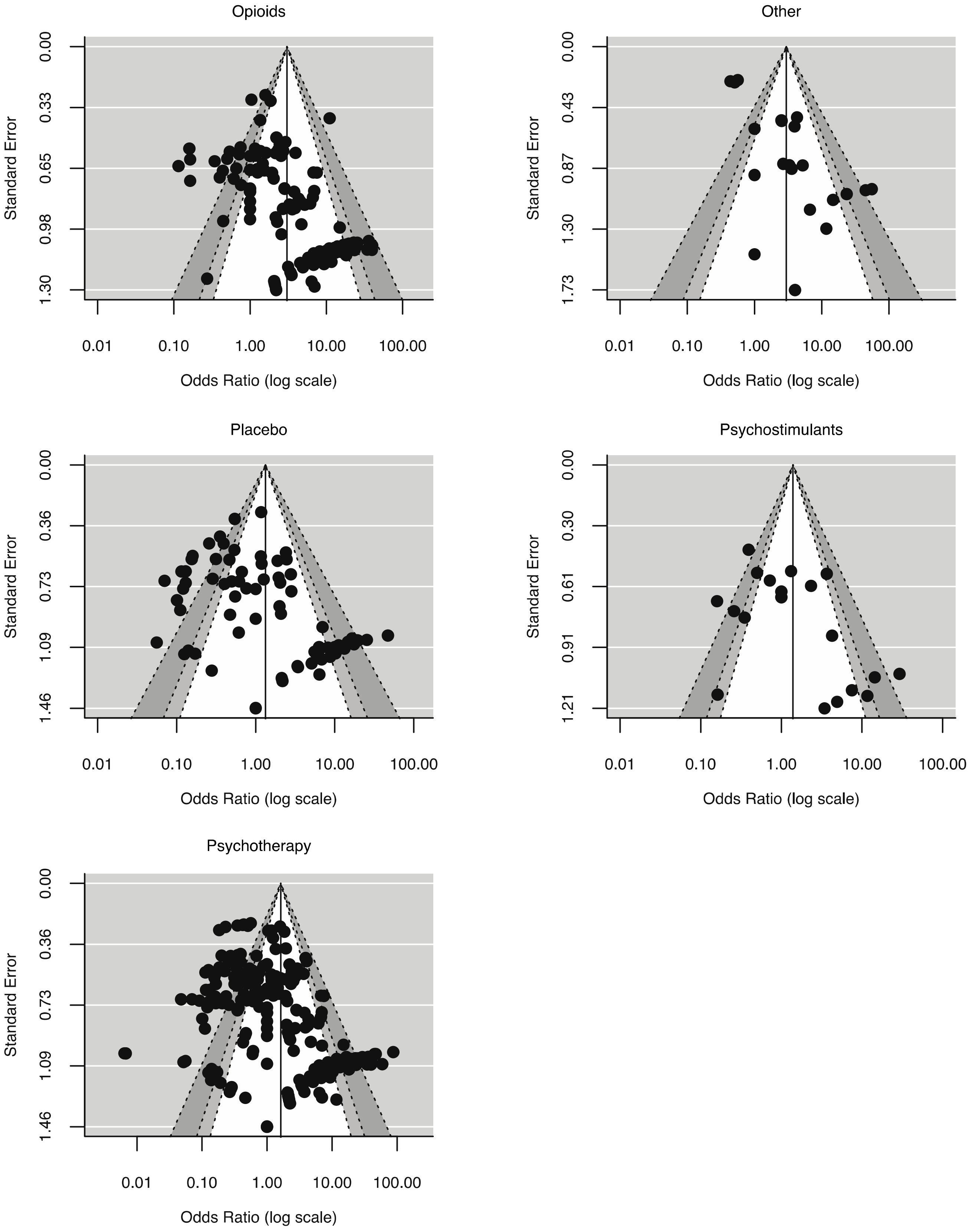

© 2021 Bentzley BS et al. *JAMA Network Open*.

eReferences

1. Rowan-Szal GA, Bartholomew NG, Chatham LR, Simpson DD. A combined cognitive and behavioral intervention for cocaine-using methadone clients. *J Psychoactive Drugs*. 2005;37(1):75-84.
2. Rawson RA, Huber A, McCann M, et al. A comparison of contingency management and cognitive-behavioral approaches during methadone maintenance treatment for cocaine dependence. *Arch Gen Psychiatry*. 2002;59(9):817-824.
3. Avants SK, Margolin A, DePhilippis D, Kosten TR. A comprehensive pharmacologic-psychosocial treatment program for HIV-seropositive cocaine- and opioid-dependent patients. preliminary findings. *J Subst Abuse Treat*. 1998;15(3):261-265.
4. Malcolm R, LaRowe S, Cochran K, et al. A controlled trial of amlodipine for cocaine dependence: a negative report. *J Subst Abuse Treat*. 2005;28(2):197-204.
5. Covi L, Hess JM, Schroeder JR, Preston KL. A dose response study of cognitive behavioral therapy in cocaine abusers. *J Subst Abuse Treat*. 2002;23(3):191-197.
6. Kampman KM, Dackis C, Lynch KG, et al. A double-blind, placebo-controlled trial of amantadine, propranolol, and their combination for the treatment of cocaine dependence in patients with severe cocaine withdrawal symptoms. *Drug Alcohol Depend*. 2006;85(2):129-137.
7. Dackis CA, Kampman KM, Lynch KG, Pettinati HM, O'Brien CP. A double-blind, placebo-controlled trial of modafinil for cocaine dependence. Neuropsychopharmacology. 2005;30(1):205-211.
8. Dackis CA, Kampman KM, Lynch KG, et al. A double-blind, placebo-controlled trial of modafinil for cocaine dependence. *J Subst Abuse Treat*. 2012;43(3):303-312.
9. Winhusen T, Somoza E, Ciraulo DA, et al. A double-blind, placebo-controlled trial of tiagabine for the treatment of cocaine dependence. *Drug Alcohol Depend*. 2007;91(2-3):141-148.
10. Somoza EC, Winship D, Gorodetzky CW, et al. A multisite, double-blind, placebo-controlled clinical trial to evaluate the safety and efficacy of vigabatrin for treating cocaine dependence. *JAMA Psychiatry*. 2013;70(6):630-637.
11. Kampman KM, Pettinati H, Lynch KG, Sparkman T, O'Brien CP. A pilot trial of olanzapine for the treatment of cocaine dependence. *Drug Alcohol Depend*. 2003;70(3):265-273.
12. Kampman KM, Pettinati H, Lynch KG, et al. A pilot trial of topiramate for the treatment of cocaine dependence. *Drug Alcohol Depend*. 2004;75(3):233-240.
13. Reid MS, Angrist B, Baker S, et al. A placebo-controlled screening trial of celecoxib for the treatment of cocaine dependence. *Addiction*. 2005;100(Suppl 1):32-42.
14. Reid MS, Casadonte P, Baker S, et al. A placebo-controlled screening trial of olanzapine, valproate, and coenzyme Q10/L-carnitine for the treatment of cocaine dependence. *Addiction*. 2005;100(Suppl 1):43-57.
15. Johnson BA, Roache JD, Ait-Daoud N, et al. A preliminary randomized, double-blind, placebo-controlled study of the safety and efficacy of ondansetron in the treatment of cocaine dependence. *Drug Alcohol Depend*. 2006;84(3):256-263.
16. Avants SK, Margolin A, Holford TR, Kosten TR. A randomized controlled trial of auricular acupuncture for cocaine dependence. *Arch Intern Med*. 2000;160(15):2305-2312.
17. Halikas JA, Crosby RD, Pearson VL, Graves NM. A randomized double-blind study of carbamazepine in the treatment of cocaine abuse. *Clin Pharmacol Ther*. 1997;62(1):89-105.
18. Petry NM, Barry D, Alessi SM, Rounsaville BJ, Carroll KM. A randomized trial adapting contingency management targets based on initial abstinence status of cocaine-dependent patients. *J Consul Clin Psychol*. 2012;80(2):276-285.
19. Silverman K, Wong CJ, Needham M, et al. A randomized trial of employment-based reinforcement of cocaine abstinence in injection drug users. *J Appl Behav Anal*. 2007;40(3):387-410.
20. Silverman K, Robles E, Mudric T, Bigelow GE, Stitzer ML. A randomized trial of long-term reinforcement of cocaine abstinence in methadone-maintained patients who inject drugs. *J Consult Clin Psychol*. 2004;72(5):839-854.
21. Petitjean SA, Dursteler-MacFarland KM, Krokar MC, et al. A randomized, controlled trial of combined cognitive-behavioral therapy plus prize-based contingency management for cocaine dependence. *Drug Alcohol Depend*. 2014;145:94-100.
22. Dursteler-MacFarland KM, Farronato NS, Strasser J, et al. A randomized, controlled, pilot trial of methylphenidate and cognitive-behavioral group therapy for cocaine dependence in heroin prescription. *J Clin Psychopharmacol*. 2013;33(1):104-108.

© 2021 Bentzley BS et al. *JAMA Network Open*.

23. Brown ES, Peterson Todd J, Hu LT, et al. A randomized, double-blind, placebo-controlled trial of citicoline for cocaine dependence in bipolar I disorder. *Am J Psychiatry*. 2015;172(10):1014-1021.
24. Shoptaw S, Kintaudi PC, Charuvastra C, Ling W. A screening trial of amantadine as a medication for cocaine dependence. *Drug Alcohol Depend*. 2002;66(3):217-224.
25. Margolin A, Kleber HD, Avants SK, et al. Acupuncture for the treatment of cocaine addiction: a randomized controlled trial. *JAMA*. 2002;287(1):55-63.
26. Malcolm R, Herron J, Sutherland SE, Brady KT. Adverse outcomes in a controlled trial of pergolide for cocaine dependence. *J Addict Dis*. 2001;20(1):81-92.
27. Grabowski J, Rhoades H, Stotts A, et al. Agonist-like or antagonist-like treatment for cocaine dependence with methadone for heroin dependence: two double-blind randomized clinical trials. *Neuropsychopharmacology*. 2004;29(5):969-981.
28. Wardle MC, Vincent JN, Suchting R, Green CE, Lane SD, Schmitz JM. Anhedonia is associated with poorer outcomes in contingency management for cocaine use disorder. *J Subst Abuse Treat*. 2017;72:32-39.
29. Beresford TP, Clapp L, Martin B, Wiberg JL, Alfers J, Beresford HF. Aripiprazole in schizophrenia with cocaine dependence: a pilot study. *J Clin Psychopharmacol*. 2005;25(4):363-366.
30. Walsh SL, Middleton LS, Wong CJ, et al. Atomoxetine does not alter cocaine use in cocaine dependent individuals: double blind randomized trial. *Drug Alcohol Depend*. 2013;130(1-3):150-157.
31. Donlin WD, Knealing TW, Needham M, Wong CJ, Silverman K. Attendance rates in a workplace predict subsequent outcome of employment-based reinforcement of cocaine abstinence in methadone patients. *J Appl Behav Anal*. 2008;41(4):499-516.
32. Monti PM, Rohsenow DJ, Michalec E, Martin RA, Abrams DB. Brief coping skills treatment for cocaine abuse: substance use outcomes at three months. *Addiction*. 1997;92(12):1717-1728.
33. Silverman K, Wong CJ, Umbricht-Schneiter A, Montoya ID, Schuster CR, Preston KL. Broad beneficial effects of cocaine abstinence reinforcement among methadone patients. *J Consult Clin Psychol*. 1998;66(5):811-824.
34. Handelsman L, Rosenblum A, Palij M, et al. Bromocriptine for cocaine dependence. a controlled clinical trial. *Am J Addict*. 1997;6(1):54-64.
35. Gorelick DA, Wilkins JN. Bromocriptine treatment for cocaine addiction: association with plasma prolactin levels. *Drug Alcohol Depend*. 2006;81(2):189-195.
36. Schottenfeld RS, Pakes JR, Oliveto A, Ziedonis D, Kosten TR. Buprenorphine vs methadone maintenance treatment for concurrent opioid dependence and cocaine abuse. *Arch Gen Psychiatry*. 1997;54(8):713-720.
37. Shoptaw S, Heinzerling KG, Rotheram-Fuller E, et al. Bupropion hydrochloride versus placebo, in combination with cognitive behavioral therapy, for the treatment of cocaine abuse/dependence. *J Addict Dis*. 2008;27(1):13-23.
38. Sofuoglu M, Poling J, Babuscio, et al. Carvedilol does not reduce cocaine use in methadone-maintained cocaine users. *J Subst Abuse Treat*. 2017;73:63-69.
39. Gonzalez G, Desai R, Sofuoglu M, et al. Clinical efficacy of gabapentin versus tiagabine for reducing cocaine use among cocaine dependent methadone-treated patients. *Drug Alcohol Depend*. 2007;87(1):1-9.
40. Mancino MJ, McGaugh J, Chopra MP, et al. Clinical efficacy of sertraline alone and augmented with gabapentin in recently abstinent cocaine-dependent patients with depressive symptoms. *J Clin Psychopharmacol*. 2014;34(2):234-239.
41. Sigmon SC, Correia CJ, Stitzer ML. Cocaine abstinence during methadone maintenance: effects of repeated brief exposure to voucher-based reinforcement. *Exp Clin Psychopharmacol*. 2004;12(4):269-275.
42. Sayers SL, Campbell EC, Kondrich J, et al. Cocaine abuse in schizophrenic patients treated with olanzapine versus haloperidol. *J Nerv Ment Dis*. 2005;193(6):379-386.
43. Martell BA, Orson FM, Poling J, et al. Cocaine vaccine for the treatment of cocaine dependence in methadone-maintained patients: a randomized, double-blind, placebo-controlled efficacy trial. *Arch Gen Psychiatry*. 2009;66(10):1116-1123.
44. Haney M, Gunderson EW, Jian H, Collins ED, Foltin RW. Cocaine-specific antibodies blunt the subjective effects of smoked cocaine in humans. *Biol Psychiatry*. 2010;67(1):59-65.
45. Epstein DH, Hawkins WE, Covi L, Umbricht A, Preston KL. Cognitive-behavioral therapy plus contingency management for cocaine use: findings during treatment and across 12-month follow-up. *Psychol Addict Behav*. 2003;17(1):73-82.
46. Higgins ST, Sigmon SC, Wong CJ, et al. Community reinforcement therapy for cocaine-dependent outpatients. *Arch Gen Psychiatry*. 2003;60(10):1043-1052.

© 2021 Bentzley BS et al. *JAMA Network Open*.

47. Miguel AQC, Madruga CS, Cogo-Moreira H, et al. Contingency management is effective in promoting abstinence and retention in treatment among crack cocaine users in Brazil: a randomized controlled trial. *Psychol Addict Behav*. 2016;30(5):536-543.
48. Goncalves-Ferreira A, do Couto FS, Campos AR, Neto LPL, Goncalves-Ferreira D, Teixeira J. Deep brain stimulation for refractory cocaine dependence. *Biol Psychiatry*. 2016;79(11):e87-e89.
49. Kosten T, Oliveto A, Feingold A, et al. Desipramine and contingency management for cocaine and opiate dependence in buprenorphine maintained patients. *Drug Alcohol Depend*. 2003;70(3):315-325.
50. Oliveto AH, Feingold A, Schottenfeld R, Jatlow P, Kosten TR. Desipramine in opioid-dependent cocaine abusers maintained on buprenorphine vs methadone. *Arch Gen Psychiatry*. 1999;56(9):812-820.
51. Grabowski J, Rhoades H, Schmitz J, et al. Dextroamphetamine for cocaine-dependence treatment: a double-blind randomized clinical trial. *J Clin Psychopharmacol*. 2001;21(5):522-526.
52. George TP, Chawarski MC, Pakes J, Carroll KM, Kosten TR, Schottenfeld RS. Disulfiram versus placebo for cocaine dependence in buprenorphine-maintained subjects: a preliminary trial. *Biol Psychiatry*. 2000;47(12):1080-1086.
53. Salloum IM, Douaihy A, Cornelius JR, Kirisci L, Kelly TM, Hayes J. Divalproex utility in bipolar disorder with co-occurring cocaine dependence: a pilot study. *Addict Behav*. 2007;32(2):410-415.
54. Schubiner H, Saules KK, Arfken CL, et al. Double-blind placebo-controlled trial of methylphenidate in the treatment of adult ADHD patients with comorbid cocaine dependence. *Exp Clin Psychopharmacol*. 2002;10(3):286-294.
55. Elkashef A, Fudala PJ, Gorgon L, et al. Double-blind, placebo-controlled trial of selegiline transdermal system (STS) for the treatment of cocaine dependence. *Drug Alcohol Depend*. 2006;85(3):191-197.
56. Licata SC, Penetar DM, Ravichandran C, et al. Effects of daily treatment with citicoline: a double-blind, placebo-controlled study in cocaine-dependent volunteers. *J Addict Med*. 2011;5(1):57-64.
57. Mooney ME, Herin DV, Schmitz JM, Moukaddam N, Green CE, Grabowski J. Effects of oral methamphetamine on cocaine use: a randomized, double-blind, placebo-controlled trial. *Drug Alcohol Depend*. 2009;101(1-2):34-41.
58. Kablinger AS, Lindner MA, Casso S, et al. Effects of the combination of metyrapone and oxazepam on cocaine craving and cocaine taking: a double-blind, randomized, placebo-controlled pilot study. *J Psychopharmacol*. 2012;26(7):973-981.
59. Oliveto A, Poling J, Sevarino KA, et al. Efficacy of dose and contingency management procedures in LAAM-maintained cocaine-dependent patients. *Drug Alcohol Depend*. 2005;79(2):157-165.
60. Baldacara L, Cogo-Moreira H, Parreira BL, et al. Efficacy of topiramate in the treatment of crack cocaine dependence: a double-blind, randomized, placebo-controlled trial. *J Clin Psychiatry*. 2016;77(3):398-406.
61. Dunn KE, Fingerhood M, Wong CJ, Svikis DS, Nuzzo P, Silverman K. Employment-based abstinence reinforcement following inpatient detoxification in HIV-positive opioid and/or cocaine-dependent patients. *Exp Clin Psychopharmacol*. 2014;22(1):75-85.
62. Rosenblum A, Magura S, Palij M, Foote J, Handelsman L, Stimmel B. Enhanced treatment outcomes for cocaine-using methadone patients. *Drug Alcohol Depend*. 1999;54(3):207-218.
63. McKee SA, Carroll KM, Sinha R, et al. Enhancing brief cognitive-behavioral therapy with motivational enhancement techniques in cocaine users. *Drug Alcohol Depend*. 2007;91(1):97-101.
64. Levin FR, Mariani JJ, Specker S, et al. Extended-release mixed amphetamine salts vs placebo for comorbid adult attention-deficit/hyperactivity disorder and cocaine use disorder: a randomized clinical trial. *JAMA Psychiatry*. 2015;72(6):593-602.
65. Schmitz JM, Averill P, Stotts AL, Moeller FG, Rhoades HM, Grabowski J. Fluoxetine treatment of cocaine-dependent patients with major depressive disorder. *Drug Alcohol Depend*. 2001;63(3):207-214.
66. Myrick H, Henderson S, Brady KT, Malcolm R. Gabapentin in the treatment of cocaine dependence: a case series. *J Clin Psychiatry*. 2001;62(1):19-23.
67. Milby JB, Schumacher JE, McNamara C, et al. Initiating abstinence in cocaine abusing dually diagnosed homeless persons. *Drug Alcohol Depend*. 2000;60(1):55-67.
68. Kosten TR, Oliveto A, Sevarino KA, Gonsai K, Feingold A. Ketoconazole increases cocaine and opioid use in methadone maintained patients. *Drug Alcohol Depend*. 2002;66(2):173-180.
69. Schmitz JM, Mooney ME, Moeller FG, Stotts AL, Green C, Grabowski J. Levodopa pharmacotherapy for cocaine dependence: choosing the optimal behavioral therapy platform. *Drug Alcohol Depend*. 2008;94(1-3):142-150.
70. Petry NM, Martin B. Low-cost contingency management for treating cocaine- and opioid-abusing methadone patients. *J Consult Clin Psychol*. 2002;70(2):398-405.

© 2021 Bentzley BS et al. *JAMA Network Open*.

71. Schottenfeld RS, Chawarski MC, Pakes JR, Pantalon MV, Carroll KM, Kosten TR. Methadone versus buprenorphine with contingency management or performance feedback for cocaine and opioid dependence. *Am J Psychiatry*. 2005;162(2):340-349.
72. Nuijten M, Blanken P, van den Brink W, Hendriks V. Modafinil in the treatment of crack-cocaine dependence in the Netherlands: results of an open-label randomised controlled feasibility trial. *J Psychopharmacol*. 2015;29(6):678-687.
73. Magura S, Rosenblum A, Lovejoy M, Handelsman L, Foote J, Stimmel B. Neurobehavioral treatment for cocaine-using methadone patients: a preliminary report. *J Addict Dis*. 1994;13(4):143-160.
74. Shoptaw S, Majewska MD, Wilkins J, Twitchell G, Yang X, Ling W. Participants receiving dehydroepiandrosterone during treatment for cocaine dependence show high rates of cocaine use in a placebo-controlled pilot study. *Exp Clin Psychopharmacol*. 2004;12(2):126-135.
75. Margolin A, Avants SK, Kosten TR. Pemoline for the treatment of cocaine dependence in methadone-maintained patients. *J Psychoactive Drugs*. 1996;28(3):301-304.
76. Kosten TR, Wu G, Huang W, et al. Pharmacogenetic randomized trial for cocaine abuse: disulfiram and dopamine β-hydroxylase. *Biol Psychiatry*. 2013;73(3):219-224.
77. Crosby RD, Pearson VL, Eller C, Winegarden T, Graves NL. Phenytoin in the treatment of cocaine abuse: a double-blind study. *Clin Pharmacol Ther*. 1996;59(4):458-468.
78. Shearer J, Wodak A, van Beek I, Mattick RP, Lewis J. Pilot randomized double blind placebo-controlled study of dexamphetamine for cocaine dependence. *Addiction*. 2003;98(8):1137-1141.
79. Mooney ME, Herin DV, Specker S, Babb D, Levin FR, Grabowski J. Pilot study of the effects of lisdexamfetamine on cocaine use: a randomized, double-blind, placebo-controlled trial. *Drug Alcohol Depend*. 2015;153:94-103.
80. Gilgun-Sherki Y, Eliaz RE, McCann DJ, et al. Placebo-controlled evaluation of a bioengineered, cocaine-metabolizing fusion protein, TV-1380 (AlbuBChE), in the treatment of cocaine dependence. *Drug Alcohol Depend*. 2016;166:13-20.
81. Petry NM, Martin B, Simcic F Jr. Prize reinforcement contingency management for cocaine dependence: integration with group therapy in a methadone clinic. *J Consult Clin Psychol*. 2005;73(2):354-359.
82. Petry NM, Tedford J, Austin M, Nich C, Carroll KM, Rounsaville BJ. Prize reinforcement contingency management for treating cocaine users: how low can we go, and with whom? *Addiction*. 2004;99(3):349-360.
83. Sofuoglu M, Poling J, Gonzalez G, Gonsai K, Oliveto A, Kosten TR. Progesterone effects on cocaine use in male cocaine users maintained on methadone: a randomized, double-blind, pilot study. *Exp Clin Psychopharmacol*. 2007;15(5):453-460.
84. Epstein DH, Schmittner J, Umbricht A, Schroeder JR, Moolchan ET, Preston KL. Promoting abstinence from cocaine and heroin with a methadone dose increase and a novel contingency. *Drug Alcohol Depend*. 2009;101(1-2):92-100.
85. Tapp A, Wood AE, Kennedy A, Sylvers P, Kilzieh N, Saxon AJ. Quetiapine for the treatment of cocaine use disorder. *Drug Alcohol Depend*. 2015;149:18-24.
86. Brown ES, Nejtek VA, Perantie DC, Bobadilla L. Quetiapine in bipolar disorder and cocaine dependence. *Bipolar Disord*. 2002;4(6):406-411.
87. Kirby KC, Carpenedo CM, Dugosh KL, et al. Randomized clinical trial examining duration of voucher-based reinforcement therapy for cocaine abstinence. *Drug Alcohol Depend*. 2013;132(3):639-645.
88. Weinstein SP, Gottheil E, Sterling RC. Randomized comparison of intensive outpatient vs. individual therapy for cocaine abusers. *J Addict Dis*. 1997;16(2):41-56.
89. Shoptaw S, Watson DW, Reiber C, et al. Randomized controlled pilot trial of cabergoline, hydergine and levodopa/carbidopa: Los Angeles Cocaine Rapid Efficacy Screening Trial (CREST). *Addiction*. 2005;100(Suppl 1):78-90.
90. Shoptaw S, Yang X, Rotheram-Fuller EJ, et al. Randomized placebo-controlled trial of baclofen for cocaine dependence: preliminary effects for individuals with chronic patterns of cocaine use. *J Clin Psychiatry*. 2003;64(12):1440-1448.
91. Preston KL, Ghitza UE, Schmittner JP, Schroeder JR, Epstein DH. Randomized trial comparing two treatment strategies using prize-based reinforcement of abstinence in cocaine and opiate users. *J Appl Behav Anal*. 2008;41(4):551-563.
92. Montoya ID, Gorelick DA, Preston KL, et al. Randomized trial of buprenorphine for treatment of concurrent opiate and cocaine dependence. *Clin Pharmacol Ther*. 2004;75(1):34-48.
93. Petry NM, Alessi SM, Hanson T, Sierra S. Randomized trial of contingent prizes versus vouchers in cocaine-using methadone patients. *J Consult Clin Psychol*. 2007;75(6):983-991.

© 2021 Bentzley BS et al. *JAMA Network Open*.

94. Ghitza UE, Epstein DH, Schmittner J, Vahabzadeh M, Lin JL, Preston KL. Randomized trial of prize-based reinforcement density for simultaneous abstinence from cocaine and heroin. *J Consult Clin Psychol*. 2007;75(5):765-774.
95. Oliveto A, Poling J, Mancino MJ, et al. Randomized, double blind, placebo-controlled trial of disulfiram for the treatment of cocaine dependence in methadone-stabilized patients. *Drug Alcohol Depend*. 2011;113(2-3):184-191.
96. Brodie JD, Case BG, Figueroa E, et al. Randomized, double-blind, placebo-controlled trial of vigabatrin for the treatment of cocaine dependence in Mexican parolees. *Am J Psychiatry*. 2009;166(11):1269-1277.
97. Grabowski J, Roache JD, Schmitz JM, Rhoades H, Creson D, Korszun A. Replacement medication for cocaine dependence: methylphenidate. *J Clin Psychopharmacol*. 1997;17(6):485-488.
98. Plebani JG, Lynch KG, Yu Q, Pettinati HM, O'Brien CP, Kampman KM. Results of an initial clinical trial of varenicline for the treatment of cocaine dependence. *Drug Alcohol Depend*. 2012;121(1-2):163-166.
99. Grabowski J, Rhoades H, Silverman P, et al. Risperidone for the treatment of cocaine dependence: randomized, double-blind trial. *J Clin Psychopharmacol*. 2000;20(3):305-310.
100. Jones HE, Johnson RE, Bigelow GE, Silverman K, Mudric T, Strain EC. Safety and efficacy of L-tryptophan and behavioral incentives for treatment of cocaine dependence: a randomized clinical trial. *Am J Addict*. 2004;13(5):421-437.
101. Mooney ME, Schmitz JM, Moeller FG, Grabowski J. Safety, tolerability and efficacy of levodopa-carbidopa treatment for cocaine dependence: two double-blind, randomized, clinical trials. *Drug Alcohol Depend*. 2007;88(2-3):214-223.
102. Preston KL, Umbricht A, Wong CJ, Epstein DH. Shaping cocaine abstinence by successive approximation. *J Consult Clin Psychol*. 2001;69(4):643-654.
103. Poling J, Oliveto A, Petry N, et al. Six-month trial of bupropion with contingency management for cocaine dependence in a methadone-maintained population. *Arch Gen Psychiatry*. 2006;63(2):219-228.
104. Maude-Griffin PM, Hohenstein JM, Humfleet GL, Reilly PM, Tusel DJ, Hall SM. Superior efficacy of cognitive-behavioral therapy for urban crack cocaine abusers: main and matching effects. *J Consult Clin Psychol*. 1998;66(5):832-837.
105. Silverman K, Higgins ST, Brooner RK, et al. Sustained cocaine abstinence in methadone maintenance patients through voucher-based reinforcement therapy. *Arch Gen Psychiatry*. 1996;53(5):409-415.
106. Shorter D, Lindsay JA, Kosten TR. The alpha-1 adrenergic antagonist doxazosin for treatment of cocaine dependence: a pilot study. *Drug Alcohol Depend*. 2013;131(1-2):66-70.
107. Katz EC, Robles-Sotelo E, Correia CJ, Silverman K, Stitzer ML, Bigelow G. The brief abstinence test: effects of continued incentive availability on cocaine abstinence. *Exp Clin Psychopharmacol*. 2002;10(1):10-17.
108. Liu S, Green CE, Lane SD, et al. The influence of dopamine β-hydroxylase gene polymorphism rs1611115 on levodopa/carbidopa treatment for cocaine dependence: a preliminary study. *Pharmacogenet Genomics*. 2014;24(7):370-373.
109. Poling J, Rounsaville B, Gonsai K, Severino K, Sofuoglu M. The safety and efficacy of varenicline in cocaine using smokers maintained on methadone: a pilot study. *Am J Addict*. 2010;19(5):401-408.
110. Holtyn AF, Koffarnus MN, DeFulio A, et al. The therapeutic workplace to promote treatment engagement and drug abstinence in out-of-treatment injection drug users: a randomized controlled trial. *Prev Med*. 2014;68:62-70.
111. Gonzalez G, Sevarino K, Sofuoglu M, et al. Tiagabine increases cocaine-free urines in cocaine-dependent methadone-treated patients: results of a randomized pilot study. *Addiction*. 2003;98(11):1625-1632.
112. Milby JB, Schumacher JE, Vuchinich RE, Freedman MJ, Kertesz S, Wallace D. Toward cost-effective initial care for substance-abusing homeless. *J Subst Abuse Treat*. 2008;34(2):180-191.
113. Brodie JD, Figueroa E, Dewey SL. Treating cocaine addiction: from preclinical to clinical trial experience with gamma-vinyl GABA. *Synapse*. 2003;50(3):261-265.
114. Levin FR, Evans SM, Brooks DJ, Garawi F. Treatment of cocaine dependent treatment seekers with adult ADHD: double-blind comparison of methylphenidate and placebo. *Drug Alcohol Depend*. 2007;87(1):20-29.
115. Focchi GRA, Leite MC, Andrade AG, Scivoletto S. Use of dopamine agonist pergolide in outpatient treatment of cocaine dependence. *Subst Use Misuse*. 2005;40(8):1169-1177.
116. Feingold A, Oliveto A, Schottenfeld R, Kosten TR. Utility of crossover designs in clinical trials: efficacy of desipramine vs. placebo in opioid-dependent cocaine abusers. *Am J Addict*. 2002;11(2):111-123.
117. Kosten TR, Domingo CB, Shorter D, et al. Vaccine for cocaine dependence: a randomized double-blind placebo-controlled efficacy trial. *Drug Alcohol Depend*. 2014;140:42-47.
118. Martell BA, Mitchell E, Poling J, Gonsai K, Kosten TR. Vaccine pharmacotherapy for the treatment of cocaine dependence. *Biol Psychiatry*. 2005;58(2):158-164.

119. Silverman K, Chutuape MA, Bigelow GE, Stitzer ML. Voucher-based reinforcement of cocaine abstinence in treatment-resistant methadone patients: effects of reinforcement magnitude. *Psychopharmacology (Berl)*. 1999;146(2):128-138.
120. Dallery J, Silverman K, Chutuape MA, Bigelow GE, Stitzer ML. Voucher-based reinforcement of opiate plus cocaine abstinence in treatment-resistant methadone patients: effects of reinforcer magnitude. *Exp Clin Psychopharmacol*. 2001;9(3):317-325.

© 2021 Bentzley BS et al. *JAMA Network Open*.
